# Cocatalytic Activity of the Furfuryl and Oxanorbornane-Substituted Guanidines in the Aldol Reaction Catalyzed by (*S*)-Proline

**DOI:** 10.3390/ijms25105570

**Published:** 2024-05-20

**Authors:** Luka Barešić, Monika Marijanović, Irena Dokli, Davor Margetić, Zoran Glasovac

**Affiliations:** 1 Centre for Nuclear Magnetic Resonance, Ruđer Bošković Institute, Bijenička cesta 54, 10000 Zagreb, Croatia; luka.baresic@irb.hr; 2 PLIVA d.d. Prilaz Baruna Filipovića 25, 10000 Zagreb, Croatia; monika.marijanovic@pliva.hr; 3 Division of Organic Chemistry and Biochemistry, Ruđer Bošković Institute, Bijenička cesta 54, 10000 Zagreb, Croatia; irena.dokli@irb.hr

**Keywords:** oxanorbornane, guanidinium salt, (*S*)-proline, aldol reaction, diastereoselectivity, enantioselectivity, DFT calculations

## Abstract

This work investigated the cocatalytic activity of recently prepared guanidinium salts containing an oxanorbornane subunit in an (*S*)-proline-catalyzed aldol reaction. The activity was interpreted by the diastereoselectivity of the reaction (*anti*/*syn* ratio) and for the most interesting polycyclic guanidinium salt, the enantioselectivity of the reaction was determined. The results indicated a negative impact on the oxanorbornane unit if present as the flexible substituent. For most of the tested aldehydes, the best cocatalysts provided enantioselectivities above 90% and above 95% at room temperature and 0 °C, respectively, culminating in >99.5% for 4–chloro– and 2–nitrobenzaldehyde as the substrate. The barriers for forming four possible enantiomers were calculated and the results for two *anti–*enantiomers are qualitatively consistent with the experiment. Obtained results suggest that the representatives of furfurylguanidinium and rigid polycyclic oxanorbornane-substituted guanidinium salts are good lead structures for developing new cocatalysts by tuning the chemical space around the guanidine moiety.

## 1. Introduction

Guanidine is well known for its high basicity (“superbasicity”) (^H2O^p*K*_a_ = 13.6) [1] and high tendency to form hydrogen bonds [2]. Due to these properties and a variety of synthetic approaches [3,4,5], guanidine compounds were recognized as easily available and powerful neutral organic bases and organocatalysts [3,4,6,7,8,9]. Guanidines can activate substrates by nucleophilic attack [10,11], but a more common and generally accepted interpretation of the catalytic cycle involves proton transfer to the guanidine subunit and subsequent formation of the complex between a guanidinium cation and one or two substrates acting as bifunctional catalyst in the latter case [6,7]. The structure of such a hydrogen-bonded intermediate with α-nitrotoluene was proven by X-ray crystallography [12]. In this respect, cyclic guanidines are preferred over acyclic analogs due to the optimal directionality of two NH bonds which allows for the stronger complexation of the substrate [13]. One typical, highly active organocatalyst based on the guanidine subunit is 1,5,7-triazabicyclo[4.4.0]dec-1-ene (**TBD**). It showed high activity in the transesterification of vegetable oil [14], Michael reaction [15], aldol condensation [16], and Morita–Baylis–Hillman reaction [17], to mention only a few. A number of guanidine derivatives were also used as enantioselective catalysts with varying success [18,19,20,21].

Another approach is to employ guanidine derivatives as additives or auxiliary groups to modulate the performance of the organocatalytic system. Recent examples of guanidine usage as the cooperative binding site in enantioselective reactions were given by Al-Taie et al. [22] and Kondoh et al. [21]. While the enantioselectivities obtained in the latter example are quite good, the activity of the prolinatoguanidine catalysts in the former was rather poor. This was an unexpected result given the well-known catalytic efficiency of (*S*)-proline [23,24,25]. In 2011, Concellon, del Amo, and coworkers employed **TBD** salts as the additives that improved the enantioselectivities by guanidine-proline supramolecular interaction [26]. They obtained an increase in *e*.*e*. from 56% to above 90% at 0–3 °C with a peak performance of the *anti/syn* ratio of 97:3 and *e*.*e*.(*anti*) = 99% for 4-bromobenzaldehyde and cyclohexanone as the substrates. Dependence of the *e*.*e*. on the type of salt was also observed and the best results were obtained using the tetrafluoroborate anion as a counterion.

Generally, employing (*S*)-proline as an organocatalyst is hampered by its low solubility in the non-protic solvents and by the formation of an oxazolidinone intermediate (parasitic side-reaction) resulting often in low selectivity [26,27,28]. Emma et al. managed to overcome these issues by carefully adjusting the H_2_O/MeOH ratio [29]. However, high enantioselectivities were in some cases associated with low conversion and/or a moderate *anti*/*syn* ratio. The abovementioned approach of Concellon, del Amo, and coworkers [26] employs guanidines as additives, which form a supramolecular associate with the (*S*)-proline, increase the solubility of the (*S*)-proline, and modulate sterics and the substrate binding in the proposed transition state complex [26,30]. An equilibrium is also shifted toward enamines minimizing oxazolidinone formation [27]. Additionally, the p*K*_a_ of the proline carboxylic group is strongly increased upon complexation, which in turn can lead to a stronger activation of the carbonyl compounds by protonation [31]. Besides guanidines, other hydrogen bond-donating compounds like thioureas, diamides, and squaramides are also suitable cocatalysts [28].

Our ongoing interest in guanidine chemistry is primarily oriented toward the role of intramolecular hydrogen bonding on the basicity and application of novel derivatives as the homogenous catalysts in the conversion of waste cooking oil to biodiesel [32,33,34]. In 2022, we described a simple synthesis of a series of polycyclic oxanorbornanes containing a guanidine moiety by the cycloaddition/hydrogenation approach [35]. Preliminary calculations of their p*K*_a_ values indicated lower basicity (^ACN^p*K*_a_ = 19–24) relative to **TBD** (^ACN^p*K*_a_ = 26 [36]), and one could expect their limited applicability on the reactions with reactive substrates or as the additive. The latter is also supported by a general observation that more acidic protic compounds are also considered better hydrogen-bond donors [37,38]. Based on the findings described above, the aldol reaction catalyzed by (*S*)-proline is recognized as a suitable system for testing the cocatalytic activity of our guanidine derivatives.

**Figure 1:**
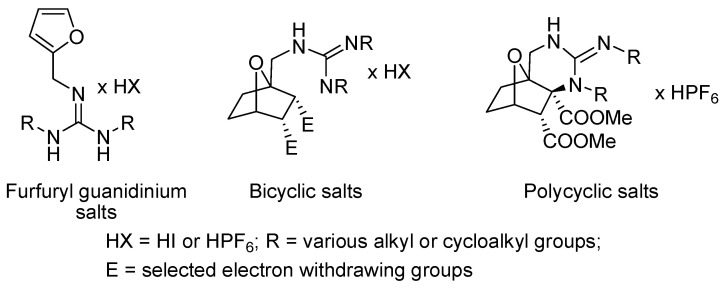
General structures of the three types of employed cocatalysts.

In this work, the results of the cocatalytic activity of three types of guanidinium salts in the (S)-proline-catalyzed direct aldol reaction are presented (Figure 1). Their common structural features are oxanorborna(e)ne and guanidine subunits. The comparison was made with respect to the simple furfuryl guanidines that were used as the precursors. We were interested to discover if the sterical demands of the oxanorbornane subunit would increase the selectivity of the reaction over simple guanidinium salts. The obtained results provide information on the role of several structural elements on the cocatalytic activity of the employed guanidinium salts. For most of the employed cocatalysts, the activity was expressed as the ratio of the diastereomers formed (*anti*:*syn*), while for the selected cocatalysts, an enantiomeric excess (*e*.*e*.) was also determined.

## 2. Results and Discussion

**Figure 2:**
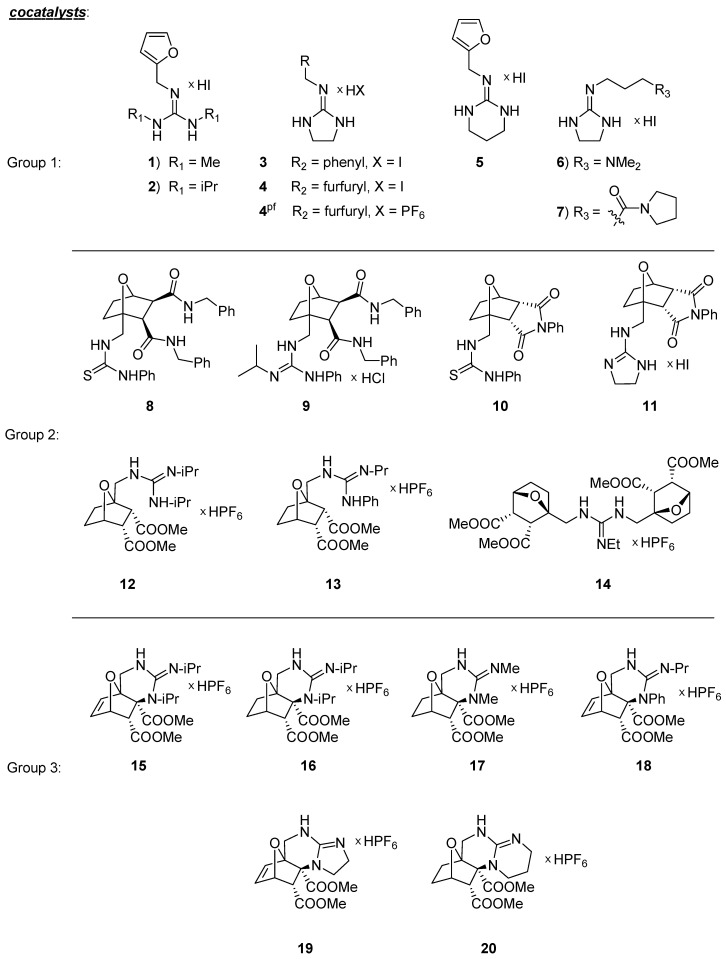
Schematic representation of guanidine salts used as cocatalysts in the model aldol reaction.

Guanidine cocatalysts used in this work are divided into three groups of derivatives according to their specific structural features (Figure 2). All salts were used as either hydroiodides or hexafluorophosphates except hydrochloride salt **9**. Hexafluorophosphates were used instead of tetrafluoroborates [26] due to their lower tendency toward hydrolysis [39] and hydrogen bonding [40].

Group 1 (**1**–**7**) of cocatalysts contains guanidines without an oxanorbornane subunit. Besides furfuryl and benzyl derivatives, 3–dimethylaminopropyl– and 3–(*N*-pyrrolidinocarbonyl)propyl- analogs (**6** and **7**) were synthesized and used to test the influence of the intramolecular hydrogen bond on the cocatalytic activity. One could expect that additional hydrogen bond-accepting functional groups will strongly affect the formation of the complex with (*S*)-proline and a substrate. Two scenarios are possible: *(i)* additional stabilization through the cooperative binding and *(ii)* inhibition of intermolecular interactions by the competitive intramolecular hydrogen bonding. In the context of the potential parasitic formation of oxazolidinone [27], an additional basic group present in **6** can also shift the equilibrium toward the enamine form.

Group 2 (**8**–**14**) contains an oxanorbornane subunit substituted with amide, imide, or ester groups in combination with phenylthiourea or guanidine groups mutually joined by a methylene spacer. Most of the cocatalysts are acyclic guanidine derivatives with high conformational freedom. Here, we should emphasize the *exo* configuration of the diamide derivatives **8** and **9** while the other derivatives contain an *endo*-oxanorbornane subunit. Thioureas were used as the nonionic analogs of guanidines to test the role of diamide and imide moieties.

Group 3 (**15**–**20**) encompasses representatives of the polycyclic guanidines formed by intramolecular cyclization of guanidine-substituted oxanorbornadienes [35]. Formed cyclic guanidine subunits imply low flexibility but the directionality of the NH bond varies strongly. Compounds **19** and **20** could be considered as the desymmetrized **TBD** analog having the desirable position and directionality of two NH bonds.

### 2.1. Cocatalytic Activity of the Guanidine Derivatives in the (S)-Proline-Catalyzed Aldol Reaction

**Scheme 1:**
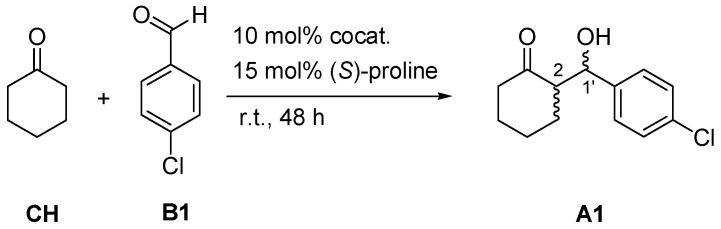
Aldol reaction used for testing the catalytic activity of the guanidinium salts. Cyclohexanone (**CH**) was used in 10-fold excess relative to **B1**. For the structure of cocatalysts, see Figure 2. Enumeration of the chiral centers was taken from Ref. [26].

Initially, all cocatalysts were tested at room temperature (18–20 °C) and their activity was interpreted according to the obtained *anti*/*syn* ratio of two possible diastereomeric products. The reaction is shown in Scheme 1 while the results are given in Table 1.

**Figure 3:**
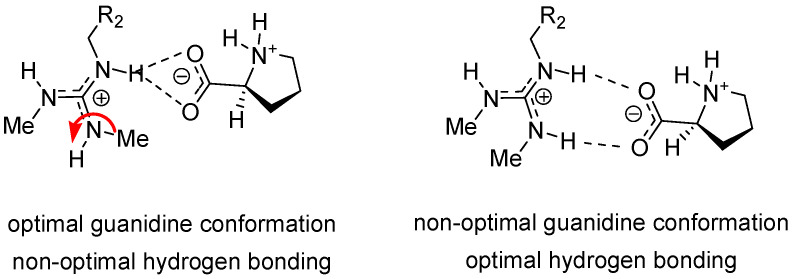
Possible motifs of the (*S*)–proline binding to two different conformations of guanidinium cation exemplified by the cation **1c** (R_2_ = 2-furyl).

As the initial test, the reaction was conducted at room temperature without the cocatalyst added (Table 1, entry 1). The obtained diastereomeric ratio was slightly higher than the literature data while the yield was slightly lower [26]. The addition of the cocatalysts belonging to group 1 mostly led to the preferential formation of the *anti* isomer in a >85% ratio. Somewhat surprising were the only slightly lower diastereomeric ratios obtained by the acyclic cocatalysts **1** and **2**. Their propeller-like conformation prevents optimal hydrogen bonding with a carboxylic group unless a conformational change by rotation around one C–N bond takes place (Figure 3). A comparison of Gibbs energies for the formation of the complexes after the guanidine isomerization indicated the endergonicity of the process by 9.4 (**1**) and 7.7 kJ mol^−1^ (**2**) (Table S1 in Supplementary Materials). The abundance of the associates in the mixture is expected to be low; nevertheless, a better solubility of (*S*)-proline was observed, which is most likely the reason for the increased selectivity and yield relative to the reaction with no guanidine added.

Usage of the cocatalysts **6** and **7** bearing dimethylamino and amide functionalities, respectively, led to lower selectivities. These substituents can form strong intramolecular hydrogen bonds enhancing the basicity [41,42]. On the other hand, a negative impact of these substituents on the proline binding due to the competition between the intra- and intermolecular hydrogen bonding could be expected. The calculations suggest their minimal impact on the energetics of the complex formation with (*S*)-proline (see Table S1 in Supplementary Materials). However, with the enamine, the active proton is either taken away by the NMe_2_ group present in the side chain of **6** or blocked by intramolecular hydrogen bonding with the amide subunit in **7** (see Table S2 in Supplementary Materials and the associated discussion). While the selectivity is lowered, high conversions and yields are kept, which could again be ascribed to the increased solubility of (*S*)-proline.

We should also emphasize that the obtained diastereoselectivities with the iodide salts of group 1 cocatalysts are comparable to the literature data for **TBD × HPF_6_** [26]. By switching from the iodide to hexafluorophosphate anion (Table 1, entries 5 and 6), further improvement in diastereoselectivity and a somewhat increased yield was obtained.

The influence of the oxanorbornane subunit was highly dependent on the structure. Most of the cocatalysts belonging to groups 2 and 3 provided slightly lower selectivities with improved yields. The exceptions are derivatives containing two benzylamide groups (**8** and **9**) which had very low solubility in the reaction media, and therefore low selectivities and yields were not surprising. The solubility problem is tackled by adding a certain amount of solvent (Table S7, Supplementary Materials). Indeed, all employed solvents (DCM, DMF, ACN, and MeOH) improved the solubility of the system. Adding methanol diminished the role of the cocatalyst while the aprotic solvents showed a small and mostly deteriorating effect on the reaction.

Incorporation of the guanidine subunit into the polycyclic skeleton was beneficial only for cocatalyst **20,** for which an *anti*/*syn* ratio of 92:8 was obtained with high conversion and very good yield. While the diastereoselectivity was similar to that obtained by cocatalyst **4^pf^**, the yield was significantly improved. The best result obtained for cocatalyst **20** was not surprising having in mind its structural similarity to **TBD**. Somewhat unexpected are the relatively poor results obtained for **19** in terms of both selectivity and yield. On the other hand, low selectivities obtained for the diisopropyl-substituted derivatives **15** and **16** were not unexpected. Namely, the sterical repulsion of two isopropyl groups most likely directs the exocyclic NH bond between them, leaving only one NH bond available to the proline for substrate binding. The same effect, albeit less pronounced, is most likely responsible for lowered selectivities of **17** and **18** as well. Here, we should emphasize that the polycyclic guanidine hexafluorophosphates **19** and **20** are more stable than **4^pf^** and show no visible physical change over one month when stored at room temperature. The salt **4^pf^** turns brownish after approximately one week even if stored in a refrigerator (+4 °C).

**Figure 4:**
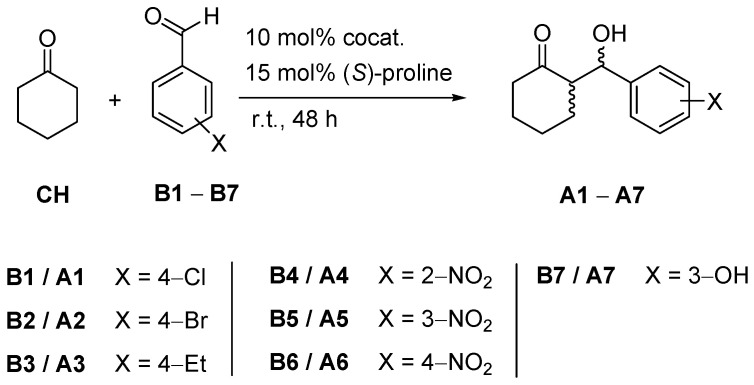
The pool of the aromatic aldehydes used to test the enantioselectivity of the (*S*)-proline/cocatalyst **20** system.

For cocatalyst **20,** a rather limited scope of reactions was tested and determined enantiomeric excess (*e.e.*). The reaction scheme and the pool of the employed aldehydes are shown in Figure 4.

Besides a quite good *anti*/*syn* ratio and yield, the aldol reaction of cyclohexanone and 4–chlorobenzaldehyde with (*S*)-proline as a catalyst and salt **20** as cocatalyst provided good enantioselectivity on the *anti* diastereomer. As expected, lowering the temperature to 0 °C improved the selectivity of the reaction up to an *ee* of 99% with a d.r. of 96:4 at the cost of slightly lower conversion and the isolated yield. The reaction conducted at 10 °C represents the balanced case where high diastereoselectivity, conversion, and yield were retained while the enantioselectivity is lowered to 95%. The addition of methanol drastically lowered the *anti*/*syn* ratio to the values commonly obtained in MeOH with other cocatalysts as well. The addition of MeOH also has a detrimental effect on *e.e.* A significant drop in the yield was obtained when the neutral guanidine **20n** (racemate) was used as a catalyst (Table 2, entry 5). The activity of **20** was also compared to **4^pf^** which was the best cocatalyst within Group 1 and had a comparable *anti*/*syn* ratio to **20** at room temperature (Table 1, entries 6 and 22). The slightly better performance of **20** in terms of conversion and yield was obtained at 0 °C. On the other hand, **4^pf^** induced a slightly better enantioselectivity of the reaction.

Switching from 4–chlorobenzaldehyde to 4–bromo (**B2**) and 4–ethyl (**B3**) derivatives reduced the diastereoselectivity but the enantioselectivity remained similar (**B3**) or even increased (**B2**) to 95% at room temperature. We could note in passing that the reaction with the aldehyde **B3** showed an unexpectedly low conversion of the reactants to products after 48 h. The best overall results were obtained with 2–nitrobenzaldehyde **B4** where the d.r. values were 94:6 and 96:4 at r.t. and 0 °C, respectively, with the *e.e.* of 98% even at room temperature. Other nitro-substituted benzaldehydes gave slightly poorer results with higher selectivities obtained for *para*-substituted benzaldehyde **B6**. Finally, we also tested benzaldehyde **B7** as the only representative having an OH group. The results are similar or worse in comparison to the reaction conducted with methanol (Table 2, entry 4) indicating a negative role of the proton-donating functional groups.

### 2.2. Modeling the Transition State Structures for the Formation of Enantiomers

**Figure 5:**
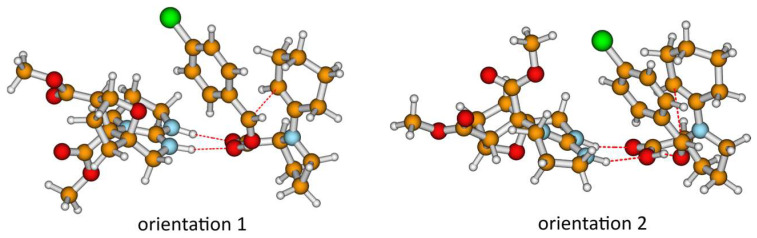
Two different orientations of the guanidinium cocatalyst **20p** with respect to enamine and the substrate exemplified for the structure **IIb**.

**Scheme 2:**
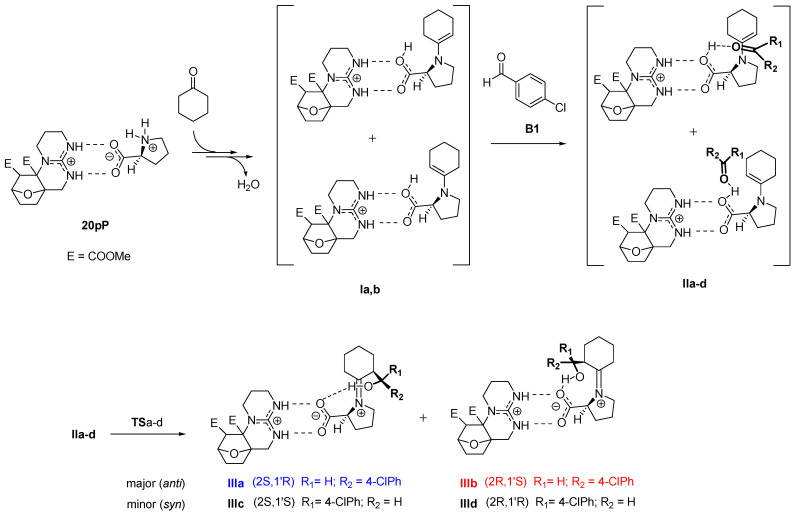
Selected reaction steps of the (*S*)-proline-catalyzed aldol reaction modeled by M06-2X DFT calculations. The benzaldehyde approach from above the enamine plane is assumed. The intermediate leading to the major and minor *anti* enantiomers in the mixture are color-coded blue and red, respectively. The catalytic cycle is presented in subsection S1.3 in Supplementary Materials.

To obtain a better insight into the role of the oxanorbornane skeleton, a computational investigation of several important steps along the reaction mechanism was conducted. The mechanism was theoretically analyzed by Coote and coworkers in detail [43]. In agreement with the previous proposition of the Zimmerman–Traxler type transition state (TS) (List-Houk model) [26,44,45,46], the results of Coote and coworkers supported CC bond formation as the step that controls the stereochemistry. The authors also concluded that two enamine conformers interconvert mutually due to the low-barrier rotation around the C-N bond [43]. While this step does not control the stereoselectivity, it allows for a population of the faster reaction sequence until irreversible product release occurs. An important feature of the proposed TS structure is hydrogen bonding between the proline carboxylic group and the aldehyde which, besides increasing the electrophilicity of the carbonyl group, also stabilizes the alkoxide formed [47]. With all this in mind, we chose a section of the reaction mechanism encompassing several stationary points as well as the transition state structures for the formation of all four possible isomers (Scheme 2). Here, we should emphasize that two orientations of the guanidine cocatalyst relative to enamine were considered (Figure 5). As the preferred orientation, the more stable conformation at the **IIa–d** stage was selected.

**Figure 6:**
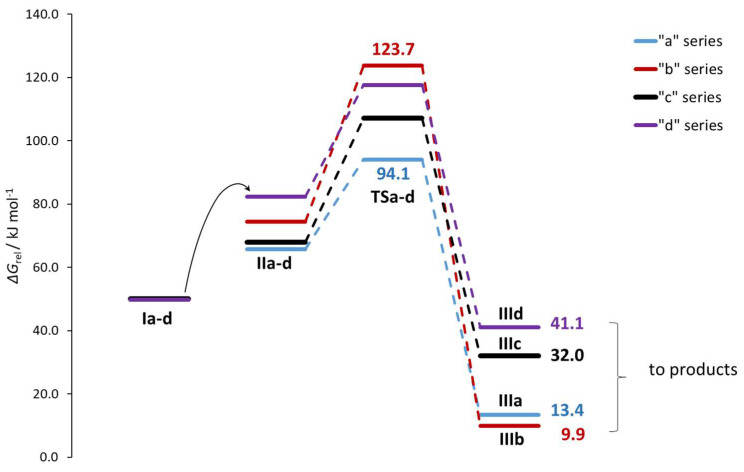
The relative Gibbs energies of several stationary points along the paths for the formation of 4 possible enantiomers in the tested aldol reaction (Scheme 2). The relative Gibbs energies (in kJ mol^−1^) were calculated against the separate components: 4-chlorobenzaldehyde (**B1**), (*S*)-proline, guanidinium cation **20p**, cyclohexanone, and water.

According to the literature, the major enantiomer produced in this reaction has a (2*S*,1′*R*)-configuration [26]. Assuming no change in the chirality during the hydrolytic liberation of the product, it would be a product of hydrolysis of the intermediate **IIIa**. Calculated relative Gibbs energies (Δ*G*_rel_) of the intermediates **Ia–d**, **IIa–d**, **IIIa–d**, and transition state structures **TSa–d** are graphically presented in Figure 6, while the tabulated data are given in Table S4 in Supplementary Materials.

Consistent with the experimental results, kinetically the most active reaction path is calculated to be the path “a” leading to the (2*S*,1′*R*) *anti-*enantiomer. The barrier along path “b” is the highest but leads to the most stable intermediate **IIIb**. The abundance of the (2*R*,1′*S*) enantiomer in the product mixture is lower than that of either of the *syn* enantiomers (Section S4, Supplementary Materials) which are in agreement with their calculated barrier heights. Here we should mention that the rotation of the guanidinum cocatalyst by 180 ° would lower the **TSb** by 1.2 kJ mol^−1^, which does not affect the relative trend in the barrier heights. Although simplified, the employed consideration of the origin of the enantioselectivity seems to be valid and is further supported by the data obtained for 2–nitrobenzaldehyde (**B4**, Figure 4) as the substrate. In this case, the relative energies of **TSa**(**B4**) and **TSb**(**B4**) amount to 94.5 and 128.8 kJ mol^−1^, respectively (Table S5 and Figure S4 in Supplementary Materials). In other words, the calculations predict a minimal effect on the formation of the major enantiomer but significantly slow down the production of the minor one. This, in turn, results in a higher estimated enantioselectivity when compared to 4-chlorobenzaldehyde. An additional comparison was performed with protonated 1,5,7-triazabicyclo[4.4.0]-dec-1-ene (**TBD**H^+^) as the guanidine cocatalyst used previously [26]. The difference in the barriers along the paths “a” and “b” amounts to 25.2 which is smaller than with guanidine **20** as a cocatalyst by ca 4 kJ mol^−1^ (Table S6 and Figure S5 in Supplementary Materials). Similarly to the other two cases, the barriers to the formation of the *syn* diastereomers fall in between these two paths.

## 3. Materials and Methods

**Scheme 3:**
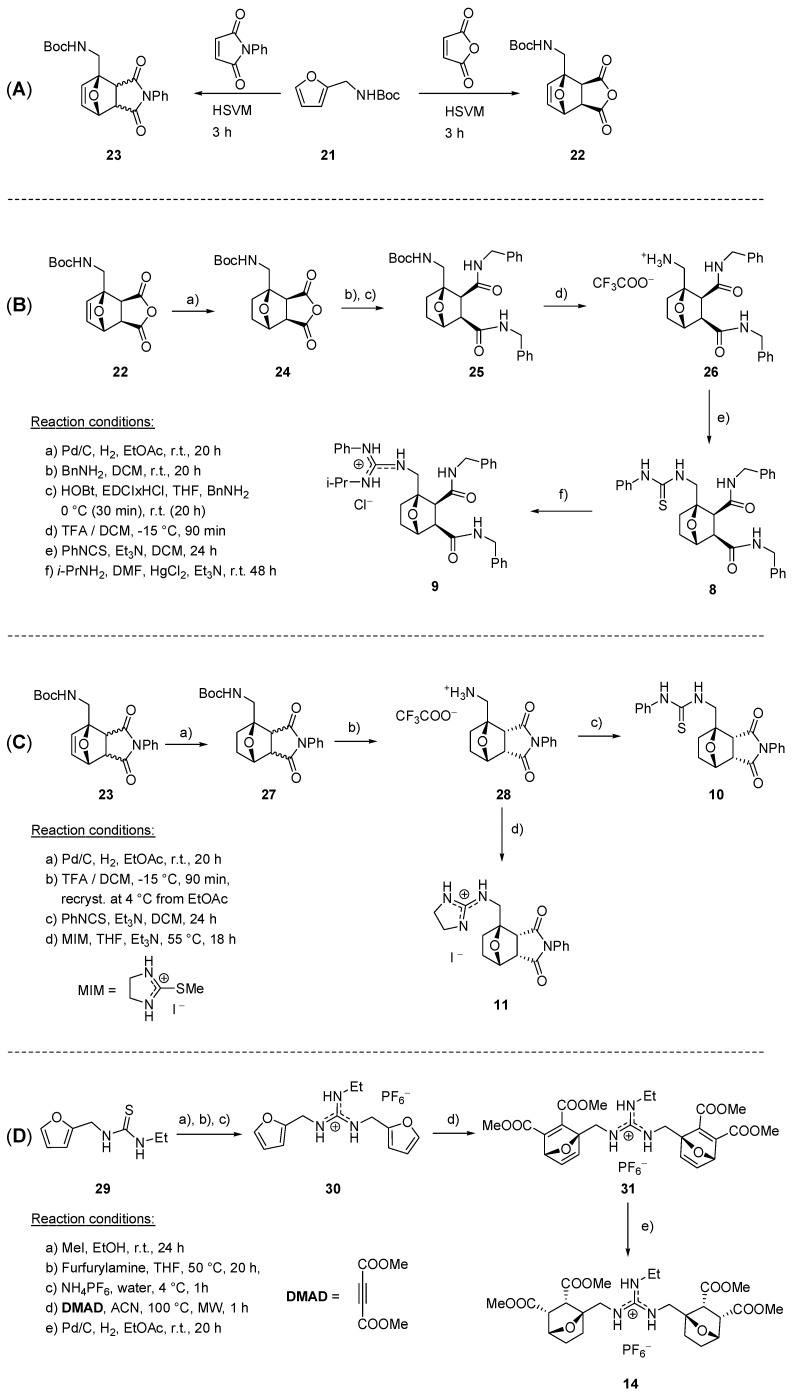
General scheme for the synthesis of the cocatalysts **9**–**11** and **14**.

*General remarks on the synthesis of the cocatalysts*: Furfurylguanidinium iodides **1–5** as well as hexafluorophosphate salts **4^pf^**, **12, 13,** and **16**–**20** were prepared as described earlier [35,48]. Salt **7** was prepared by converting the Boc-protected-γ-aminobutyric acid [49] to the 4-amino-1-(pyrrolidin-1-yl)butan-1-one hydrate followed by its guanidinylation with isothiouronium iodides according to the slightly modified general procedure described by Aoyagi and Endo [48]. The oxanorbornane-substituted thioureas **8** and **10** and guanidinium salts **9**, **11**, and **14** were prepared according to the reactions shown in Scheme 3. Derivative **15** was obtained by base-catalyzed cyclization of the corresponding oxanorbornadiene [35]. Cycloadditions of *N*-Boc-furfurylamine with maleic anhydride and *N*-phenylmaleimide were conducted mechanochemically [50] and the analytical data for adduct **22** are identical to those prepared according to the literature [51]. The details are given in the Supplementary Materials.

*General procedure of the cocatalysis*: An amount of 4 mmol of the freshly distilled cyclohexanone, 0.4 mmol of aldehyde **B1**–**B7**, 0.06 mmol (15 mol%) of (*S*)-proline, and 0.04 mmol (10 mol%) of guanidinium salt were stirred under conditions described in Table 1 or Table 2. After the desired time, the reaction was quenched with NH_4_Cl_(sat)_ and extracted with 2 × 15 cm^3^ DCM. The organic layer was dried over MgSO_4_ and the solvent was evaporated under vacuum. The product was then isolated as a mixture of *anti* and *syn* diastereomers by column chromatography (12 g of silica, EtOAc:petrolether = 1:3 or 1:2, see Supplementary Materials).

### Computational Details

All structures were optimized using the M06-2X/6-31G(d,p) DFT approach in cyclohexanone modeled as the dielectric continuum (SMD, ε_0_(cyclohexanone) = 15.619) [52,53]. The stationary points were verified by vibrational analysis showing no imaginary frequencies for minima or 1 for the transition state structures. Electronic energies were further refined by the single point calculations using the (SMD)/M06-2X/6-311++G(3df,2pd) (in cyclohexanone) approach. All calculations were performed using the Gaussian16 program package [54] and Molden 5.9 was used for visualization [55,56].

## 4. Conclusions

The results obtained in this work show the following trend of the cocatalytic activity: furfurylguanidinium salts (Group 1) ≈ polycyclic guanidinium salts (Group 3) > flexible oxanorbornane-substituted guanidinium salts (Group 2). Among the tested cocatalysts, the salts **4^pf^** and **20** showed similar and overall the best results. The highest enantioselectivities for cocatalyst **20** were obtained with 2–nitrobenzaldehyde (**B4**, *e.e.* > 99.5% at 0 °C) and 4–chlorobenzaldehyde (**B1**, *e.e.* = 99% at 0 °C). The comparable results for 4–chlorobenzaldehyde were also obtained with cocatalyst **4^pf^**. Somewhat surprising was the only moderate performance of cocatalyst **19** despite its well-defined directionality of NH bonds. The presence of the strong hydrogen bond-accepting groups in the structure significantly reduced the activity of the cocatalysts. Also, the presence of the hydroxy group in the substrate deteriorates the diastereo- and enantioselectivity to a similar extent as methanol presumably due to competitive hydrogen bonding. The obtained results are particularly promising since they were obtained with hexafluorophosphate salts which are known to perform slightly poorer than the tetrafluoroborates [26].

An increase in enantioselectivity going from 4-chloro to 2-nitrobenzaldehyde was rationalized by modeling the C-C bond formation step. Calculations predict that the paths “a” and “b” leading to the “*anti*” pre-organized intermediates have either the lowest (path “a”, 2*S*,1′*R*) or the highest (path “b”, 2*R*,1′*S*) barriers. The increase in enantioselectivity is attributed to the increased difference in the barriers that go from 30 kJ mol^−1^ (aldehyde **B1**) to 34.3 kJ mol^−1^ (aldehyde **B4**). While these differences in barriers presume a stereospecific reaction, the reversibility and supramolecular nature of the catalytic complex implies a certain amount of the free enamine present in the mixture, which could be responsible for the formation of the less abundant *anti*-enantiomer [26].

The results presented here associated with the relatively simple synthesis of such polycyclic structures [35] indicate that polycyclic derivative **20** as well as the structurally much simpler, but less stable salt **4^pf^** could be very good lead structures for the development of new cocatalysts.

## Figures and Tables

**Table 1:** Cocatalytic activity of investigated guanidinium salts at room temperature ^a^.

Entry	cocat	*anti*/*syn* ^b^	Conv. (%) ^b^	Yield (%) ^c^
1	-	67:33	73	53
2	**1**	84:16	97	66
3	**2**	83:17	88	61
4	**3**	85:15	97	66
5	**4**	89:11	98	72
6	**4^pf^**	92:8	98	83
7	**5**	89:11	98	75
8	**6**	61:39	98	75
9	**7**	77:23	98	72
10	**8**	69:31	78	54
11	**9**	70:30	73	55
12	**10**	79:21	93	75
13	**11**	83:17	97	82
14	**12**	75:25	90	73
15	**13**	79:21	92	84
16	**14**	88:12	90	90
17	**15**	69:31	87	58
18	**16**	73:27	84	75
19	**17**	75:25	90	75
20	**18**	76:24	94	80
21	**19**	84:16	97	62
22	**20**	92:8	97	88

^a^ r.t. = 18–20 °C. Reaction conditions and the reaction component ratios are shown in Scheme 1. All data were obtained on a one-reaction basis. ^b^ Determined by ^1^H NMR from the crude reaction mixture. ^c^ Isolated yield.

**Table 2:** Performance of cocatalyst **20** in the aldol reaction with different benzaldehydes ^a^.

Entry	benzaldehyde	Conditions ^b^	*anti/syn* ^c^	Conv. (%) ^c^	Yield (%) ^d^	*e.e.* /% ^e^
1	**B1**	10 °C	94:6	97	93	95 (80)
2	**B1**	0 °C	96:4	86	70	99
3	**B1**	MeOH	79:21	93	80	33
4	**B1 ^f^**		44:56	99	48	rac.
5	**B1 ^g^**	0 °C	95:5	80	63	99.5
6	**B2**		85:15	98	95	95
7	**B3**		83:17	66	53	84
8	**B4**		94:6	99	93	98
9	**B4**	0 °C	98:2	99	97	>99.5
10	**B5**		87:13	99	97	89
11	**B6**		86:14	99	96	92
12	**B6**	0 °C	94:6	99	97	95
13	**B7**		79:21	89	65	55

^a^ r.t. = 18–20 °C. Reaction conditions and the reaction component ratios are shown in Figure 4. All data were obtained on a one-reaction basis. ^b^ Reaction parameter that deviates from the standard conditions. ^c^ Determined by ^1^H NMR from the crude reaction mixture. ^d^ Isolated yield. ^e^ *e*.*e*. was determined for *anti* diastereomer. Value in parenthesis refers to a reaction conducted at room temperature (see Table 1). In all cases, the (*S*)-2-((*R*)-hydroxyarylmethyl)cyclohexan-1-one configuration was ascribed [30]. ^f^ Neutral guanidine **20n** was used as the catalyst (without (*S*)-proline). Neutral guanidine was obtained by deprotonation of the salt with an equimolar amount of KOH (see Supplementary Materials). ^g^ Cocatalyst **4^pf^** was used instead of **20**.

## Data Availability

The data are available in this publication and Supplementary Materials.

## Supplementary Materials

The following supporting information can be downloaded at https://www.mdpi.com/article/10.3390/ijms25105570/s1. Reference [57] is cited in Supplementary Materials.
