# Supplementary material for: Cocatalytic Activity of the Furfuryl and Oxanorbornane-Substituted Guanidines in the Aldol Reaction Catalyzed by (S)-Proline
Source: Int J Mol Sci. 2024 May 20;25(10):5570. doi: 10.3390/ijms25105570 (PMC11121891; doi:10.3390/ijms25105570)
Supplement: Supplementary file 1 [file ijms-25-05570-s001.zip › ijms-3008503-supplementary.pdf]

Supporting Materials

for

Cocatalytic Activity of the Furfuryl and Oxanorbornane-substituted Guanidines in the Aldol reaction Catalyzed by (*S*)-Proline

Luka Barešić, Monika Marijanović, Irena Dokli, Davor Margetić and Zoran Glasovac

Table of Contents:

S1. Complexation energies for the selected guanidinium cations with (*S*)-proline 2

S1.1. The role of the intramolecular hydrogen bonding 3

S1.2. Replacement of the furan with oxanorbornane subunit... 4

S2. The Influence of organic solvents on the cocatalytic activity of the selected guanidinium salts. 11

S3. Synthesis of novel oxanorbornane-substituted guanidines 12

S3.1. General remarks. 12

S3.2. Experimental procedures 12

S3.2.1. Synthesis of cocatalyst **7**.. 12

S3.2.3. Synthesis of cocatalysts **10** and **11**. 18

S3.2.4. Synthesis of cocatalyst **15**.. 21

S3.3. NMR spectra 23

S4. Diastereo- and enantioselectivities as determined by ^{1}H NMR and Chiral HPLC................................ 37

S5. Cartesian coordinates 57

S6. References 147

S1. Complexation energies for the selected guanidinium cations with (*S*)-proline

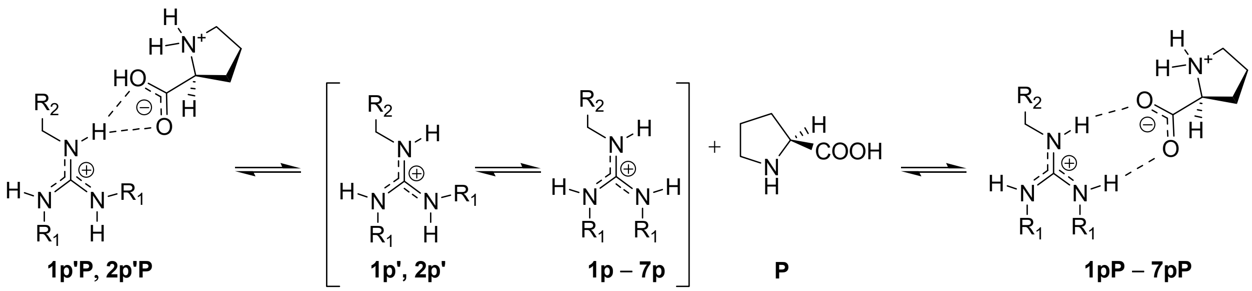

1p: R_1 = Me, R_2 = 2-furyl

2p: R_1 = *i*-Pr, R_2 = 2-furyl

4p: R_1 = -$(CH_2)_2$ -, R_2 = 2-furyl

5p: R_1 = -$(CH_2)_3$ -, R_2 = 2-furyl

6p: R_1 = -$(CH_2)_2$ -, R_2 = -$(CH_2)_2N(CH_3)_2$

7p: R_1 = -$(CH_2)_2$ -, R_2 = -$(CH_2)_2C(O)N(CH_2)_4$

11p: R_1 = -$(CH_2)_2$ -, R_2 =

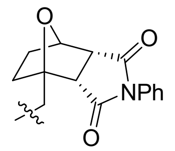

12p: R_1 = *i*-Pr, R_2 =

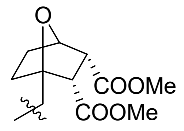

Figure S1. Structures of the modeled guanidinium cations and their complexes with (*S*)-proline.

Table S1. Gibbs energies for the formation of the hydrogen-bonded complexes between the selected guanidine salts and (*S*)-proline.

	E_{scf} / a.u.	G_{corr} / a.u.	G / a.u.	ΔG_{HB}[a] / kJ mol^{-1}
**(*S*)-Proline**	−401.15871	0.11436	−401.04436	
**1pP**	−953.75941	0.32593	−953.43348	9.4
**(1p)**	(−552.58129)	(0.18857)	(−552.39271)	
**[1p']** [a]	[−552.57585]	[0.18771]	[−552.38815]	[12.7] [c]
**2pP**	−1110.99576	0.43142	−1110.56434	7.7
**(2p)**	(−709.81879)	(0.29587)	(−709.52293)	
**[2p']** [a]	[−709.81275]	[0.29282]	[−709.51993]	[13.2] [c]
**4pP**	−952.56279	0.30458	−952.25821	-1.7
**(4p)**	(−551.38166)	(0.16846)	(−551.21321)	
**5pP**	−991.87838	0.33227	−991.54612	-1.1
**(5p)**	(−590.69716)	(0.19581)	(−590.50135)	
**6pP**	−936.30779	0.38242	−935.92536	-0.9
**(6p)**	(−535.12889)	(0.24822)	(−534.88067)	
**7pP**	−1127.09233	0.42592	−1126.66641	1.9
**(7p)**	(−725.91351)	(0.29072)	(−725.62279)	
**11pP**	-1544.29523	0.47284	-1543.82239	-4.7
**(11p)**	(-1143.11432)	(0.33810)	(-1142.77622)	
**12pP**	-1646.59035	0.58978	-1646.00058	14.5
**(12p)**	(-1245.41586)	(0.45412)	(-1244.96174)	
**TBD**	-840.46578	0.32109	-840.14469	-1.1

[a] Calculated against the most stable isomer of **1p–7p.**

[b] The values in square brackets were calculated for the most stable ("propeller-like") conformation of guanidines **1p** and **2p**.
[c] ΔG_{HB} for the formation of **1p'P** and **2p'P** complexes (Scheme S1).
[d] 1,5,7-triazabicyclo[4.4.0]dec-1-ene (**TBD**).

In most cases, the formation of the complex with proline is practically thermoneutral or weakly exergonic. The exceptions are derivatives **1p** and **2p** where the endergonicity of the complexation is mainly due to the conformational change in the guanidine subunit necessary for the optimal proline binding with two hydrogen bonds.

S1.1. The role of the intramolecular hydrogen bonding

The guanidinium salts **6** and **7** were used to test the role of the intramolecular hydrogen bonding (IMHB).

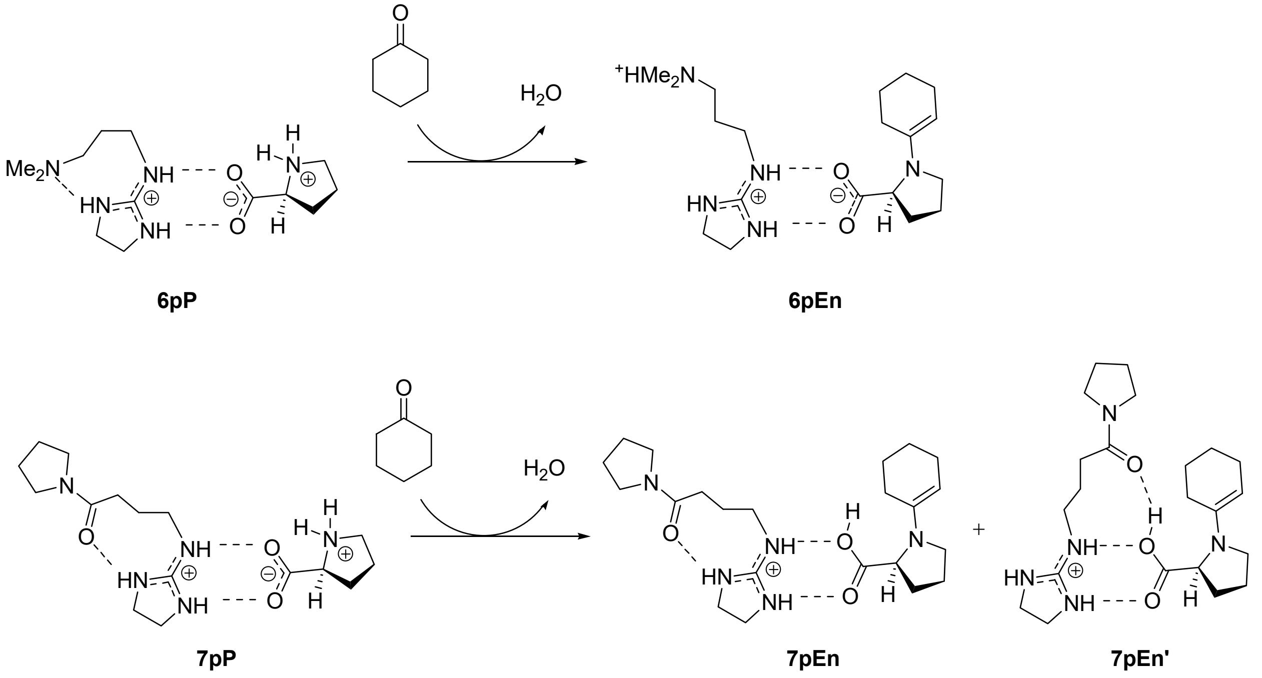

Scheme S1. Schematic structures of proline (**6pP** and **7pP**) and enamine (**6pEn** and **7pEn**) complexes with guanidinium cations capable of strong intramolecular hydrogen bonding.

Table S2. Gibbs energies for the formation of the hydrogen-bonded complexes between the guanidine salts **6** or **7** and (*S*)-1-cyclohexenylpyrrolidine-2-carboxylic acid (enamine, **En**).

	E_{scf} / a.u.	G_{corr} / a.u.	G / a.u.	ΔG_{HB}[a] / kJ mol^{-1}
**(*S*)-Proline**	−401.15871	0.11436	−401.04436	
**Cyclohexanone**	-309.86716	0.12189	-309.74528	
**water**	-76.43344	0.00380	-76.42965	
**6pEn**	-1169.72230	0.49762	-1169.22467	42.0
**7pEn**	-1360.50611	0.54396<br>(0.57403)[b]	-1359.96215<br>(-1359.93208)[b]	54.2<br>(0.0)[b,c]
**7pEn'**	-1360.51161	0.55021<br>(0.57228)[b]	-1359.96141<br>(-1359.93933)[b]	56.1<br>(-19.0)[b,c]

[a] Calculated against isolated reactants
[b] the data corrected for the low-energy vibration contributions to the entropy are given in parentheses
[c] relative stabilities of two isomeric complexes of the enamine **7pEn**. The Gibbs energies are entropy corrected for the low energy vibrations.

Calculated data show different origins of the decrease in the selectivity for cocatalysts **6** and **7**. At the enamine stage, cocatalyst **6** most likely deprotonates the enamine carboxylic group preventing the substrate binding and activation. The binding of enamine by the cocatalyst **7** is a more endergonic process than that of **6**, **11** (see next subsection), or **20** (49.4 kJ mol^{-1}). Two isomeric structures close in energy were identified one of which has an intramolecular hydrogen bond between amide functionality and proline proton. The Gibbs energies uncorrected for the contribution of low-energy vibrations to the entropy imply the structure **7pEn** to be slightly more stable ($\Delta(\Delta G_{HB})$= 1.9 kJ mol^{-1}. After correction, the trend is reversed and **7pEn'** becomes more stable by 19 kJ mol^{-1}. This result implies intramolecular hydrogen bonding as the main reason for the lowered diastereoselectivity of **7**. From the results described, we can conclude that in both cases (usage of the cocatalysts **6** and **7**) concurrent formation of the product by an uncoordinated (*S*)-proline is expected largely. Therefore, lower diastereoselectivities than in the case of **4** or **6**, are not surprising.

S1.2. Replacement of the furan with oxanorbornane subunit

The negative impact of the replacement of the furan subunit by oxanorbornane cage is evident in a decrease of stereoselectivity going from **2** to **12** and **4** to **11**. Calculations indicate different origins of this result (Scheme S2 and Table S2).

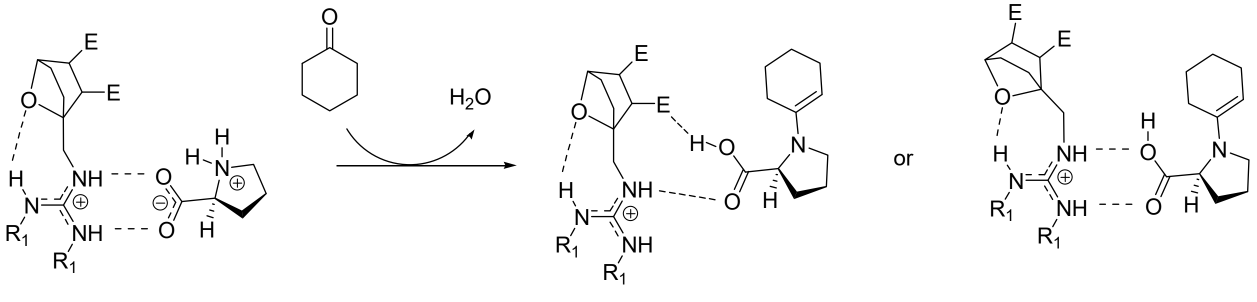

Scheme S2. Schematic representation of two lowest energy cocatalyst/enamine complexes starting from the guanidinium salts **11** or **12**.

Table S3. Gibbs energies for the formation of the hydrogen-bonded complexes between the selected guanidine salts and (*S*)-Proline and its cyclohexane enamine .[a]

	E_{scf} / a.u.	G_{corr} / a.u.	G / a.u.	ΔG_{HB}[a,b] / kJ mol^{-1}
**11pP**	-1544.29523	0.47284	-1543.82239	-4.7
**12pP**	-1646.59035	0.58978	-1646.00058	14.5
**enamine struct.**[c]				
**11pEn**	-1777.71501	0.59749	-1777.11752	49.1
**11pEn'**	-1777.70609	0.59184	-1777.11425	57.7
**12pEn**	-1880.00745	0.71393	-1879.29352	74.0

**12pEn'**	-1880.00401	0.70924	-1879.29476	70.8

[a] For the identification of the structures see Scheme S2. The optimized structures of **11pEn** and **12pEn'** are also shown in Figure S3.

[b] calculated against separate reactants.

[c] The relative Gibbs energies of **11pEn** and **12pEn** were calculated against **11pEn'** and **12pEn'**, respectively.

Cocatalyst **11** forms a more stable complex with (*S*)-proline than its furan analog **4**. However, at the enamine stage, the imide subunit tends to form an intramolecular hydrogen bond with the active carboxylic proton and obscures it for the substrate approach (Fig S2).

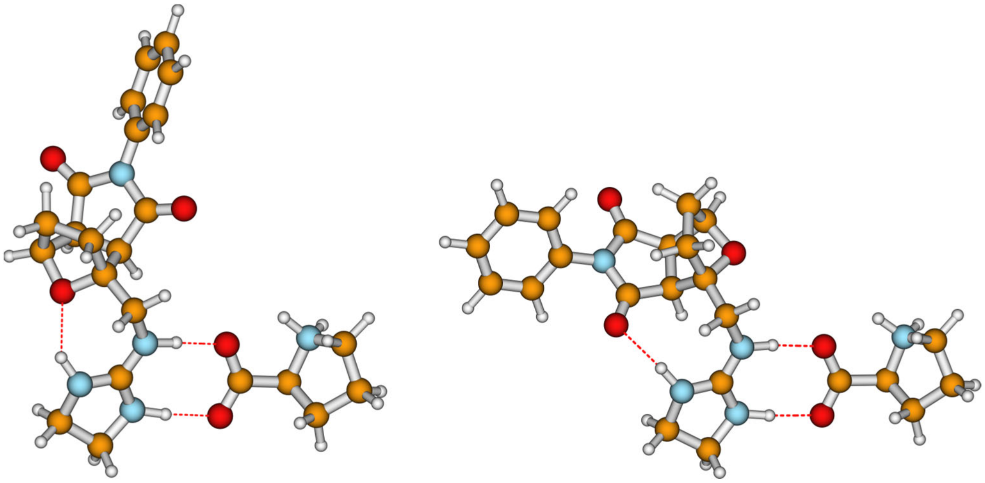

11pP (conf. 1, Table S2) **11pP** (conf. 2)

ΔG_{rel} /kJ mol^{-1} = **0.0** **5.2**

Figure S2. Structures and relative Gibbs energies of two (*S*)-proline complexes of **11p**.

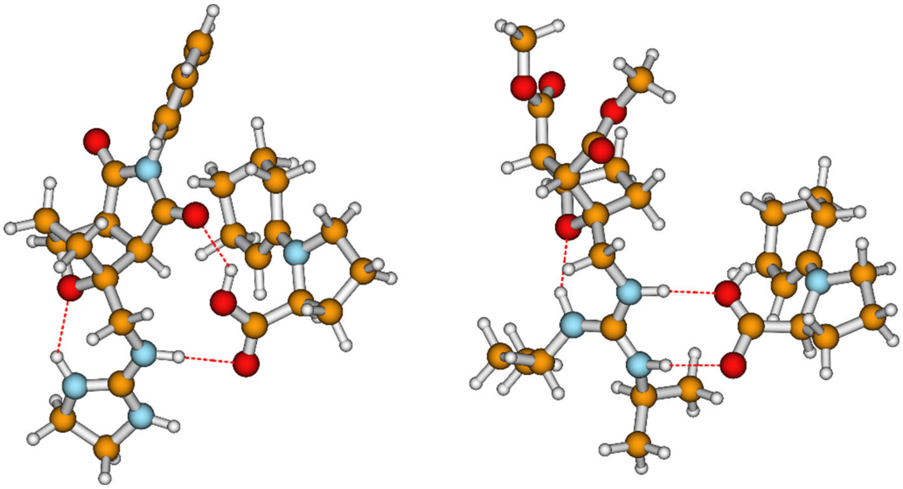

11pEn (see Table S2) **12pEn'**

ΔG_{rel} /kJ mol^{-1} = **49.1** **70.8**

Figure S3. Optimized structures of **11pEn** (left) and **12pEn'** (right) as the most stable conformers of the cocatalyst/enamine complexes.

In the case of diisopropyl derivative **12**, the formation of the proline complex **12pP** is more endergonic than for **2pP** (14.5 and 7.7 kJ mol^{-1}, respectively). Ester groups do not form sufficiently strong hydrogen bonds and the structure **12pEn'** (Scheme S2) is more stable than the **12pEn**. The approach of the substrate is mostly affected by the sterical influence of the oxanorbornane and diisopropyl groups. Due to the supramolecular nature of the cocatalyst/proline complex, unbound forms are expected to be more abundant and a significant amount of the product is most likely formed without the influence of the cocatalyst besides the increase in solubility of (*S*)-proline. The lower stability of the complex with the proline and more endergonic reaction imply a slower reaction and it is most likely the reason for the lower conversion.

S1.3. Energy profiles for the C-C-bond formation steps

a) Mechanism of the reaction

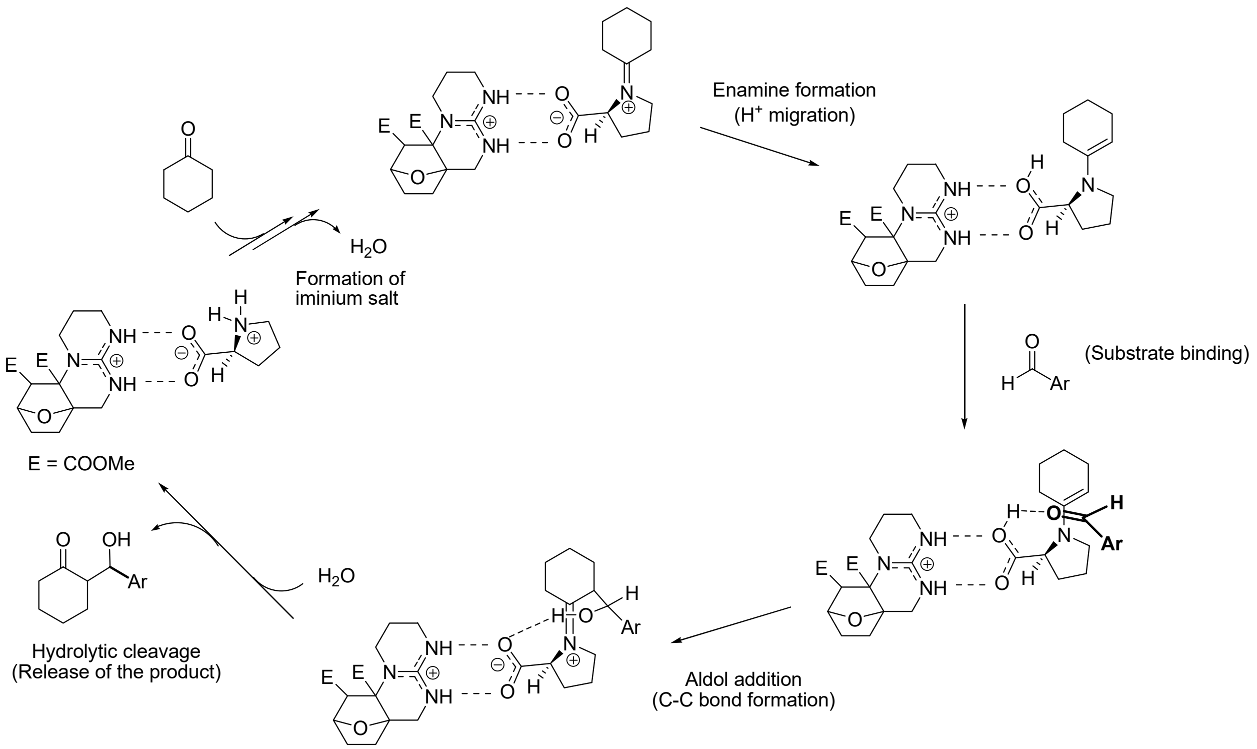

Scheme S3. Schematic representation of the catalytic cycle[1].

b) 4-Chlorobenzaldehyde as the substrate:

Table S4. Gibbs energies of the structures along the C-C bond formation path[a,b].

	E_{scf} / a.u.	G_{corr} / a.u.	G / a.u.	ΔG_{HB}[a] / kJ mol^{-1}
**20p**	-1123.84335	0.31218	-1123.53117	
**B1**	-805.15913	0.06870	-805.09043	
**Ia = Ic**	-1758.43657	0.56494	-1757.87163	51.3
**Ib = Id**	-1758.43708	0.56429	-1757.87279	48.2
**Path "a"**				
**IIa**	-2563.61189	0.65469	-2562.95721	64.0
**TS1a**	-2563.60231	0.65556	-2562.94675	91.5
**IIIa**	-2563.63900	0.66255	-2562.97644	13.5
**Path "b"**				
**IIb**	-2563.61323	0.66244	-2562.95079	80.8
**TS1b**	-2563.59411	0.65937	-2562.93474	123.0
**IIIb**	-2563.64071	0.66333	-2562.97738	11.0
**Path "c"**				
**IIc**	-2563.61358	0.66060	-2562.95298	75.1
**TS1c**	-2563.59627	0.65518	-2562.94109	106.3
**IIIc**	-2563.63325	0.66362	-2562.96963	31.4
**Path "d"**				
**IId**	-2563.61071	0.66351	-2562.94720	90.3
**TS1d**	-2563.59796	0.66147	-2562.93649	118.4
**IIId**	-2563.63120	0.66631	-2562.96489	43.8

[a] Calculated against the sum of energies of the isolated reactants (**20p, B1**, and cyclohexanone) and corrected for the loss of water. Gibbs energies of (*S*)–proline, cyclohexanone and water are given in Table S2.

[b] Calculated using SMD/M06-2X/6-311++(3df,2pd)//SMD/M06-2X/6-31G(d,p) assuming cyclohexanone as a solvent.

c) 2-Nitrobenzaldehyde as the substrate

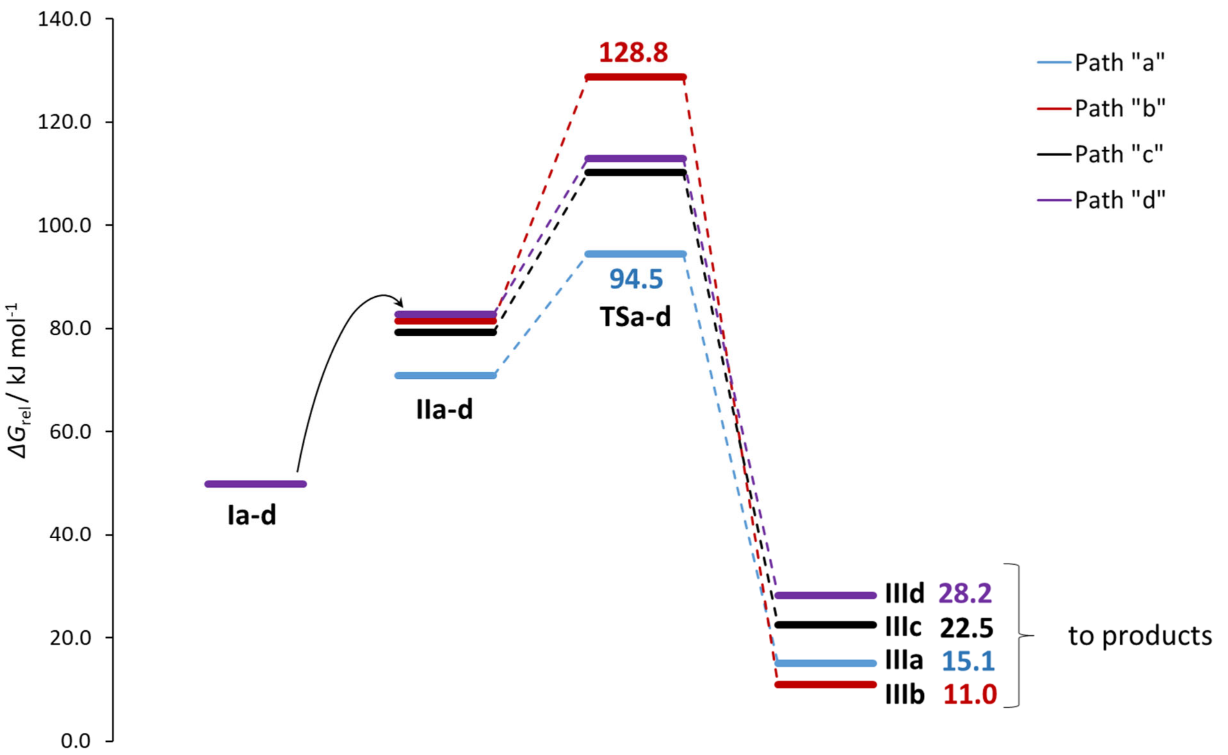

Figure S4. The relative Gibbs energies of several stationary points along the paths for the formation of 4 possible enantiomers starting from 2-NO_2-benzaldehyde. The relative Gibbs energies (in kJ mol^{-1}) were calculated against the separate components: (*S*)-proline, guanidinium cation **20p**, cyclohexanone, 2-nitrobenzaldehyde (**B4**) and water.

Table S5. Gibbs energies of the structures along the C-C bond formation path[a,b].

	E_{scf} / a.u.	G_{corr} / a.u.	G / a.u.	ΔG_{HB}[a] / kJ mol^{-1}
**20p**	(see Table S4)			
**B4**	-550.05209	0.07971	-549.97237	
**Ia = Ic**	(see Table S4)			
**Ib = Id**	(see Table S4)			
**Path "a"**				
**IIa**	-2309.74456	0.69366	-2309.05090	70.9
**TS1a**	-2309.73641	0.69450	-2309.04191	94.5
**IIIa**	-2309.77365	0.70150	-2309.07216	15.1
**Path "b"**				
**IIb**	-2309.74188	0.69497	-2309.04691	81.4
**TS1b**	-2309.72679	0.69792	-2309.02887	128.8
**IIIb**	-2309.77496	0.70125	-2309.07371	11.0
**Path "c"**				
**IIc**	-2309.74392	0.69619	-2309.04773	79.2
**TS1c**	-2309.72900	0.69310	-2309.03590	110.3
**IIIc**	-2309.76951	0.70019	-2309.06933	22.5
**Path "d"**				

**IId**	-2309.74662	0.70019	-2309.04642	82.7
**TS1d**	-2309.73470	0.69979	-2309.03491	112.9
**IIId**	-2309.76734	0.70016	-2309.06718	28.2

[a] Calculated against the sum of energies of the isolated reactants (**20p, B4**, and cyclohexanone) and corrected for the loss of water. Gibbs energies of (*S*)–proline, cyclohexanone and water are given in Table S2 while the **20p** data are given in Table S4.

[b] Calculated using SMD/M06-2X/6-311++(3df,2pd)//SMD/M06-2X/6-31G(d,p) assuming cyclohexanone as a solvent.

d) $TBDH^+$ as the cocatalyst

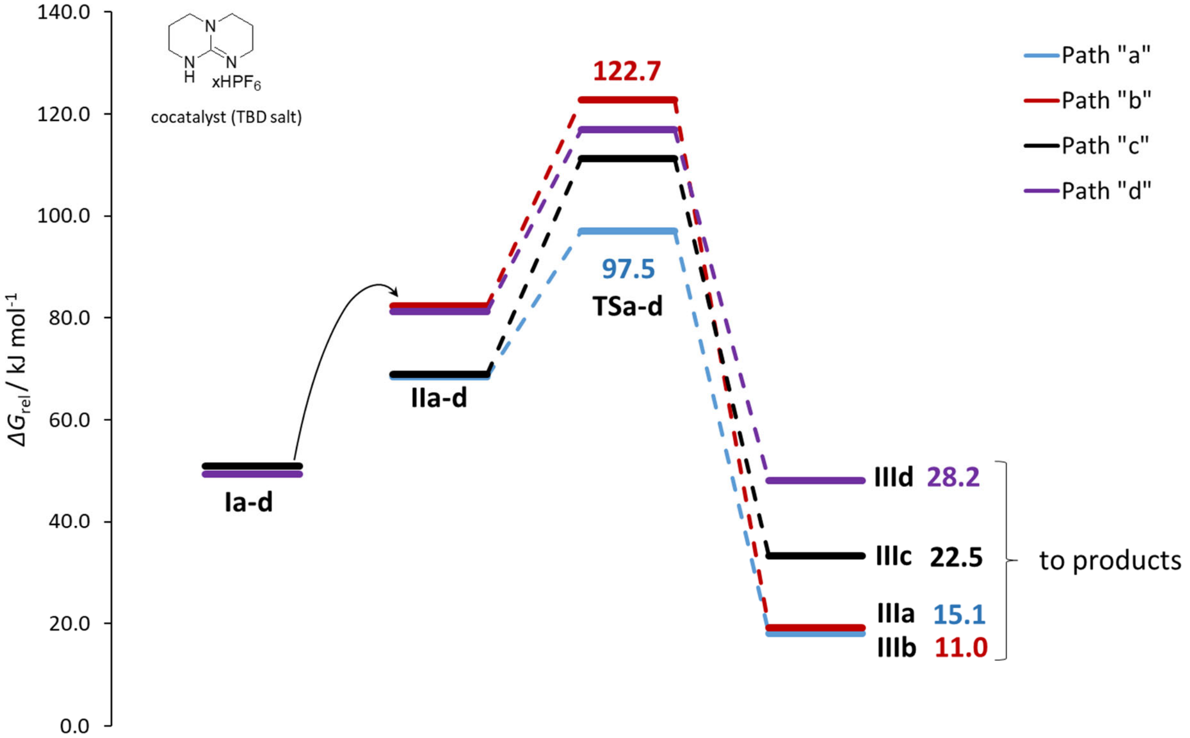

Figure S5. The relative Gibbs energies of several stationary points along the paths for the formation of 4 possible enantiomers using the protonated **TBD** hexafluorophosphate as the cocatalyst . The relative Gibbs energies (in kJ mol^{-1}) were calculated against the separate components: (*S*)-proline, guanidinium cation **TBDp**, cyclohexanone, 4-chlorobenzaldehyde (**B1**) and water.

Table S6. Gibbs energies of the structures along the C-C bond formation path with $TBDH^+$ as the cocatalytic component[a,b].

	E_{scf} / a.u.	G_{corr} / a.u.	G / a.u.	ΔG_{HB}[a] / kJ mol^{-1}
**$TBDH^+$**	-439.28702	0.18711	-439.09991	
**B1**	(see Table S4)			
**Ia = Ic**	-1073.88016	0.43966	-1073.44049	50.9
**Ib = Id**	-1073.88039	0.43930	-1073.44109	49.4
**Path "a"**				
**IIa**	-1879.05498	0.53072	-1878.52425	68.4
**TS1a**	-1879.04504	0.53168	-1878.51336	97.0
**IIIa**	-1879.08115	0.53773	-1878.54342	18.1
**Path "b"**				
**IIb**	-1879.05335	0.53437	-1878.51898	82.3
**TS1b**	-1879.03745	0.53385	-1878.50360	122.7
**IIIb**	-1879.08327	0.54025	-1878.54302	19.2
**Path "c"**				
**IIc**	-1879.05439	0.53032	-1878.52407	68.9
**TS1c**	-1879.03894	0.53098	-1878.50796	111.2
**IIIc**	-1879.07541	0.53776	-1878.53764	33.3
**Path "d"**				
**IId**	-1879.05332	0.53397	-1878.51935	81.3
**TS1d**	-1879.04056	0.53476	-1878.50580	116.9
**IIId**	-1879.07394	0.54192	-1878.53202	48.1

[a] Calculated against the sum of energies of the isolated reactants (**$TBDH^+$, B1**, and cyclohexanone) and corrected for the loss of water. Gibbs energies of (*S*)–proline, cyclohexanone and water are given in Table S2.

[b] Calculated using SMD/M06-2X/6-311++(3df,2pd)//SMD/M06-2X/6-31G(d,p) assuming cyclohexanone as a solvent.

S2. The Influence of organic solvents on the cocatalytic activity of the selected guanidinium salts.

The influence of solvent was tested for the poorly soluble cocatalysts **8** and **9**. In these reactions, 2 mmol of cyclohexanone was replaced with an equal volume of the corresponding solvent (217 μL). A reduced amount of the cyclohexanone relative to the 4-chlorobenzaldehyde (**B1**) (5-fold excess) was considered to have minimal impact on the reaction rate and the duration of the reaction was kept unchanged. The solubility of the cocatalysts was greatly improved in the employed solvents. In the case of methanol, the results with cocatalysts **10** and **11** were added for comparison. The results are presented in Table S7.

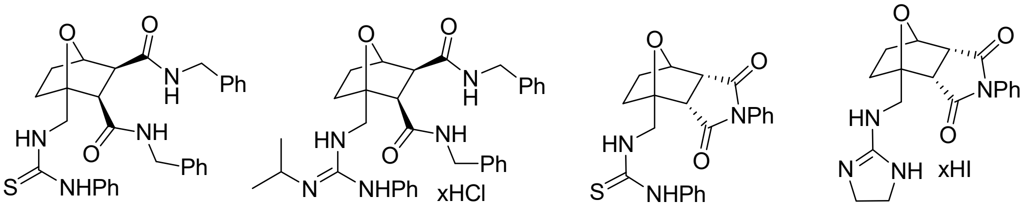

8 **9** **10** **11**

Table S7. Influence of solvent on the cocatalytic activity of the selected guanidinium salts[a]

Entry	cocat	solvent[a]	***anti : syn***[b]	Conv. (%)[b]	Yield (%)[c]
1	-	MeOH	78 : 22	95	90
2	**8**	MeOH	79 : 21	99	93
3	**8**	DCM	70 : 30	59	20
4	**9**	MeOH	80 : 20	97	88
5	**9**	DCM	73 : 27	72	28
6	**9**	ACN	70 : 30	63	38
7	**9**	DMF	58 : 42	96	79
8	**9**	MeOH (-20)	95 : 5	84	80
9	**9**	MeOH (+50)	46 : 54	96	40
10	**10**	MeOH	78 : 22	94	83
11	**11**	MeOH	74 : 26	94	85

[a] r.t. = 18-20 °C unless otherwise stated (in parentheses). Reaction conditions and the reaction component ratios are shown in Scheme 1.

[b] Determined by ^{1}H NMR.

[c] Isolated yield.

As expected, methanol strongly improved selectivity, conversion, and isolated yields. The presence of cocatalyst did not influence the reaction in methanol significantly. This is reflected in small variations of the selectivity regardless of whether the change in solubility of the cocatalyst occurred or not. DCM and ACN had minimal effect while DMF deteriorated selectivity and improved the yield of the reaction compared to the results in cyclohexanone.

S3. Synthesis of novel oxanorbornane-substituted guanidines

S3.1. General remarks.

All commercially available chemicals and solvents were used without prior purification. Furfurylamine and imidazolidine-2-thione were purchased from Alfa Æsar, and solvents were purchased from WWR (THF), KEFO d.o.o. (EtOH, MeOH, and EtOAc), Gram-Mol (Petroleum ether, b.p. 65-90 °C and CH_2Cl_2). or Riedel-de-Haën (CH_3CN). TLC plates (TLC Silicagel 60, 63-200 µm, 254 nm) were purchased from Merck KGaA. Microwave-assisted reactions were conducted on CEM Discover single-mode instrument in a closed vessel equipped with a magnetic stirrer. The temperature was controlled by the external IR sensor located below the vessel. NMR spectra were recorded on Bruker Avance AV600 and AV300 using TMS as the reference compound. X-ray structure determination was conducted on Rigaku XtaLAB Synergy S diffractometer. High-resolution mass spectra (HRMS) were recorded on an Agilent 6550 Series Accurate-Mass-Quadrupole Time-of-Flight (Q-TOF). Infrared spectra (FTIR-ATR) were recorded on the Fourier Transform Infrared Attenuated Total Reflection PerkinElmer UATR Two Spectrometer (range 400-4000 cm^{-1}). Chiral HPLC analyses were done on Agilent 1260 Series II instrument equipped with a chiral column.

N-Boc-furfurylamine (**21**), furfurylguanidinium iodides **1–6**, as well as hexafluorophosphate salts **4^{pf}**, **12, 13** and **16–20** were prepared as described earlier[1–4]. Salt **7** was prepared via the guanidinylation of 4-oxo-4-(pyrrolidin-1-yl)butanammonium hydroxide with imidazolidin-2-ylidene(methyl)sulfonium iodide (**MIM**, Scheme 3C, the main text). The oxanorbornane-substituted thioureas (**8** and **10**) and guanidinium salts **9** and **11** were prepared according to the reactions shown in Scheme 3 in the main text.

S3.2. Experimental procedures

<u>S3.2.1. Synthesis of cocatalyst **7**</u>

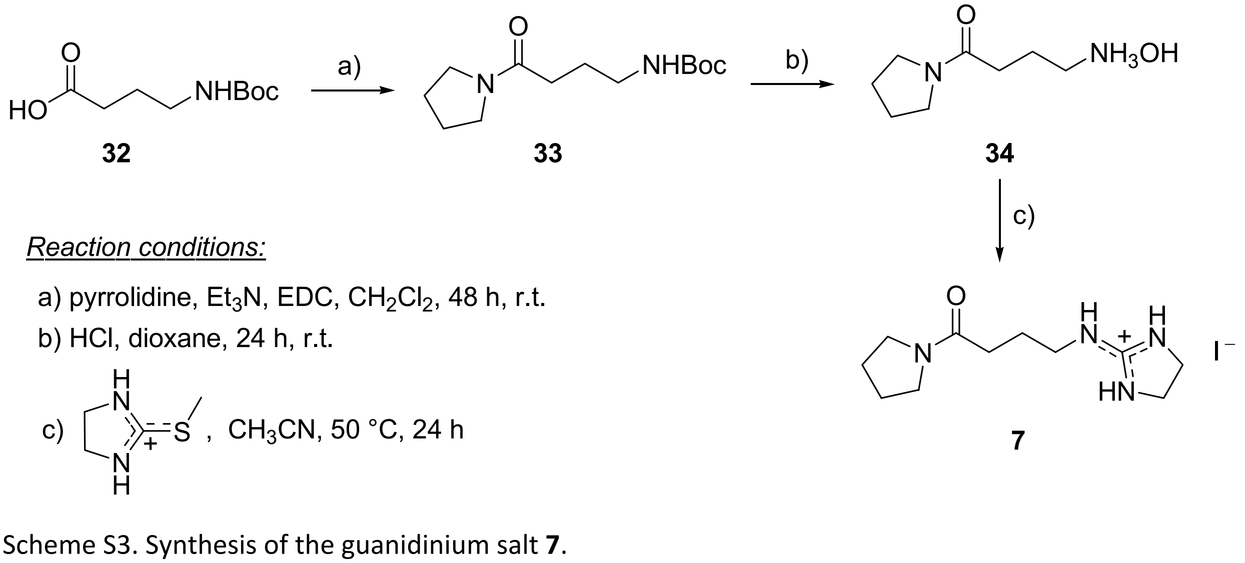

Scheme S3. Synthesis of the guanidinium salt **7**.

Tert-butyl 4-oxo-4-(pyrrolidin-1-yl)butylcarbamate (33):

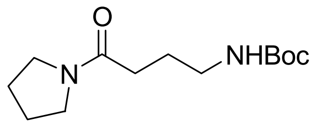

In a round-bottomed flask filled with argon, **32**[5] (5.584 g, 27.50 mmol) was dissolved in 50 cm^3 of dry CH_2Cl_2. To this solution, pyrrolidine (1.630 g, 1.91 cm^3, 22.92 mmol) was added, and after brief stirring, triethylamine (2.319 g, 3.19 cm^3, 22.92 mmol) and 1-(3-dimethylaminopropyl)-3-ethylcarbodiimide hydrochloride (EDC) (5.272 g, 27.50 mmol) were added in that order at the room temperature. The stirring was continued for the next 48 h. The resulting mixture was transferred to the separation funnel, and washed with 1 N HCl (3 x 10 cm^3), 5% aqueous $NaHCO_3$ (3 x 10 cm^3), and brine (3 x 10 cm^3). The organic layer was dried ($MgSO_4$), filtered and volatiles were evaporated providing the white solid as the product **33** (5,547 g, yield 94%).

^1H NMR (DMSO-d_6, 300 MHz) δ/ppm: 6.80 (t, 1H, $^3J_{H-H}$=4.5 Hz, NH), 3.36 (t, 2H, $^3J_{H-H}$ = 6,8), 3.26 (t, 2H, $^3J_{H-H}$ = 6.7), 2.89-2.95 (m, 2H), 2.20 (t, 2H, $^3J_{H-H}$ = 7.2 Hz), 1.81-1.90 (m, 2H), 1.70-1.79 (m, 2H), 1.59 (quintet, 2H, $^3J_{H-H}$=7.2 Hz), 1.37 (s, 9H).

^{13}C NMR ($CDCl_3$, 150.9 MHz) δ/ppm: 171.2, 156.3, 79.1, 46.7, 45.8, 40.6, 32.2, 28.6, 26.2, 25.0, 24.5.

HRMS- ESI-Q-TOF: Found: $[M]^+$: 257.1863, calculated for $[C_{15}H_{18}N_2O_3]^+$, $[M+H]^+$: 257.1860

4-(Hydroxyamino)-1-(pyrrolidin-1-yl)butan-1-one (34):

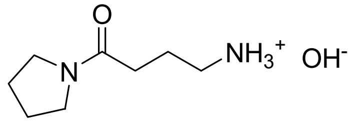

Amide **33** (220 mg, 0.858 mmol) was dissolved in dioxane (4 cm^3) and conc- HCl (1 cm^3) was added dropwise. The stirring was continued for the next 24 h at room temperature. After that time, pH was adjusted to 9 by adding the aqueous Na_2CO_3. Volatiles were removed in vacuo, and acetonitrile (5 cm^3) was added to precipitate the product. The precipitate was filtered with suction, and washed with an additional amount of acetonitrile (5 cm^3). The product **34** was obtained in a hydrated form (113 mg, yield 84%).

^{1}H NMR (DMSO-d_6, 300 MHz): δ/ppm: 8.07 (s, 3H, NH_3^+), 3.50 (br s, 1H, OH), 3.35-3.40 (t, 2H, $^3J_{H-H}$=6.8 Hz), 3.25-3.30 (t, 2H, $^3J_{H-H}$=6.7 Hz), 2.74-2.85 (m, 2H), 2.35-2.39 (t, 2H, $^3J_{H-H}$=7.1 Hz), 1.72-1.92 (m, 6H).

^{13}C NMR (DMSO-d_6, 75.5 MHz) δ/ppm: 169.5, 45.8, 45.2, 38.4, 30.6, 25.5, 23.9, 22.2.

HRMS- Q-TOF: Found: $[M - OH]^+$: 157.1339, calculated for $[C_8H_{17}N_2O]^+$, $[M - OH]^+$: 157.1335

4-(Imidazolidin-2-ylideneamino)-1-(pyrrolidin-1-yl)butan-1-one hydroiodide (7)

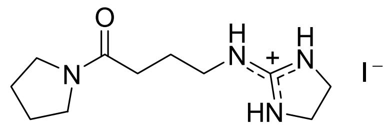

To the solution of **34** (150 mg, 0.861 mmol) in acetonitrile (3 cm^3), 2-methylthio-4,5-dihydro-1*H* imidazole hydroiodide (191 mg, 0.783 mmol), and triethylamine (174 mg, 0.440 cm^3, 1.727 mmol) were added. The reaction mixture was stirred at 50 °C for 24 h. The volatiles were then removed and a methanolic solution of NaOH was added to deprotonate the triethylammonium salts if present in the reaction mixture. The amount of NaOH was determined from the ^{1}H NMR and should be equimolar to the $Et_3N\times HI$. Acetonitrile (5 cm^3) was then added and the precipitate was filtered off. The filtrate was collected and evaporated giving an oily product **7**, which crystallizes upon standing (149 mg, 54 %).

^{1}H NMR (DMSO-d_6, 300 MHz) δ/ppm: 7.07-9.14 (br s, 3H), 3.59 (s, 4H), 3.26-3.40 (m, 7H, *H*$_2$O), 3.13 (t, 2H, $^3J_{H\text{-}H}$=7.0 Hz), 2.29 (t, 2H, $^3J_{H\text{-}H}$=7.0 Hz), 1.84-1.95 (m, 2H), 1.67-1.81 (m, 4H).

^{13}C NMR (DMSO-d_6, 150.9 MHz) δ/ppm: 169.8, 159.4, 45.9, 45.3, 42.5, 41.8, 40.1, 30.4, 25.6, 23.9, 23.8.

FTIR (ATR, $\tilde{\nu}$/cm^{-1}): 3138 (N-H stretch), 1662 (C=N stretch)

HRMS- Q-TOF: Found: $[M - I^-]^+$: 225.1713, calculated for $[C_{11}H_{21}N_4O]^+$, $[M\text{-}Boc+H_2O]^+]^+$: 225.1710.

S3.2.2. Synthesis of cocatalysts **8** and **9**.

***Tert*-butyl ((1,3-dioxo-1,3,3a,4,7,7a-hexahydro-4,7-epoxyisobenzofuran-4-yl)methyl)carbamate (22)**[6]:

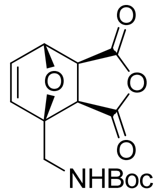

A 10 cm^3 stainless steel jar was charged with *N*-Boc-furfuryl amine (1.170 g, 5.93 mmol), maleic anhydride (1.163 g, 11.86 mmol), and one stainless steel ball (d= 12 mm). The reaction mixture was subjected to a ball milling for 3 h at 30 Hz. After the reaction was completed, the reaction mixture was suspended in EtOAc and filtered over a short plug of Celite. The organic solvent was evaporated under reduced pressure and the remaining dark yellow solid was suspended in DCM (5 cm^3), filtered, and additionally washed with DCM (5 cm^3), and then with petrol ether (100 cm^3) to remove the excess maleic anhydride. The remaining white solid was identified as **22** yielding in 1.148 g, 3.88 mmol, 65%. The product was sufficiently pure for the next step.

^{1}H-NMR (DMSO-d_6, 600 MHz) δ/ppm: 7.01 (t, $^3J_{H\text{-}H}$ = 5.9 Hz, 1H, N*H*), 5.59 (d, $^3J_{H\text{-}H}$ = 4.9 Hz, 1H, *H*C=CH), 6.44 (d, $^3J_{H\text{-}H}$ = 5.5 Hz, 1H, HC=C*H*), 5.28 (d, $^3J_{H\text{-}H}$ = 1.4 Hz, 1H, oxo-bridge-C*H*), 3.80 (dd, $^2J_{H\text{-}H}$ = 14.9 Hz, $^3J_{H\text{-}H}$ = 6.0 Hz, 1H, C*H*$_2$), 3.45 (d, $^3J_{H\text{-}H}$ = 6.9 Hz, 1H, *exo-H*), 3.39 (overlapping, dd, $^2J_{H\text{-}H}$ = 14.9 Hz, $^3J_{H\text{-}H}$ = 6.0 Hz, 1H, C*H*$_2$), 3.37 (overlapping, d, $^3J_{H\text{-}H}$ = 6.6 Hz, 1H, *exo-H*), 1.38 (s, 9H, C(O)O(C*H*$_3$)$_3$).

^{13}C NMR (DMSO-d_6, 150.9 MHz) δ/ppm: 171.8, 170.3, 156.2, 138.3, 138.2, 92.2, 81.8, 78.5, 52.7, 50.7, 39.5, 28.6.

FTIR (ATR $ν_{max}$/cm^{-1}): 3397 (N-H stretch), 1773 (C=O stretch), 1683 (C=O stretch), 1519(C=O stretch).

***Tert*-butyl ((1,3-dioxooctahydro-4,7-epoxyisobenzofuran-4-yl)methyl)carbamate (24):**

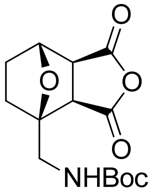

A round bottom flask was charged with **22** (894 mg, 3.02 mmol), Pd/C (10%, 246 mg), and EtOAc (20 cm^3), and reduced under a hydrogen atmosphere (1 atm) for 18 h. The reaction mixture was filtered over a short plug of Celite and additionally washed with EtOAC (3×10 cm^3). After solvent evaporation under reduced pressure remaining white solid was identified as **24** (853 mg, 2.87mmol) in a 95% yield.

^{1}H-NMR ($CDCl_3$, 600 MHz) δ/ppm: 5.05 (br s, 1H, N*H*), 4.97 (d, $^3J_{H-H}$ = 5.2 Hz, 1H, oxa-bridge), 3.79 (dd, $^2J_{H-H}$ = 14.7 Hz, $^3J_{H-H}$ = 6.7 Hz, 1H, C*H*$_2$NH), 3.63 (dd, $^2J_{H-H}$ = 14.7 Hz, $^3J_{H-H}$ = 6.6 Hz, 1H, C*H*$_2$NH), 3.29 (d, $^3J_{H-H}$ = 7.5 Hz, 1H, *endo*-H), 3.23 (d, $^3J_{H-H}$ = 7.5 Hz, 1H, *endo*-H), 2.03-1.98 (m, 1h, C*H*$_2$CH$_2$), 1.93-1.88 (m, 1H, C*H*$_2$CH$_2$), 1.72-1.64 (m, 2H, CH$_2$C*H*$_2$), 1.44 (s, 9H, CO(C*H*$_3$)$_3$).

^{13}C NMR ($CDCl_3$, 150.9 MHz) δ/ppm: 170.9, 170.2, 155.9, 89.1, 80.0, 79.9, 51.8, 51.5, 41.1, 31.5, 29.6, 28.3.

FTIR (ATR $ν_{max}$/cm^{-1}) 3352 (N–H stretch), 1779 (C=O stretch), 1639 (C=O stretch).

HRMS- Q-TOF: Found: [M-Boc+H_2O]$^+$: 216.0875, calculated for [$C_{15}H_{17}N_2O_3$]$^+$, [M-Boc+H_2O]$^+$: 216.0866; Found [M+H_2O+Na]$^+$: 338.1224, calculated for [$C_{14}H_{21}NNaO_7$]$^+$, [M+H_2O+Na]$^+$: 338.1210

Tert-butyl ((2,3-bis(benzylcarbamoyl)-7-oxabicyclo[2.2.1]heptan-1-yl)methyl)carbamate (25):

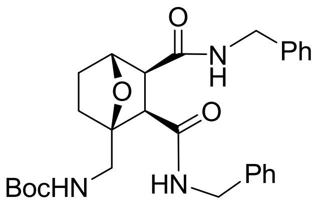

To a stirring solution of **24** (853 mg, 2.87 mmol) in DCM (10 cm^3), benzylamine (615 mg, 5.74 mmol) was added dropwise. After all of the benzylamine was added the reaction mixture was left stirring at ambient temperature for 18 h. DCM was then evaporated under reduced pressure and the remaining yellowish oil was taken up in Et_2O (5 cm^3). The precipitated white solid was filtered off with suction and washed with Et_2O (2×10 cm^3) yielding 1.328 g of the crude product. Obtained material was used in the next step without further purification.

755 mg of the above-mentioned product was added in THF (20 cm^3) at 0°C, after which benzylamine (378 mg, 3.53 mmol), 1-hydroxybenzotriazole (540 mg, 3.53 mmol) and *N*-(3-dimethylaminopropyl)-*N'*-ethylcarbodiimide hydrochloride (845 mg, 4.41 mmol) were added in that order. The reaction mixture was allowed to slowly warm to ambient temperature and was left stirring for 24 hours. The reaction mixture

was then concentrated under reduced pressure. The remaining material was suspended in water (5 cm^3) and vacuum filtered and additionally washed with methanol (2×5 cm^3) and acetone (2×10 cm^3). The product **25** was obtained as white solid (599 mg, 1.21 mmol, 82% yield).

^{1}H-NMR (DMSO-d_6, 600 MHz) δ/ppm: 7.77 (t, $^3J_{H-H}$ = 4.8 Hz, 1H, N*H*), 7.42 (br s, 1H, N*H*), 7.30-7.20 (m, 10H, 2×Ph), 6.37 (br s, 1H, N*H*), 4.78 (d, $^3J_{H-H}$ = 3.4 Hz, 1H, oxa-bridge), 4.23-4.10 (m, 4H, overlapping 2×CH_2NH), 3.42 (d, $^3J_{H-H}$ = 5.9 Hz, CH_2NH), 3.11 (d, $^3J_{H-H}$ = 9.7 Hz, 1H, *endo*-H), 2.97 (d, $^3J_{H-H}$ = 9.6 Hz, 1H, *endo*-H), 1.69-1.45 (m, 4H, CH_2CH_2), 1.39 (s, 9H, CO(CH_3)$_3$).

^{13}C NMR (DMSO-d_6, 150.9 MHz) δ/ppm: 170.5, 169.0, 139.3, 138.9, 128.1, 128.0, 127.6, 127.4, 126.7, 126.6, 87.0, 77.8, 77.1, 56.5, 53.8, 42.6, 42.5, 40.3, 28.2.

FTIR (ATR ν_{max}/cm^{-1}): 3360 (N-H stretch), 1681 (C=O stretch), 1655 (C=O stretch), 1528 (C=O stretch).

HRMS- Q-TOF: Found: $[M+H]^+$: 494.2656, calculated for $[C_{28}H_{36}N_3O_5]^+$, $[M+H]^+$: 494.2655; Complex with Na^+: found: $[M+Na]^+$: 516.2481, calculated for $[C_{28}H_{36}N_3O_5]^+$, $[M+Na]^+$: 516.2474.

2,3-bis(benzylcarbamoyl)-7-oxabicyclo[2.2.1]heptan-1-yl)methanaminium trifluoroacetate (26):

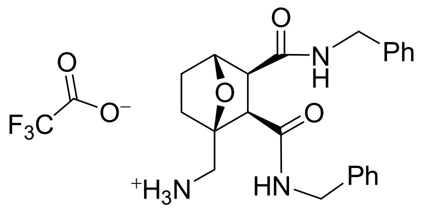

To a stirring solution of **25** (394 mg, 0.80 mmol) in DCM (20 cm^3) precooled at −15°C, a solution of trifluoroacetic acid (TFA 12 cm^3) in DCM (5 cm^3) has been added dropwise and left stirring for 90 minutes. After that time, the solvent and the excess of TFA were evaporated under reduced pressure. The remaining white material was suspended in Et_2O (10 cm^3)and vacuum-filtered. The product **26** was obtained as the white solid (404 mg, 0.79 mmol, 99% yield).

^{1}H-NMR (DMSO-d_6, 600 MHz) δ/ppm: 8.14 (t, $^3J_{H-H}$ = 5.8 Hz, 1H, CON*H*CH_2), 8.06 (br s, 3H, CH2NH_3^+), 7.97 (t, $^3J_{H-H}$ = 5.8 Hz, 1H, CON*H*CH_2), 7.27 (m, 10H, Ph), 4.85 (d, $^3J_{H-H}$ = 4.8 Hz, 1H, oxa-bridge), 4.15 (m, 4H,2x CONHCH_2Ph), 3.49 (m, 1H, C$H_2$$NH_3^+$), 3.25 (m, 1H, C$H_2$$NH_3^+$), 3.21 (d, $^3J_{H-H}$ = 9.7 Hz, 1H, *endo*-H), 3.07 (d, $^3J_{H-H}$ = 9.7 Hz, 1H, *endo*-H), 1.72 (m, 3H, CH_2CH_2), 1.55 (m, 1H, CH_2CH_2).

^{13}C NMR (DMSO-d_6, 150.9 MHz) δ/ppm: 169.9, 169.3, 1158.1, 139.3, 138.7, 128.2, 128.1, 127.5, 127.4, 126.8, 126.7, 83.9, 77.6, 55.6, 53.6, 42.4, 42.3, 39.3, 31.8, 29.9.

FTIR (ATR ν_{max}/cm^{-1}): 3294 (N-H stretch), 1655 (C=O stretch), 1543 (C=O stretch).

HRMS- Q-TOF: Found: $[M - CF_3COO^-]^+$: 394.2134, calculated for $[C_{23}H_{28}N_3O_3]^+$, $[M - CF_3COO^-]^+$: 394.2125.

***N*^{2},*N*^{3}-dibenzyl-1-((3-phenylthioureido)methyl)-7-oxabicyclo[2.2.1]heptane-2,3-dicarboxamide (8):**

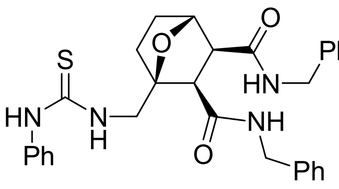

To a stirring solution of **26** (397 mg, 0.78 mmol) in DCM (30 cm^3), *N*,*N*-diisopropylethylamine (103 mg, 0.80 mmol) and phenyl isothiocyanate (108 mg, 0.80 mmol) were added in that order and left to stir at ambient temperature for 24 hours. The solvent and volatiles were then evaporated under reduced pressure. Remained white solid was suspended in DCM (10 cm^3) and vacuum filtered. The product **8** was obtained as a white solid (309 mg, 0.58 mmol, 75% yield).

^{1}H-NMR (DMSO-d$_6$, 600 MHz) δ/ppm: 9.71 (br s, 1H, N*H*), 8.00 (br s, 1H, N*H*), 7.69 (br s, 1H, N*H*), 7.59 (br s, 1H, N*H*), 7.50, (d, $^3J_{H-H}$ = 7.9 Hz, 2H, Ph), 7.28 (m, 12H, Ph), 7.09 (t, $^3J_{H-H}$ = 7.3 Hz, 1H, Ph), 4.86 (d, $^3J_{H-H}$ = 2.5 Hz, 1H, oxa-bridge), 3.16 (d, $^3J_{H-H}$ = 9.6 Hz, 1H, *endo*-H), 2.99 (d, $^3J_{H-H}$ = 9.6 Hz, 1H, *endo*-H), 1.69 (m, 3H, C*H*$_2$C*H*$_2$), 1.49 (m, 1H, CH$_2$C*H*$_2$).

^{13}C NMR (DMSO-d$_6$, 150.9 MHz) δ/ppm: 181.1, 170.3, 168.8, 139.4, 138.9, 128.5, 128.2, 128.1, 127.6, 127.4, 126.8, 126.6, 123.9, 122.6, 86.6, 76.9, 56.5, 53.3, 43.9, 42.4, 42.4, 40.0, 31.0, 30.3.

FTIR (ATR ν_{max}/cm^{-1}): 3288 (N–H stretch), 1649 (C=O stretch), 1538 (C=O stretch).

HRMS (MALDI-TOF/TOF): Found: 529.2278, calculated for $C_{30}H_{32}N_4O_3S$, [M+H]$^+$: 529.2273.

N-((((-2,3-bis(benzylcarbamoyl)-7-oxabicyclo[2.2.1]heptan-1-yl)methyl)amino)(phenylamino) methylene)propan-2-aminium chloride (9):

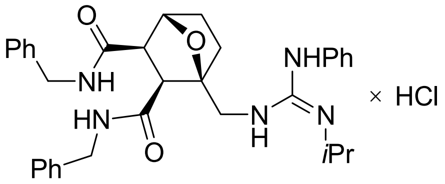

To a stirring solution of **8** (864 mg, 1.63 mmol) in DMF (65 cm^3), Et$_3$N (331 mg, 3.27 mmol) and isopropylamine (145 mg, 2.45 mmol) were added and the mixture was briefly stirred. HgCl$_2$ (448 mg, 1.65 mg) was then added upon which the reaction mixture turned black. The stirring was continued for 48 hours at the ambient temperature. After the reaction was completed, the mixture was vacuum-filtered over a short plug of Celite. The celite was additionally washed with methanol (3×10 cm^3). Volatiles from the filtrate were removed under reduced pressure. To the remained white solid, water (10 cm^3) was added; the insoluble material was vacuum filtered and additionally washed with water (10 cm^3) and dried. Product **9** was isolated as a white solid (824 mg, 1.40 mmol, 85% yield).

^{1}H-NMR (DMSO-d$_6$, 600 MHz) δ/ppm: 9.64 (br s, 1H), 8.10 (br s, 1H), 8.01 (br s, 1H), 7.85 (br s, 1H), 7.45-7.18 (m, 15 H), 4.88 (br s, 1H), 4.23-3.93 (m, 5H), 3.64 (br s, 2H), 3.26 (d, $^3J_{H-H}$ = 9.6 Hz, 1H), 3.04 (d, $^3J_{H-H}$ = 9.3 Hz, 1H), 1.76-1.53 (m, 4H), 1.17 (br s, 6H).

^{13}**C NMR** (DMSO-d$_6$, 150.9 MHz) δ/ppm: 171.3, 169.8, 169.3, 139.5, 138.7, 136.8, 129.5, 128.2, 128.1, 128.1, 128.0, 127.6, 127.4, 127.1, 126.8, 126.6, 86.3, 77.1, 55.3, 53.7, 45.3, 44.7, 42.9, 42.4, 42.3, 42.0, 31.5, 30.7, 29.9, 22.2, 22.0, 20.3, 8.4.

FTIR (ATR v_{max}/cm^{-1}): 3239 (N–H stretch), 1660 (C=O stretch), 1647 (C=O stretch).

HRMS (MALDI-TOF/TOF): Found: 589.2810, calculated for $C_{33}H_{40}ClN_5O_3$, $[M - Cl^-]^+$: calculated 589.2820.

S3.2.3. Synthesis of cocatalysts **10** and **11**.

Tert-butyl ((1,3-dioxo-2-phenyl-2,3,3a,4,7,7a-hexahydro-1H-4,7-epoxyisoindol-4-yl)methyl)carbamate (23):

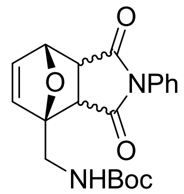

10 cm^3 stainless steel jar was charged with *N*-Boc-furfuryl amine (369 mg, 1.87 mmol), *N*-phenylmaleimide (971 mg, 5.61 mmol), and one stainless steel ball (d= 12 mm). The reaction mixture was ball-milled for 3 h at 30 Hz. After the reaction was completed, the solid material was dissolved in MeOH (20 cm^3), filtered over a short plug of Celite, and additionally washed with 20 cm^3 of MeOH. The organic solvent was evaporated under reduced pressure. The remaining green-yellow solid was suspended in Et_2O (10 cm^3), vacuum filtered, and additionally washed with Et_2O until the white solid remained. The product (407 mg, 1.10 mmol, 57 %) was identified as a mixture of *exo*-**23** and *endo*-**23** in a 1:1.3 ratio.

^{1}**H-NMR** (DMSO-d$_6$, 600 MHz) δ/ppm:

egzo: 7.09 (t, $^3J_{H\text{-}H}$ = 6.0 Hz, 1H, N*H*), 6.47 (d, $^3J_{H\text{-}H}$ = 5.7 Hz, 1H, HC=C*H*), 5.19 (d, $^3J_{H\text{-}H}$ = 1.5 Hz, 1H, oxa-bridge), 3.43 (dd, $^3J_{H\text{-}H}$ = 6.0 Hz, $^3J_{H\text{-}H}$ = 14.8 Hz, 1H, C*H*$_2$NHBoc), 3.22 (d, $^3J_{H\text{-}H}$ = 6.5 Hz, 1H, *endo*-H), 3.10 (d, $^3J_{H\text{-}H}$ = 6.5 Hz, 1H, *endo*-H)

endo: 7.09 (t, $^3J_{H\text{-}H}$ = 6.2 Hz, 1H, N*H*), 6.50 (d, $^3J_{H\text{-}H}$ = 5.7 Hz, 1H, HC=C*H*), 5.32 (dd, $^3J_{H\text{-}H}$ = 1.2 Hz, $^3J_{H\text{-}H}$ = 5.5 Hz, 1H, oxa-bridge), 3.53 (dd, $^3J_{H\text{-}H}$ = 5.6 Hz, $^3J_{H\text{-}H}$ = 14.9 Hz, 1H, C*H*$_2$NHBoc), 3.47 (d, $^3J_{H\text{-}H}$ = 7.6 Hz, 1H, *exo*-H)

The remaining signals are overlapping and could not be assigned to an individual isomer: 7.51-7.10 (2 × Ph), 6.60 (2×*H*C=CH), 3.87-3.79 (C*H*$_2$NHBoc and *exo*-H), 1.38 (2×Boc).

^{13}**C NMR** (DMSO-d$_6$, 150.9 MHz) δ/ppm: 175.4, 174.2, 174.0, 155.8, 137.8, 137.4, 135.7, 135.5, 132.0, 131.9, 128.9, 128.8, 128.4, 128.4, 126.9, 126.7, 91.4, 91.0, 80.5, 78.6, 77.9, 77.8, 50.4, 48.5, 47.9, 47.5, 40.8, 28.2

FTIR (ATR v_{max}/cm^{-1}): 3407 (N–H stretch), 1699 (C=O stretch).

HRMS- Q-TOF: Found: $[M+Na]^+$: 393.1427, calculated for $[C_{20}H_{22}N_2NaO_5]^+$, $[M+Na]^+$: 393.1421; found $[2M+Na]^+$: 763.2966, calculated for $[C_{40}H_{44}N_4NaO_{10}]^+$, $[2M+Na]^+$: 763.2950.

Tert-butyl (((4S,7R)-1,3-dioxo-2-phenyloctahydro-1H-4,7-epoxyisoindol-4-yl)methyl)carbamate (27):

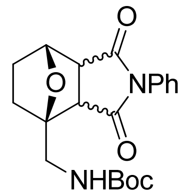

A round bottom flask was charged with **26** (787 mg, 2.12 mmol), methanol (15 cm^3), and Pd/C 10% (258 mg), and reduced under a hydrogen atmosphere (1 atm) for 18 h. The reaction mixture was filtered over a short plug of Celite and additionally washed with methanol (3×10 cm^3). After solvent evaporation under reduced pressure, the remaining white solid was identified as *exo*-**27** and *endo*-**27** in a 1:1.3 ratio, yielding 783 mg, 2.10 mmol, 99%.

^{1}H-NMR (DMSO-d_6, 600 MHz) δ/ppm:

egzo: 6.72 (t, $^3J_{H\text{-}H}$ = 5.9 Hz, 1H, N*H*), 4.74 (d, $^3J_{H\text{-}H}$ = 4.9 Hz, 1H, oxa-bridge), 3.31 (d, $^3J_{H\text{-}H}$ = 7.2 Hz, 1H, *endo*-H), 3.25 (d, $^3J_{H\text{-}H}$ = 7.2 Hz, 1H, *endo*-H)

endo: 7.00 (t, $^3J_{H\text{-}H}$ = 5.9 Hz, 1H, N*H*), 4.84 (t, $^3J_{H\text{-}H}$ = 5.7 Hz, 1H, oxa-bridge), 3.66 (ddd, $^3J_{H\text{-}H}$ = 1.7 Hz, $^3J_{H\text{-}H}$ = 6.2 Hz, $^3J_{H\text{-}H}$ = 9.7 Hz, 1H, *exo*-H), 3.38 (d, $^3J_{H\text{-}H}$ = 9.7 Hz, 1H, *exo*-H)

The remaining signals are overlapping and could not be assigned to an individual isomer: 7.53-7.41 (m, Ph), 7.27-7.24 (m, Ph), 7.21-7.20 (m, Ph) 3.57-3.44 (m, 2×CH_2NHBoc), 1.99-1.57 (m, 2×CH_2CH_2) 1.39 (2×Boc).

^{13}C NMR (DMSO-d_6, 150.9 MHz) δ/ppm: 176.9, 176.6, 175.2, 174.8, 174.7, 155.8, 155.7, 132.2, 131.9, 129.1, 128.9, 128.8, 128.6, 128.4, 128.1, 127.1, 127.0, 126.8, 88.1, 87.3, 78.5, 77.9, 77.8, 76.8, 52.8, 52.2, 51.1, 50.7, 42.4, 40.5, 30.3, 29.0, 28.4, 28.2, 28.1, 27.8, 26.9.

FTIR (ATR ν_{max}/cm^{-1}): 3352 (N-H stretch), 2975 (C-H stretch), 1698 (C=O stretch).

HRMS- Q-TOF: Found: $[M+Na]^+$: 395.1581, calculated for $[C_{15}H_{17}N_2O_3]^+$, $[M+Na]^+$: 395.1583; Found $[2M+Na]^+$: 767.3283, calculated for $[C_{40}H_{48}N_4NaO_{10}]^+$, $[2M+Na]^+$: 767.3263.

(1,3-dioxo-2-phenyloctahydro-1H-4,7-epoxyisoindol-4-yl)methanaminium trifluoroacetate (28):

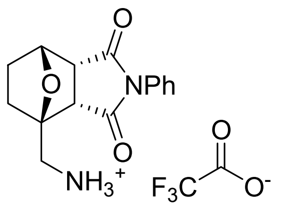

The round bottom flask was charged with **27** (714 mg, 1.92 mmol) and DCM (25 cm^3), and the reaction mixture was cooled down to −15°C. To the cooled mixture, a solution of trifluoroacetic acid (TFA, 7 cm^3) in DCM (10 cm^3) has been added dropwise. The reaction mixture was stirred for 90 minutes. After that time,

the solvent and excess of TFA were evaporated under reduced pressure. To the remaining yellowish oil, EtOAc (10 cm^3) was added and left at 4°C for 16 hours during which time the white solid precipitated. The precipitate was vacuum-filtered and washed with cold EtOAc (5 cm^3). The product was identified as pure *endo*-isomer **28** (318 mg, 0.82 mmol, 42% yield).

^{1}H-NMR (DMSO-d_6, 600 MHz) δ/ppm: 8.30 (br s, 3H, $CH_2N\mathit{H}_3^+$), 7.51-7.53 (m, 2H, Ph), 7.44-7.47 (m, 1H, Ph), 7.27-7.28 (m, 2H, Ph), 5.00 (t, $^3J_{H-H}$ = 5.7 Hz, 1H, oxa-bridge), 3.72-3.75 (m, 1H, $C\mathit{H}_2NH_3^+$), 3.62 (dd, $^2J_{H-H}$ = 9.8 Hz, $^3J_{H-H}$ = 2.0 Hz, 1H, $C\mathit{H}_2NH_3^+$), 3.48 (d, $^3J_{H-H}$ = 14.2 Hz, 1H, *exo*-H), 3.38 (d, overlapping with water in DMSO- d_6, *exo*-H), 1.84-1.95 (m, 2H, $C\mathit{H}_2CH_2$), 1.65-1.76 (m, 2H, $CH_2C\mathit{H}_2$).

^{13}C NMR (DMSO-d_6, 150.9 MHz) δ/ppm: 174.4, 158.3, 158.1, 131.8, 129.1, 128.7, 127.0, 118.2, 116.2, 85.2, 77.5, 52.6, 52.0, 40.8, 40.0, 28.0, 27.0.

FTIR (ATR v_{max}/cm^{-1}): 3019 (N–H stretch), 2892 (C–H stretch), 1705 (C=O stretch).

HRMS- Q-TOF: Found: $[M - CF_3COO^-]^+$: 273.1243, calculated for $[C_{15}H_{17}N_2O_3]^+$, $[M - CF_3COO^-]^+$: 273.1234

1-((1,3-dioxo-2-phenyloctahydro-1H-4,7-epoxyisoindol-4-yl)methyl)-3-phenylthiourea (10):

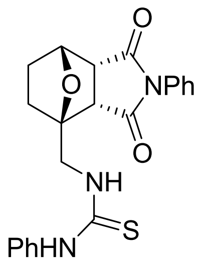

To a stirring solution of **28** (349 mg, 0.93 mmol) in DCM (20 cm^3), triethylamine (122 mg, 1.20 mmol) and subsequently phenyl isothiocyanate (148 mg, 1.10 mmol) were added and left to stir at the ambient temperature for 18 hours. After reaction completion, the solvent and volatiles were evaporated under reduced pressure. The remaining solid was dissolved in DCM (5 cm^3) and passed through a short plug of silica gel (9 g). The first fraction (unreacted phenyl isothiocyanate) was collected by washing silica gel with DCM until it could not be seen on TLC. The second fraction (product) was eluted with EtOAc. Solvent was evaporated under reduced pressure leaving product **10** as the white solid (397 mg, 0.90 mmol, 96%).

^{1}H-NMR (DMSO-d_6, 600 MHz) δ/ppm: 9.73 (br s, 1H, N*H*), 7.80 (br s, 1H, N*H*), 7.51-7.54 (m, 4H, Ph), 7.44-7.47 (m, 1H, Ph), 7.27-7.33 (m, 4H, Ph), 7.09-7.11 (m, 1H, Ph), 4.94 (t, $^3J_{H-H}$ = 5.7 Hz, 1H, oxa-bridge), 4.29 (br s, 1H, $C\mathit{H}_2NHCS$), 4.06 (dd, $^2J_{H-H}$= 14.4 Hz, $^3J_{H-H}$= 5.0 Hz, 1H, $C\mathit{H}_2NHCS$), 3.72 (ddd, $^3J_{H-H}$= 9.7 Hz, $^3J_{H-H}$= 6.2 Hz, $^3J_{H-H}$= 1.6 Hz, exo-H), 3.48 (dd, $^3J_{H-H}$= 9.8 Hz, $^3J_{H-H}$=2.0 Hz, 1H, exo-H), 1.88-1.94 (m, 1H, $C\mathit{H}_2CH_2$), 1.65-1.79 (m, 3H. $C\mathit{H}_2C\mathit{H}_2$).

^{13}C NMR (DMSO-d_6, 150.9 MHz) δ/ppm: 181.3, 174.7, 174.5, 139.4, 131.9, 129.1, 128.6, 128.4, 127.0, 124.1, 122.7, 87.8, 77.0, 52.8, 52.3, 45.7, 27.9, 27.0.

FTIR (ATR v_{max}/cm^{-1}): 3306 (N–H stretch), 2959 (C–H stretch), 1705 (C=O stretch).

HRMS (MALDI-TOF/TOF): Found: $[M+H]^+$ 408.1380, calculated for $[C_{22}H_{21}N_3O_3S]^+$, $[M+H]^+$: 408.1382,.

2-(((1,3-dioxo-2-phenyloctahydro-1H-4,7-epoxyisoindol-4-yl)methyl)amino)-4,5-dihydro-1H-imidazol-3-ium iodide (11):

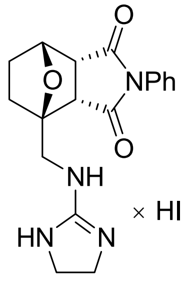

The round bottom flask was charged with THF (5 cm^3), **28** (300 mg, 0,80 mmol), imidazolidin-2-ylidene(methyl)sulfonium iodide (235 mg, 0.96 mmol), and triethylamine (82 mg, 0.81 mmol) and the flask was topped with a condenser. The reaction mixture has been heated at 55°C with stirring for 18 hours. After solvent evaporation under reduced pressure, a white solid remained. Solid was suspended in water (5 cm^3), vacuum filtered and the remaining material was washed water (2×1 cm^3). The white solid precipitates from the water mother liquor, which is then vacuum filtered, dried, and identified as **11** (232 mg, 0.5 mmol, 62% yield).

^{1}H-NMR (DMSO-d$_6$, 600 MHz) δ/ppm: two NH protons are broadened between 8.64 and 7.48 ppm, 7.52 (t, $^3J_{H\text{-}H}$ = 7.5 Hz, 2H, Ph), 8.50 (t, $^3J_{H\text{-}H}$ = 6.1 Hz, 1H, CH_2NH), 7.46 (t, $^3J_{H\text{-}H}$ = 7.4 Hz, 1H, Ph), 7.27 (d, $^3J_{H\text{-}H}$ = 7.5 Hz, 2H, Ph), 4.96 (t, $^3J_{H\text{-}H}$ =5.7 Hz, 1H, oxa-bridge), 3.72 (m, 3H, overlapping *exo*-H and CH_2NH), 3.61 (br s, 4H, CH_2CH_2 ring), 3.44 (d, $^3J_{H\text{-}H}$ = 9.8 Hz, 1H, *exo*-H), 1.90 (m, 1H, CH_2CH_2), 1.66-1.75 (m, 3H, CH_2CH_2).

^{13}C NMR (DMSO-d$_6$, 150.9 MHz) δ/ppm: 174.6, 174.5, 131.8, 129.1, 128.7, 126.9, 87.2, 77.2, 52.4, 52.2, 44.3, 42.5, 28.0, 27.0.

FTIR (ATR ν_{max}/cm^{-1}): 3336 (N-H stretch), 1710 (C=O stretch), 1656 (C=O stretch).

HRMS (MALDI-TOF/TOF): Found: $[M+H]^+$ 469.0730, calculated for $C_{18}H_{21}IN_4O_3$, $[M+H]^+$: 469.0737.

S3.2.4. Synthesis of cocatalyst **15**.

1,3-Diisopropyl-2-((2,3-dimethoxycarbonyl-7-oxabicyclo[2.2.1]hepta-2,5-dien-1-yl)methyl)guanidinium hexafluorphosphate (15)

The crude product obtained by the cycloaddition of N^1,N^3-diisopropyl-N^2-furfurylguanidinium hexafluorophosphate (**2**) (369 mg, 1.0 mmol) and DMAD-a (740 µL, 856 mg, 6.0 mmol) in acetonitrile (1 cm^3) as described previously[3], was dissolved in acetonitrile (5 cm^3). To the solution, *N,N',N''-tris*-(3-dimethylaminopropyl)guanidine (314 mg, 1.0 mmol) was added. The reaction mixture was stirred for 16 h at room temperature. The solvent is then evaporated and the remaining dark brown viscous mass was filtered over short silica column (7.5 g) and eluted with EtOAc (50 cm^3) and acetonitrile (100 cm^3). The fractions were collected and evaporated. The remaining reddish-brown viscous oil was treated with EtOAc. Shortly after, the white crystalline precipitate was formed. The precipitated product (312 mg, 0,61 mmol, 61%) was identified as the hexafluorophosphate salt **15**.

^{1}H-NMR (CD_3CN, 600 MHz) δ/ppm: 6.67 (br s, 1H), 6.98 (dd, 1H, $^3J_{HH}$ = 5.7 Hz, $^3J_{HH}$ =1.7 Hz), 6.14 (d, 1H, $^3J_{HH}$ = 5.7 Hz), 5.19 (dd, 1H, $^3J_{HH}$ = 4.4 Hz $^3J_{HH}$ = 1.7 Hz), 5.00 (br d, 1H, $^3J_{HH}$ = 6.4 Hz), 4.08 (hep, 1H, $^3J_{HH}$ = 7.0 Hz), 3.94 (d, 1H, $^3J_{HH}$ = 14.7 Hz), 3.71–3.77 (m, 1H), 3.72 (s, 3H), 3.70 (d, 1H, $^3J_{HH}$ = 14.5 Hz), 3.51 (d, 1H, $^3J_{HH}$ = 4.4 Hz), 3.66 (s, 3H,), 1.57 (d, 3H, $^3J_{HH}$ = 7.0 Hz), 1.40 (d, 3H, $^3J_{HH}$ = 7.0 Hz), 1.33 (d, 3H, $^3J_{HH}$ = 6.4 Hz), 1.30 (d, 3H, $^3J_{HH}$ = 6.4 Hz),

^{13}C-NMR (CD_3CN, 150.9 MHz) δ/ppm: 169.7, 168.5, 151.6, 143.2, 131.6, 88.4, 80.5, 77.3, 58.2, 55.5, 54.3, 52.8, 46.4, 38.5, 22.5, 22.5, 22.3, 19.7.

FTIR–ATR ν_{max}/cm^{-1}: 3327 (N–H stretch), 1596 (C=O stretch), 833 (P–F stretch).

HRMS (MALDI-TOF/TOF): $C_{18}H_{28}N_3O_5$ $[M-PF_6^-]^+$: calculated: 366.2029, found: 366.2038.

S3.3. NMR spectra

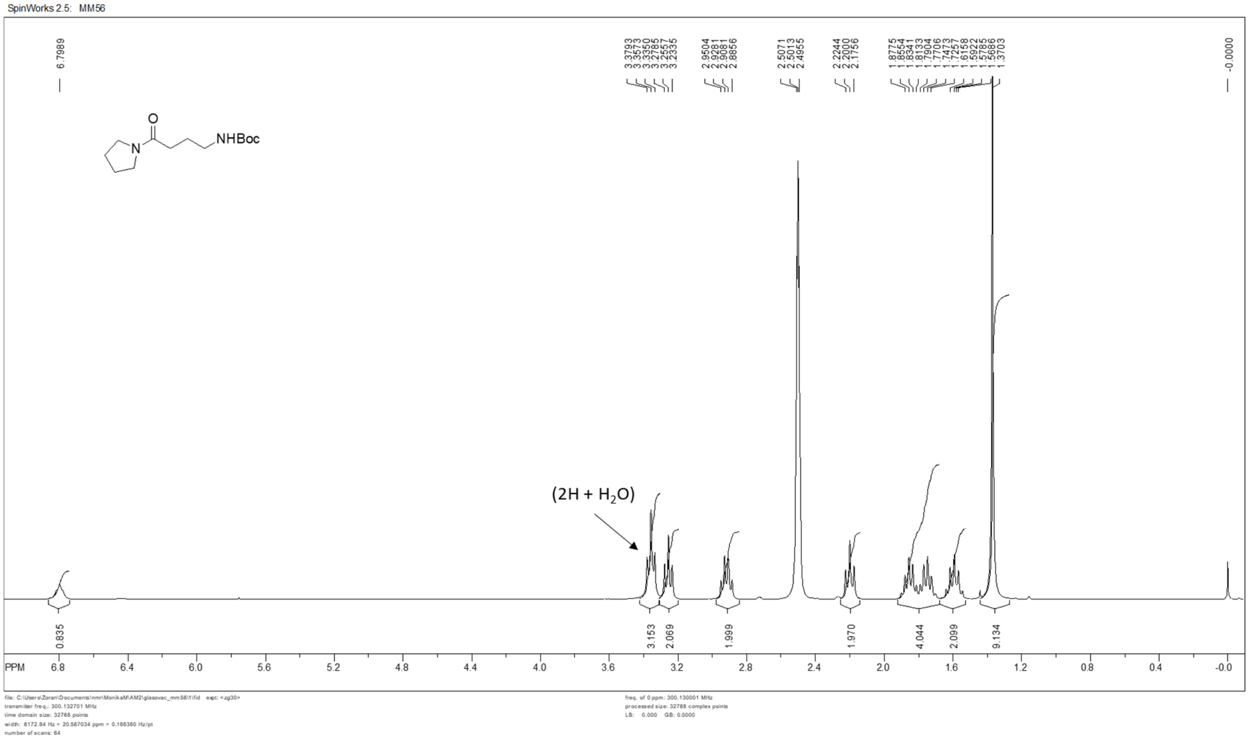

Figure S6. ^{1}H-NMR (DMSO-d$_6$, 300 MHz) spectrum of **32**.

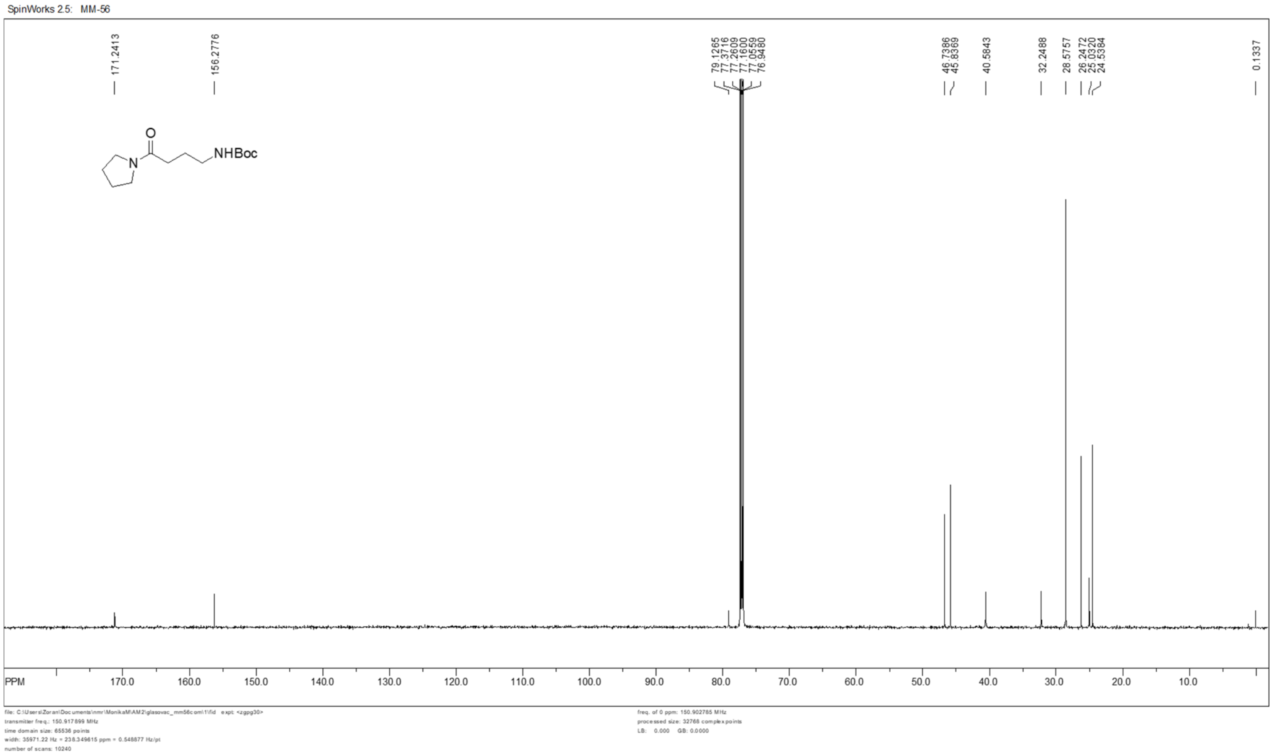

Figure S7. ^{13}C-NMR (CDCl$_3$, 150.9 MHz) spectrum of **32**.

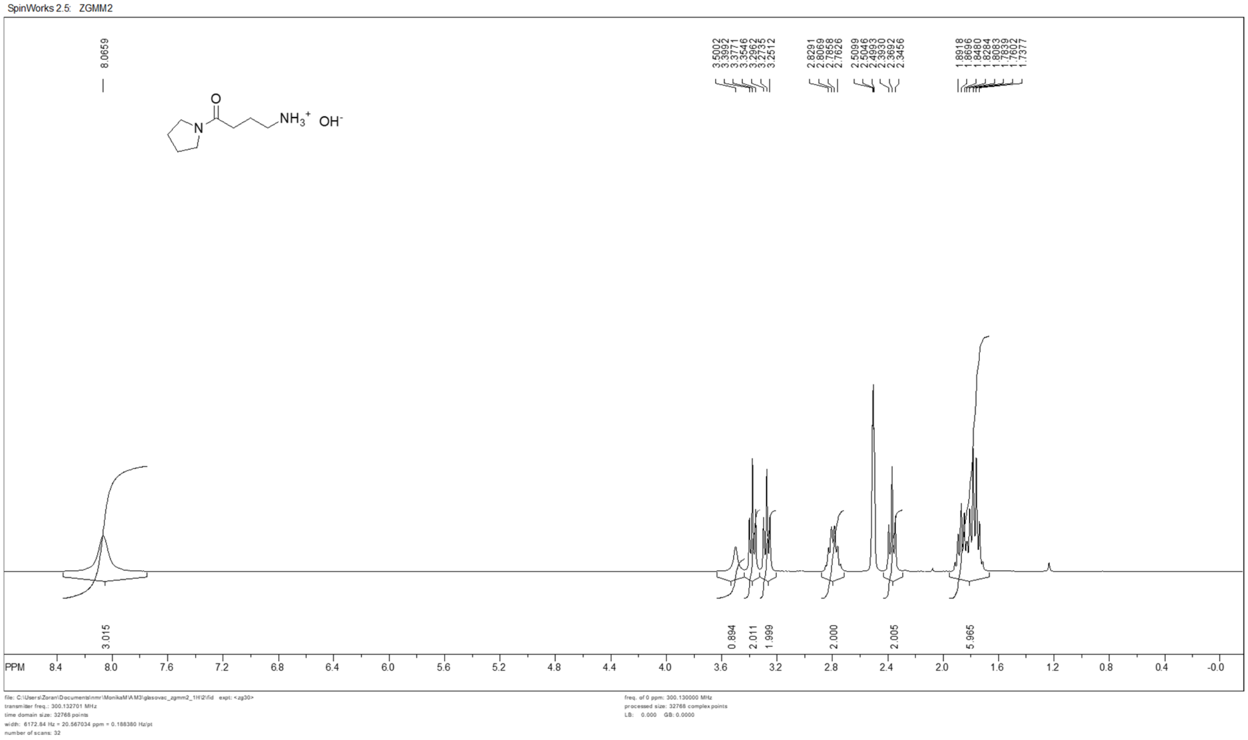

Figure S8. ^{1}H-NMR (DMSO-d_6, 300 MHz) spectrum of **33**.

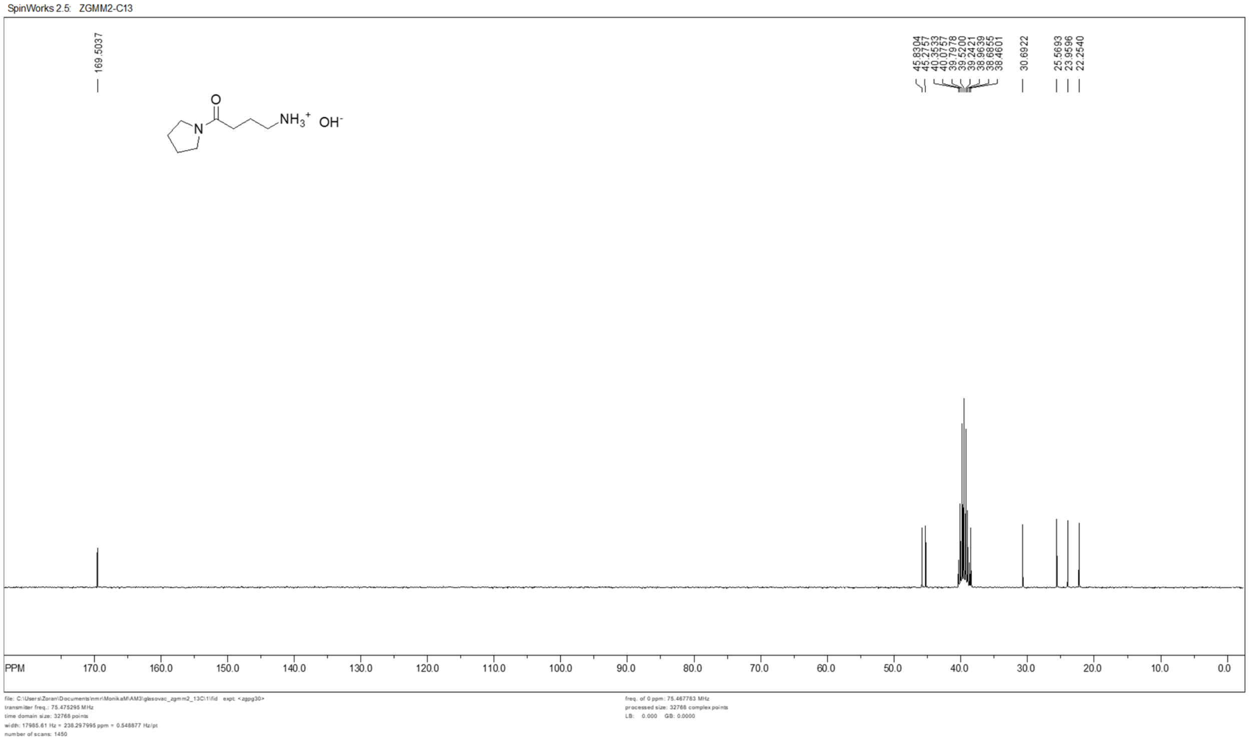

Figure S9. ^{13}C-NMR (DMSO-d_6, 75.5 MHz) spectrum of **33**.

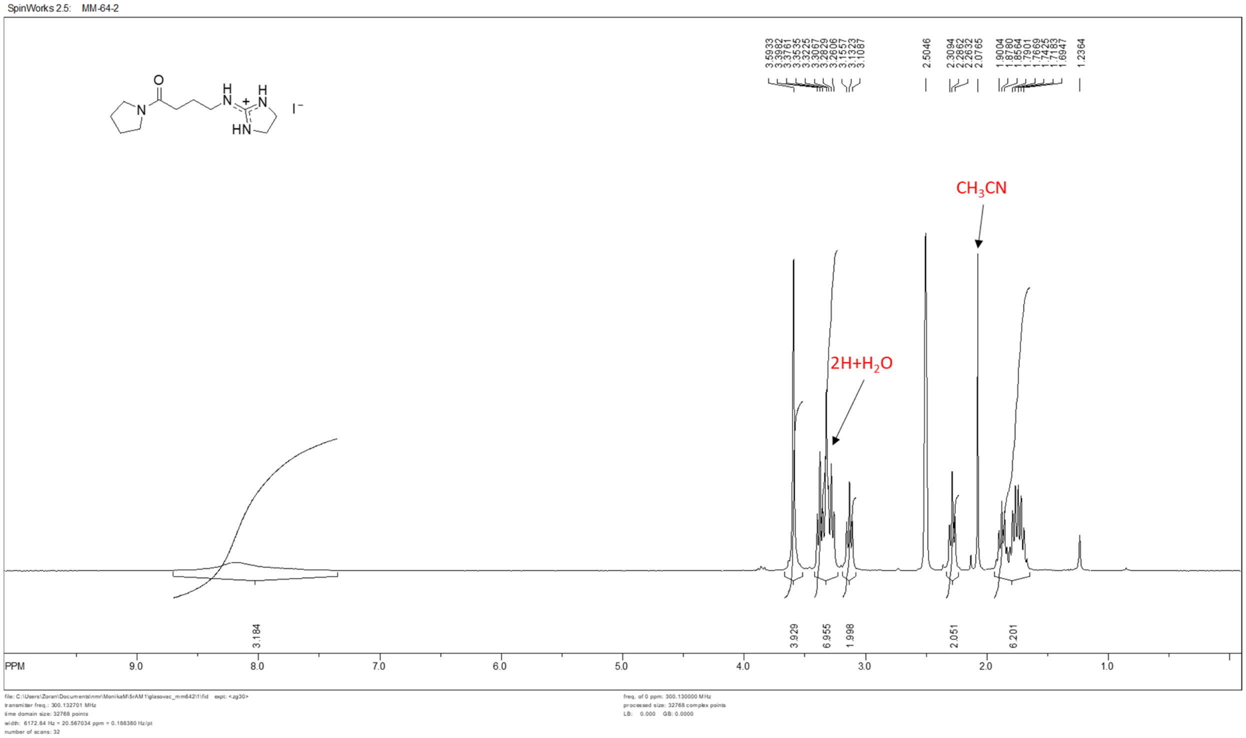

Figure S10. ^{1}H-NMR (DMSO-d_6, 300 MHz) spectrum of **7**.

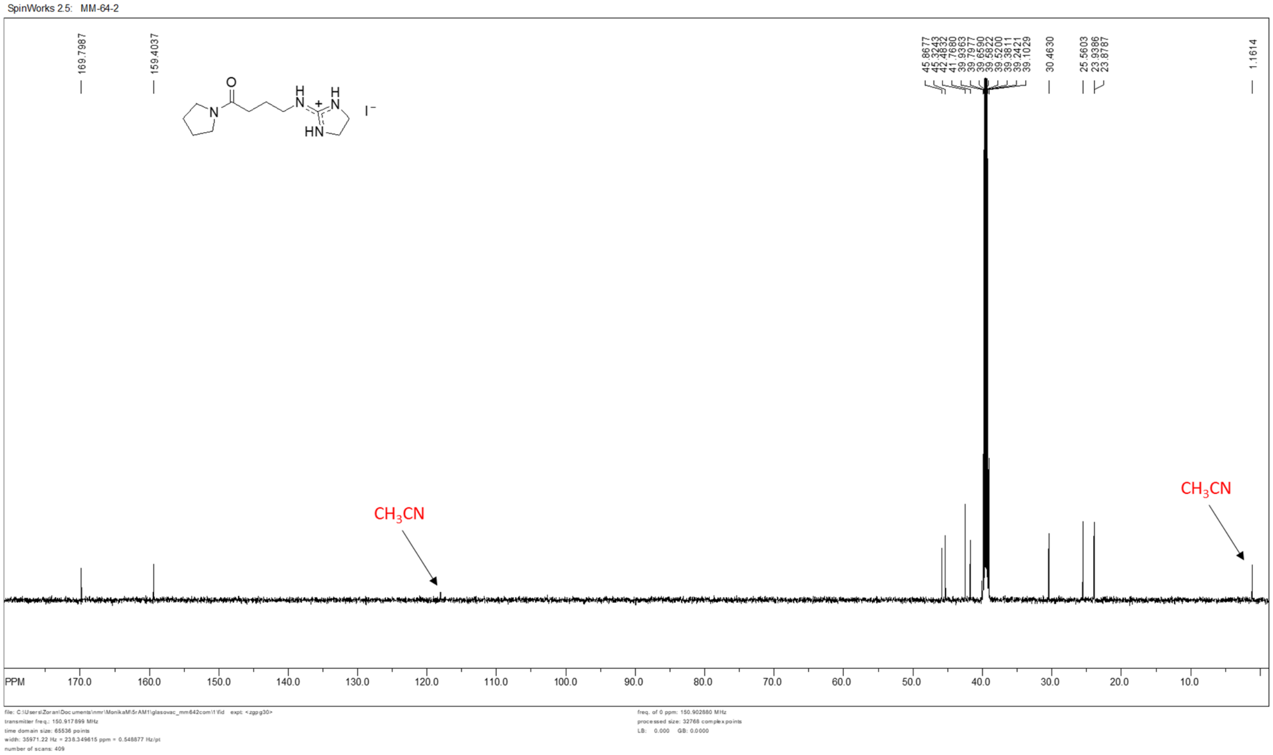

Figure S11. ^{13}C-NMR (DMSO-d_6, 150.9 MHz) spectrum of **7**.

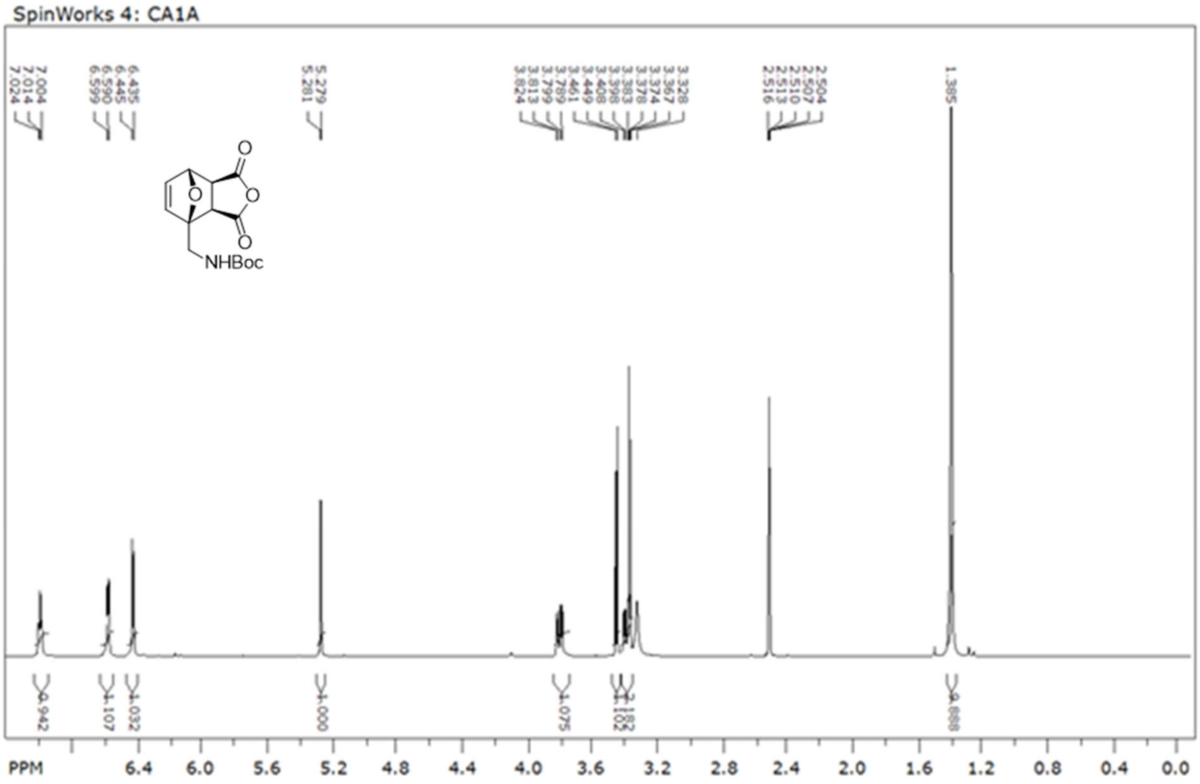

Figure S12. **1**H-NMR (DMSO-d$_6$, 600 MHz) spectrum of **22**.

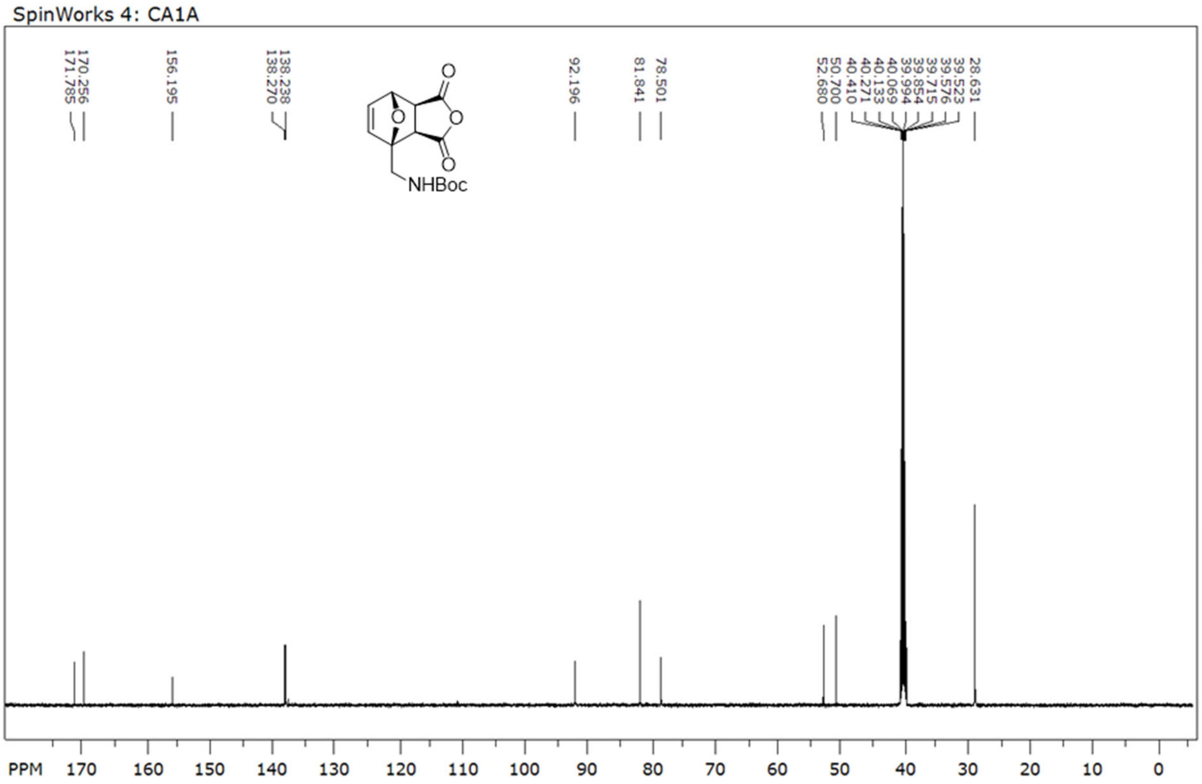

Figure S13. ^{13}C NMR (DMSO-d$_6$, 150.9 MHz) spectrum of **22**.

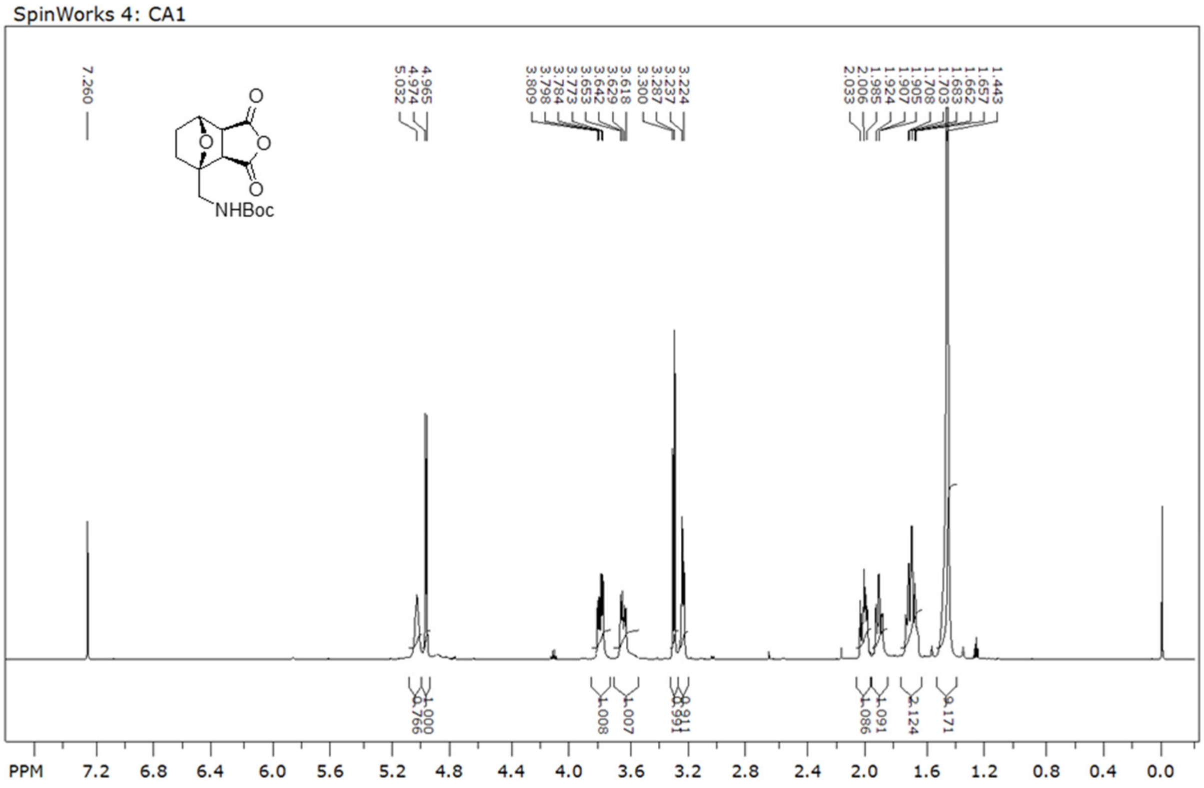

Figure S14. **^{1}**H-NMR ($CDCl_3$, 600 MHz) spectrum of **24**.

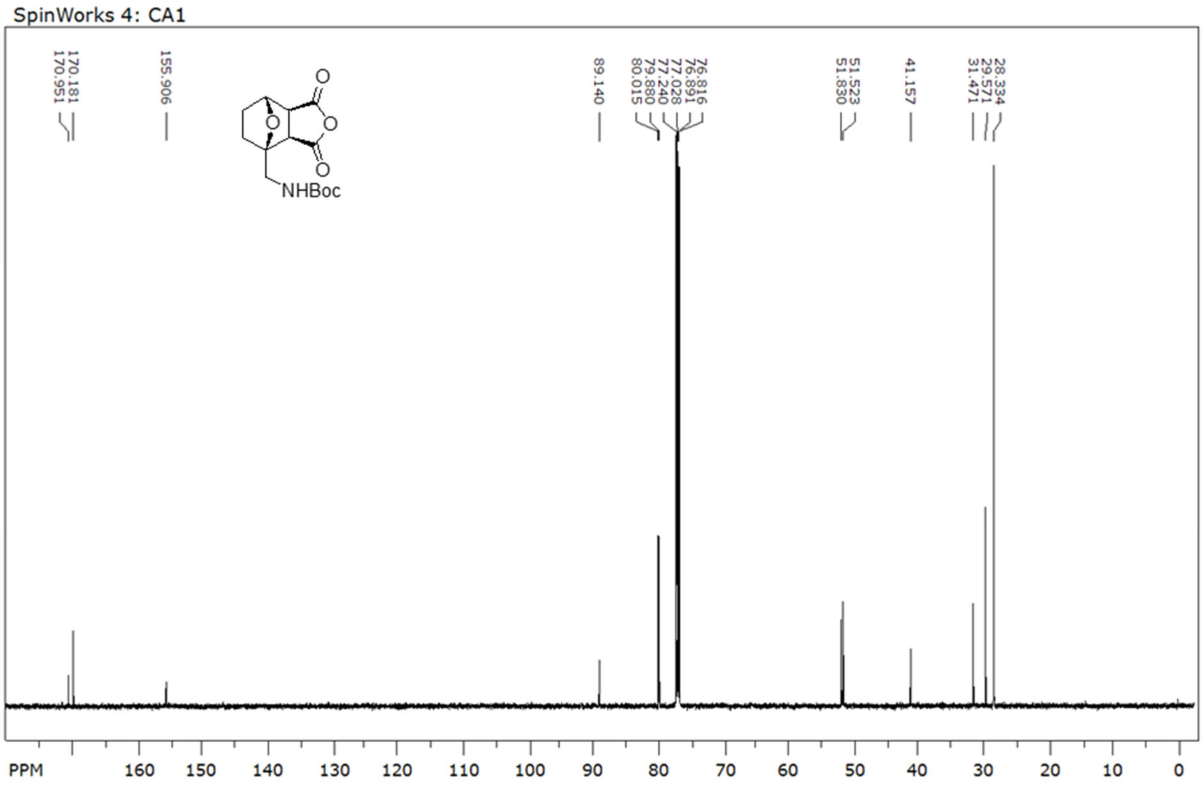

Figure S15. ^{13}C NMR ($CDCl_3$, 150.9 MHz) spectrum of **24**.

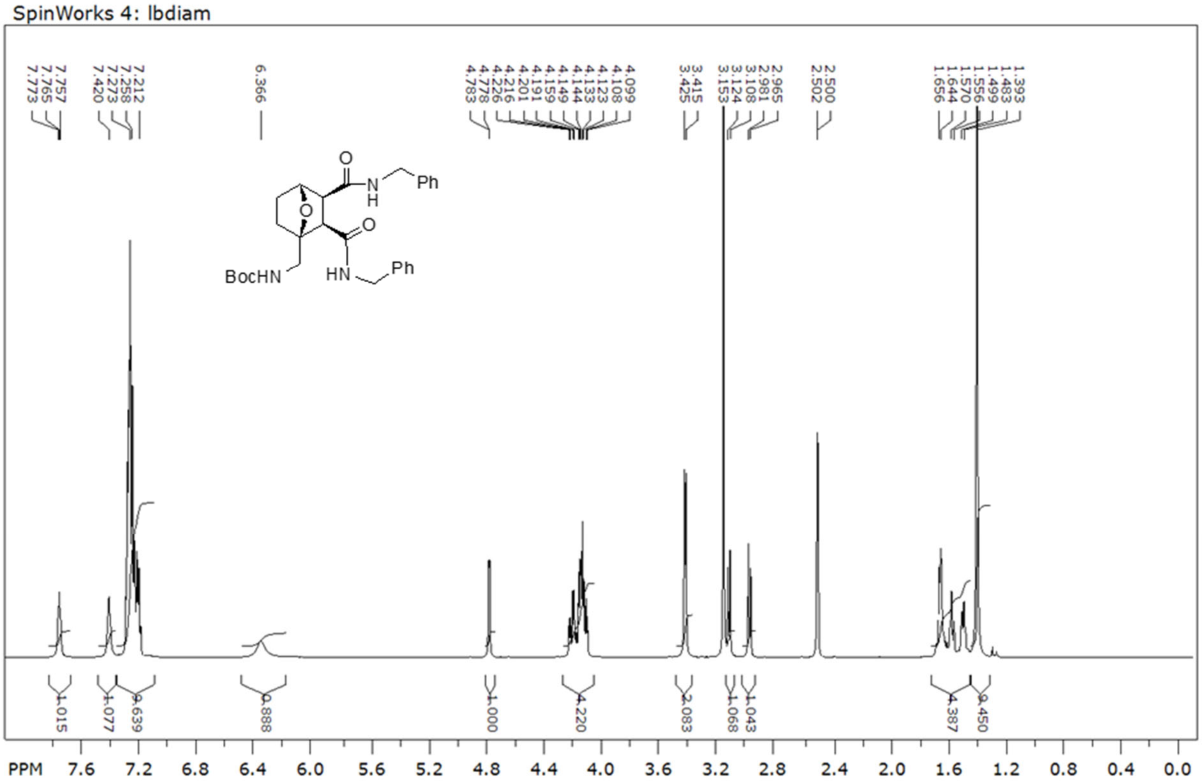

Figure S16. ^{1}H-NMR (DMSO-d_6, 600 MHz) spectrum of **25**.

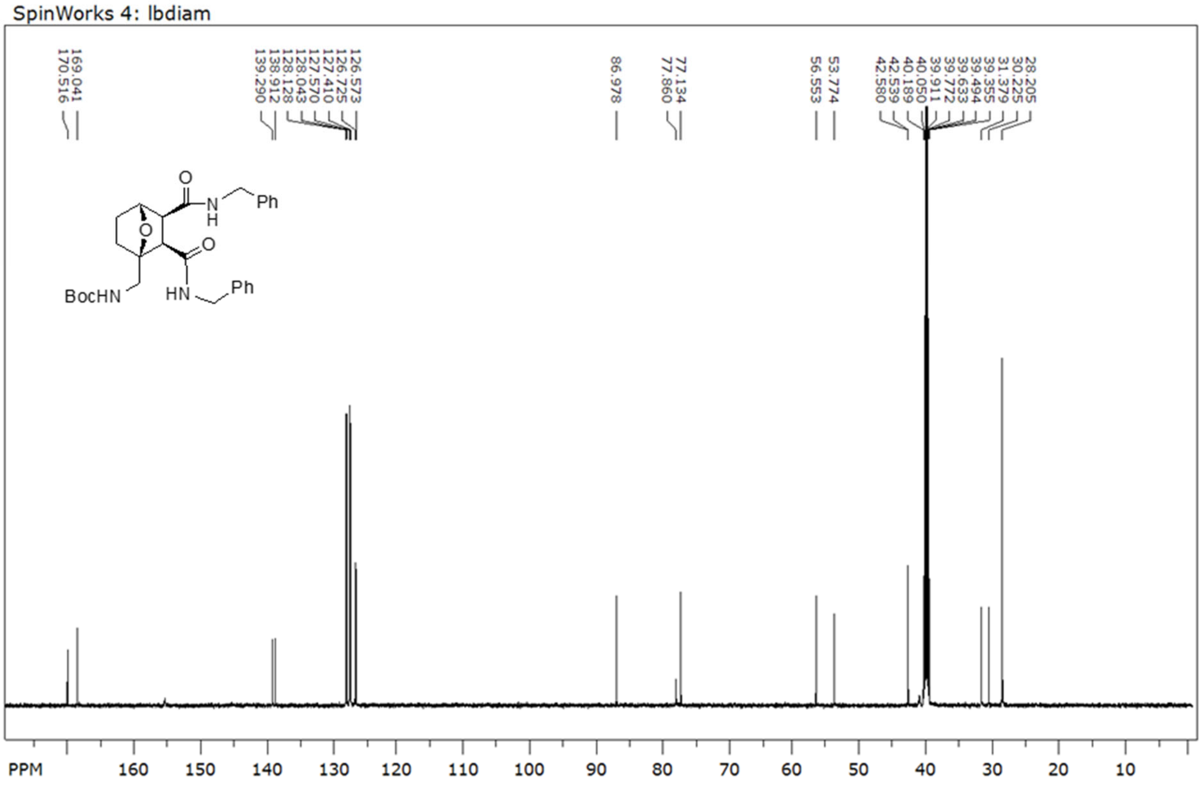

Figure S17. ^{13}C NMR (DMSO-d_6, 150.9 MHz) spectrum of **25**.

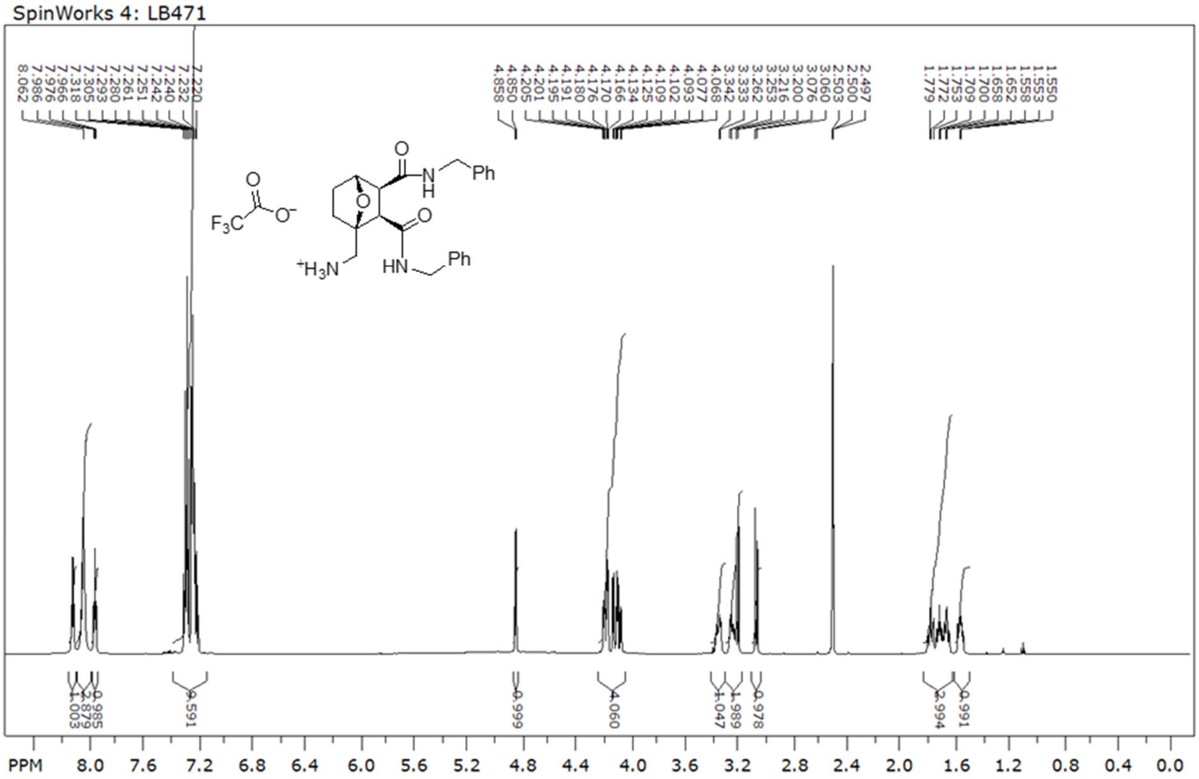

Figure S18. **1**H-NMR (DMSO-d_6, 600 MHz) spectrum of **26**.

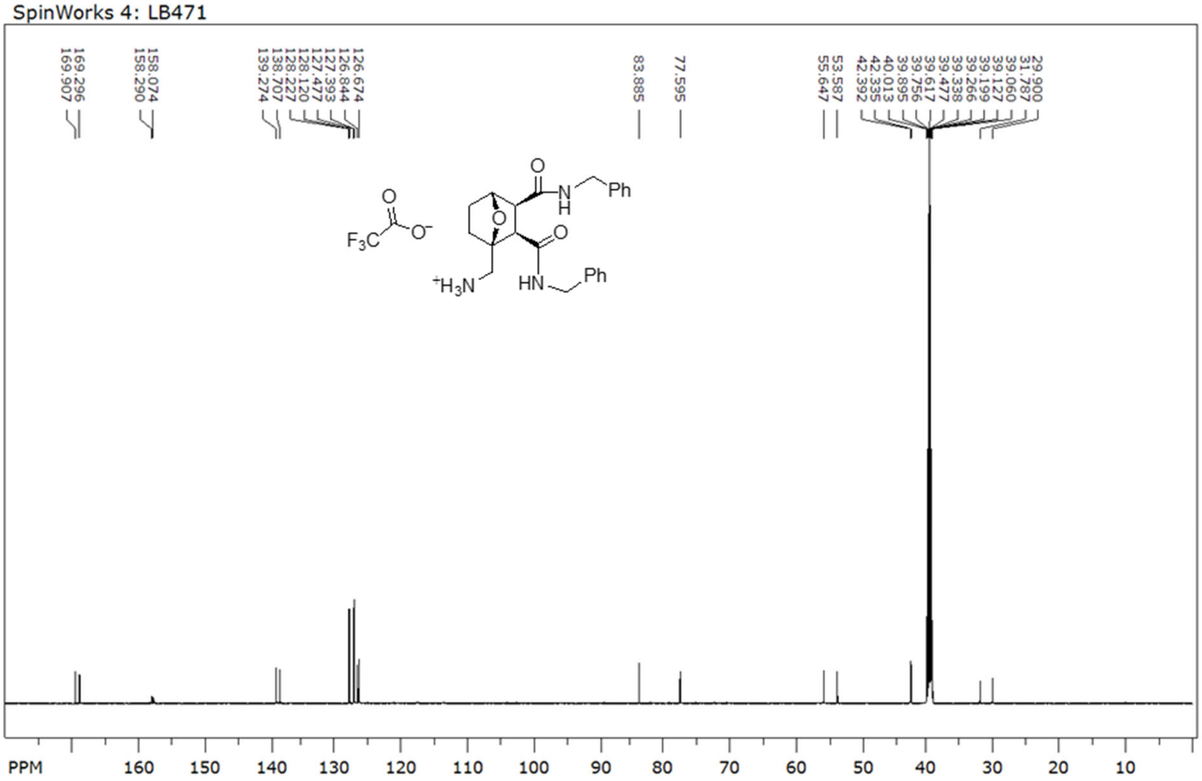

Figure S19. ^{13}C NMR (DMSO-d_6, 150.9 MHz) spectrum of **26**.

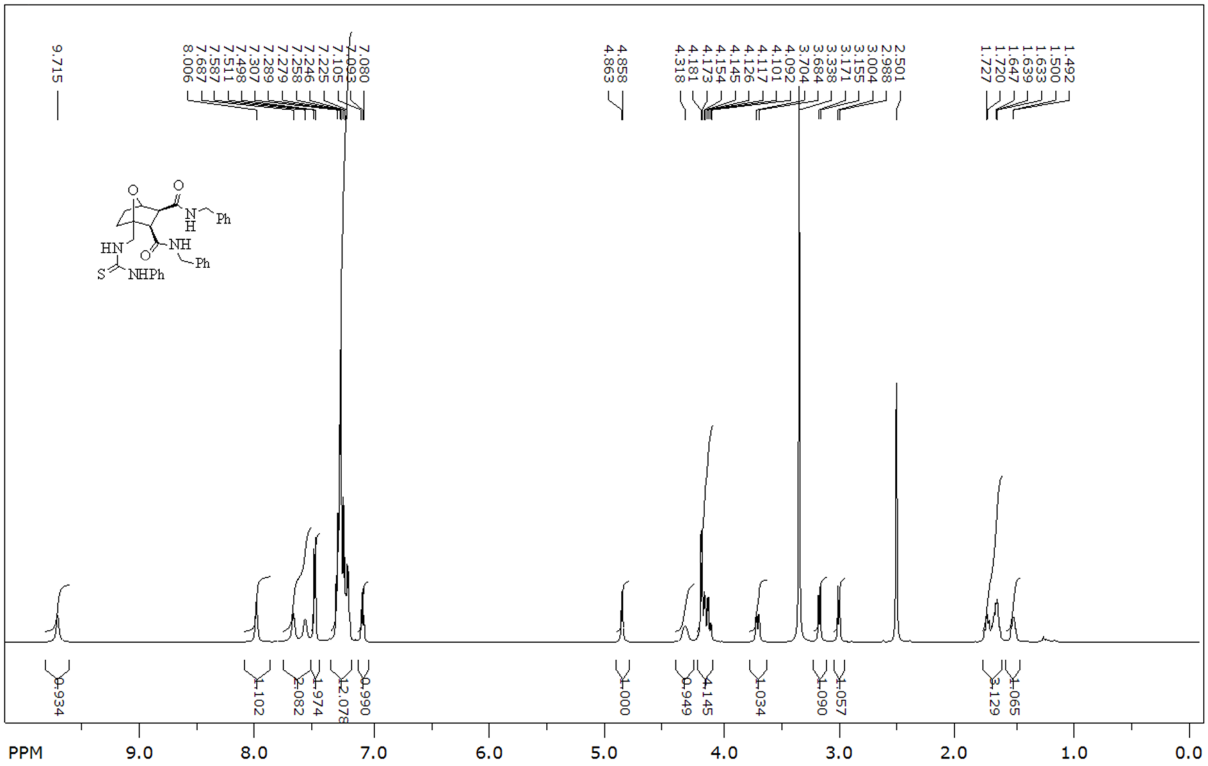

Figure S20. ^{1}H-NMR (DMSO-d$_6$, 600 MHz) spectrum of **8**.

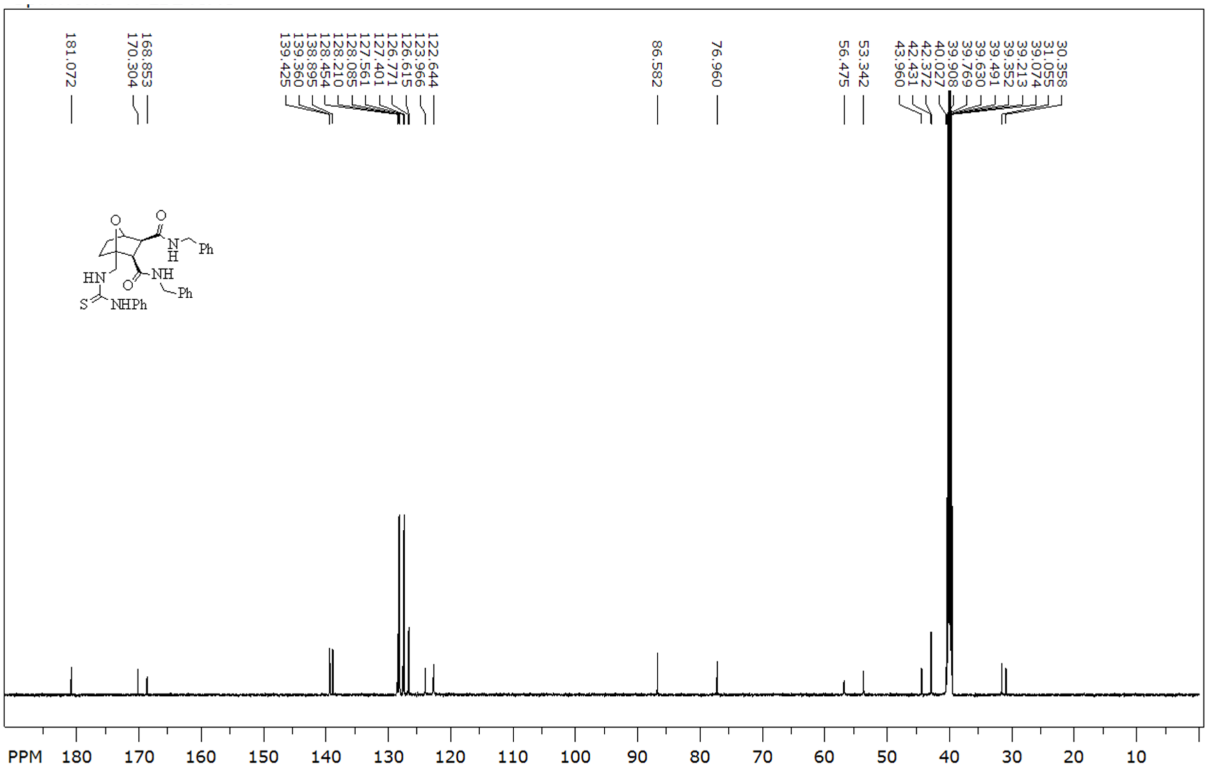

Figure S21. ^{13}C-NMR (DMSO-d$_6$, 150.9 MHz) spectrum of **8**.

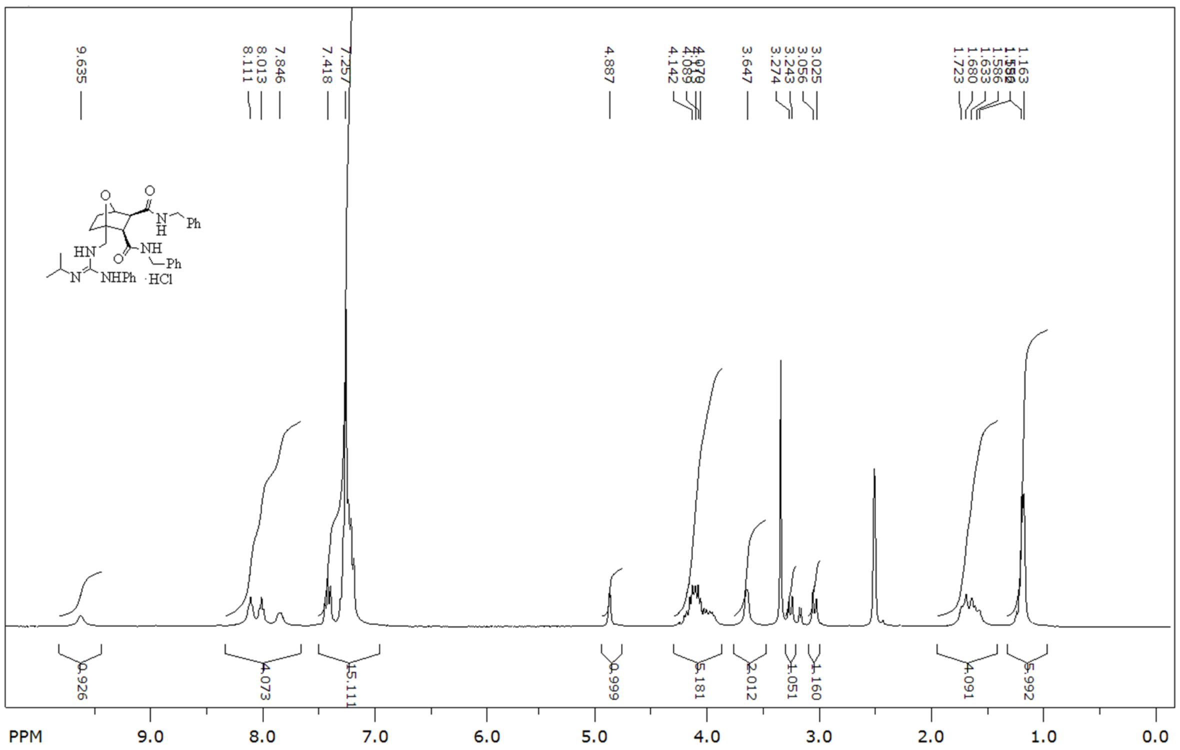

Figure S22. ^{1}H-NMR (DMSO-d$_6$, 600 MHz) spectrum of **9**.

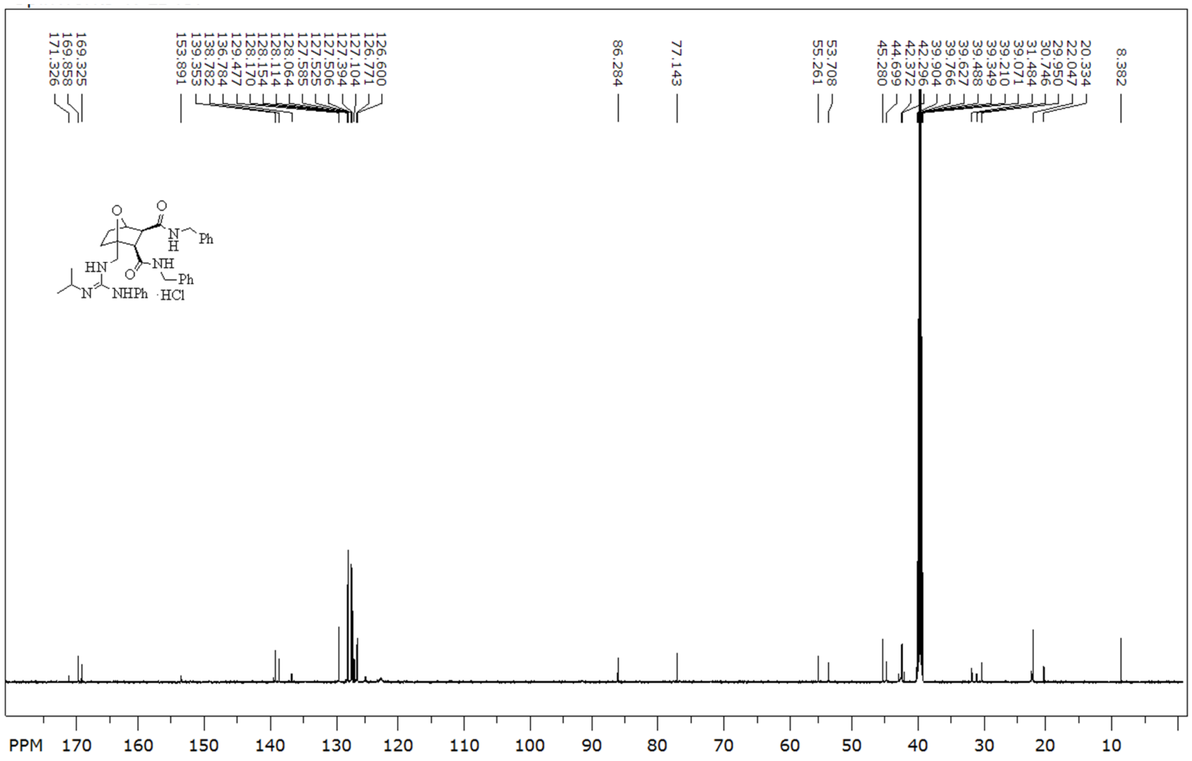

Figure S23. ^{13}C-NMR (DMSO-d$_6$, 150.9 MHz) spectrum of **9**.

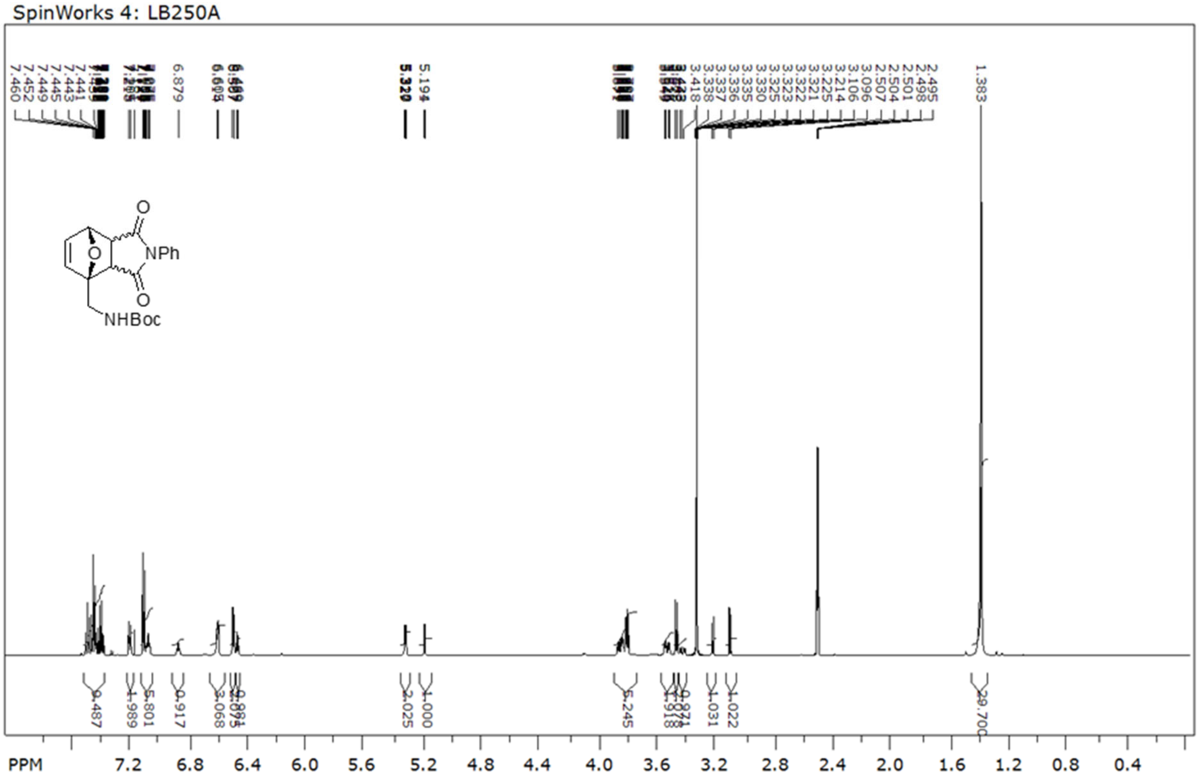

Figure S24. ^{1}H-NMR (DMSO-d$_6$, 600 MHz) of **23**.

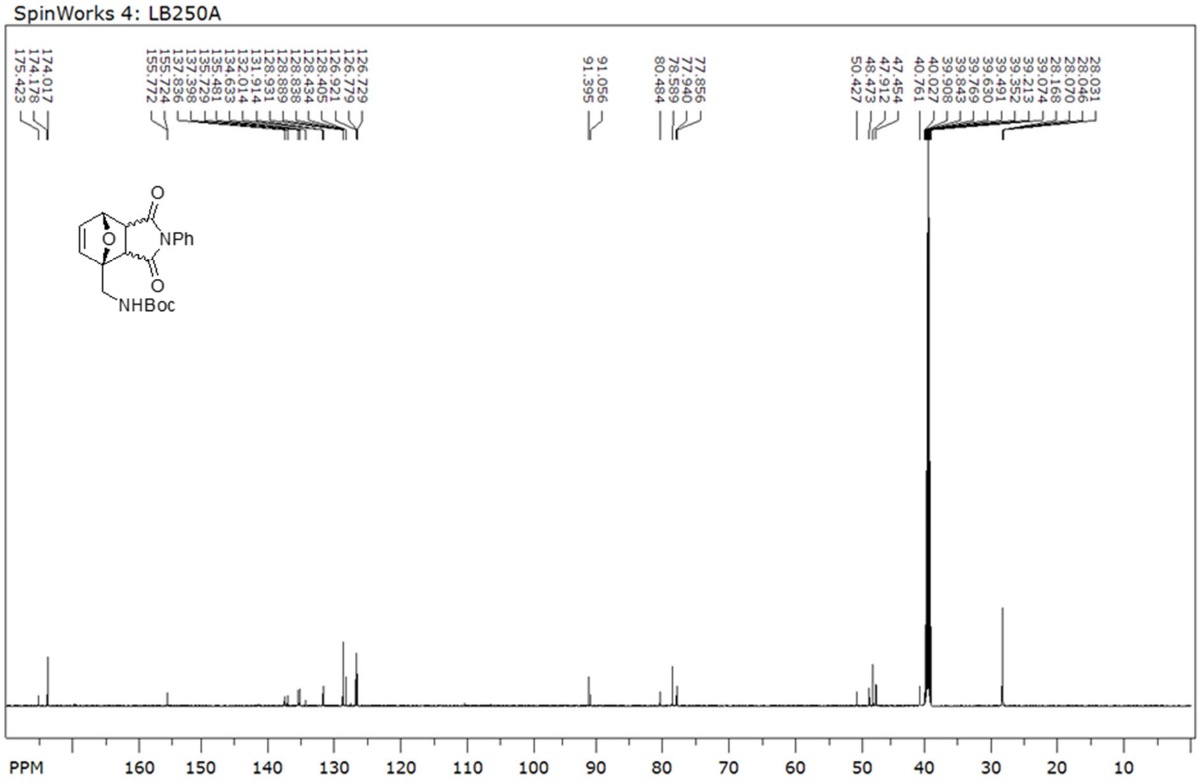

Figure S25. ^{13}C NMR (DMSO-d$_6$, 150.9 MHz) of **23**.

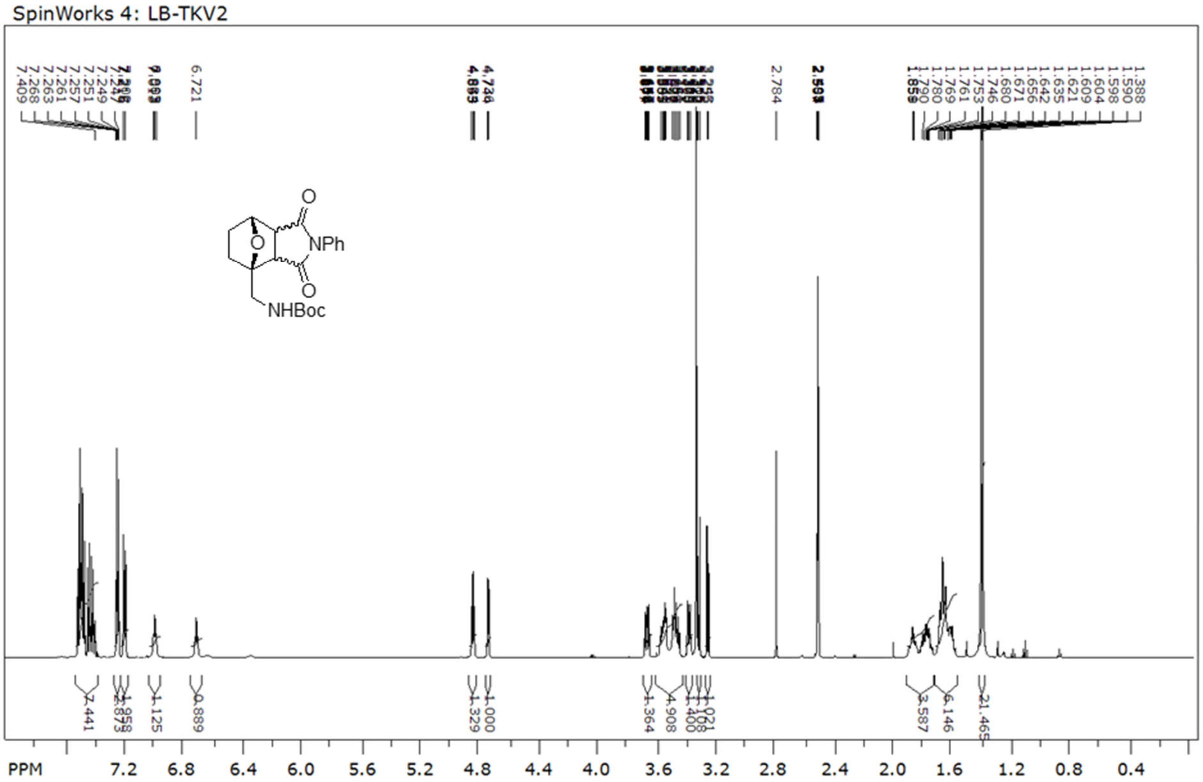

Figure S26. ^{1}H-NMR (DMSO-d$_6$, 600 MHz) of **27**.

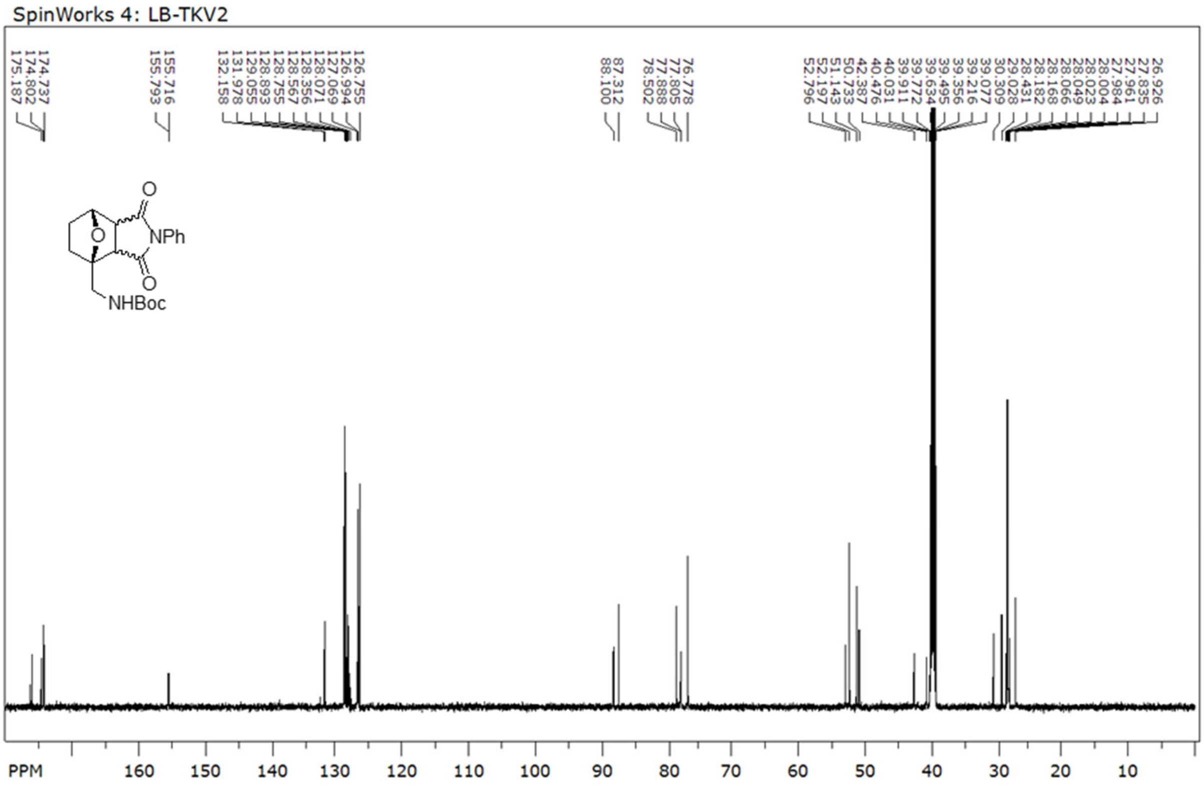

Figure S27. ^{13}C NMR (DMSO-d$_6$, 150.9 MHz) of **27**.

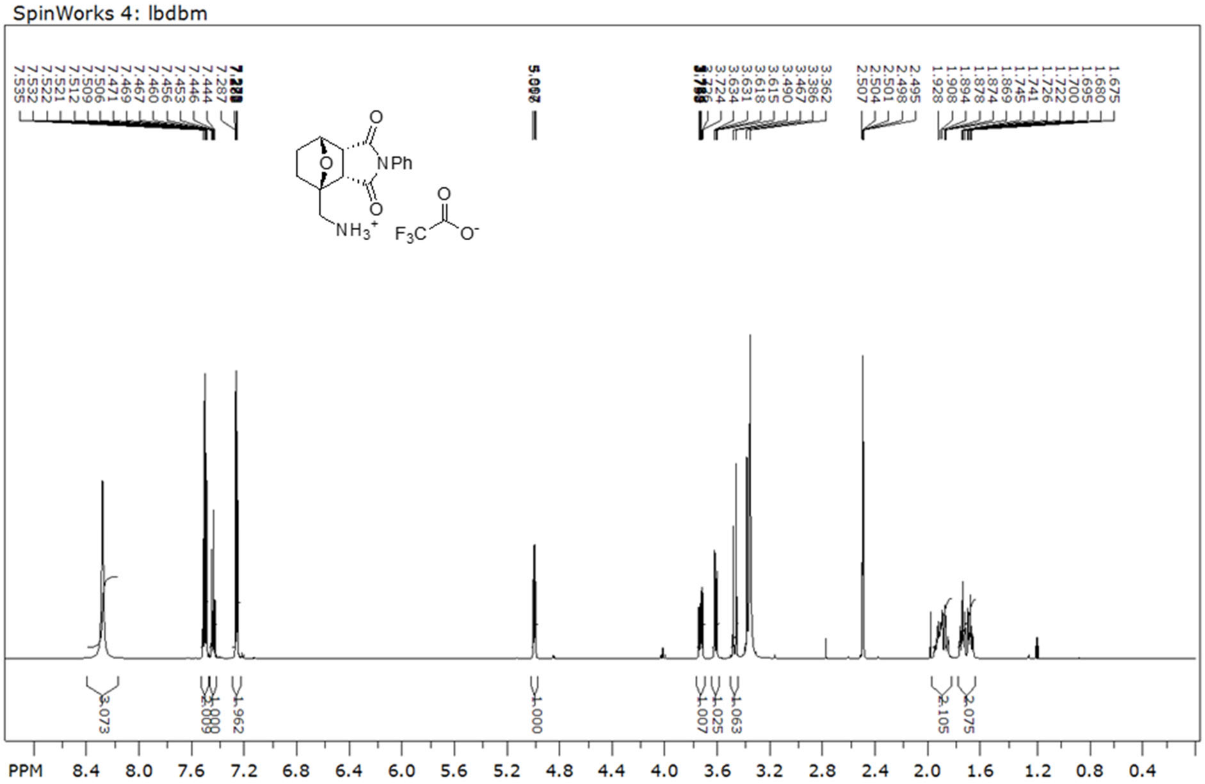

Figure S28. **1**H-NMR (DMSO-d$_6$, 600 MHz) of **28**.

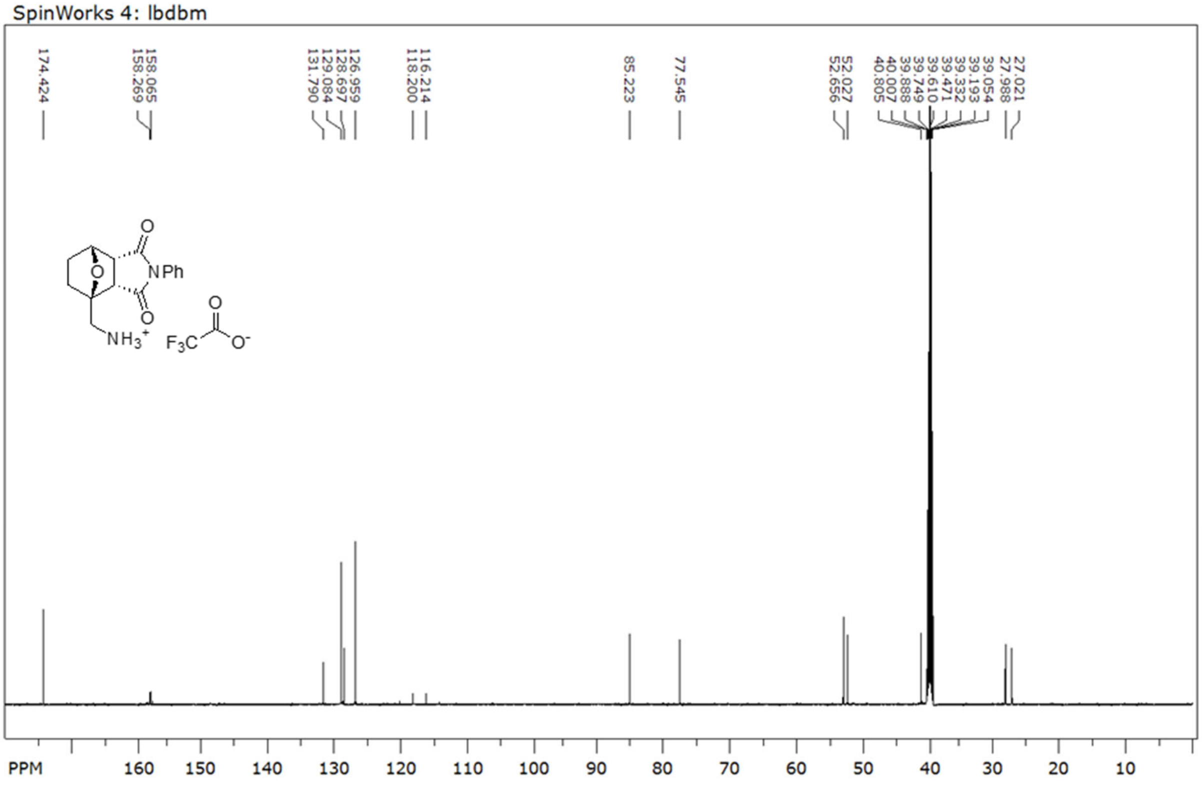

Figure S29. ^{13}C NMR (DMSO-d$_6$, 150.9 MHz) of **28**.

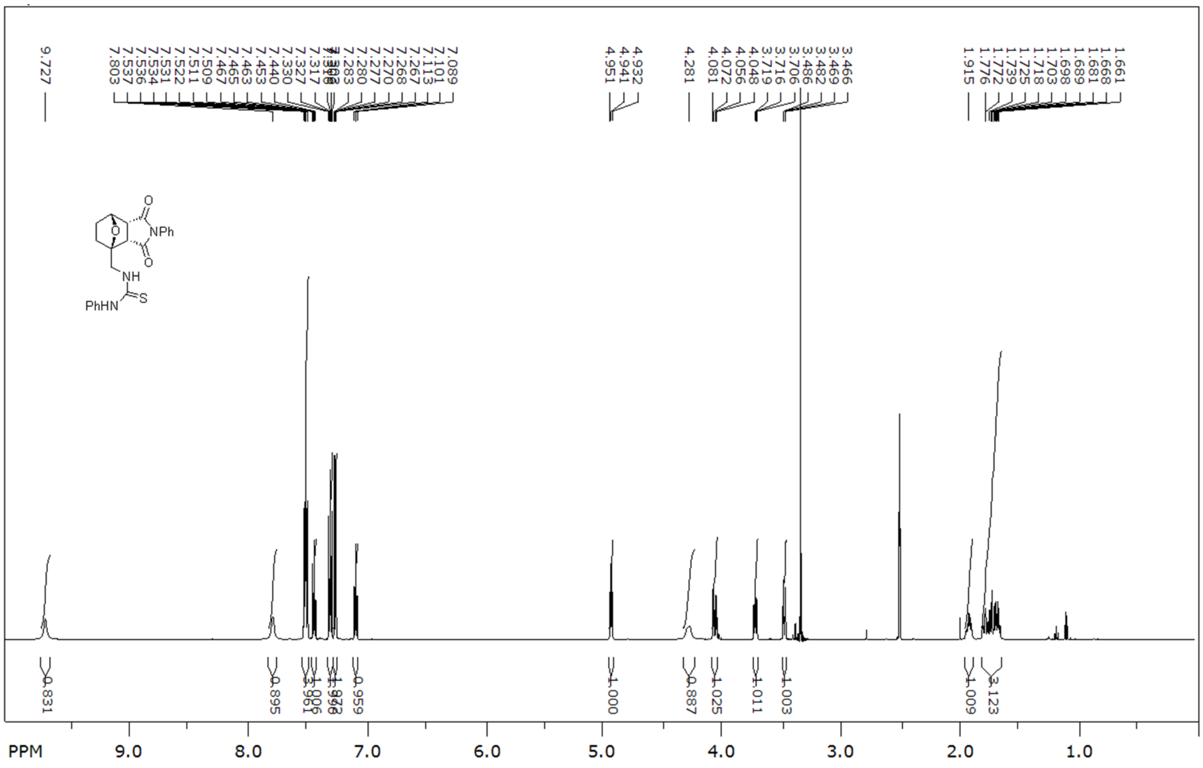

Figure S30. ^{1}H-NMR (DMSO-d_6, 600 MHz) spectrum of **10**

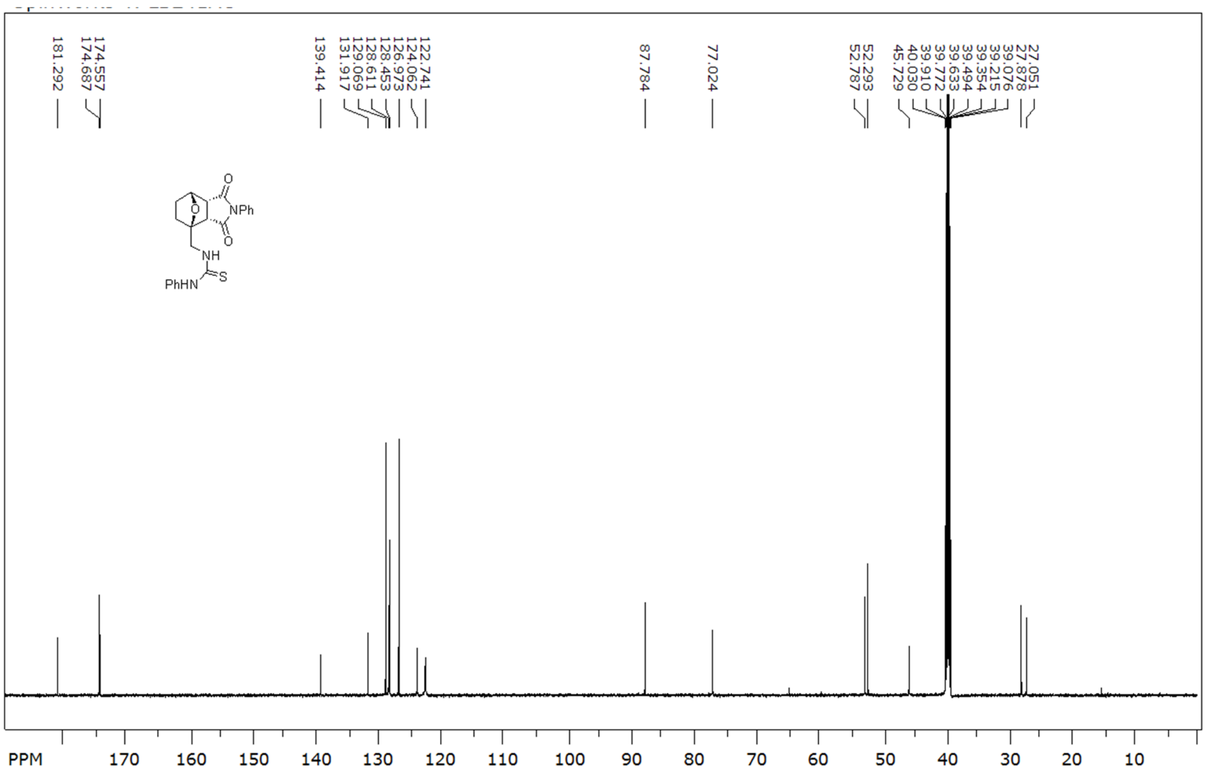

Figure S31. ^{13}C-NMR (DMSO-d_6, 150.9 MHz) spectrum of **10.**

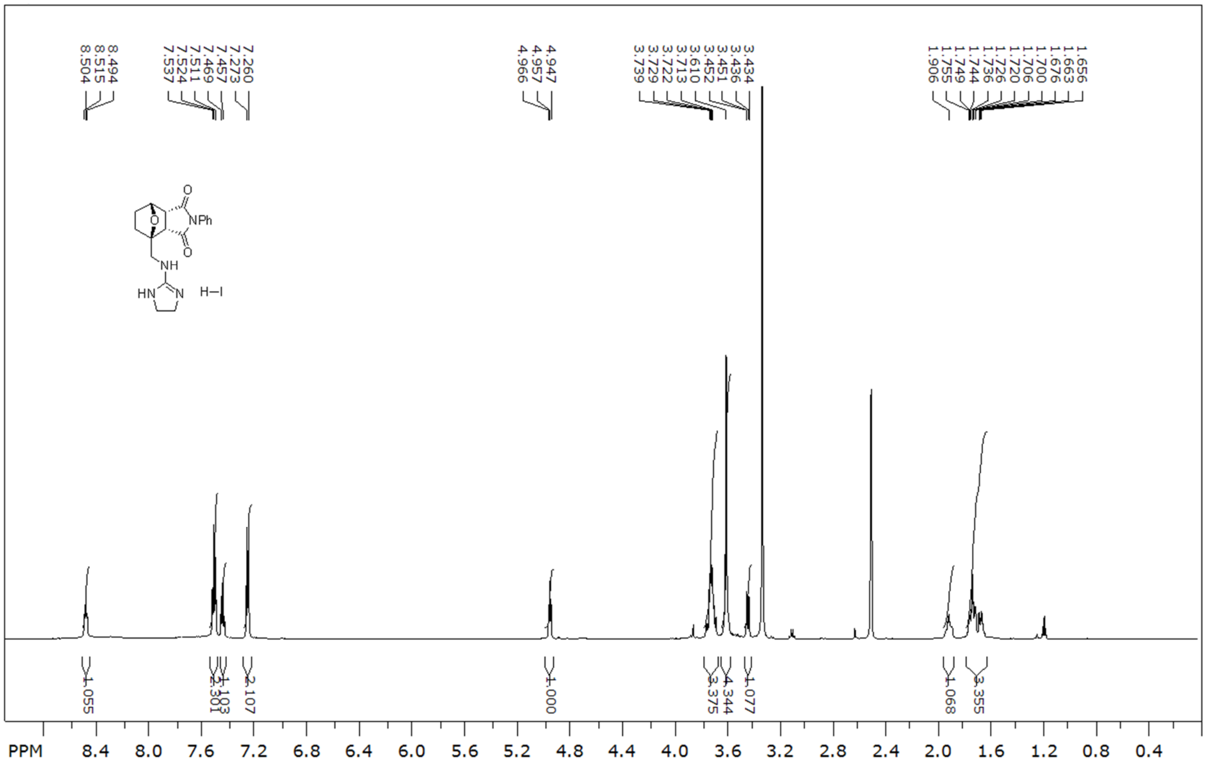

Figure S32. ^{1}H-NMR (DMSO-d_6, 600 MHz) spectrum of **11**.

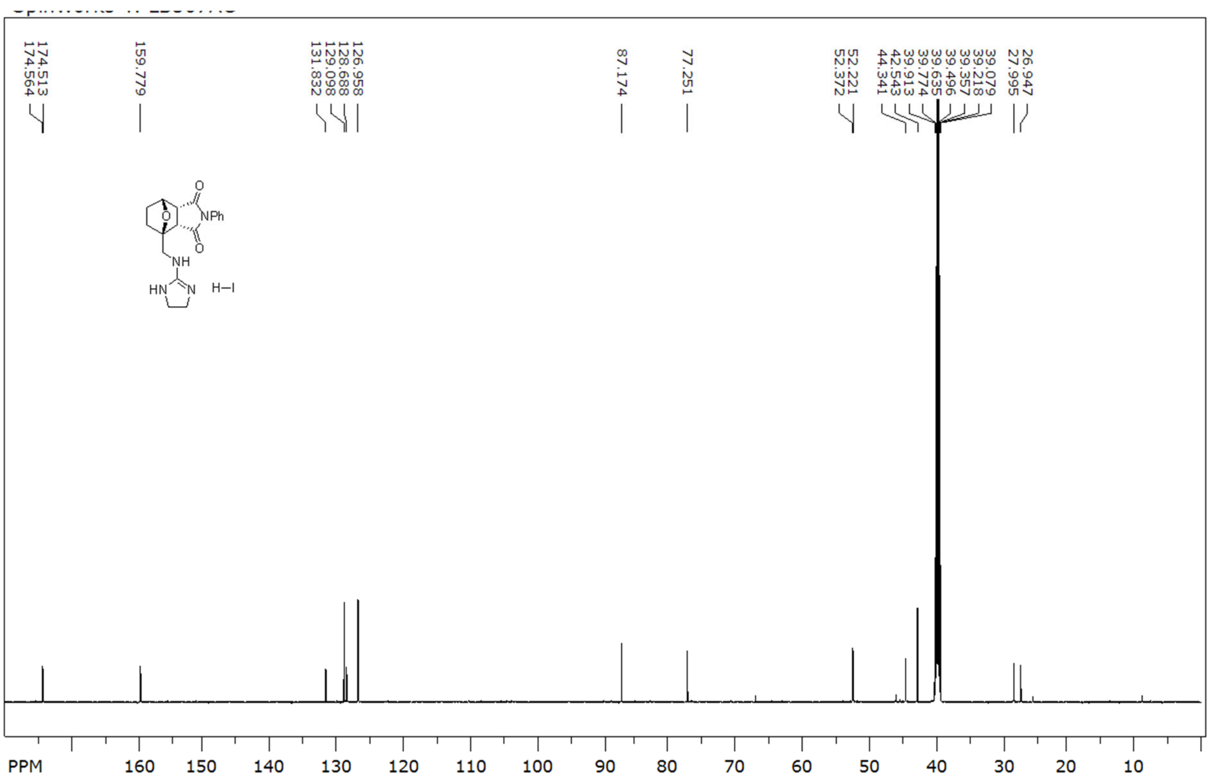

Figure S33. ^{13}C-NMR (DMSO-d_6, 150.9 MHz) spectrum of **11**.

S4. Diastereo- and enantioselectivities as determined by ^{1}H NMR and Chiral HPLC

2-((4-Chlorophenyl)(hydroxy)methyl)cyclohexanone (A1)

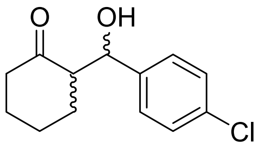

Chiralpak AD-3 (4.6 mm x 250 mm, 3 µm), *n*-hexane/2-propanol= 90:10, 220 nm, 1 ml/min

Table 2, Entry 4: Reaction conducted at r.t. (with **20n**, no (*S*)-proline): *racemate*

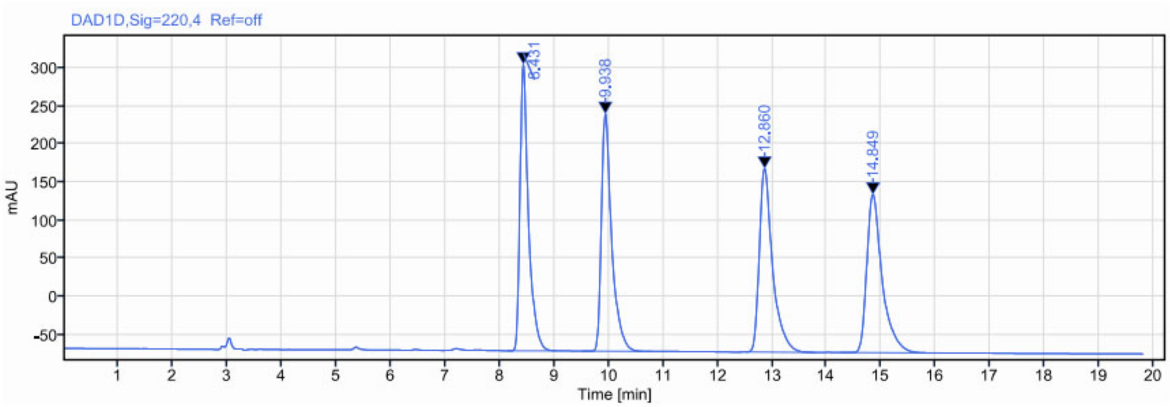

Signal: DAD1D,Sig=220,4 Ref=off

RT [min]	Type	Width [min]	Area	Height	Area%	Name
8.431	MM m	0.88	4074.74	375.21	25.04	
9.938	MM m	1.27	4079.20	310.78	25.07	
12.860	MM m	1.29	4066.74	240.21	24.99	
14.849	MM m	2.02	4051.78	206.94	24.90	
		**Sum**	**16272.46**			

Table 2, Entry 1: Reaction conducted at r.t.: *e.e.* (*syn*) = 11%, *e.e.* (*anti*) = 80%

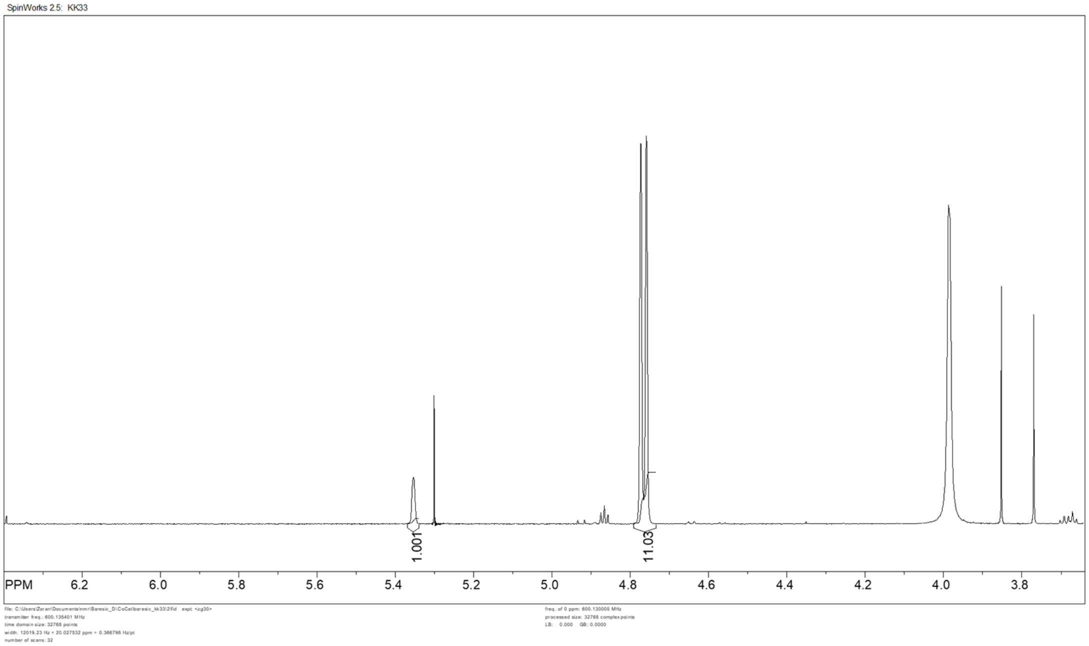

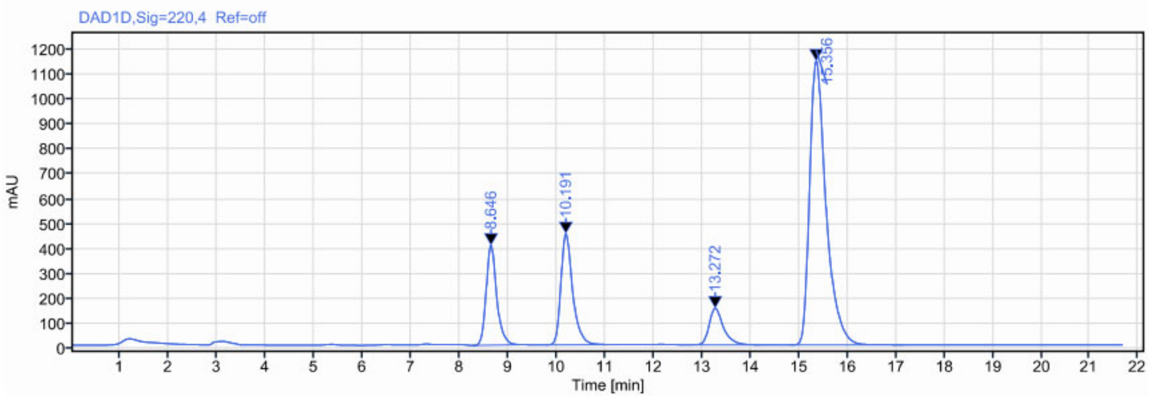

Signal: DAD1D,Sig=220,4 Ref=off

RT [min]	Type	Width [min]	Area	Height	Area%	Name
8.646	MM m	1.08	6075.19	400.76	13.99	
10.191	MM m	1.50	7520.10	445.18	17.32	
13.272	MM m	1.41	2974.42	146.51	6.85	
15.356	MM m	2.36	26848.83	1138.49	61.84	
		**Sum**	**43418.53**			

Table 2, Entry 1: Reaction conducted at 10 °C: *e.e.* (*syn*) = - 26%, *e.e.* (*anti*) = 95%

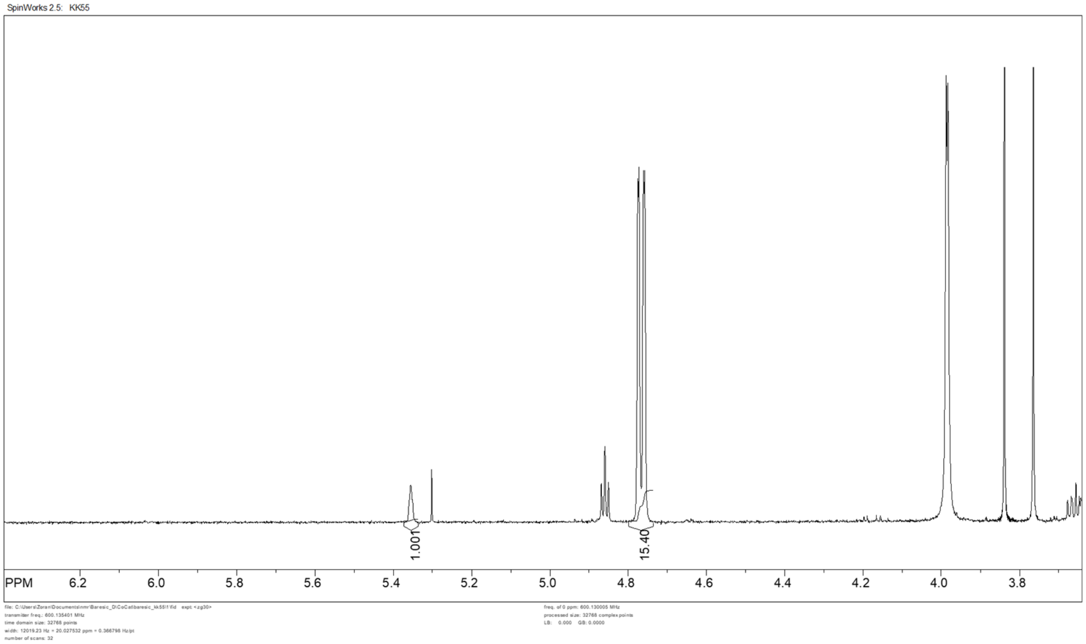

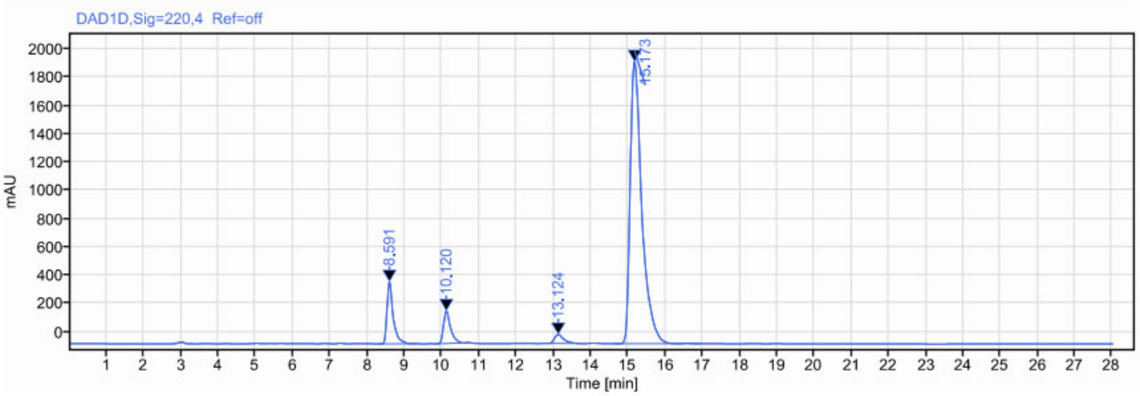

Signal: DAD1D,Sig=220,4 Ref=off

RT [min]	Type	Width [min]	Area	Height	Area%	Name
8.591	MM m	1.00	5048.60	437.72	9.60	
10.120	MM m	0.88	2962.92	228.79	5.63	
13.124	MM m	0.82	1067.69	63.03	2.03	
15.173	MM m	2.33	43526.27	1992.78	82.74	
		**Sum**	**52605.48**			

Table 2, Entry 2: Reaction conducted at 0 °C: *e.e.* (*syn*) = - 26%, *e.e.* (*anti*) = 99%

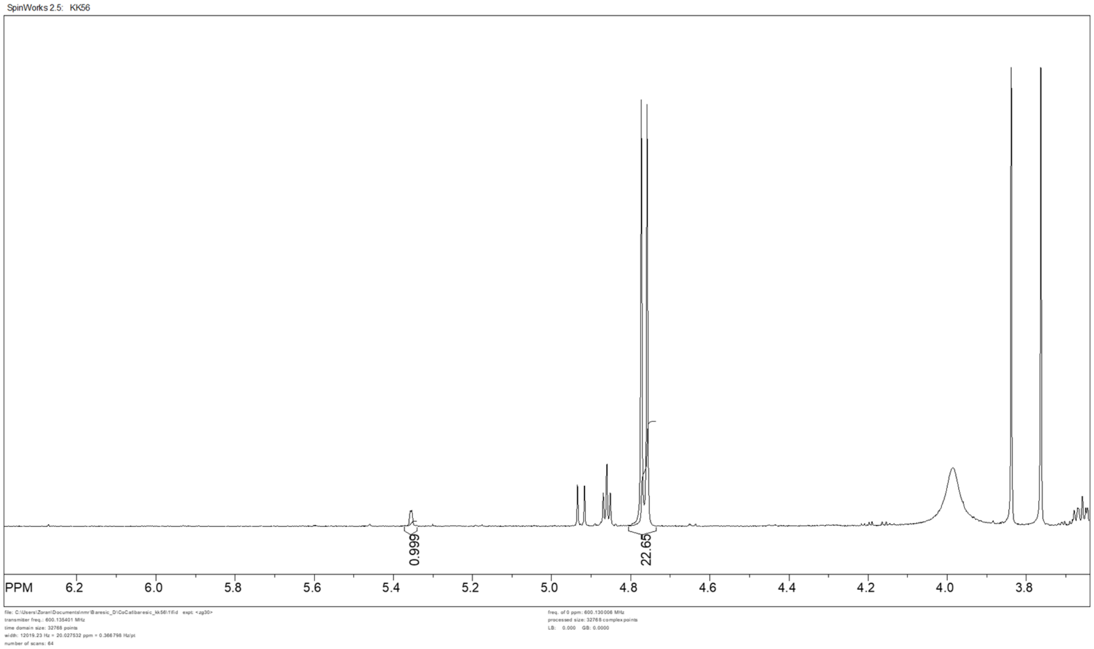

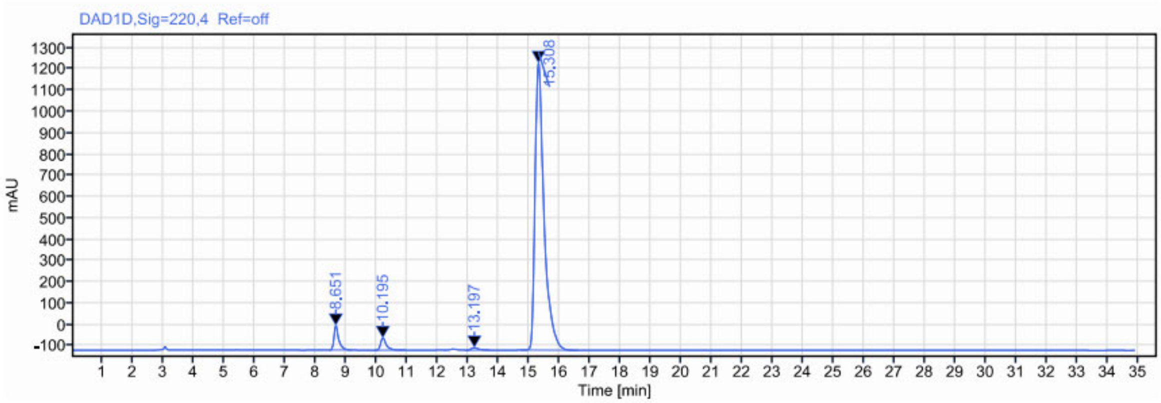

Signal: DAD1D,Sig=220,4 Ref=off

RT [min]	Type	Width [min]	Area	Height	Area%	Name
8.651	MM m	0.74	1248.23	114.89	4.17	
10.195	MM m	0.99	738.94	56.80	2.47	
13.197	MM m	0.76	181.60	11.43	0.61	
15.308	MM m	1.89	27778.42	1349.44	92.76	
		**Sum**	**29947.20**			

Table 2, Entry 3: Reaction conducted at r.t. (in MeOH): *e.e.* (*syn*) = 9%, *e.e.* (*anti*) = 33%

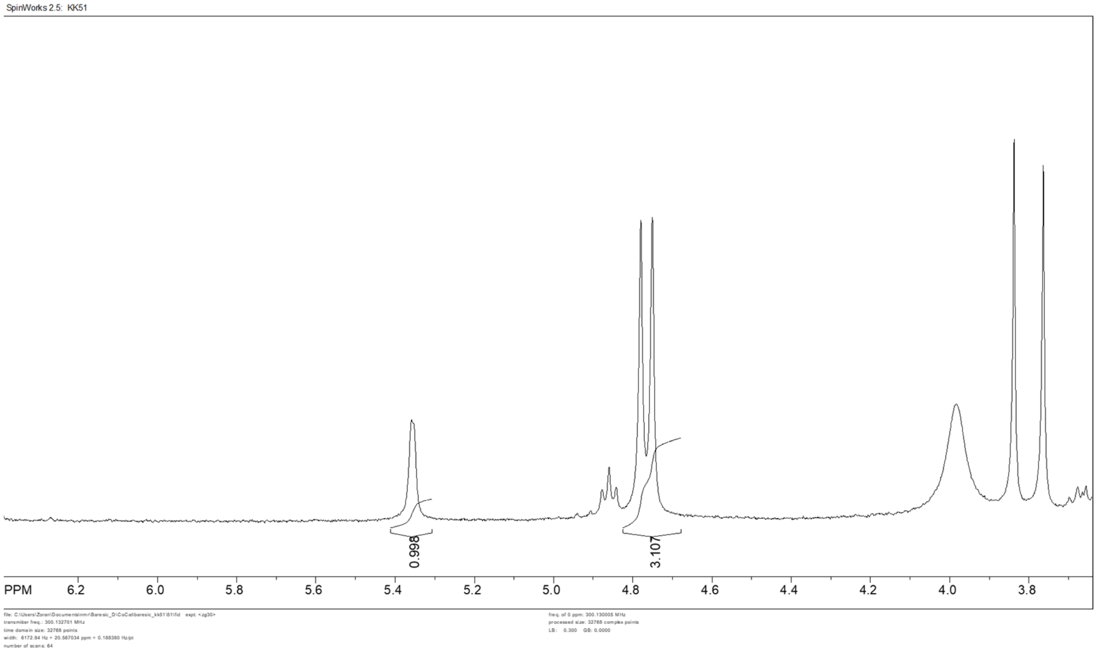

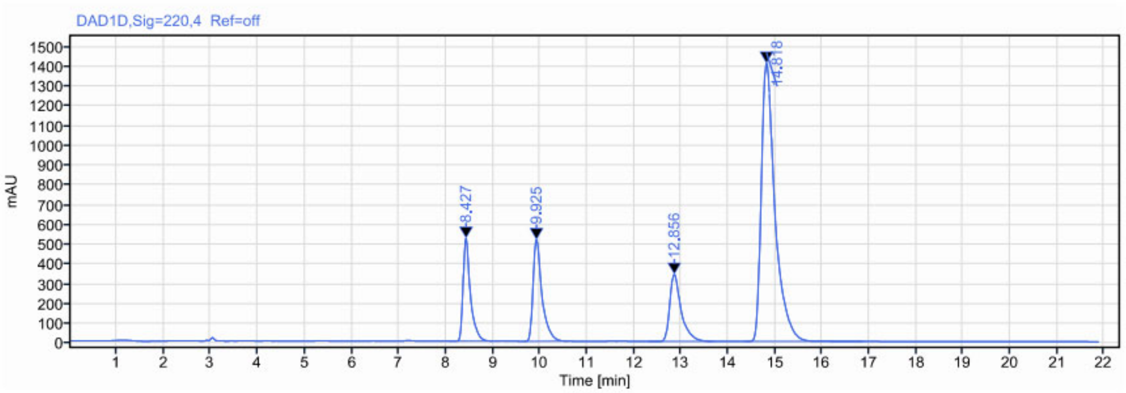

Signal: DAD1D,Sig=220,4 Ref=off

RT [min]	Type	Width [min]	Area	Height	Area%	Name
8.427	MM m	0.93	5620.65	519.68	12.08	
9.925	MM m	0.94	6745.15	512.54	14.50	
12.856	MM m	1.37	5712.51	338.38	12.28	
14.818	MM m	2.51	28436.20	1406.26	61.13	
		**Sum**	**46514.51**			

Table 2, Entry 5: Reaction conducted at 0 °C. with **4**[pf] as cocatalyst: *e.e.* (*syn*) = -15%, *e.e.* (*anti*) = 99.5%

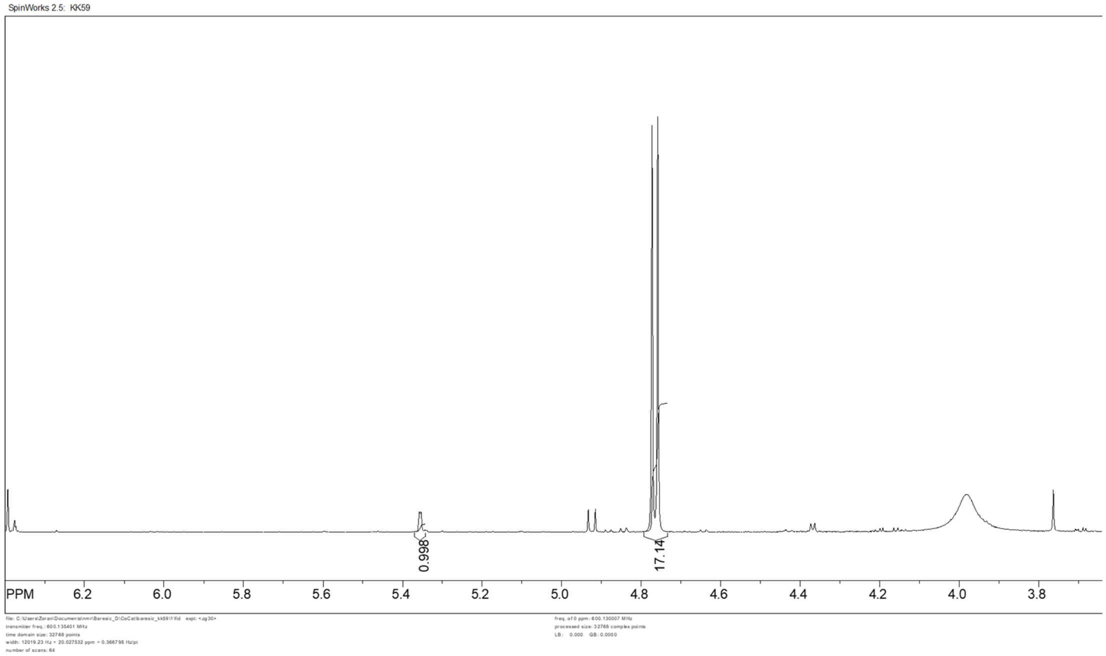

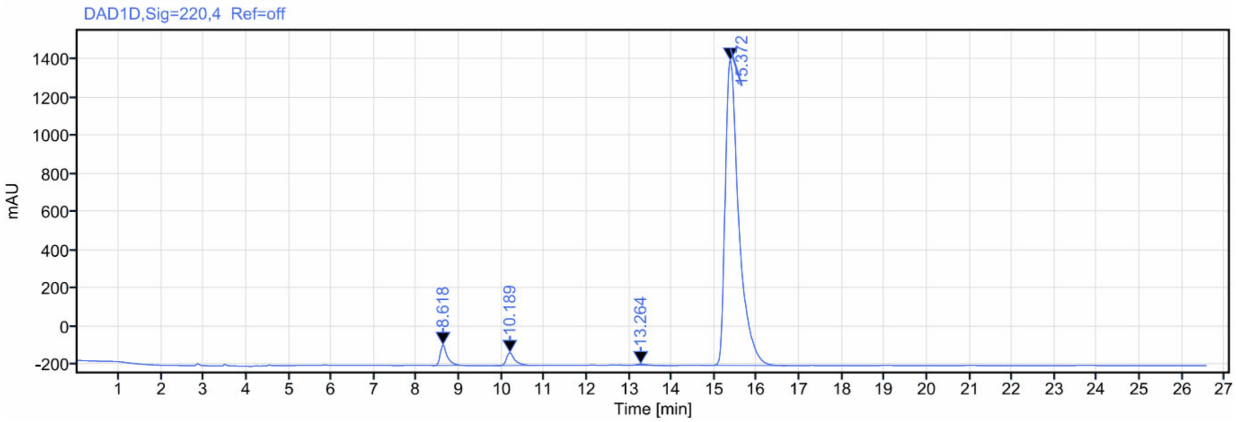

Signal: DAD1D,Sig=220,4 Ref=off

RT [min]	Type	Width [min]	Area	Height	Area%	Name
8.618	MM m	0.71	1216.51	107.23	3.22	
10.189	MM m	1.00	901.41	66.51	2.39	
13.264	MM m	1.03	89.26	6.73	0.24	
15.372	MM m	2.00	35557.98	1598.58	94.16	
		**Sum**	**37765.16**			

2-((4-Bromophenyl)(hydroxy)methyl)cyclohexanone (A2)

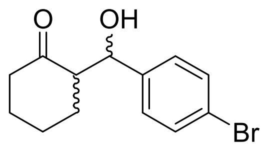

Chiralpak AD-3 (4.6 mm x 250 mm, 3 µm), *n*-hexane/2-propanol= 90:10, 220 nm, 1 ml/min

racemate

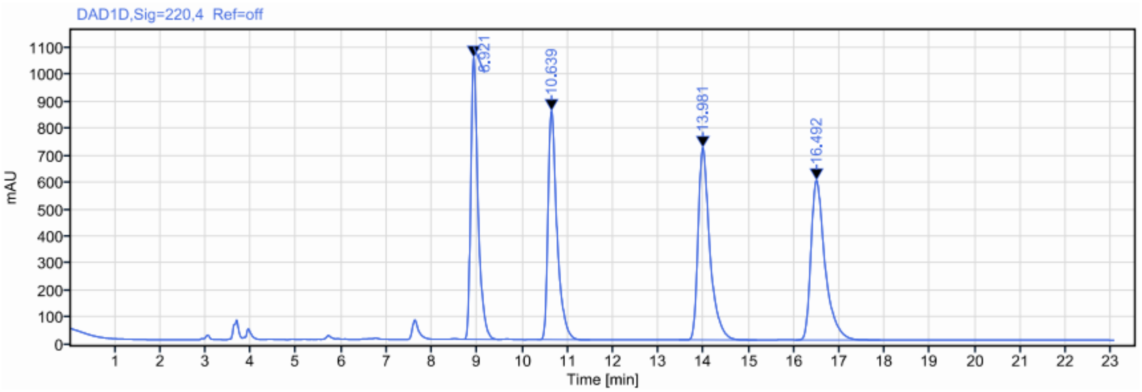

Signal: DAD1D,Sig=220,4 Ref=off

RT [min]	Type	Width [min]	Area	Height	Area%	Name
8.921	MM m	0.84	11617.02	1043.71	23.79	
10.639	MM m	1.16	11644.23	846.82	23.85	
13.981	MM m	1.72	12793.06	711.43	26.20	
16.492	MM m	1.87	12776.45	591.20	26.16	
		**Sum**	**48830.76**			

Table 2, Entry 6: Reaction conducted at r.t.: *e.e.* (*syn*) = 17%, *e.e.* (*anti*) = 95%

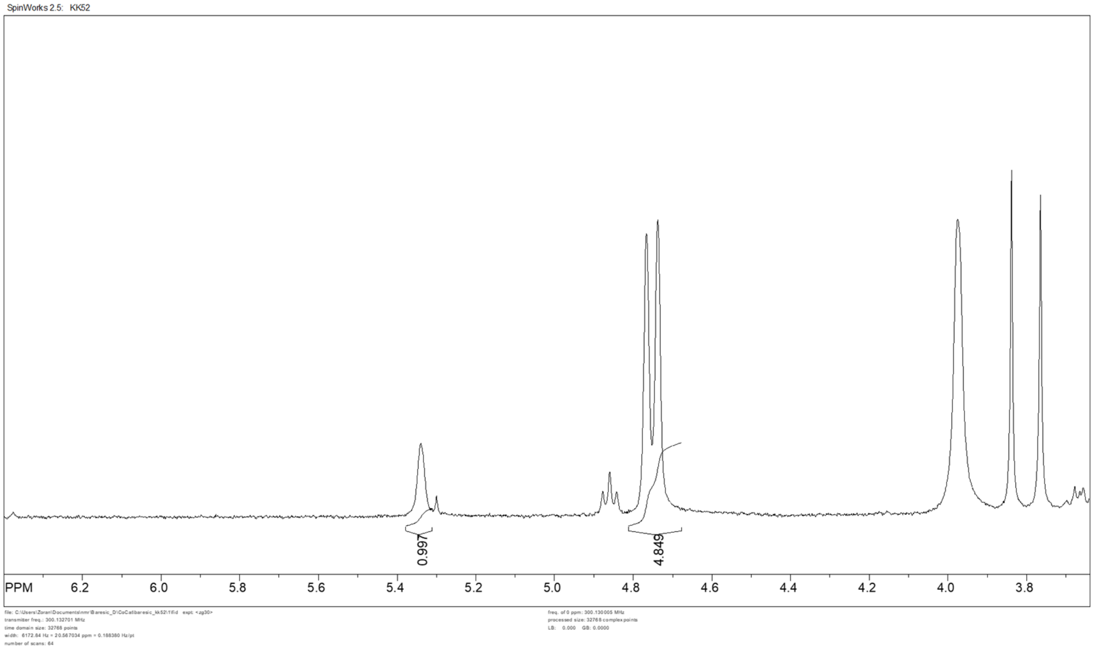

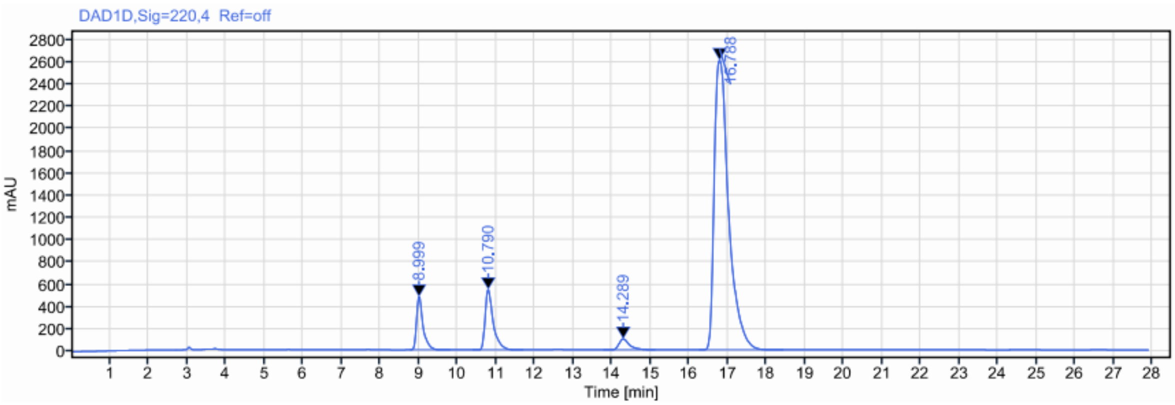

Signal: DAD1D,Sig=220,4 Ref=off

RT [min]	Type	Width [min]	Area	Height	Area%	Name
8.999	MM m	1.04	5539.74	475.04	6.71	
10.790	MM m	1.30	7825.97	540.96	9.48	
14.289	MM m	1.34	1839.13	98.34	2.23	
16.788	MM m	2.18	67325.39	2602.68	81.58	
		**Sum**	**82530.23**			

2-((4-Ethylphenyl)(hydroxy)methyl)cyclohexanone (A3)

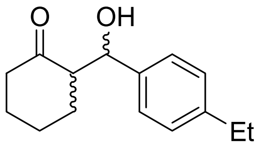

Chiralcel OD-3 (4.6 mm x 250 mm, 3 µm), *n*-hexane/2-propanol= 95:5, 220 nm, 1 ml/min

racemate:

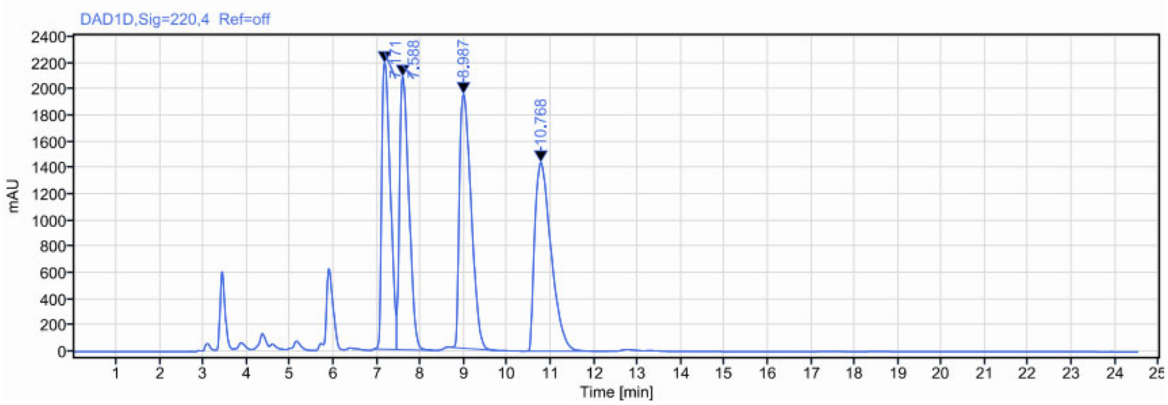

Signal: DAD1D,Sig=220,4 Ref=off

RT [min]	Type	Width [min]	Area	Height	Area%	Name
7.171	MM m	0.51	29908.00	2177.22	22.11	
7.588	MM m	0.83	31321.37	2077.37	23.15	
8.987	MM m	1.13	36420.65	1934.29	26.92	
10.768	MM m	1.97	37633.00	1436.19	27.82	
		**Sum**	**135283.03**			

Table 2, Entry 7: Reaction conducted at r.t.: *e.e.* (*syn*) = 51%, *e.e.* (*anti*) = 84%

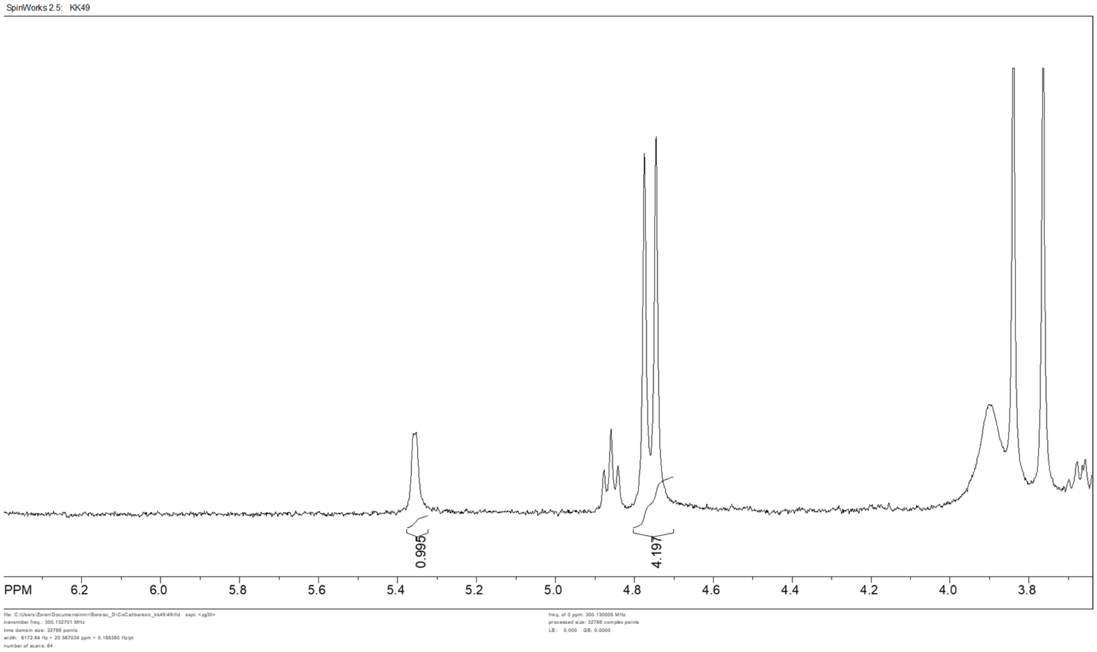

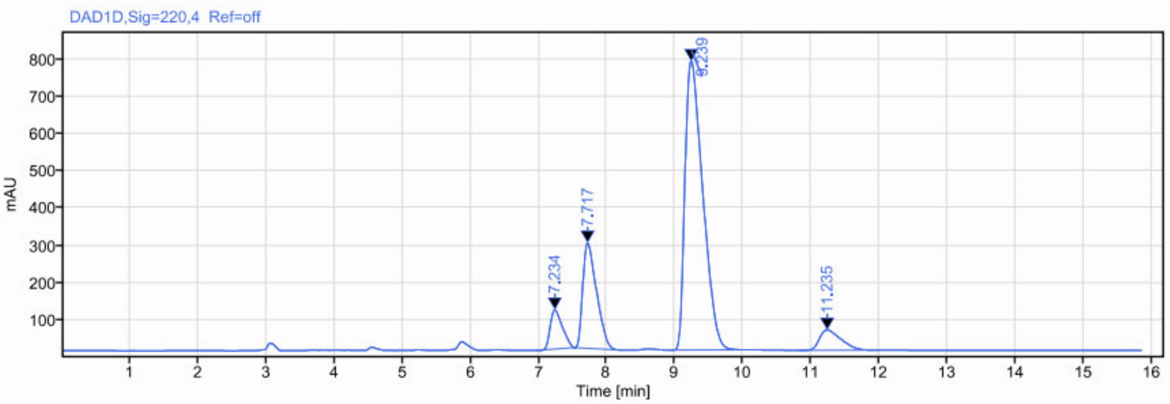

Signal: DAD1D,Sig=220,4 Ref=off

RT [min]	Type	Width [min]	Area	Height	Area%	Name
7.234	MM m	0.56	1295.14	105.37	6.28	
7.717	MM m	0.69	3991.18	283.88	19.34	
9.239	MM m	1.15	14163.28	777.74	68.62	
11.235	MM m	1.17	1190.04	54.79	5.77	
		**Sum**	**20639.65**			

2-((Hydroxy)(2-nitrophenyl)methyl)cyclohexanone (A4)

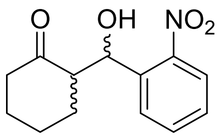

Chiralpak AD-3 (4.6 mm x 250 mm, 3 µm), *n*-hexane/2-propanol= 90:10, 220 nm, 1 ml/min

Racemate

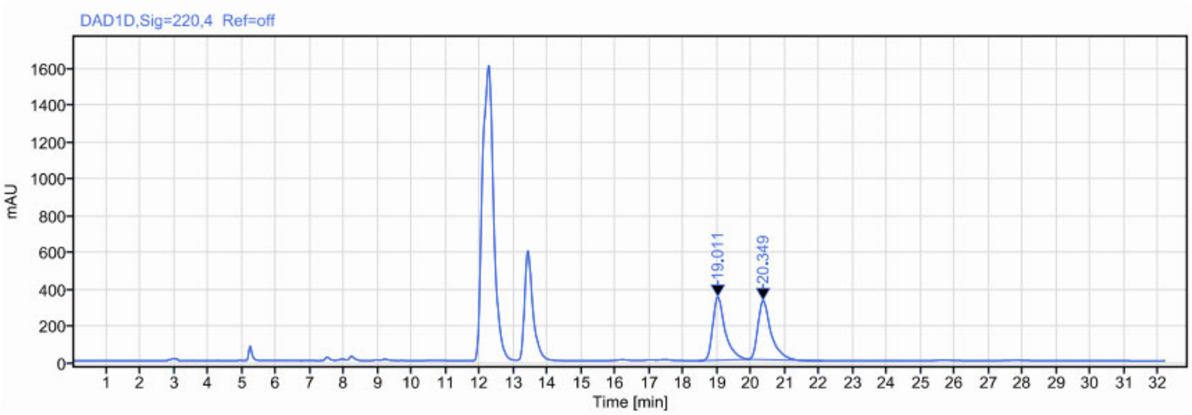

Signal: DAD1D,Sig=220,4 Ref=off

RT [min]	Type	Width [min]	Area	Height	Area%	Name
19.011	MM m	1.47	8675.23	342.28	50.21	
20.349	MM m	2.22	8600.98	320.37	49.79	
		**Sum**	**17276.21**			

Table 2, Entry 8: Reaction conducted at r.t.: *e.e.* (*anti*) = 98%

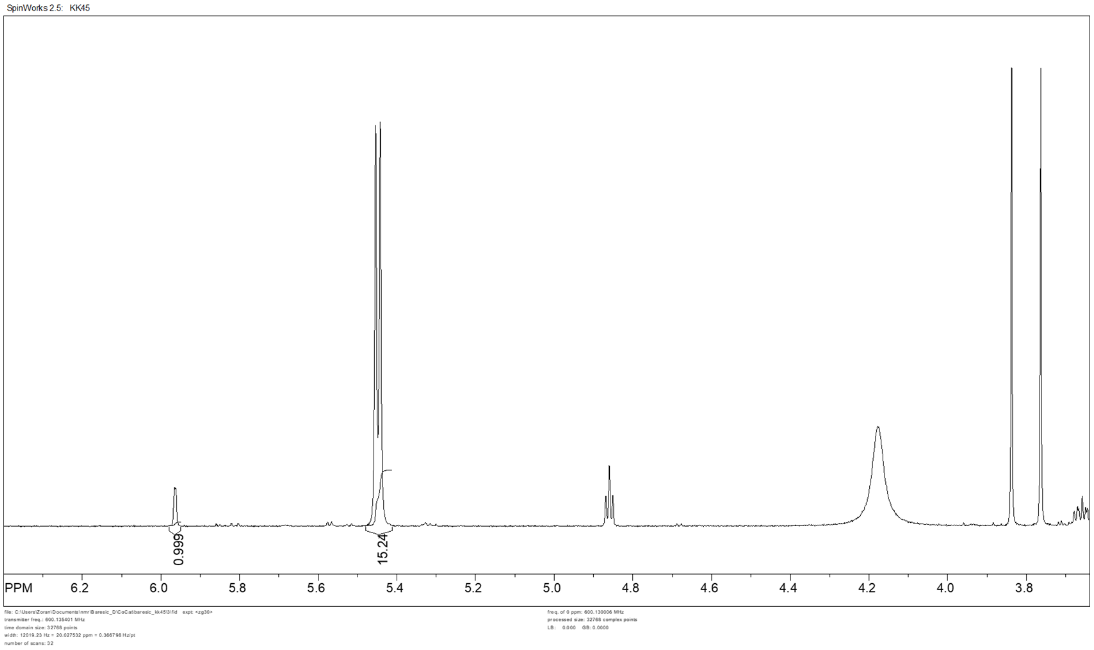

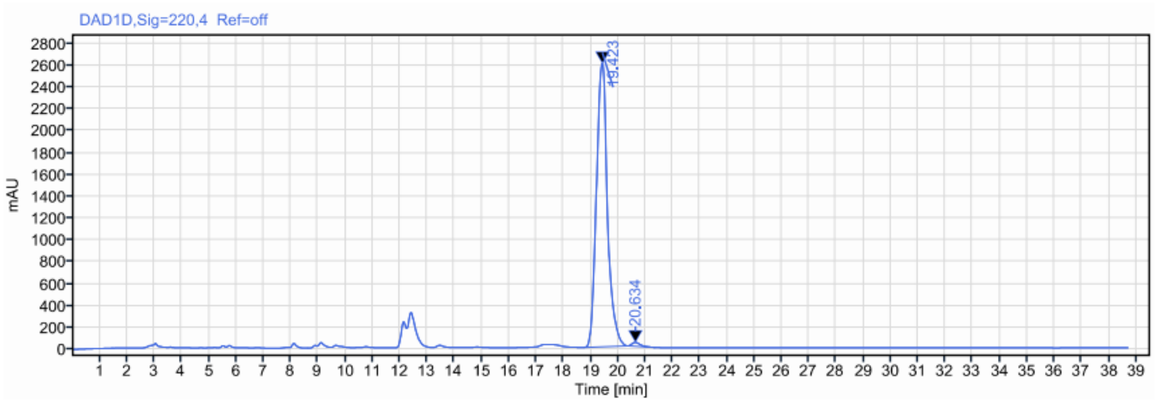

Signal:	DAD1D,Sig=220,4 Ref=off					
RT [min]	Type	Width [min]	Area	Height	Area%	Name
19.423	MM m	1.60	73039.02	2593.50	98.99	
20.634	MM m	1.00	747.06	35.90	1.01	
		Sum	73786.08			

Table 2, Entry 9: Reaction conducted at 0°C: *e.e.* (*anti*) >99.5%.

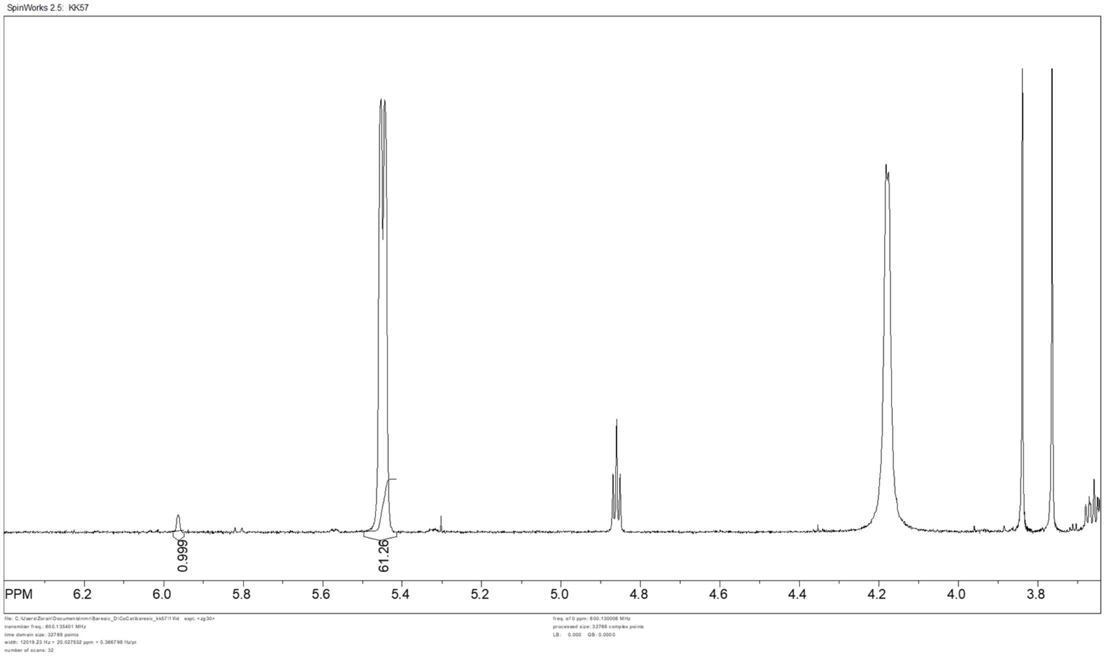

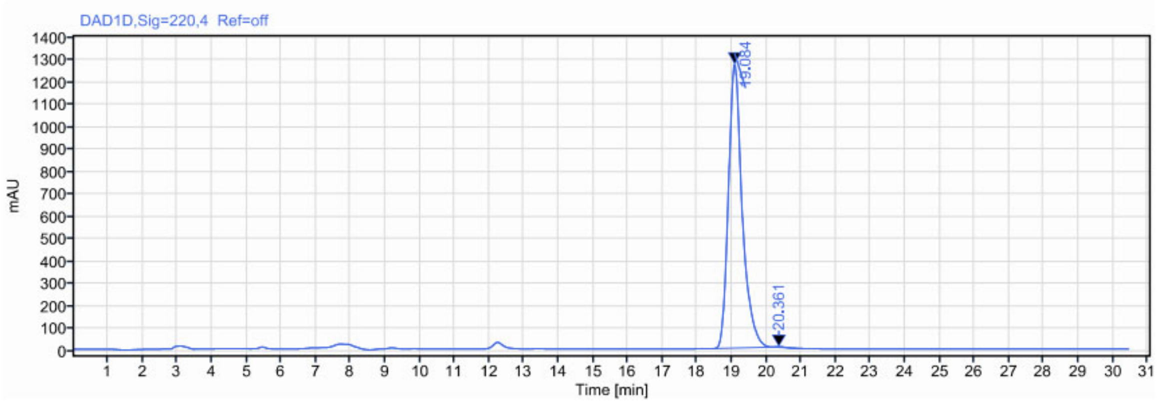

Signal: DAD1D,Sig=220,4 Ref=off

RT [min]	Type	Width [min]	Area	Height	Area%	Name
19.084	MM m	1.74	35177.12	1266.10	99.81	
20.361	MM m	0.77	65.78	3.54	0.19	
		**Sum**	**35242.90**			

2-((Hydroxy)(3-nitrophenyl)methyl)cyclohexanone (A5)

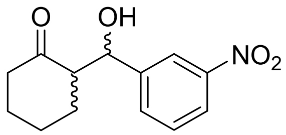

Chiralpak AD-3 (4.6 mm x 250 mm, 3 µm), *n*-hexane/2-propanol= 90:10, 220 nm, 1 ml/min

Racemate

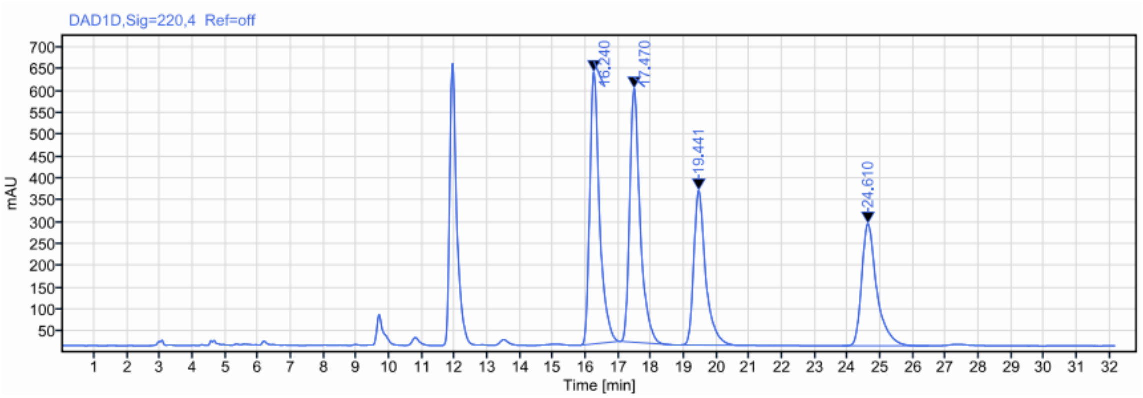

Signal: DAD1D,Sig=220,4 Ref=off

RT [min]	Type	Width [min]	Area	Height	Area%	Name
16.240	MM m	1.20	12880.42	622.52	29.53	
17.470	MM m	1.71	12879.37	580.95	29.53	
19.441	MM m	2.28	8884.07	354.28	20.37	
24.610	MM m	2.63	8967.38	279.73	20.56	
		**Sum**	**43611.24**			

Table 2, Entry 10: Reaction conducted at r.t.: *e.e.* (*syn*) = 16%, *e.e.* (*anti*) = 89%.

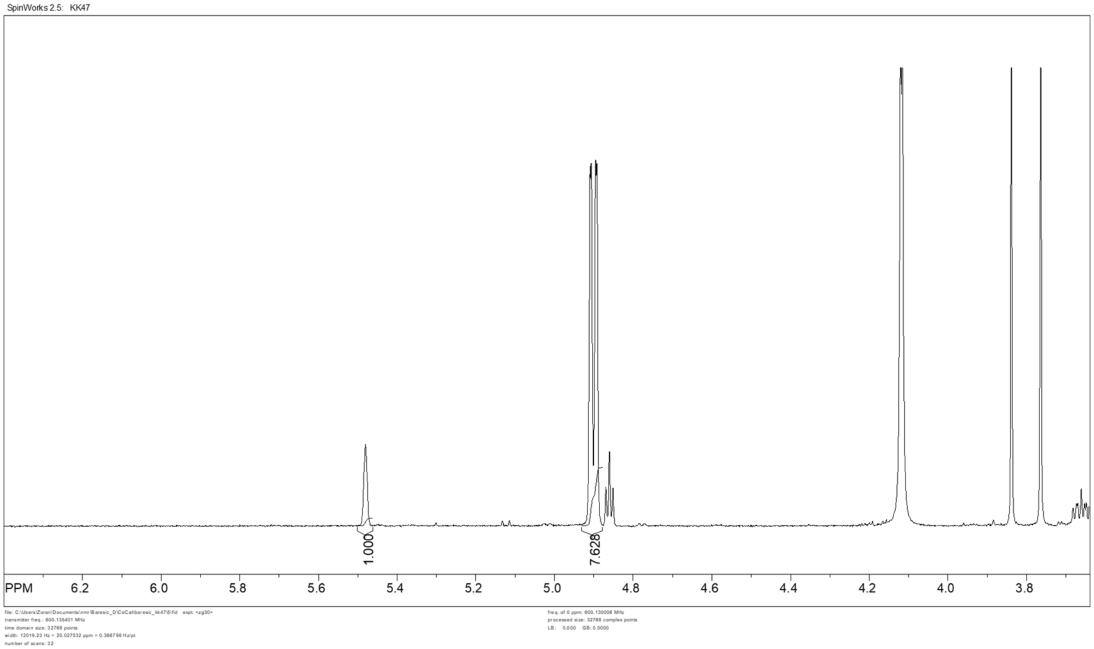

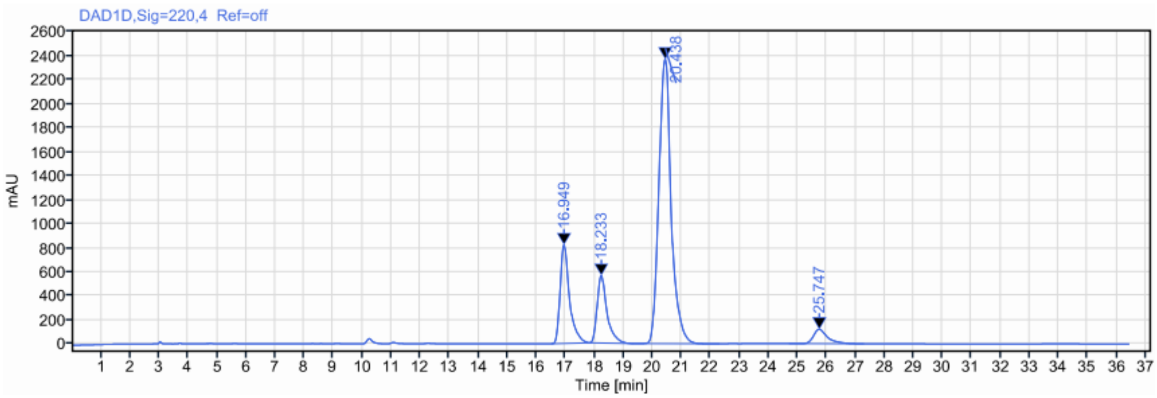

Signal: DAD1D,Sig=220,4 Ref=off

RT [min]	Type	Width [min]	Area	Height	Area%	Name
16.949	MM m	1.30	18305.10	819.41	17.35	
18.233	MM m	1.52	13279.44	560.29	12.59	
20.438	MM m	2.98	69854.53	2366.73	66.21	
25.747	MM m	2.58	4069.83	119.86	3.86	
		**Sum**	**105508.90**			

2-((Hydroxy)(4-nitrophenyl)methyl)cyclohexanone (A6)

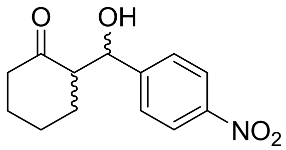

Chiralpak AD-3 (4.6 mm x 250 mm, 3 µm), *n*-hexane/2-propanol = 90:10, 220 nm, 1 ml/min

Racemate

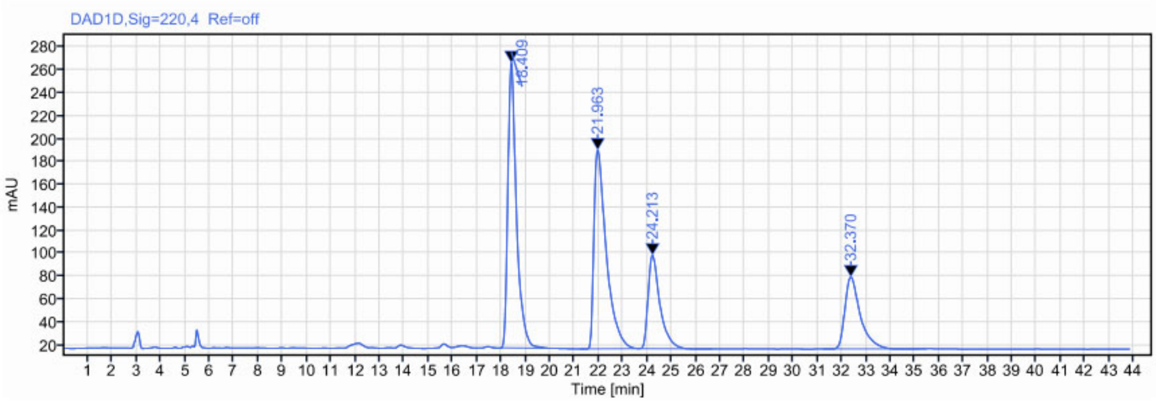

Signal: DAD1D,Sig=220,4 Ref=off

RT [min]	Type	Width [min]	Area	Height	Area%	Name
18.409	MM m	2.46	6128.00	248.91	34.51	
21.963	MM m	2.40	6131.90	173.28	34.54	
24.213	MM m	3.04	2762.96	81.45	15.56	
32.370	MM m	4.19	2732.10	62.64	15.39	
		**Sum**	**17754.96**			

Table 2, Entry 11: Reaction conducted at r.t.: *e.e.* (*syn*) = 38%, *e.e.* (*anti*) = 92%

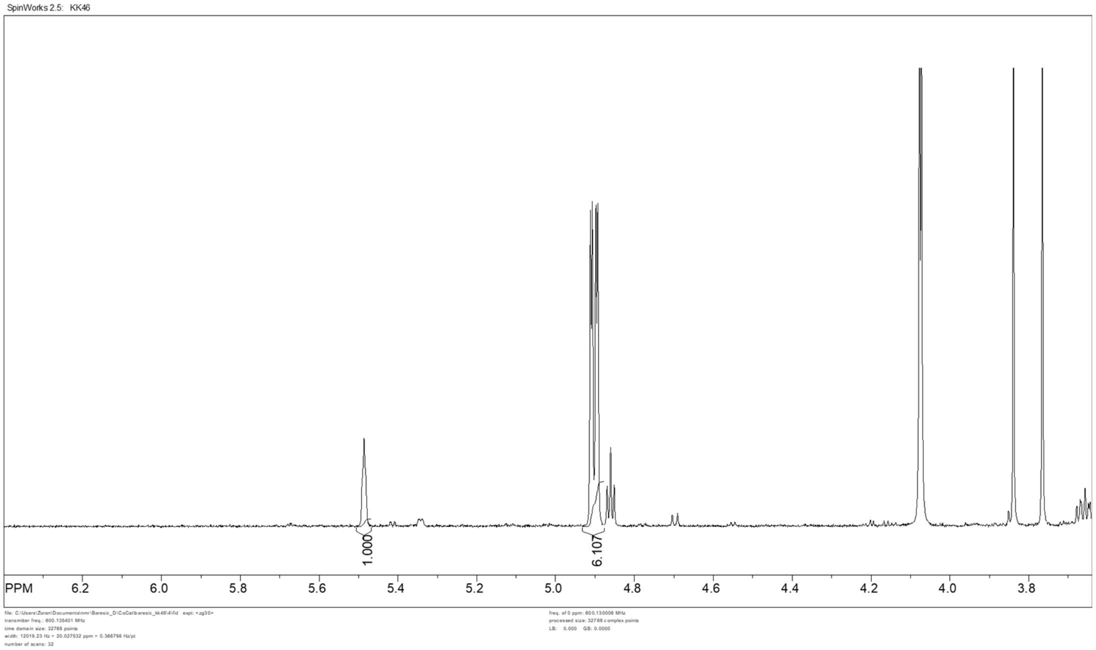

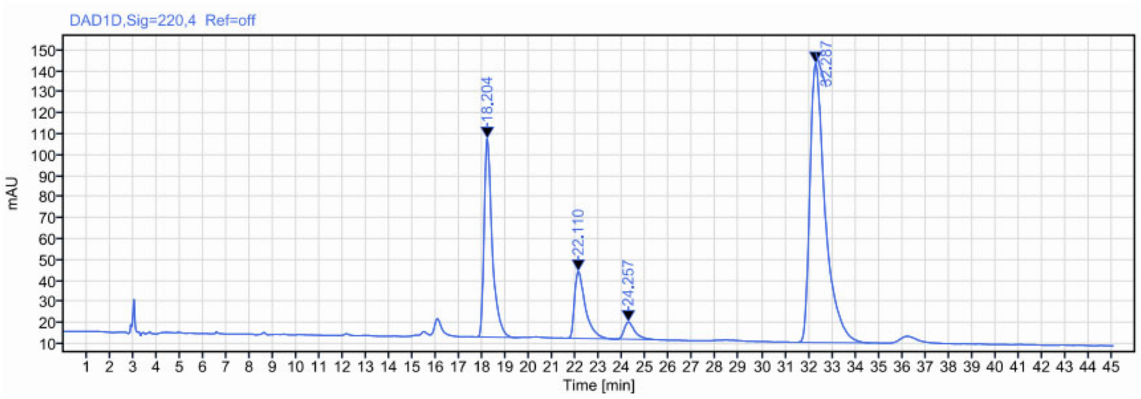

Signal: DAD1D,Sig=220,4 Ref=off

RT [min]	Type	Width [min]	Area	Height	Area%	Name
18.204	MM m	2.01	2278.34	94.47	23.66	
22.110	MM m	2.17	1031.00	31.89	10.71	
24.257	MM m	2.01	258.81	8.18	2.69	
32.287	MM m	3.26	6059.92	132.98	62.94	
		**Sum**	**9628.07**			

Table 2, Entry 12: Reaction conducted at 0°C, *e.e.* (*syn*) = 41%, *e.e.* (*anti*) = 95%.

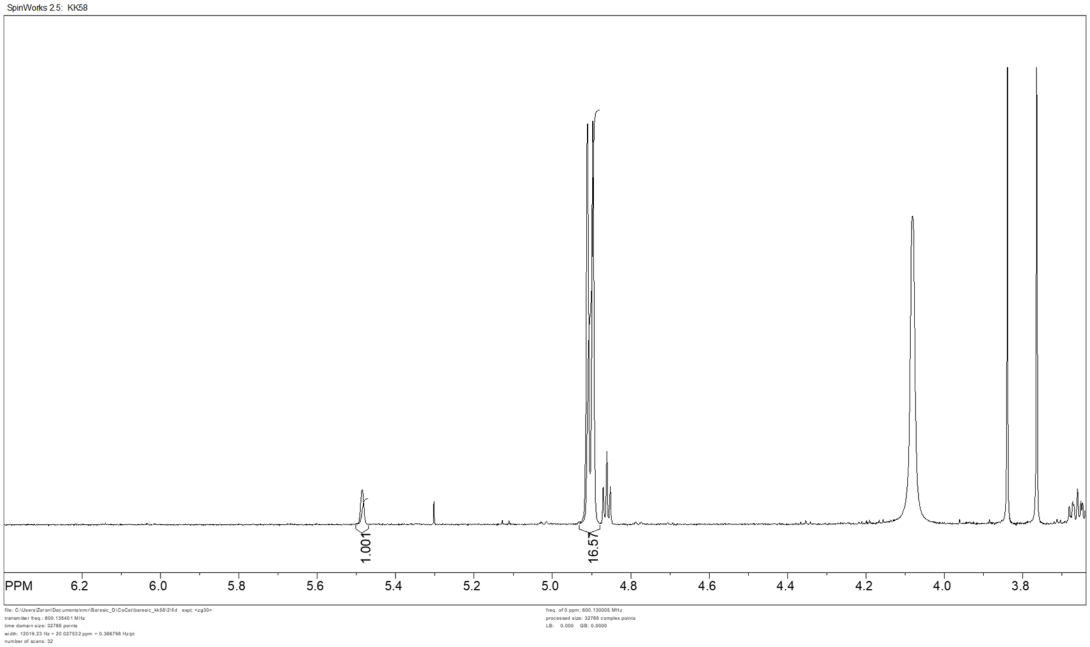

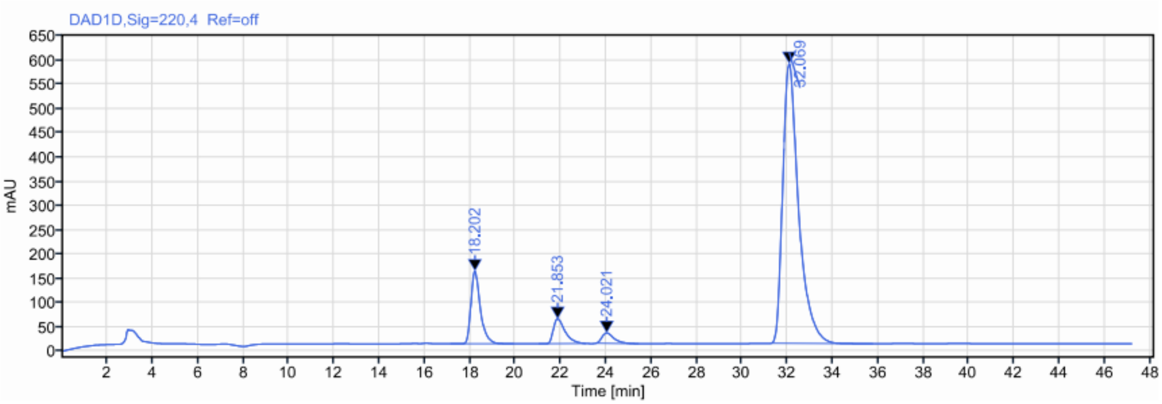

Signal: DAD1D,Sig=220,4 Ref=off

RT [min]	Type	Width [min]	Area	Height	Area%	Name
18.202	MM m	2.79	4317.03	149.72	12.31	
21.853	MM m	2.07	1822.75	51.10	5.20	
24.021	MM m	1.86	772.87	21.38	2.20	
32.069	MM m	4.48	28147.64	576.91	80.28	
		**Sum**	**35060.29**			

2-((Hydroxy)(3-hydroxyphenyl)methyl)cyclohexanone (A7)

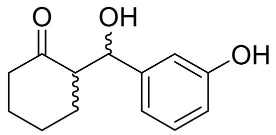

Chiralpak IC-3 (4.6 mm x 250 mm, 3 μm), *n*-hexane/2-propanol = 90:10, 220 nm, 1 ml/min

Racemate

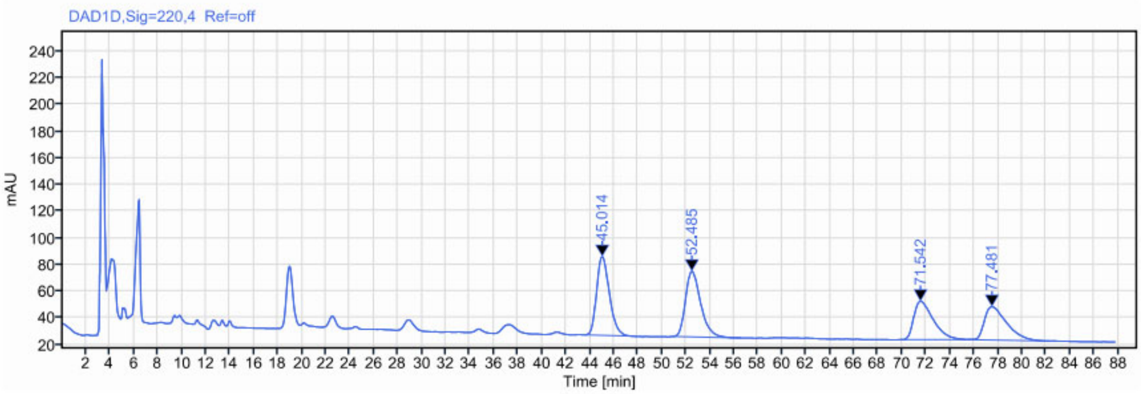

Signal: DAD1D,Sig=220,4 Ref=off

RT [min]	Type	Width [min]	Area	Height	Area%	Name
45.014	MM m	4.16	4393.48	59.20	28.88	
52.485	MM m	5.64	4353.61	49.30	28.62	
71.542	MM m	5.51	3249.82	28.84	21.36	
77.481	MM m	7.81	3215.73	25.00	21.14	
		**Sum**	**15212.64**			

Table 2, Entry 13: Reaction conducted at r.t.: *e.e.* (*syn*) = 16%, *e.e.* (*anti*) = 55%.

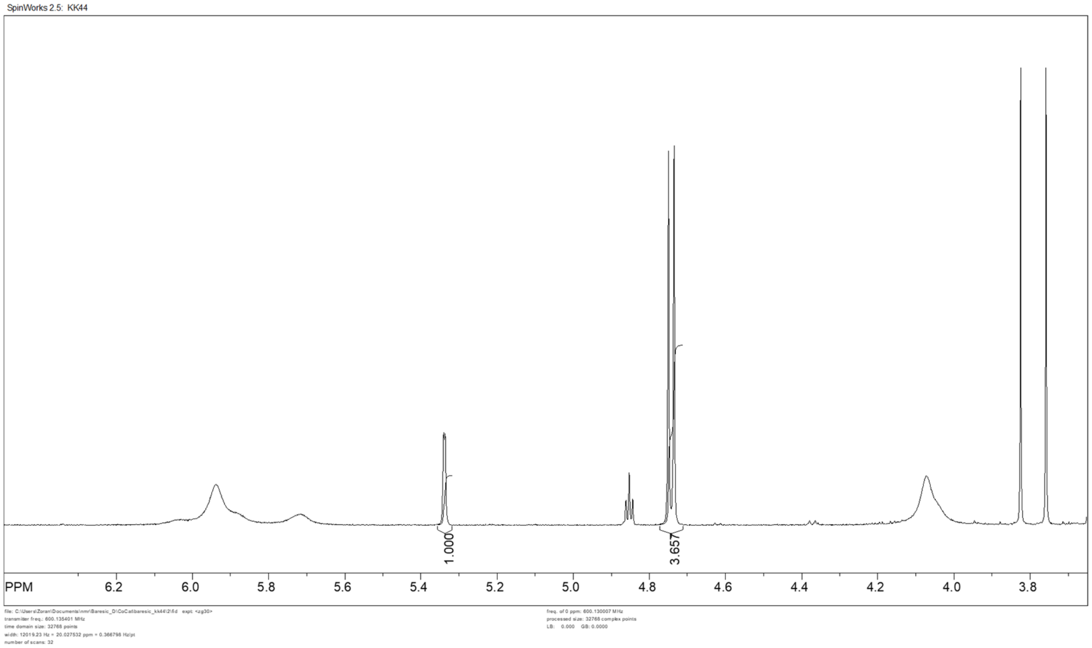

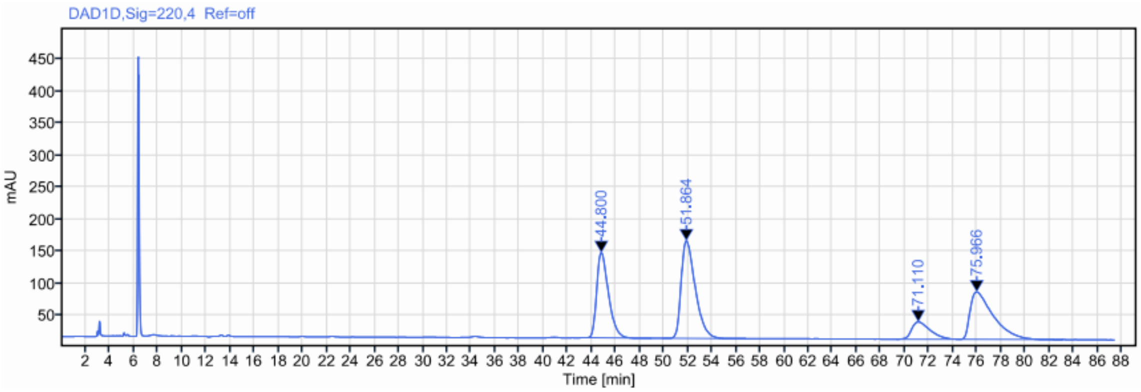

Signal: DAD1D,Sig=220,4 Ref=off

RT [min]	Type	Width [min]	Area	Height	Area%	Name
44.800	MM m	5.23	9001.59	132.43	26.44	
51.864	MM m	6.96	12357.02	151.73	36.29	
71.110	MM m	5.17	2875.53	26.92	8.45	
75.966	MM m	8.68	9813.37	73.76	28.82	
		**Sum**	**34047.50**			

S5. Cartesian coordinates

20p

C	2.2816409324	-2.3172836699	-0.1539585246
C	1.49083675	-1.7017385936	-1.3232854504
O	0.1908584398	-2.3003308003	-1.2022499148
C	-0.0458264217	-1.9418930941	0.1550389244
C	1.1513703084	-2.5875624862	0.8658353904
C	1.1144521646	-0.2306653237	-1.0620913303
C	0.0828632272	-0.3696640269	0.1068834261
C	-1.3975786919	-2.3969319125	0.627755661
N	-2.4284835562	-1.6943158486	-0.1111832602
C	-2.3437849799	-0.3915256817	-0.3943054336
N	-1.1921993125	0.2768577462	-0.2191343792
N	-3.4291305648	0.2308101419	-0.8560821391
C	-3.4749320357	1.6470238977	-1.2101245151
C	-2.4732250098	2.3827614161	-0.340422333
C	-1.114074204	1.7261943112	-0.4888057567
C	0.6173155087	0.1660124133	1.4400777294
O	-0.3574898779	0.533220262	2.2642109584
C	0.0747934952	0.9883514419	3.5581981627
C	2.2074582224	0.8001540156	-0.8881586789
O	2.0086559201	1.9449307624	-0.5536135301
O	1.7921197147	0.1808691988	1.720329504
O	3.4014141713	0.3267334665	-1.2450783217
C	4.4812725419	1.2682549513	-1.1695039919
H	-4.2787068899	-0.3142064897	-0.9283725677
H	-0.7178468131	1.8828874922	-1.498054529
H	-0.3967700143	2.1498963127	0.2157217827
H	-4.4933913332	1.9943906449	-1.0346777091
H	-3.2389785389	1.7767845218	-2.2715869206
H	-1.5078925486	-3.4658948484	0.4408214436
H	-1.4854206844	-2.2155404208	1.7057877495
H	-2.3992924179	3.4270005807	-0.6488341843
H	-2.7926771418	2.3503511358	0.7056301955
H	1.8945834487	-1.8812805863	-2.3182570118
H	4.5964604432	1.6261766138	-0.1447818812
H	4.2935130902	2.1141500253	-1.8333477936
H	5.3701579388	0.7257974077	-1.4860672713
H	-0.8335964524	1.2537175803	4.0948833589
H	0.7255153414	1.8577726225	3.4506659676
H	0.608810915	0.1904573596	4.0772479248
H	1.3483773223	-2.202005783	1.8651171598
H	2.7689920443	-3.2414818271	-0.4678898456
H	0.5504917338	0.1022732815	-1.9399194106
H	-3.3280664116	-2.1420780047	-0.2307781408
H	3.03719437	-1.6319006639	0.2292415586
H	0.935328856	-3.6559565423	0.944847928

B1

C	0.0113846894	-0.0000001375	-0.0151247065
C	-0.0025497462	-0.0000000904	1.3799777636

C	1.1910017652	-0.0000000077	2.0938064131
C	2.3874881614	0.0000000443	1.3844414423
C	2.4253497713	0.0000000027	-0.0097420081
C	1.2262771046	-0.0000000942	-0.7069238604
H	-0.9512283381	-0.0000001189	1.9102269344
H	1.1960550082	0.0000000253	3.1779056638
Cl	3.8943673287	0.0000001486	2.2692843362
H	3.376223457	0.0000000393	-0.5306713239
H	1.2190731787	-0.0000001263	-1.7923753445
C	-1.2709827299	-0.0000002509	-0.7559382435
O	-1.3525616986	0.000000275	-1.965268651
H	-2.1781689518	0.0000002908	-0.1205984155

Cyclohexanone

C	-0.0114853395	-0.0772152546	0.0075236966
C	0.0698963135	0.0222976748	1.5169951853
O	0.9722138652	-0.234008056	-0.688167621
C	-0.7094492974	1.2385173501	2.0423150861
H	-0.3818707259	-0.8923840457	1.9253264396
H	1.1214824284	0.0461739633	1.8121988125
C	-2.1452258513	1.2439924792	1.5170620025
H	-0.695740085	1.2342781776	3.1363516336
H	-0.2037806393	2.1567083491	1.7181759231
C	-2.1630782029	1.2384063258	-0.0117661218
H	-2.6735292686	0.3568027537	1.8911064054
H	-2.6800325993	2.1209837871	1.8954693835
C	-1.4075353225	0.0227441308	-0.5720953305
H	-3.190172825	1.2335276409	-0.3887205263
H	-1.6897637631	2.1569651047	-0.3806078416
H	-1.9428269792	-0.8923021953	-0.2828444413
H	-1.3354897082	0.0472158144	-1.6619576856

(S)-Proline

N	0.1552804146	0.4999621176	-0.0230161243
C	0.0788985077	0.2292518511	1.4255969269
C	1.5102878507	0.365416379	2.0099113754
C	2.4010139069	0.4831199975	0.7656581214
C	1.4639352332	1.1432333912	-0.2460612518
C	-0.8909387933	1.2278542268	2.0613666686
H	-0.3402216413	-0.765589178	1.5996700119
H	1.7719120078	-0.4836083801	2.642964287
H	1.577312038	1.2694948102	2.6216513271
H	2.6868147425	-0.5102631903	0.4036762035
H	3.3108924794	1.0592716059	0.9490208654
H	1.7682611	1.0301174344	-1.2879304839
H	1.3663419045	2.2138601916	-0.027667787
H	0.1167659911	-0.3756604705	-0.5371885346
O	-1.5836648687	1.9322854263	1.1719080685
O	-1.0242809239	1.3591573493	3.2555596414
H	-1.2249449493	1.6149164379	0.2995496844

Water

O	-0.00748297	0.	-0.0057939344
H	-0.0006412522	0.	0.9585573507

H 0.9278644805 0. -0.2406244201

1p

C	0.1662532593	0.5319185697	0.8999456165
N	0.5983306008	0.3071292211	2.1405563724
N	1.0355077272	0.9212291032	-0.0410749457
N	-1.1349348475	0.4252839494	0.6161769796
H	-0.0225174634	-0.176741852	2.7756930435
C	1.9657361463	0.545851519	2.5780611736
H	-1.3945232402	0.3080938367	-0.3551168237
C	-2.1479461183	0.1462388537	1.6233736642
H	-2.0741697066	0.8673645121	2.4407856109
H	-3.1230791634	0.2510461056	1.1513206923
H	-2.0567218693	-0.8692288519	2.023330006
H	1.988622699	0.4528065759	3.6623079598
H	2.6613863558	-0.1809117269	2.1467731139
H	2.2776550883	1.5567826508	2.3051438382
C	0.6684114829	1.4483050115	-1.3548252453
C	0.4585664643	0.3966185653	-2.3905462135
H	1.4801509011	2.1001178741	-1.6790747009
H	-0.2282996822	2.0662338792	-1.2422434053
O	-0.6649429895	-0.3640940544	-2.2590362655
C	-0.6700198388	-1.2620914787	-3.280681589
C	0.4226610254	-1.0862666192	-4.062596337
C	1.1622034762	-0.0024858572	-3.4797536014
H	2.0929666266	0.4235184132	-3.8256645839
H	0.6756073249	-1.6545412519	-4.9454592579
H	-1.5068474298	-1.9432949049	-3.3108333921
H	2.0213471709	0.7852759567	0.1421052903

1p'

N	1.7056738783	1.504742833	-0.1007855742
C	2.0851372244	0.2361749385	0.0545275135
N	3.3311659438	-0.1240050199	-0.2511250575
N	1.2097507019	-0.6513063351	0.555883961
C	2.6165732955	2.60372687	-0.3820534085
H	2.0871258542	3.5348713115	-0.186781714
H	3.4863260558	2.5494405441	0.2768983776
H	2.9475451374	2.5984903245	-1.4252936807
C	4.0573432158	-1.2582436864	0.3113427258
H	5.0682710371	-0.9264966085	0.5499811018
H	3.5723616987	-1.5860235091	1.2313354165
H	4.1121948613	-2.0927447844	-0.3911823079
C	1.145233983	-2.0698744564	0.1802317388
C	-0.2148207246	-2.4282213068	-0.3059581304
H	1.8806418517	-2.2472177147	-0.6075178221
H	1.391590615	-2.7071959567	1.0328207582
H	0.7113042279	1.6935872448	-0.1038383046
H	0.370063341	-0.2617031951	0.9700513779
H	3.8706393294	0.5308856724	-0.8029522448
O	-0.6313306006	-1.8428669503	-1.4595508845
C	-1.87904048	-2.3080414555	-1.7166934555
C	-2.2696639145	-3.1804044286	-0.753934212
C	-1.1787591912	-3.2564743831	0.1742761767

H	-1.115916196	-3.8485736994	1.0759591424
H	-3.2130936376	-3.7037929498	-0.70612043
H	-2.3528325077	-1.9295222989	-2.6100080637

1pP

N	-3.8630541232	-0.3459641107	0.448241735
C	-3.2118790552	0.7764168888	-0.3234368062
C	-3.9932001753	2.0378751066	0.0826601979
C	-5.3442475688	1.4982557955	0.5571630679
C	-4.9475684668	0.2376314397	1.3117723747
C	-1.7249068221	0.8357089478	0.0670066072
H	-3.3131702711	0.5609301671	-1.3873887525
H	-4.0709696411	2.7353656794	-0.7511559258
H	-3.4808780136	2.5420947877	0.9081609718
H	-5.9801646924	1.2410721059	-0.295717165
H	-5.8837988587	2.1972665592	1.1977926502
H	-5.7361950037	-0.5007441663	1.444835766
H	-4.4922554255	0.4770648739	2.2741385788
H	-4.2390222111	-1.0558031677	-0.1842574032
O	-1.3728501922	0.0767560561	1.0080764553
O	-1.0352539412	1.6562717035	-0.5677777119
N	1.6804413439	1.45628924	-0.1418332643
C	2.1057592565	0.211677641	0.0516300648
N	3.3812388945	-0.1190154476	-0.18150059
N	1.237212729	-0.697515139	0.5283847406
C	2.565474859	2.5717391676	-0.423333523
H	1.9873262811	3.490742024	-0.3313230398
H	3.3889847807	2.6027100603	0.2955872162
H	2.9793390251	2.522780356	-1.436992341
C	4.0786261254	-1.2472933004	0.4217385405
H	5.0872281694	-0.9254529524	0.6835514186
H	3.5624290506	-1.5503260503	1.3339297297
H	4.1443978381	-2.1010706385	-0.2572540101
C	1.2009468656	-2.0952183938	0.0843775994
C	-0.1825361026	-2.440555484	-0.3514145019
H	1.9020147529	-2.2277437886	-0.7450277075
H	1.4913815119	-2.7776274684	0.8880079609
H	0.653392087	1.5859331505	-0.2469686078
H	0.3184155591	-0.3216799695	0.8212003511
H	3.9437749064	0.5481704677	-0.6928037372
O	-0.6754250739	-1.7479443641	-1.4098013232
C	-1.95079377	-2.1630912228	-1.6047479481
C	-2.2809244232	-3.1213901027	-0.6997670469
C	-1.1213804521	-3.2987000415	0.126290365
H	-1.0022984347	-3.9683181938	0.9658127318
H	-3.2259047812	-3.6411979128	-0.6315605383
H	-2.4756379175	-1.7166907631	-2.4366717155
H	-3.101014619	-0.7775065402	1.0012015362

2p

C	0.1874338864	0.4964224354	0.9065804247
N	0.642897794	0.2556757338	2.1375419366
N	1.0347650672	0.902717276	-0.0512994788
N	-1.1149878754	0.3831168559	0.6308326042

H	0.0375832152	-0.2595164029	2.7663960606
C	1.991376311	0.5575781303	2.6403792912
H	-1.3749336241	0.2805154575	-0.3443952104
C	-2.1889890664	0.1582310537	1.6090992311
H	-1.8664367813	0.6151066129	2.549906074
C	-3.4343545211	0.8823258293	1.1139862206
C	-2.4449991593	-1.3332015062	1.8075038579
C	1.8698357625	0.9592627263	4.1046754587
C	2.9241444873	-0.636792181	2.4571321468
H	2.3592579542	1.4155871058	2.0693757404
C	0.6349088047	1.4404812846	-1.3506515038
C	0.4435004433	0.3947361191	-2.39698503
H	1.4223014992	2.1199225331	-1.6782396782
H	-0.2771839811	2.030535673	-1.2150871395
O	-0.6238018402	-0.4368729925	-2.2318462245
C	-0.6181898859	-1.3170348841	-3.2686138829
C	0.4263823929	-1.0613108984	-4.0930092653
C	1.1227347873	0.056999968	-3.5220078801
H	2.0098514186	0.5452007807	-3.899047673
H	0.675823701	-1.5993994287	-4.9955171763
H	-1.4111096476	-2.0494524603	-3.2745165014
H	2.0238218178	0.7442576265	0.090815422
H	-3.2390412987	-1.482216855	2.5438724257
H	-2.760408184	-1.7860249505	0.8620530307
H	-1.5502172459	-1.857035719	2.1561424107
H	-4.2496200319	0.7480519909	1.8286404119
H	-3.2442952288	1.9515750716	0.994018393
H	-3.7559043723	0.4721089984	0.1506686199
H	2.855764012	1.2108414312	4.502141053
H	1.2129294148	1.8246321609	4.2206204015
H	1.4670919019	0.1289655716	4.6947686579
H	3.9258413655	-0.386713885	2.8164346277
H	2.5537051336	-1.4915585791	3.0317731523
H	2.9995205736	-0.9366116835	1.4074499912

2p'

N	1.790613148	1.5484292451	-0.0771611096
C	2.1564008656	0.2663259054	-0.0247404133
N	3.4173319624	-0.0953570841	-0.2899858792
N	1.2332471705	-0.6456424985	0.3148728697
C	2.6830445493	2.7158166693	-0.0919753383
C	2.045600162	3.8122937183	0.7516610906
H	3.6207085436	2.404205509	0.3793484889
C	2.9465175104	3.1811037524	-1.5213186647
C	4.1594036496	-1.2318344737	0.3051051233
C	5.6079771581	-1.0941532654	-0.1417265366
C	4.0389516436	-1.2413796984	1.8242098607
H	3.7647935283	-2.1670287507	-0.099500663
C	1.2200563272	-2.047897018	-0.1136417161
C	-0.1772331166	-2.4977214001	-0.3545279988
H	1.8058755959	-2.1316995953	-1.0336506713
H	1.666935097	-2.6964178884	0.6469055331
H	0.7942217724	1.7363011422	-0.1216425663
H	0.3764833184	-0.2863278941	0.7204403325

H	3.9938484832	0.6116539709	-0.7320404369
H	2.6979472455	4.6882600093	0.7738362839
H	1.0853239292	4.1144091856	0.3200369351
H	1.87934496	3.4723253454	1.776675925
H	3.6226764112	4.0400385798	-1.5169871468
H	3.3986225809	2.388789239	-2.1252118963
H	2.0075606841	3.48134788	-1.9968778694
H	6.1893675969	-1.9369567353	0.2376001991
H	5.6842201769	-1.0850227897	-1.2325512297
H	6.0435322858	-0.16970936	0.2521674708
H	4.6004518009	-2.0890378914	2.2257682421
H	4.454575551	-0.3185530538	2.2413015389
H	2.9999266661	-1.3370153488	2.1512895621
O	-0.8399713996	-1.9196637797	-1.3910890761
C	-2.0781308877	-2.4704496089	-1.4315565136
C	-2.2227255847	-3.3898411961	-0.4443638456
C	-0.9764188961	-3.4049097907	0.2650960737
H	-0.7080796021	-4.0084433568	1.1202664282
H	-3.1026908229	-3.9823377822	-0.2423298943
H	-2.7334380642	-2.1089058917	-2.2097534917

2pP

N	-3.8689796154	0.3277373138	-0.4293092313
C	-3.2105413868	-0.767624214	0.3737210994
C	-3.9837430385	-2.0487922156	0.0105408839
C	-5.3160884669	-1.5383529377	-0.5446337839
C	-4.8963292281	-0.3027241478	-1.3268828462
C	-1.7231386063	-0.8241609324	-0.0175878889
H	-3.3051303421	-0.5173550264	1.430827369
H	-4.0968269451	-2.697978551	0.8786179649
H	-3.4413611875	-2.6010615929	-0.7629221897
H	-5.9921591922	-1.2548016988	0.2679851262
H	-5.8209088375	-2.2661416762	-1.1814561795
H	-5.6860008736	0.4180928518	-1.5313263409
H	-4.3845419646	-0.5714000124	-2.2529298053
H	-4.2951934965	1.0279887133	0.1818244873
O	-1.365247929	-0.0372210405	-0.9344069482
O	-1.0351566119	-1.6611785189	0.5960011303
N	1.6971008722	-1.4754088731	0.0875090749
C	2.1202241061	-0.2183002088	-0.0188279403
N	3.4043890078	0.1102888891	0.2008809266
N	1.2228269768	0.7129788302	-0.3768164257
C	2.5408303649	-2.6689499114	0.1761543757
C	1.8324269541	-3.802142085	-0.5577635425
H	3.4835860701	-2.4478601667	-0.3370332616
C	2.8181357232	-3.0336749146	1.6335040121
C	4.1401028482	1.2264289507	-0.4294139889
C	5.5901243503	1.113542011	0.0208196445
C	4.018757574	1.1949971052	-1.9488612073
H	3.738610066	2.1715577844	-0.0547881128
C	1.2093918246	2.0926265787	0.1115195682
C	-0.1928166242	2.4668179675	0.4529179008
H	1.8440389913	2.1696426944	0.9997688138
H	1.5858026044	2.7907237472	-0.6437760981

H	0.6730102455	-1.6006890718	0.2165484638
H	0.3012424298	0.3557044709	-0.6875604499
H	3.9844632775	-0.6165174022	0.6026296335
H	2.4358666294	-4.7122107402	-0.5165071991
H	0.8654593022	-4.0104183792	-0.0877551356
H	1.6603201251	-3.541670733	-1.6050904868
H	3.4499957836	-3.9244473922	1.688524392
H	3.3232062877	-2.2192839334	2.1617506655
H	1.8758380327	-3.2433472137	2.1499542637
H	6.1683429603	1.9472298196	-0.3830043067
H	5.665983891	1.1353870284	1.1115398317
H	6.0317200711	0.1801737806	-0.3448469662
H	4.5791301238	2.0304556989	-2.3772377549
H	4.4325474526	0.2604060251	-2.3412856415
H	2.9778053243	1.2802231826	-2.2719184173
O	-0.7607773877	1.7914315261	1.4850622068
C	-2.0429320482	2.2175680777	1.5886847284
C	-2.30580893	3.163084758	0.6487460313
C	-1.09177566	3.3228576358	-0.0988638683
H	-0.9120470259	3.9799079697	-0.9375187448
H	-3.2399607256	3.6881521612	0.5098116716
H	-2.6245068533	1.7889462766	2.3918665986
H	-3.1007902934	0.7802527412	-0.9569751023

4p

C	0.1700217689	0.2641606615	0.1529698206
N	0.0831309496	0.2529182992	1.4908914255
C	1.3741355091	0.6344613727	2.0755859623
C	2.3356038854	0.3119513807	0.9226217023
N	1.4405422336	0.4112390019	-0.2372180549
H	-0.7953467895	0.442135982	1.9577449891
H	1.5847120335	0.0500252064	2.9699256977
H	1.3807020026	1.7006244803	2.3199520876
H	2.7374078436	-0.7019987872	1.0030676058
H	3.1524117357	1.0277677861	0.8446512105
H	1.7505455706	0.1960168743	-1.1762413524
N	-0.8619789628	0.1419039596	-0.6666774001
C	-0.7350503098	0.1722016921	-2.1281574142
C	-2.0383058809	-0.1442635062	-2.7663120297
H	-0.4101984302	1.1624055817	-2.4614094576
H	0.0166663473	-0.5647457347	-2.4318033426
H	-1.7909289084	0.1555567296	-0.2606619728
C	-2.9170524196	0.5954191071	-3.4913144591
C	-3.9950662782	-0.2867135495	-3.831274411
C	-3.6830679237	-1.4864807754	-3.2797322736
O	-2.4952676399	-1.4171323193	-2.6301674844
H	-4.1795595382	-2.4450068999	-3.2601548109
H	-4.8786821665	-0.0549960168	-4.407121893
H	-2.8074666321	1.6383904737	-3.7515151453

4pP

C	0.3202101158	0.5068750437	0.8522705922
N	0.5945222402	-0.0725370497	2.0209464191
N	1.2453437228	0.8827386038	-0.0149215448

N	-1.0104394075	0.6775122482	0.6844552715
H	1.5549177749	-0.0237970324	2.4200267088
C	-0.6277110399	-0.1765894401	2.8197114525
H	-1.3935993728	0.6957992801	-0.253316631
C	-1.7176442113	-0.0565262736	1.7434633897
H	-2.5891530816	0.4982426411	2.089148135
H	-2.0302378237	-1.0395983804	1.3785713288
H	-0.672030412	-1.1255673981	3.3538333132
H	-0.6843851633	0.6462964928	3.5395455869
C	0.9403575574	1.5885981755	-1.2561097022
C	0.5464243519	0.6879818962	-2.3780918305
H	1.8394605899	2.1327889712	-1.5471195005
H	0.1518552861	2.3261422156	-1.0674676231
O	-0.6814974552	0.1038702859	-2.2815463934
C	-0.8475417709	-0.678182117	-3.3815327267
C	0.2445550646	-0.6009386017	-4.1801296545
C	1.1572668791	0.2916238374	-3.523222497
H	2.1378536167	0.5979863023	-3.8577979779
H	0.387030625	-1.112774474	-5.1203907355
H	-1.7821993403	-1.2140441789	-3.447242069
H	2.2387522215	0.7246076082	0.2529207736
O	3.8443404964	0.5195697979	0.7894229365
C	4.0313613014	0.2995118699	2.015651578
O	3.1871743696	0.09904229	2.9086494094
C	5.4967410532	0.2822568054	2.4844822285
C	5.8000844296	1.3195269026	3.5818239709
C	7.132527299	1.944053443	3.1567568177
C	7.0083204322	1.9939983881	1.6417605665
N	6.3787036512	0.6679125466	1.3223571174
H	5.013158844	2.078760976	3.5974881394
H	5.8400651119	0.851078063	4.5649772044
H	7.9719536561	1.3016347742	3.4405034039
H	7.2931391203	2.9361629848	3.5808214166
H	7.9428569622	2.0900650335	1.0919732351
H	6.3028579047	2.7637090665	1.3229773732
H	7.0994773777	-0.0391762993	1.1578526851
H	5.7616650178	0.7085016892	0.4942931027
H	5.7671150053	-0.7297369875	2.7878867295

5p

C	-0.0873971062	1.0671650133	-0.1742886606
N	-0.2181798144	2.0216731464	0.746281473
N	1.0620922384	0.9266234168	-0.8474891972
C	-1.4281551562	2.2107197116	1.5521171724
H	0.5037170167	2.7260912295	0.8136049869
C	-2.1866076725	0.8973307041	1.6530138725
H	-1.1152819882	2.5628236562	2.5360824284
H	-2.0534405151	2.9814237725	1.0899102957
C	-2.389842983	0.3226133379	0.2594011487
H	-1.6243123673	0.1865984877	2.2657899962
H	-3.1536929911	1.0729322135	2.1273224247
N	-1.0920460337	0.2212739613	-0.408554157
H	-2.8129553897	-0.6812016366	0.3024613064
H	-3.0620335596	0.9581650915	-0.3268816615

H	-0.932636932	-0.5327167887	-1.0640821641
C	2.2433614846	1.7659203748	-0.6469282381
H	2.1038963978	2.7590837352	-1.0870694732
H	2.4133191093	1.8814199537	0.4293254296
C	3.4329734209	1.1315088673	-1.2727950046
C	4.2074186434	1.4662489729	-2.3373161803
C	5.2109758952	0.4464732577	-2.4365460526
C	4.9580728785	-0.4242583678	-1.4273032375
O	3.8807864518	-0.0212454971	-0.7096753883
H	5.4313936284	-1.3380927704	-1.1007536117
H	6.0079505493	0.3754357631	-3.1617723788
H	4.0777463898	2.3324818159	-2.9700481411
H	1.0623564047	0.2996935772	-1.6433419878

5pP

C	-0.3499242634	0.8040803489	0.0288280352
N	-0.2702667788	1.982169638	0.6586075728
N	0.6974628804	0.3132548499	-0.6337756495
C	-1.4124275314	2.5790423711	1.3517196023
H	0.6348937818	2.4229076127	0.7448892683
C	-2.3090917887	1.4726113866	1.8885205773
H	-1.0195190045	3.1979240419	2.1589293737
H	-1.9694665663	3.2238361113	0.6631651172
C	-2.6837651571	0.5316892507	0.7531697373
H	-1.7807748629	0.9208405661	2.6721191586
H	-3.213670398	1.9042004796	2.3214266675
N	-1.4802767585	0.0936977843	0.0515807997
H	-3.1933655114	-0.3561461452	1.1334472778
H	-3.358756478	1.0370175248	0.0524908216
H	-1.5487969567	-0.6757165516	-0.6331458278
H	0.6500536379	-0.6690620244	-0.9727719546
C	1.92567786	1.0536143056	-0.8987386663
H	2.5361454864	1.1458485105	0.0088142175
H	1.6906942675	2.0603546377	-1.2589243624
C	2.6859149323	0.3340424473	-1.9583228923
C	2.8435136078	0.5358383642	-3.2930364982
C	3.5914045961	-0.587501121	-3.7794620225
C	3.8185378475	-1.3800895777	-2.6991348926
O	3.2849084498	-0.8249893884	-1.5855163202
H	4.3483469509	-2.3112504359	-2.561172735
H	3.9191200006	-0.7717952781	-4.7924609119
H	2.4667149075	1.3758101092	-3.858833167
O	0.651928024	-2.1857619692	-1.6992196956
C	-0.4539981853	-2.3649644111	-2.2804601408
O	-1.5512189732	-1.8439238091	-2.0173830478
C	-0.4291385895	-3.3442489634	-3.4768033214
N	1.0063150304	-3.7719719784	-3.6736143385
C	1.4740373924	-3.2819349453	-5.0172499564
C	0.5319363087	-2.1215984552	-5.3034669783
C	-0.8090384486	-2.6543275158	-4.7917769186
H	1.5152601839	-3.2919121232	-2.9034345251
H	1.1338307607	-4.7805223226	-3.5679362955
H	1.3329800795	-4.0964109662	-5.7287495873
H	2.5293341134	-3.0205027205	-4.9480285745

H	0.5201653849	-1.8663840276	-6.3637785142
H	0.8413544715	-1.2388308864	-4.7323159828
H	-1.2216976404	-3.3808593942	-5.4969843726
H	-1.5528498293	-1.878574782	-4.6085723259
H	-1.0380772339	-4.2183075478	-3.2469047513

6pEn

C	3.5883582808	3.6751739483	-1.5714363291
N	3.9513031666	2.4116803229	-0.9174384352
C	2.9148694129	1.5469847193	-1.0353442858
N	2.9543784526	0.2702532648	-0.7195333648
C	4.1626242869	-0.3671307218	-0.224246942
C	3.8204255345	-1.7849532265	0.2236215155
C	5.0690586697	-2.4670275658	0.7545172607
N	4.8697771889	-3.9345010901	0.9913844391
N	1.8206651897	2.1635095736	-1.4951935475
C	2.0538344748	3.6068247054	-1.5305999413
H	4.9023371971	2.06746881	-0.961829666
H	0.8895908163	1.7059960124	-1.3545893588
H	4.5718425865	0.2032016117	0.6199173489
H	4.9313670192	-0.3995464782	-1.0076253487
H	3.0400851739	-1.7333017402	0.9879562902
H	3.4183723403	-2.3451525785	-0.6288196847
H	5.3924006681	-2.0446122705	1.709651057
H	5.888922633	-2.3871660826	0.0364335121
H	2.0378218125	-0.2949584247	-0.8003381731
H	3.9592121629	3.6954896785	-2.6012683614
H	3.9858896045	4.52945967	-1.0242588474
H	1.6590214977	4.0853814711	-0.627710501
H	1.5945478264	4.0632943781	-2.4074885097
O	0.7529803884	-1.1261945712	-0.8619361183
C	-0.3327825373	-0.4845026217	-0.9599567342
O	-0.4669950355	0.7541588307	-1.0873586223
C	-1.606261519	-1.3536308958	-0.9662404302
C	-1.7976327678	-1.964393848	-2.3607037591
C	-2.4557734603	-0.8136193914	-3.1292869096
C	-3.3425026219	-0.1257860097	-2.0789143962
N	-2.8412145648	-0.6063800199	-0.7951202028
H	-0.8529016288	-2.2977682777	-2.794825829
H	-2.4757103915	-2.8206115968	-2.2911434371
H	-1.6864477666	-0.1159148322	-3.4708767451
H	-3.0231174859	-1.1493974485	-3.9996767461
H	-3.2621964317	0.9678090221	-2.1416102204
H	-4.404161756	-0.3846893522	-2.1918159133
C	-3.1685244225	0.0657210459	0.3722886909
H	-1.4939032454	-2.1273799361	-0.2015486794
H	4.5696823433	-4.3514370177	0.1020211463
C	-4.1488630728	0.9942909866	0.4303818361
C	-4.6170261147	1.6417519515	1.7104296265
H	-4.6671081332	1.281570345	-0.4795432582
C	-4.1352938422	0.8809010157	2.944319349
H	-5.7125397752	1.7018060376	1.7132529887
H	-4.2643419278	2.682998639	1.7706215071
C	-2.6502547945	0.5644899319	2.8007105904

H	-4.6950215648	-0.0590971537	3.0370837209
H	-4.3244826957	1.463457924	3.8518174352
C	-2.4055717518	-0.3615274974	1.6095212047
H	-2.2532325492	0.1028956287	3.7103979554
H	-2.100085193	1.5020120048	2.6484002551
H	-2.6919669062	-1.3911924346	1.8653387941
H	-1.3333936085	-0.3885134226	1.3859622877
C	6.1560153759	-4.5807021442	1.3782995811
C	3.8105113406	-4.2200755464	2.0002201491
H	3.7757363547	-5.296856443	2.1595557225
H	4.0785233541	-3.7055617723	2.9237222452
H	2.8506202021	-3.8685550257	1.6276627139
H	5.9860105489	-5.6506587906	1.4878140023
H	6.8913637944	-4.3879446383	0.5981393415
H	6.4790798665	-4.1468886629	2.3251477307

6pP

C	2.4932016317	1.0161905912	1.4283298566
N	2.1499649017	0.1105781851	0.3351984183
C	3.303400953	-0.0921673798	-0.5353459842
C	1.63627172	-1.15062296	0.882598231
C	1.1739789471	-2.1435673344	-0.1807301349
C	0.1964536528	-1.5651838575	-1.2034782794
N	-1.0732941365	-1.1249257624	-0.6338065393
C	-1.333681563	0.1350381699	-0.3112451294
N	-0.418610932	1.1065770747	-0.2481097025
C	-1.0563589433	2.3763634306	0.1005329798
H	-1.1168053768	3.0261365714	-0.7778722052
N	-2.5606359375	0.5892803077	-0.0172027516
C	-2.4562406537	1.9266450463	0.5690529193
H	-0.0378896929	-2.3281967105	-1.9496980595
H	0.6527924922	-0.7226243529	-1.7339314863
H	2.0323120001	-2.5480256638	-0.727518159
H	0.7016283699	-2.989018355	0.3308093976
H	2.3997588644	-1.6313592675	1.5180912026
H	0.7917883459	-0.8961614675	1.5341244094
H	0.6000676002	0.8991873457	-0.2215893511
H	-1.8679846494	-1.7863372342	-0.6019273838
H	-0.4996324149	2.8916050242	0.8841176
H	-3.3361919647	-0.0712866842	0.1795774162
H	3.6775372987	0.8796590762	-0.8666346913
H	4.1193267333	-0.6221022895	-0.0165617211
H	3.0223936553	-0.6672714649	-1.4206405089
H	2.825766822	1.9745953202	1.0206572034
H	1.6099760053	1.1875473859	2.0515375558
H	3.2945585999	0.6090262486	2.0659235749
H	-2.5215709848	1.8766203171	1.6613824143
H	-3.2438634085	2.5817983358	0.1961224342
O	-3.2616715288	-2.8523273755	-0.5180575846
C	-4.305788699	-2.4204666173	0.0367604379
O	-4.5437717832	-1.2787278068	0.4729874702
C	-5.4451222486	-3.4385378456	0.2233491768
C	-5.8028621045	-3.6890299518	1.6988963986
C	-6.0393677433	-5.1994601235	1.7754646479

C	-4.966011537	-5.7495243441	0.8481248305
N	-4.9903450676	-4.7793037963	-0.300626054
H	-4.9592963067	-3.4063102517	2.3357302698
H	-6.6693297364	-3.0995570397	1.9978697999
H	-7.0317924534	-5.457361576	1.3927647618
H	-5.9398996562	-5.5974629842	2.786251363
H	-5.1374118416	-6.7559896878	0.4704181044
H	-3.974681596	-5.6820997311	1.2995021804
H	-5.6119446195	-5.1136481778	-1.0406378215
H	-4.0531414477	-4.6198650872	-0.7077281267
H	-6.3125545659	-3.1278732501	-0.3597473801

7pEn'

C	-5.2386684543	-1.5638326041	1.4708476531
N	-4.2776117293	-2.0475815145	0.4713586652
C	-3.6301436725	-1.0052732662	-0.0741866749
N	-2.591613957	-1.0785753602	-0.8887643997
C	-1.8056320377	-2.2768585361	-1.165721585
C	-0.7442550518	-2.5480871066	-0.0976685463
C	0.2112556666	-1.3673536954	0.1464573772
C	1.1042813105	-1.0880767318	-1.0410771683
N	2.2696194596	-1.7208787158	-1.1196530871
N	-4.1912374699	0.1475889454	0.3018054905
C	-5.4351340368	-0.1033262219	1.0302049902
O	0.7463051012	-0.3184831931	-1.9693534824
H	-3.8527306585	-2.9633161863	0.5379629323
H	-3.9004707287	1.044504519	-0.0815894498
H	-1.3431591565	-2.1201302345	-2.1430008231
H	-2.4775404205	-3.1346008767	-1.2588636272
H	-0.1801065589	-3.4394501732	-0.3933558269
H	-1.2419294619	-2.7808538292	0.8497833884
H	0.8166062915	-1.5614462506	1.0343938487
H	-0.3640675109	-0.457724948	0.3409451826
C	2.8390196865	-2.6172954381	-0.0974012626
C	4.0722619305	-3.1932600283	-0.7945086388
C	4.5121300225	-2.0546803308	-1.7189386686
C	3.1860060139	-1.5186454468	-2.2528761637
H	3.11671394	-2.0298464058	0.786068991
H	2.1182983837	-3.3823719858	0.1988084543
H	3.7870733258	-4.0675725671	-1.3880270755
H	4.8392026207	-3.4950062681	-0.0796013948
H	5.1778125394	-2.3804400702	-2.5196371016
H	5.0183886073	-1.2753929202	-1.1395818937
H	2.8312445679	-2.0946918213	-3.1147787639
H	3.2083008786	-0.4636629667	-2.5314667873
H	-2.2432092812	-0.1935844983	-1.2625546977
H	-4.8077692907	-1.627721533	2.4743986324
H	-6.1629811363	-2.1384825617	1.4316993973
H	-6.29771712	0.0040650555	0.3656085485
H	-5.5384831526	0.5764637879	1.8749356797
O	-1.162843736	1.1936894431	-1.6267663314
C	-1.3040749505	2.1691101821	-0.7420705921
O	-2.4104429134	2.477835013	-0.3359373255
C	-0.0449623328	2.9234098704	-0.3224070413

C	0.4630468788	3.8026508025	-1.4864100246
C	1.5366455916	2.9410872445	-2.1551948724
C	2.1817019427	2.2308060805	-0.9714533533
N	1.0706852475	2.0223008745	-0.047910371
H	-0.3396739662	4.1089885003	-2.1615050996
H	0.9145077389	4.7036879636	-1.0624275971
H	1.0837067436	2.2056311675	-2.8269580324
H	2.2538317554	3.533255737	-2.726502463
H	2.6293290514	1.2720280039	-1.2517111779
H	2.9633353261	2.8467249185	-0.4992440224
C	1.3057459828	1.4785882135	1.2137108065
H	-0.3241914025	3.5320718725	0.5408052414
H	-0.2487994424	0.682377122	-1.6988282378
C	2.4773400234	0.8930971287	1.5409830884
C	2.751898844	0.2767697419	2.8911716766
H	3.2780745636	0.8443579472	0.8079068119
C	1.7339175662	0.7128915459	3.9433080013
H	2.7423502683	-0.8223503638	2.8170192481
H	3.7650803413	0.541508353	3.2165133164
C	0.3227116198	0.6032793482	3.3739122199
H	1.9245746315	1.7557159811	4.227746674
H	1.8373302743	0.105337141	4.8478344766
C	0.1499710334	1.5539098566	2.1909172815
H	-0.4295885351	0.8291411547	4.1357288924
H	0.1476610189	-0.4289688309	3.042398212
H	-0.7934374872	1.3379052807	1.673065853
H	0.0611208618	2.5857306852	2.5571446292

7pEn

C	0.915612361	3.5293491693	-0.4688795389
N	1.1998089633	2.1183599073	-0.1980317184
C	0.098922726	1.3880171846	-0.3786844714
N	0.0717555171	0.0622903523	-0.3346233404
C	1.2933960745	-0.7252226914	-0.1974820333
C	1.8693892379	-0.7009359432	1.2218659978
C	3.334743721	-1.1185572445	1.2296317362
C	4.2387284039	-0.0416217427	0.6482096071
N	5.5552286918	-0.291843954	0.6586606787
N	-0.9673240834	2.1656037641	-0.6221085248
C	-0.6181512349	3.5653936474	-0.3584459929
O	3.7982069114	1.0249086054	0.1900941033
H	2.1411289014	1.7211406327	-0.0978900787
H	-1.9193795597	1.8084502247	-0.5287033037
H	1.0499880898	-1.746904101	-0.493580116
H	2.0307093704	-0.3440369524	-0.911365194
H	1.7854351824	0.3066405924	1.639710404
H	1.2864798294	-1.36806895	1.8623392138
H	3.6705206473	-1.3355459641	2.249737479
H	3.4785393626	-2.0443575546	0.6579734118
C	6.204828871	-1.501077951	1.1816557663
C	7.6421406858	-1.3583264156	0.6774756516
C	7.8589144613	0.1577858271	0.6755682926
C	6.5222254369	0.6974538395	0.1667338199
H	6.1664533517	-1.5094042265	2.2778365182

H	5.7132829319	-2.4033781403	0.8103841213
H	7.7138558921	-1.7475443971	-0.3431991491
H	8.3526542024	-1.8989467692	1.3049870969
H	8.6965335819	0.4730112575	0.0510855992
H	8.0366747579	0.5105567347	1.6965518906
H	6.4886303654	0.736911465	-0.928283707
H	6.2713083901	1.6883627021	0.5505106987
H	-0.8275520064	-0.4007685127	-0.4120875022
H	1.2544446562	3.7990234813	-1.4738915347
H	1.4028615147	4.173459568	0.262419367
H	-0.9424517335	3.8590275392	0.6449380116
H	-1.0729126682	4.2269028206	-1.0949631151
O	-2.7690327427	-0.9841730045	-0.4794025651
C	-3.8040603597	-0.1486730526	-0.4499602542
O	-3.6597775998	1.0544510517	-0.4100586033
C	-5.1638382047	-0.8409218614	-0.4262326468
C	-5.7787518897	-0.7518538964	0.9875498638
C	-5.3758589507	-2.0812437143	1.6268760541
C	-5.5355331779	-3.0430471708	0.4584080006
N	-4.9792934912	-2.2863428355	-0.6754640304
H	-5.4357599559	0.1323933079	1.5277295403
H	-6.8659868091	-0.6968612334	0.8920306991
H	-4.3324721822	-2.0539214373	1.9596106584
H	-6.004185153	-2.3507769201	2.4770688056
H	-4.9987584264	-3.9876873801	0.5746930426
H	-6.5999706978	-3.262626707	0.2946247882
C	-5.1781443776	-2.7866053666	-1.9926796981
H	-5.7992756097	-0.3493557136	-1.1669708389
H	-3.1896354849	-1.8869434265	-0.5276063223
C	-5.8116060668	-3.9433515433	-2.235922231
C	-5.9030161422	-4.5713593741	-3.6037748426
H	-6.2738689945	-4.4902619094	-1.4198836919
C	-4.9114698819	-3.9561992133	-4.5899666304
H	-5.7332526402	-5.6508069268	-3.5180601698
H	-6.9260570774	-4.4598344126	-3.9903562053
C	-4.9449211126	-2.4349214156	-4.4807199698
H	-3.8985022125	-4.3093069328	-4.357855574
H	-5.1421710915	-4.2790667638	-5.6096302567
C	-4.5021353318	-1.9890976129	-3.0871108508
H	-4.3005829261	-1.9708071689	-5.2330984471
H	-5.9671480177	-2.0825090923	-4.6666884849
H	-4.7053128757	-0.9186994358	-2.9673338298
H	-3.4140853203	-2.1078346427	-2.9848714543

7p

C	0.6648973359	3.5901038465	0.1728421552
N	0.8362133761	2.1390188937	0.0647029834
C	-0.3305453894	1.5576346929	-0.20227294
N	-0.4731575517	0.2677310799	-0.4719770644
C	0.678165394	-0.6213498555	-0.6076122082
C	1.3201642612	-0.9797263558	0.7363161936
C	2.7445717447	-1.4869439342	0.5482459893
C	3.7062592834	-0.3673626175	0.1793689367
N	4.9972999139	-0.7012673033	0.0521845684

N	-1.3346266883	2.4539864193	-0.1777330437
C	-0.8497993413	3.7093400099	0.4085900876
O	3.3329336536	0.8049763518	0.0084734181
H	1.7436383774	1.6593915161	0.0058494039
H	-2.2941159543	2.1503978213	-0.0679532363
H	0.3292656187	-1.520148107	-1.1178119177
H	1.4102461619	-0.1360396431	-1.2610121194
H	1.3400577771	-0.1001270954	1.3865638411
H	0.7151344383	-1.7401218194	1.2368481602
H	3.1086290333	-1.9655789784	1.4639422277
H	2.7830339064	-2.2559317927	-0.233834066
C	5.5706766471	-2.0402711255	0.2457594452
C	6.9974859535	-1.8781395376	-0.2821422309
C	7.3274540672	-0.4206583206	0.0527564667
C	6.0191061669	0.3085062018	-0.2520020645
H	5.5673369768	-2.3036817354	1.31051074
H	5.0011651081	-2.7956193052	-0.3007294216
H	7.0067363611	-2.0240351981	-1.3670134711
H	7.6852513754	-2.5951325745	0.1692190881
H	8.1652909345	-0.0245539931	-0.523475542
H	7.5645240343	-0.3269484122	1.1174100918
H	5.9508481986	0.5995816678	-1.3066868916
H	5.8570247132	1.1992363051	0.3581893319
H	-1.4086806586	-0.093598546	-0.6053268781
H	0.9677892199	4.0775460002	-0.7585718212
H	1.2498509206	3.9910864455	0.999766357
H	-1.0894785802	3.7518532788	1.4754407963
H	-1.2841007892	4.5696827198	-0.0988093654

7pP

C	0.6332381478	3.6084146765	0.1784435802
N	0.8270724556	2.1600311589	0.0998374286
C	-0.335355378	1.5626653274	-0.1924203932
N	-0.4706592189	0.2738942972	-0.4640281768
C	0.6875414262	-0.5993761367	-0.5980380744
C	1.3341434558	-0.9610360055	0.743722076
C	2.7549166148	-1.4778505267	0.5533242157
C	3.7248454457	-0.3628672913	0.190999071
N	5.0139920537	-0.708291668	0.0568074797
N	-1.3511826652	2.4376164617	-0.1786270565
C	-0.8878705565	3.7045340297	0.3904694119
O	3.3623917473	0.8130688369	0.0332437016
H	1.7378377791	1.6971873161	0.0263694397
H	-2.3309527101	2.1009026654	-0.1284096766
H	0.3511472414	-1.5032785649	-1.1103200672
H	1.4231133848	-0.1125571508	-1.2475663324
H	1.3612262277	-0.079751789	1.3914196629
H	0.7255114473	-1.7165265293	1.2480806999
H	3.1180965371	-1.9652751716	1.4649362951
H	2.7874940671	-2.2418651903	-0.2340890539
C	5.5754808615	-2.0539685606	0.233112248
C	7.0008790408	-1.8992120754	-0.3007608276
C	7.3466808516	-0.4493004641	0.050678296
C	6.0434055474	0.2960660856	-0.2373046223

H	5.5757089814	-2.3300875964	1.2947592702
H	4.9964301157	-2.7982142076	-0.3187404286
H	7.0024682134	-2.0311350953	-1.3875066257
H	7.6846605479	-2.628399619	0.1370575291
H	8.1853165693	-0.0539838445	-0.524987077
H	7.590422311	-0.3716447771	1.1151575425
H	5.9736623947	0.604254589	-1.2871031954
H	5.8924418502	1.1792615849	0.3866828289
H	-1.4314096326	-0.0868124874	-0.6000849068
H	0.939850214	4.0885482816	-0.7566784228
H	1.1994199817	4.0367539217	1.0053156794
H	-1.1394273239	3.7658748709	1.4549772792
H	-1.3262812472	4.5554804666	-0.1307765837
O	-3.0860878981	-0.6058036423	-0.7769720465
C	-4.0044371503	0.1978827022	-0.4664879117
O	-3.9138915256	1.3855958312	-0.1053281074
C	-5.4388960012	-0.3594411777	-0.5320358642
C	-6.1901057758	-0.2777374919	0.8098067778
C	-6.8824411507	-1.6375741724	0.9418878669
C	-5.859173279	-2.5910506952	0.3441640521
N	-5.3653937912	-1.8320094749	-0.8541973065
H	-5.4732476421	-0.1363555816	1.6234942389
H	-6.886100737	0.5608808832	0.8205099666
H	-7.804502299	-1.664022212	0.3527832803
H	-7.1201059288	-1.8971696275	1.9743831776
H	-6.2425389206	-3.5582982609	0.0238874065
H	-5.0031978916	-2.7263211524	1.0080018647
H	-5.9279982166	-2.058076739	-1.6777781042
H	-4.371872864	-2.0245736394	-1.0682788595
H	-5.9817587078	0.133468632	-1.3392876459

11p

N	2.1826390778	2.78201239	0.1638081224
C	1.2673102793	2.0457584642	-0.4782937106
N	0.1450400546	2.7442671272	-0.6892866549
C	0.2294813767	4.0339064265	0.0118065269
C	1.7430932648	4.1810652611	0.2254584053
N	1.4480984883	0.7802751357	-0.8389046784
C	0.5101531457	0.0322587406	-1.6631769787
C	-0.4256770116	-0.8791855654	-0.8954690425
O	0.2656551623	-1.9680972623	-0.2788311477
C	-0.8840611499	-2.6451401384	0.2513568103
C	-1.7287898482	-2.8909166098	-1.0061600367
C	-1.4452565242	-1.5966616793	-1.8085648699
C	-1.1292255361	-0.2667898602	0.348142325
C	-1.5182156834	-1.527318508	1.1301969073
C	-3.0324548215	-1.4981953914	1.1771508226
O	-3.7729362352	-2.3073905034	1.6749085876
C	-2.4201326454	0.4425349615	0.0436341189
O	-2.5376670893	1.4972435881	-0.5534297784
N	-3.4692816297	-0.3316260605	0.5106880748
C	-4.8449410223	0.0016652355	0.3238796768
H	2.3477695979	0.3617858988	-0.6344000971
H	-0.3095563465	3.9759349382	0.9616743496

H	-0.1866818899	4.8350878164	-0.5971822005
H	2.2095122135	4.7586973337	-0.5773593909
H	1.9889037344	4.6256820843	1.1887066972
H	-0.0835510542	0.7337150761	-2.258153035
H	1.0890198551	-0.5760814211	-2.3617637654
H	-0.5772453966	-3.5310293317	0.8034897429
H	-2.336611922	-0.9968452338	-2.0088987891
H	-1.3558128212	-3.7707587116	-1.5324722497
H	-0.7619985826	2.2945775645	-0.8234848341
H	-0.9726791606	-1.8182290336	-2.768424777
H	-2.7873543645	-3.0497436297	-0.7890253288
H	-1.126760459	-1.5522846169	2.1481246502
H	-0.4515011661	0.3906455031	0.8955566837
H	3.1632918423	2.529290923	0.140823569
C	-5.7045327283	-0.9407188961	-0.2364900515
C	-7.0446117037	-0.612337583	-0.4182077905
C	-7.5137842654	0.6484941441	-0.054287541
C	-6.6422832459	1.5840735163	0.5000609934
C	-5.3027698862	1.2629265852	0.6986236886
H	-5.325232633	-1.915709541	-0.5236043502
H	-7.720731898	-1.3423094875	-0.8509459882
H	-8.5585719983	0.9017373348	-0.2024825441
H	-7.0052168507	2.5658814633	0.7858381737
H	-4.6165585232	1.9785735522	1.1381457047

11pP

N	2.2092473462	2.0255847954	0.8851171694
C	1.5376780352	1.2580790009	0.0277520067
N	0.2701372493	1.692285313	-0.1469290178
C	-0.0120205495	2.7259162554	0.8598714265
C	1.4022065525	3.1956954365	1.235286248
N	2.0683309337	0.2104945368	-0.5874130047
C	1.3913891288	-0.5227353594	-1.6437223436
C	0.4597043681	-1.6097285051	-1.147302393
O	-0.6110914101	-1.0509791739	-0.3677075634
C	-1.3050602221	-2.2751566186	-0.0839785882
C	-1.5725452393	-2.8320171976	-1.4918250463
C	-0.3076962883	-2.3576106923	-2.2547801822
C	1.0636663741	-2.6120071592	-0.1238982669
C	-0.1879838411	-3.0793131898	0.6341492827
C	-0.243381394	-4.5772476614	0.4265977054
O	-1.0732758788	-5.3441814802	0.8513755075
C	1.6582646537	-3.8621737213	-0.7310735984
O	2.6540261321	-3.9403911749	-1.4112506108
N	0.8377590532	-4.9444585853	-0.3887600849
C	1.0713698183	-6.2805985365	-0.8286426062
H	3.0733706807	0.0196385737	-0.3955893532
H	-0.5276279172	2.2848273715	1.7184412998
H	-0.6220895124	3.5256373856	0.4412545366
H	1.7082143833	4.0608630487	0.6383714287
H	1.4944092544	3.4387739729	2.2937815531
H	0.8220971586	0.1688820094	-2.2752593886
H	2.1611124062	-0.9863229389	-2.2656133725
H	-2.1842664556	-2.0776409028	0.5260844154

H	0.2698706506	-3.1715232608	-2.6988743612
H	-2.4743869423	-2.3753663316	-1.9022532271
H	-0.44048093	0.9994428215	-0.3640004141
H	-0.5656382721	-1.6560552549	-3.0519323872
H	-1.7081075853	-3.9159939803	-1.5041121547
H	-0.170083495	-2.8561776068	1.7019151936
H	1.8085535542	-2.1168766183	0.5032151343
H	3.2500309956	2.0025818569	0.913514947
C	1.0625740258	-7.3214837304	0.098544034
C	1.2807741207	-8.6244095518	-0.3392132701
C	1.5184148812	-8.8825404388	-1.6878853746
C	1.5320905265	-7.8333684656	-2.6049641142
C	1.302309431	-6.5278650315	-2.1810424714
H	0.8836768634	-7.1094564436	1.1466249319
H	1.2704245911	-9.4381764246	0.3785879176
H	1.6926072841	-9.8997367567	-2.0235692205
H	1.7158372263	-8.0296497834	-3.6562899476
H	1.3041435185	-5.7056869399	-2.888471882
O	4.7200611853	-0.229338954	-0.0481586199
C	5.3710894046	0.7216668058	0.4605410554
O	4.9495748878	1.8146118143	0.8826970086
C	6.8926684197	0.5215026757	0.5665314191
C	7.7010705536	1.571101498	-0.2188570232
C	8.8047678633	0.7622997694	-0.9062163815
C	8.0883665531	-0.5262096436	-1.2801840382
N	7.2535623328	-0.8028095923	-0.0615112696
H	7.0595849785	2.0464997943	-0.9664285419
H	8.0845950416	2.346942977	0.4434305165
H	9.6209966333	0.5467113841	-0.2096980946
H	9.2171862293	1.2626529959	-1.783429659
H	8.7280175305	-1.3843196878	-1.4787848777
H	7.3956822066	-0.3730804609	-2.1097099633
H	7.7663820226	-1.3935495659	0.5974668535
H	6.358992982	-1.2666364375	-0.290631072
H	7.1818699112	0.4830857654	1.6172821949

12p

C	-2.8742214413	-1.6832501354	-1.7538042575
C	-2.2246234591	-2.2667855715	-0.4844005465
O	-0.8353715289	-2.3692633068	-0.8423895767
C	-0.6474273903	-1.0131586009	-1.2428814945
C	-1.7047699757	-0.8617579333	-2.349506321
C	-2.1354038288	-1.2159118748	0.6351251547
C	-1.0875158857	-0.2318984894	0.0448370396
C	-1.4908674096	1.1700960683	-0.3250651784
O	-2.7913098747	1.3453412916	-0.510077307
C	-3.200944916	2.6523099619	-0.9448646947
C	-3.4334546482	-0.631100099	1.1318425513
O	-3.2004752052	0.3125964388	2.052481714
C	0.7748699684	-0.7675465704	-1.7019009326
N	1.7729526191	-1.0242435126	-0.6747836837
C	2.5232143049	-0.09693882	-0.0555670412
N	3.6576152123	-0.4607954072	0.5461576358
C	4.2905778378	-1.7868760031	0.4920811209

N	2.1414683908	1.1832813299	-0.0529786255
O	-0.6651298807	2.045132149	-0.518420773
O	-4.5387239738	-0.992657801	0.8044014461
H	2.000358455	-1.9951384708	-0.5067352029
H	4.0871033624	0.210373136	1.1720236894
C	5.7984309271	-1.5885668628	0.571950972
H	4.0454385867	-2.2213844136	-0.4822671656
H	0.8738731512	0.2598193158	-2.0621998533
H	0.9808293994	-1.4300461445	-2.5474561672
H	-2.6001441098	-3.2371041729	-0.1630910427
H	-4.2800287845	2.5945743134	-1.0716626132
H	-2.716502601	2.9020117809	-1.8906412211
H	-2.9414036274	3.3956064286	-0.1891476848
H	-1.9621114928	0.1741971101	-2.5795825962
H	-3.1828723592	-2.4840553587	-2.4276452123
H	1.1807285323	1.4067562359	-0.3146682799
C	-4.3618483206	1.0025169436	2.5239396359
H	-4.0026371362	1.7392018005	3.2407374666
H	-4.867839214	1.4984285263	1.691694778
H	-5.0531480132	0.3092980727	3.0070921677
C	2.9500008161	2.3037030355	0.4597353179
C	3.7764529912	-2.6872852845	1.6118860549
C	2.0301915737	3.5096236848	0.5940506292
H	3.3135962826	2.0436575314	1.4619940231
C	4.1297535607	2.5980908543	-0.4617263639
H	4.2514659796	-3.6698020653	1.5487989675
H	4.0163329909	-2.2477037799	2.5850608327
H	2.6927060245	-2.8259740835	1.5546404915
H	6.3019966253	-2.5566866509	0.524468875
H	6.1567115695	-0.9664342113	-0.2520265772
H	6.0694801512	-1.1101558153	1.519249845
H	2.5948107356	4.3545409979	0.993903329
H	1.6272914926	3.7934381299	-0.3832057667
H	1.1948837937	3.296751317	1.26591007
H	4.7177462984	3.4243927338	-0.0536561312
H	4.7892076393	1.7328988786	-0.5708937328
H	3.7642424991	2.8850043899	-1.4521738311
H	-3.7458386496	-1.074141137	-1.5134029017
H	-1.3175367392	-1.3236668613	-3.2614200295
H	-0.2337439183	-0.1378882457	0.7188118709
H	-1.6736573877	-1.6921027728	1.506073127

12pP

C	-2.3710813385	-0.4689234114	2.004600368
C	-2.9371592708	0.8545552712	1.4554222715
O	-1.8701810779	1.3414702979	0.6224130421
C	-1.7167586523	0.1711036355	-0.1939679398
C	-1.4139533265	-0.8980176624	0.8644773121
C	-4.010116251	0.6117996176	0.3815382853
C	-3.1626932634	-0.0025640305	-0.7725400386
C	-3.4588411471	-1.4259523079	-1.1882424115
O	-3.8962768263	-2.1906190165	-0.1802580631
C	-4.2204672745	-3.5428827248	-0.5234843785
C	-5.2389881441	-0.1441987345	0.8175634289

O	-6.0328560376	-0.4046309926	-0.2293847436
C	-0.6751933865	0.3987004693	-1.2740002854
N	0.6763760922	0.5550067632	-0.7722627856
C	1.2491157337	1.7243725049	-0.4631309955
N	2.5882410238	1.7683241624	-0.4346789192
C	3.4084955264	2.5421254709	0.5090811002
N	0.4910492043	2.800382236	-0.2128547947
O	-3.2799994654	-1.8425500833	-2.3089527332
O	-5.5181766749	-0.4268845719	1.9586783086
H	1.263740954	-0.2953404798	-0.64776879
H	3.0607072943	0.9303530182	-0.8077914346
C	4.009634902	1.5859634326	1.5387063291
H	2.7528145465	3.2504272276	1.0205720988
H	-0.6851814717	-0.4656763553	-1.9449510067
H	-0.9577647356	1.2797549085	-1.8612141705
H	-3.2020554767	1.6033674835	2.2002136536
H	-4.5666294539	-4.0085138607	0.3975980005
H	-3.3376637976	-4.0609920584	-0.9034694388
H	-5.0076249903	-3.5602408249	-1.2805289452
H	-1.5759203017	-1.9177606206	0.5125706125
H	-1.8304209286	-0.293497186	2.936287004
H	-0.4858832698	2.6104252636	-0.0003062243
C	-7.1894158817	-1.1935215203	0.0664665358
H	-7.683098865	-1.3710947609	-0.8875950347
H	-6.8939188495	-2.1406243352	0.5245959671
H	-7.8573034148	-0.6579447592	0.7443784169
C	0.8308289234	4.2057054818	-0.4727043073
C	4.4980919198	3.2924889609	-0.2484845516
C	0.5865175334	5.0396935062	0.77978278
H	1.8873808529	4.2391297036	-0.7461514631
C	-0.0038220512	4.7109682456	-1.6471604758
H	5.1182212227	3.8649889029	0.446456106
H	5.1427659186	2.5815701976	-0.7765800472
H	4.074558038	3.9797958511	-0.9858300809
H	4.632800037	2.1355476965	2.2497294346
H	3.2258201185	1.0626551218	2.0935215929
H	4.6320549599	0.8398934196	1.0332396055
H	0.8422117123	6.0856313869	0.5917464179
H	-0.4713805102	4.9928047728	1.0603816264
H	1.1797509642	4.6809173059	1.6249278826
H	0.2261048241	5.7605482215	-1.8502265498
H	0.1969862871	4.1296585948	-2.5509504211
H	-1.0712972255	4.6345593087	-1.4130904187
H	-3.1643789382	-1.1927956583	2.1916764617
H	-0.3683011421	-0.7995597457	1.1656379244
H	-3.2521510395	0.596926723	-1.6807218024
H	-4.3695786082	1.5876374335	0.0390478307
O	4.0201689343	-0.5656659359	-1.1496332688
C	3.4363831518	-1.5461616439	-0.619556349
O	2.2397352792	-1.6469375293	-0.2824643101
C	4.3038235914	-2.7816424393	-0.3235704794
C	4.3779346554	-3.1243341048	1.1745389903
C	5.8398250296	-3.5203440262	1.3949152278
C	6.5870198317	-2.5362665279	0.5072518319

N	5.727156489	-2.4788674392	-0.7253256159
H	4.1321046549	-2.2383394994	1.7682497716
H	3.6720482152	-3.9138500178	1.4313893194
H	6.0200965472	-4.5441583446	1.052630876
H	6.1531022668	-3.4394100329	2.4366839087
H	7.5978778059	-2.8260124604	0.2257804475
H	6.5915291509	-1.5337190453	0.9385547208
H	6.0600706523	-3.1432475641	-1.4277992033
H	5.7034251237	-1.5365936554	-1.1518001355
H	3.9590421197	-3.6272616305	-0.9193658783

11pEn

N	-3.7231529478	-3.1640016421	0.8090927845
C	-2.6050001632	-3.0422528093	0.0807468088
N	-1.9116776525	-4.1898003688	0.062431173
C	-2.5102319878	-5.1357449428	1.0152340742
C	-3.9300296619	-4.5713258268	1.1702364689
N	-2.2443607742	-1.9277793073	-0.5383981
C	-1.1233824907	-1.8413682249	-1.464949081
C	0.2176321954	-1.5958749136	-0.8029177656
O	0.5325984913	-2.6917460684	0.0719324741
C	1.8287246685	-2.2469586953	0.4923269547
C	2.5649754644	-2.0775262768	-0.8464376325
C	1.4159074374	-1.5986267143	-1.7724252694
C	0.3303201004	-0.3957421842	0.1890170623
C	1.4676576889	-0.8722266557	1.1125982035
C	2.5522445433	0.172375931	0.9925452404
O	3.6204218003	0.1929797663	1.5511561904
C	0.829108043	0.9116416753	-0.3964725207
O	0.2452515095	1.6646796107	-1.1524800284
N	2.1174283234	1.1438464615	0.0731238623
C	2.8795428453	2.3023082472	-0.2644750271
H	-2.8944511391	-1.1441047056	-0.4845197936
H	-1.9622383814	-5.1103320472	1.9612855252
H	-2.504591394	-6.1487209336	0.6154308807
H	-4.6288730191	-5.0416488756	0.472821259
H	-4.3085904796	-4.6630649055	2.1873219845
H	-1.0572229511	-2.7704485406	-2.0419485226
H	-1.3363525544	-1.0270532636	-2.1580771612
H	2.2652151327	-2.9574204927	1.1912879541
H	1.5949290917	-0.6214038593	-2.2271592457
H	2.9553028424	-3.0425292452	-1.1728530445
H	-0.903923787	-4.1486595228	-0.060124171
H	1.2316778194	-2.3137061573	-2.5775225278
H	3.4023313936	-1.3782754331	-0.7919486462
H	1.1718236059	-0.976415216	2.1581017665
H	-0.6189665972	-0.22030284	0.7031871169
H	-4.501029709	-2.5285586168	0.6792394108
C	3.4157212984	3.0850651191	0.7572336638
C	4.1455086362	4.223236543	0.4269268406
C	4.328531952	4.5764599352	-0.9089009451
C	3.785047925	3.7867746335	-1.9203052181
C	3.0602897542	2.6415096034	-1.6031709524
H	3.2625570998	2.8000594727	1.792902697

H	4.567383601	4.8359116281	1.2169990977
H	4.8951182296	5.4668486315	-1.1615094546
H	3.9273594484	4.058799327	-2.9609459141
H	2.6351257064	2.0173683668	-2.3819937637
O	-2.314990426	0.8874211484	-1.019584049
C	-3.3882734015	1.1988401151	-0.3003213003
O	-4.1717583168	0.3260665592	0.0160432242
C	-3.5948918736	2.6719522364	-0.0088089225
C	-4.271810489	3.3457799781	-1.2138206542
C	-3.8487013875	4.8024692611	-1.0388695818
C	-2.4095842639	4.7101636356	-0.521017545
N	-2.3294470225	3.3735403799	0.109467772
H	-3.8591803675	2.9353723481	-2.1419505893
H	-5.3516403165	3.1924414645	-1.2111877566
H	-4.4820387217	5.2787239433	-0.2847525832
H	-3.9214562305	5.3795245083	-1.9623067248
H	-2.2071430496	5.5059964129	0.1991391285
H	-1.676031009	4.7872524772	-1.3309111026
C	-1.516346757	3.1255007182	1.2270191385
H	-4.2188624591	2.7486202339	0.8925791262
C	-0.2457033935	3.944141174	1.3043545365
C	0.4334764562	3.8306521451	2.6681763636
H	0.4316145371	3.6085791679	0.5057437763
H	-0.4616354228	4.9954958004	1.0958709268
C	0.5187752436	2.3723361752	3.104426936
H	1.4268376169	4.2875746913	2.6177617521
H	-0.144803349	4.3948267717	3.4109823626
C	-0.8841373908	1.7930031506	3.2732205916
H	1.0515248645	1.8090300545	2.3279339651
H	1.0909260511	2.2677558527	4.0315365776
C	-1.7968087201	2.1626336529	2.1296776033
H	-0.8323281869	0.7003210092	3.3626619719
H	-1.3141433477	2.1477195759	4.2211623101
H	-2.7288405145	1.6069200466	2.068215106
H	-1.6590593101	1.6182106439	-1.0562770681

11pEn'

N	-2.5231105336	3.7649344354	-1.5911964142
C	-1.5850334227	3.66557616	-0.6402858681
N	-0.9920923458	4.8485732951	-0.4151179536
C	-1.4289515957	5.8118520078	-1.4360449548
C	-2.7365530437	5.1765170588	-1.9310293417
N	-1.2940716433	2.5408164269	-0.0032406257
C	-0.390548167	2.4690492676	1.1373053521
C	1.0711335254	2.3437799794	0.7613383554
O	1.5093738943	3.5028444952	0.0334137407
C	2.8810982631	3.1152003057	-0.1415854161
C	3.3402202989	2.8782795677	1.3069145711
C	2.0424145873	2.330257466	1.9569905395
C	1.4291476898	1.2173672762	-0.2485148675
C	2.7028658245	1.7743987454	-0.9025969284
C	3.7739684723	0.7394591564	-0.6357062438
O	4.9288656101	0.7750534611	-0.9852497707
C	1.8405268045	-0.0947259332	0.3815472351

O	1.140516518	-0.8527615202	1.0097823997
N	3.2054137116	-0.2861923408	0.1335933833
C	3.9440300114	-1.4054950726	0.6184467402
H	-1.8360059546	1.7084685456	-0.2501771968
H	-0.6844165891	5.8731829913	-2.2347948777
H	-1.5822397916	6.7988020436	-1.0017942392
H	-3.6036058358	5.5677824184	-1.3911683767
H	-2.8845142799	5.3037732436	-3.0026505417
H	-0.5214927058	3.3562838432	1.7663835694
H	-0.6778834597	1.594241417	1.7246984812
H	3.421863847	3.8810482908	-0.6937922028
H	2.1553369405	1.3392134712	2.4020801568
H	3.6360616126	3.826373993	1.7582801211
H	-0.0322899438	4.8565920493	-0.0812667856
H	1.6713938634	3.0057874312	2.7315715772
H	4.1869311021	2.1919274944	1.3810687645
H	2.6130909083	1.9442811781	-1.9764327445
H	0.6067629048	1.0467125887	-0.9470317866
H	-3.2665439766	3.0766195795	-1.6631631508
C	4.7223227992	-2.1512581109	-0.2650374944
C	5.4472291341	-3.2368459382	0.217608644
C	5.3842122444	-3.5800805533	1.5668233737
C	4.5980452726	-2.8305406153	2.4399201694
C	3.8787287633	-1.7352846112	1.9710531397
H	4.757936844	-1.8796227733	-1.3141397791
H	6.0573587365	-3.8187202641	-0.4655150769
H	5.9470705091	-4.4306789357	1.937483638
H	4.5472157815	-3.0938653478	3.4913924685
H	3.2697066605	-1.1406450049	2.643569065
O	-4.4391142338	1.3904294619	-1.5847783927
C	-3.898422693	0.2333439849	-1.2009704184
O	-2.7966717112	0.1915070459	-0.697360111
C	-4.7288541276	-0.9932396789	-1.5270158378
C	-4.3941960149	-1.50243754	-2.9380576855
C	-5.6630312753	-2.2757974536	-3.2942909369
C	-6.7946568969	-1.4384044346	-2.6854606153
N	-6.1355814424	-0.6330577289	-1.6249807063
H	-4.258564044	-0.6513073048	-3.6143712367
H	-3.4869023923	-2.1072848245	-2.9467556289
H	-5.6343068982	-3.2602679717	-2.8188597918
H	-5.7840812081	-2.4178921998	-4.3694498642
H	-7.5739744654	-2.0781089556	-2.2635322507
H	-7.2567771274	-0.7746681282	-3.4224817664
C	-6.8387489807	-0.3205080507	-0.436396787
H	-4.5166518323	-1.7635012887	-0.774443457
C	-8.2653971446	0.1290101856	-0.6557803762
C	-9.0383067731	0.2701809475	0.6552156903
H	-8.2422282539	1.0887636905	-1.1913733943
H	-8.7831761364	-0.5733495327	-1.3164904479
C	-8.2020845628	1.0114203604	1.6938283755
H	-9.9837846424	0.7874219308	0.4670784403
H	-9.2861935964	-0.7271036647	1.0398350629
C	-6.9443813983	0.2069879601	2.018182239
H	-7.9150817851	1.9909906558	1.2900388922

H	-8.7811079594	1.1923499915	2.6044993503
C	-6.2544304976	-0.2928544303	0.7733384634
H	-6.2434599339	0.8166064734	2.6000666612
H	-7.2075881012	-0.6437508588	2.6627665456
H	-5.2264822128	-0.6247401248	0.8950840493
H	-5.3439715052	1.1802688214	-1.911187913

12pEn

N	3.219161716	-1.8814284992	-0.1356226746
C	2.9547712893	-0.6127327169	-0.4831846383
N	3.7052155063	0.4186239925	-0.0953291502
C	5.1432045445	0.4096018929	0.2187603611
H	5.4767813375	-0.6298456202	0.1831017914
C	3.7575105019	-2.3448959756	1.1568422055
N	1.8837244363	-0.4016329038	-1.2610742955
C	1.626240725	0.7601447773	-2.0934223789
C	0.8382622722	1.8603770411	-1.4077649997
O	1.6288518208	2.4535322026	-0.3683195711
C	0.6702674637	3.4335362743	0.0680134951
C	0.322594207	4.18016074	-1.2330655445
C	0.5477135848	3.0769847911	-2.2971318331
C	-0.4390665625	1.4106860049	-0.6098000079
C	-0.4657914295	2.4988374303	0.5102501553
C	-1.7901114974	3.1517178694	0.8142690142
O	-2.7410362711	2.2273629936	1.0020387812
C	-4.0577793306	2.7327118277	1.2456060926
C	-1.7002944823	1.2774101706	-1.4352995873
O	-2.1573812577	0.2197154289	-1.823897381
O	-2.2720732643	2.4455759958	-1.7132424112
C	-3.5259588328	2.3845735621	-2.4096817779
O	-1.9709389955	4.3394534123	0.9376041458
H	2.6456541631	-2.5749560984	-0.6145025191
H	1.2386642438	-1.1749892837	-1.3778028864
C	5.3768461101	0.9965077708	1.6056938512
C	5.8816422627	1.2040573196	-0.8549256343
H	2.5731173403	1.1816366109	-2.4483237603
H	1.0651571435	0.4158376571	-2.9667170595
H	1.0921136124	4.0401187018	0.8675942729
H	-4.3503262699	3.4250088507	0.4525755047
H	-4.0983693266	3.2473446552	2.208297494
H	-4.7143329793	1.863513098	1.2533188524
H	-3.8653778179	3.4151036873	-2.494625978
H	-4.2406020497	1.7883987345	-1.8382638627
H	-3.3878997184	1.9443223109	-3.3987612487
H	1.4400036688	3.2812293192	-2.8951883261
H	1.000140391	5.0225578921	-1.3807402065
H	3.2783810155	1.3374354249	-0.2012278075
H	-0.2954251269	2.9346574231	-2.9725173424
H	-0.7012704517	4.5542692361	-1.2172571493
H	-0.1548406936	2.0155456563	1.4425889665
H	-0.2585038416	0.4252653785	-0.1726782053
C	2.6197451496	-2.9324866817	1.9881861681
C	4.8627521468	-3.363024016	0.9089570042
H	4.1627745729	-1.4741747747	1.6748817437

H	5.2729547281	-3.7118907235	1.8599878226
H	4.4631001346	-4.2288908929	0.3702957516
H	5.6728168734	-2.9318672535	0.3146024593
H	3.0097717052	-3.3262112388	2.9307835695
H	1.867223958	-2.1721825853	2.2168184235
H	2.1338005253	-3.7513034341	1.446766144
H	6.4436343254	0.9838260788	1.8427942544
H	5.0329821488	2.0359464578	1.6341277684
H	4.8403364147	0.4385285217	2.3774792918
H	6.953682661	1.2150960969	-0.6410169733
H	5.7272141469	0.7646481066	-1.8437610128
H	5.5259690485	2.2398809629	-0.8724234427
O	-0.4886498705	-1.8813889614	-1.7095374693
C	-0.362350101	-3.0201957998	-1.0248407719
O	0.7281600123	-3.539652587	-0.9150254538
C	-1.6512825677	-3.6072370949	-0.4870797768
C	-2.3772777938	-4.3336449065	-1.6355793302
C	-3.8368624438	-4.2674860747	-1.1955816726
C	-3.9560136318	-2.8856765701	-0.5501949738
N	-2.5946040761	-2.565836328	-0.0895856013
H	-2.2378735485	-3.7755218461	-2.567778534
H	-1.9960399594	-5.3460722718	-1.7739147099
H	-4.0308405916	-5.0413251359	-0.4469682793
H	-4.534292131	-4.4067330206	-2.0234348622
H	-4.6462804224	-2.9042296028	0.2989113877
H	-4.3073434186	-2.1232066605	-1.2552304152
C	-2.4215274683	-1.8547667827	1.1131924902
H	-1.3929266499	-4.3051891697	0.3178347141
C	-3.415113446	-1.1507570692	1.6898495919
C	-3.2858611109	-0.4462974577	3.0168681669
H	-4.3809103193	-1.0831848542	1.1982378224
C	-2.0404898562	-0.8910456848	3.7789088223
H	-3.2451529968	0.6415923827	2.8662060784
H	-4.185047921	-0.6381233525	3.6146062561
C	-0.8459347057	-0.8720453705	2.8324075122
H	-1.8661213925	-0.2396194626	4.6407687444
H	-2.1821753575	-1.9100937031	4.1614516183
C	-1.0287582469	-1.8946095847	1.7113355594
H	-0.7639141506	0.130314279	2.3912076587
H	0.0900676523	-1.0736120622	3.3624110222
H	-0.810857809	-2.9058606082	2.0818543148
H	-0.2833940226	-1.6890767533	0.9326135362
H	-1.3599643514	-1.4383215468	-1.5742851647

12pEn'

C	-4.0718814196	-0.5949808002	1.5766081124
C	-4.7306591696	0.3984772302	0.600943043
O	-3.601129633	1.0379066988	-0.0219901477
C	-2.9294589303	-0.1504562097	-0.4653616062
C	-2.7320733964	-0.8987560703	0.8598289196
C	-5.3236562895	-0.3164567955	-0.6238612138
C	-4.0383653594	-0.8321917437	-1.3377979195
C	-3.849693915	-2.3281691148	-1.4607582081
O	-4.3866794226	-3.0129376908	-0.4439569873

C	-4.2698495869	-4.4385151543	-0.5255807783
C	-6.4151744309	-1.3203953584	-0.3525324693
O	-6.7542328028	-1.9667268889	-1.4752152571
C	-1.691754451	0.2004146499	-1.2710323562
N	-0.6062630358	0.7742487544	-0.4927490983
C	-0.4100991762	2.0883676142	-0.2806171789
N	0.8316496925	2.4931129387	0.0057163242
C	1.2151282088	3.6006933361	0.8968186975
N	-1.4452245384	2.9238044073	-0.3750527678
O	-3.240853655	-2.8498775782	-2.3655077395
O	-6.9651183123	-1.4850927918	0.7105643663
H	0.0928855809	0.146466337	-0.1110448079
H	1.5676307263	1.8027655812	-0.1549512411
C	1.8462455055	3.021522029	2.1617156923
H	0.301199762	4.134502196	1.1656161724
H	-1.3146700892	-0.7107171093	-1.7430130805
H	-1.9736010631	0.8972573182	-2.0674951268
H	-5.3820861321	1.1459787312	1.0505449263
H	-4.767736981	-4.8283741417	0.3604464412
H	-3.2187486286	-4.7339055408	-0.5331762204
H	-4.7582313381	-4.8041105623	-1.4314811501
H	-2.5303298742	-1.9633306232	0.7341597816
H	-3.9078782389	-0.1233341539	2.5469026642
H	-2.3661246497	2.4898001973	-0.3260037502
C	-7.7317774747	-2.9993107846	-1.3121380399
H	-7.8425892702	-3.4640203206	-2.2905883491
H	-7.3885316153	-3.7329049512	-0.5785770379
H	-8.6839456233	-2.5782329216	-0.9828729368
C	-1.4277292748	4.3399329973	-0.7714917067
C	2.1809613975	4.5365586569	0.1794779159
C	-2.1311829596	5.1819537293	0.2864434757
H	-0.3826486748	4.6413350626	-0.8616104915
C	-2.1046893322	4.4736312107	-2.1330123633
H	2.4681793363	5.3582511262	0.8406991384
H	3.0881997303	3.9917978927	-0.103008379
H	1.7393098898	4.9566380052	-0.7278751748
H	2.1130976	3.8316439134	2.8461770114
H	1.1609352297	2.3400988924	2.6734924336
H	2.7595638293	2.4715820988	1.9090219322
H	-2.110790334	6.236688824	0.0009738048
H	-3.1781253744	4.8726478574	0.3762283862
H	-1.6586858886	5.0748150152	1.2663493035
H	-2.1226479923	5.5221545495	-2.4421239171
H	-1.5742276625	3.8947625152	-2.8935479656
H	-3.138342841	4.1149494957	-2.0781762314
H	-4.6905469023	-1.4811758679	1.7183315812
H	-1.8907765942	-0.4471241472	1.3912901395
H	-3.9769816691	-0.4402526467	-2.354988705
H	-5.7748787822	0.4425559442	-1.2711849347
O	3.0556586237	0.5216457733	-0.0395583052
C	2.7826662288	-0.3068844644	0.8009285923
O	1.5274113571	-0.452904836	1.2378076294
C	3.7646015587	-1.3128207423	1.3733448263
C	3.7583645572	-2.6022137827	0.536720072

C	4.2727328632	-3.6270760025	1.5472343551
C	3.6339708395	-3.1966607764	2.8735808823
N	3.3036558054	-1.7614478577	2.6792636639
H	2.732494684	-2.8405730613	0.2345039524
H	4.3763751852	-2.51051256	-0.3570174102
H	5.3614171325	-3.551726232	1.619104681
H	4.017346834	-4.6529955268	1.2768250701
H	4.3282226994	-3.3286898199	3.7076337918
H	2.722701281	-3.7614757566	3.0925218557
C	3.3876213342	-0.8656781655	3.772263661
H	4.7639415234	-0.8598853955	1.3812316147
C	2.7516476771	-1.3780067996	5.0443582314
C	3.0264451833	-0.4677573437	6.2411454543
H	1.6696415481	-1.4661772482	4.8707844091
H	3.1068910385	-2.3909765935	5.2598737206
C	2.8171366842	0.9956674833	5.8634564854
H	2.3801807881	-0.7568583393	7.0751959226
H	4.0631960229	-0.6066841079	6.5725051734
C	3.811813767	1.3977341242	4.7759969447
H	1.7935086795	1.1275092503	5.4887077619
H	2.9291824449	1.6439778	6.7378030759
C	3.8700267686	0.3836357116	3.6608425861
H	3.5474057636	2.3776420481	4.3616352755
H	4.8110908088	1.5190779302	5.2181344093
H	4.3260959184	0.7149126572	2.7310540935
H	1.5598526966	-1.1305192049	1.951360598

Ia

C	3.8616413569	2.1837490074	-1.9904816431
C	3.1992171767	2.3037309366	-0.6043291104
O	1.8670368367	1.818553922	-0.8287601662
C	2.2260092327	0.5636219263	-1.3992649448
C	3.0867813257	0.990218763	-2.5944662524
C	3.7061536947	1.2259221648	0.3744179952
C	3.0928416077	-0.076389876	-0.246155852
C	4.1590496444	-1.0158722718	-0.8164248089
O	3.7857975506	-2.2895260037	-0.783249411
C	4.727076614	-3.2181468786	-1.3480586245
C	5.1769050798	1.1992157648	0.7331991756
O	5.8073233076	2.3041522958	0.33952234
C	1.0159257533	-0.2638219096	-1.735091235
N	0.2906926726	-0.584820084	-0.522349684
C	0.9168592992	-0.9114624085	0.6111165371
N	0.191996734	-1.366733019	1.6318960204
C	0.696113502	-1.6382681813	2.9777767751
C	2.0651623001	-1.0053681749	3.1493962918
C	2.905852056	-1.3238977713	1.9239143657
N	2.2503557619	-0.7848514313	0.7194084779
O	5.196483622	-0.6191207361	-1.2924911409
O	5.7006972273	0.3241660524	1.3848506214
H	-0.7230593995	-0.5515319066	-0.5265255786
H	-0.7857897211	-1.5889014347	1.4377803039
H	0.7576972327	-2.7217860236	3.1212259484
H	-0.023147696	-1.2369747579	3.6948531744

H	3.8957934839	-0.8691358882	1.989290365
H	3.0322377919	-2.4053001219	1.8048272553
H	1.3334368146	-1.1714170835	-2.2626943106
H	0.3546157696	0.3058195956	-2.3895540273
H	2.5440340761	-1.4041340083	4.0456403169
H	1.9763816264	0.0800428937	3.2595823339
H	3.1668712433	3.3047911776	-0.1774057234
H	4.2744949705	-4.201039336	-1.2343541408
H	5.6737577412	-3.1657718266	-0.8077149459
H	4.892145806	-2.9906941494	-2.4025984834
H	3.7226804118	0.2032603874	-2.9965375868
H	3.7046754444	3.0974867529	-2.5656661461
H	3.1879431015	1.3871454197	1.326749538
C	7.1936916609	2.3845304874	0.701403788
H	7.5490932588	3.3317894914	0.300368482
H	7.3034800049	2.3636126577	1.7871326055
H	7.7452189918	1.5517530686	0.2612936862
O	-2.6064454393	-1.9837607466	1.10361291
C	-3.2776138471	-1.3243945865	0.3381515991
O	-2.7276384749	-0.447320391	-0.4970773952
C	-4.7937135316	-1.4473068884	0.2159210482
N	-5.2986694075	-0.3870422051	-0.6794910256
C	-5.9434639327	-1.0047861591	-1.8514779333
C	-5.2662754449	-2.3649599995	-1.9527321441
C	-5.1603248312	-2.7725263463	-0.4829606245
C	-5.9005785885	0.7644816464	-0.1048359834
H	-7.0217531014	-1.1237308446	-1.6734747618
H	-5.8056909488	-0.3739789555	-2.733052994
H	-5.844345686	-3.0719121264	-2.5495951177
H	-4.2717126747	-2.2655673616	-2.4012445321
H	-6.1341173066	-3.103685031	-0.1133952272
H	-4.4286756448	-3.5572022573	-0.2826331724
H	-5.2078342333	-1.397453016	1.2250252137
C	-6.9088442306	1.4211591551	-0.6983429174
C	-7.5217801766	2.681351937	-0.1425162664
H	-7.31145001	1.0614168745	-1.640356386
C	-7.1102405142	2.9392997876	1.3060226649
H	-7.2286211313	3.5372679581	-0.767391332
H	-8.6132561953	2.6167306398	-0.2202392379
C	-5.6101913346	2.7112528855	1.4703481681
H	-7.6504586171	2.2512643048	1.9687642791
H	-7.3844202813	3.9565801745	1.6013460408
C	-5.2624779431	1.251686088	1.1792392215
H	-5.2800808501	2.9718532495	2.4802170751
H	-5.0674573435	3.3605519684	0.7719542713
H	-5.5904869486	0.6231771344	2.01750896
H	-4.1738384758	1.1344984089	1.1129121169
H	-3.5022130533	-0.0417229809	-0.9741220412
H	4.9317122971	1.9936530469	-1.9160640904
H	2.4019679323	1.3181971541	-3.3804489681

Ib

C	3.7839536005	2.4041700362	-1.7038401591
C	3.1758903282	2.3473592494	-0.2890412319

O	1.8265321121	1.9182709022	-0.5235748181
C	2.1367299699	0.7461155763	-1.2707856898
C	2.9602871452	1.3171821693	-2.4317599033
C	3.694715294	1.1398641868	0.5169627367
C	3.0368194297	-0.0570322938	-0.2528884779
C	4.0647316125	-0.9157635208	-0.995236411
O	3.652386683	-2.1660635489	-1.1672951779
C	4.5499376674	-3.0092830632	-1.909721586
C	5.1754787961	1.0435258048	0.8191992582
O	5.8191759878	2.1750709131	0.538966667
C	0.8957906813	-0.0077452254	-1.6628439973
N	0.2168848609	-0.4769534522	-0.471542679
C	0.8839337561	-0.9834009451	0.5687847983
N	0.1968589077	-1.5818921942	1.5401475997
C	0.7541264405	-2.0613762426	2.8049509894
C	2.1383590548	-1.4743581534	3.0104686036
C	2.9211775026	-1.608843825	1.7149516735
N	2.2233133545	-0.8884772896	0.6358025363
O	5.1039435775	-0.4731630154	-1.4247201355
O	5.6991452891	0.0874333027	1.3449829919
H	-0.7973212427	-0.5013036263	-0.4594855089
H	-0.8027507116	-1.7217346436	1.3834182515
H	0.8043840597	-3.1545913127	2.7785442447
H	0.0713385867	-1.7680730592	3.6050982391
H	3.9190218345	-1.1764079312	1.8053892025
H	3.0264111723	-2.6608779036	1.4282939789
H	1.1679946556	-0.840068078	-2.3233078668
H	0.2215386887	0.6593233838	-2.2018975923
H	2.6491111614	-2.0096015023	3.8130485418
H	2.0706408302	-0.4178072582	3.2886527705
H	3.1797566444	3.2826008713	0.2683646733
H	4.072526314	-3.9861784633	-1.9468852895
H	5.5126821524	-3.0709403825	-1.3997803228
H	4.6913010606	-2.6120160137	-2.9164483135
H	3.5629760904	0.5822877846	-2.9633009811
H	3.6268605574	3.3898001402	-2.1446088652
H	3.2108059465	1.178189521	1.4995332947
C	7.2168292629	2.1842418213	0.8643133077
H	7.5828264669	3.1653776315	0.5679368799
H	7.3572662454	2.0315749405	1.9359985209
H	7.7357960877	1.3991870294	0.311484957
O	-2.6779589316	-1.9441901129	1.2384356548
C	-3.3532260649	-1.3788191805	0.4050581402
O	-2.7996996513	-0.6281640051	-0.5448772321
C	-4.8687482009	-1.497088214	0.31665613
N	-5.3855611456	-0.445018519	-0.5590372284
C	-6.3025159898	-1.0427578743	-1.5496847851
C	-5.6858507871	-2.4168379078	-1.7699954455
C	-5.28878562	-2.8297783616	-0.3510753908
C	-5.6804511511	0.8176539515	0.0130855517
H	-6.3372731548	-0.4378981863	-2.4565097775
H	-7.3197175865	-1.1312691651	-1.1430549317
H	-4.8014291158	-2.3276008119	-2.4094797445
H	-6.3812410909	-3.1181873085	-2.2335511051

H	-6.156315437	-3.2234066659	0.1833953644
H	-4.4970569788	-3.5805214939	-0.3121473971
H	-5.2609070256	-1.4318192001	1.3369951257
C	-6.4381619594	1.7583871961	-0.8966501214
C	-6.3879886959	3.2060154806	-0.4080112871
H	-7.4803779139	1.4211554406	-0.9766811819
C	-6.6397186258	3.277465352	1.0949601273
H	-7.1196014502	3.8029080209	-0.9604616624
H	-5.3977546463	3.6267237374	-0.6238889756
C	-5.5316517564	2.5350946952	1.8389145442
H	-7.6075538864	2.8117398851	1.3206027216
H	-6.6920297019	4.3170767674	1.4322646069
C	-5.2476868683	1.1846981155	1.2311836289
H	-5.8037139634	2.404802724	2.8926394783
H	-4.6159228924	3.1438219883	1.8389504755
H	-4.6589845746	0.501361021	1.837778368
H	-3.5588731078	-0.2518123112	-1.0570005842
H	-6.0152624794	1.6909728419	-1.9052906286
H	4.8512862264	2.1863483447	-1.6980857222
H	2.2529333122	1.7596514358	-3.1376014265

IIa

C	-4.2027213166	3.4196916284	-0.178854396
C	-4.0009156952	2.3405987785	-1.2604101392
O	-2.7580931609	1.7256193917	-0.8891848997
C	-3.1089152094	1.4066399883	0.4541531526
C	-3.4703972357	2.7834285488	1.0248049256
C	-4.9504651216	1.1415550496	-1.067623178
C	-4.3862886739	0.5026405525	0.2475058018
C	-5.3458846195	0.6429915178	1.4319551837
O	-5.1667458022	-0.3057197253	2.3434437721
C	-5.9900952347	-0.1907719563	3.5166761174
C	-6.4449412534	1.3732045988	-1.145121792
O	-6.7404635245	2.5818521681	-1.6200443357
C	-2.0006216713	0.6801501148	1.1649205136
N	-1.7576317941	-0.5917289344	0.5154729565
C	-2.7560921322	-1.3632739494	0.0796481113
N	-2.4752942462	-2.5997191127	-0.328125699
C	-3.4231928228	-3.5260869043	-0.9455380482
C	-4.6600995325	-2.7698146679	-1.3952613894
C	-5.101885621	-1.8448438785	-0.2732277261
N	-4.0184367854	-0.900316184	0.049653498
O	-6.1277896731	1.5578853739	1.5411346263
O	-7.2761590768	0.5287361243	-0.8989580993
H	-0.8049615715	-0.9393837516	0.4506223789
H	-1.5200399735	-2.9267449284	-0.1686014193
H	-3.6933487724	-4.297063105	-0.2166890671
H	-2.9221940057	-4.0097505268	-1.7866021137
H	-5.9742707736	-1.2564881964	-0.5629739446
H	-5.3554836233	-2.4148883932	0.6272093547
H	-2.2732089359	0.5468932343	2.2191519139
H	-1.0851636964	1.2720782458	1.1151079843
H	-5.4575409424	-3.4768225863	-1.6314591826
H	-4.4449635871	-2.1802595615	-2.2921062293

H	-3.9494154253	2.7002692042	-2.2867429906
H	-5.7296839125	-1.0412283187	4.1432691493
H	-7.0446646225	-0.2264844798	3.2389592143
H	-5.7771161724	0.7467041673	4.0336154156
H	-4.0594312241	2.7536389512	1.9403108029
H	-3.7335876297	4.3558128017	-0.4854473748
H	-4.7348502267	0.4293454771	-1.8722616166
C	-8.1402631181	2.8515044114	-1.7850830208
H	-8.2025969411	3.8691525481	-2.1657388574
H	-8.579638837	2.1496021183	-2.4962231055
H	-8.6537141008	2.7696359502	-0.8253771173
O	0.244661958	-3.4924049722	0.1118321239
C	1.2169205434	-2.7952048647	0.3322846083
O	1.0813680153	-1.4830410465	0.4596862495
C	2.6018637522	-3.4045944856	0.5241246898
N	3.6729076078	-2.4320505529	0.3718066984
C	4.4716270426	-2.3120406118	1.5896871184
C	3.5059462581	-2.7728952661	2.6737919458
C	2.7563300092	-3.9102572743	1.9786505566
C	4.1931077156	-2.0555847844	-0.8638494835
H	5.3627377397	-2.9570142922	1.5430685377
H	4.8031161988	-1.2775469557	1.7184595805
H	4.0155209653	-3.0960322493	3.5829722057
H	2.8211224322	-1.9569299254	2.9294211375
H	3.373351858	-4.8123850928	1.9636634907
H	1.7962238074	-4.1600227333	2.4346363329
H	2.6628350061	-4.2400618119	-0.1793771096
C	5.41312963	-1.4891597082	-0.9851739475
C	5.9554732441	-0.9417779273	-2.2800445692
H	6.0603565956	-1.4186607075	-0.1158307769
C	4.8597464085	-0.7477942155	-3.3261192406
H	6.4663601013	0.0100516825	-2.0855818931
H	6.7270825579	-1.6161021085	-2.6797125504
C	3.9713585998	-1.9866778483	-3.3815168299
H	4.2433368622	0.1209030856	-3.0560391555
H	5.3007305298	-0.5399723466	-4.3058819358
C	3.2583308744	-2.1987896051	-2.046105115
H	3.2284604173	-1.9054108503	-4.1804687512
H	4.5917604695	-2.8636520316	-3.6043414155
H	2.7867017845	-3.187294618	-2.0393898649
H	2.4427148421	-1.4679730895	-1.9377456648
C	3.5774537631	0.7893362012	0.0211008877
C	4.7762173781	1.6240433184	0.2021505482
H	3.2149295898	0.680992793	-1.0151288448
O	2.9780476716	0.2705238712	0.9546116445
C	5.356065177	2.2336172004	-0.9128196343
C	6.4907614918	3.0242103829	-0.7677204561
C	7.0262049017	3.1885538342	0.5057228346
C	6.4603139321	2.5928539514	1.6334638172
C	5.3278321397	1.8089919047	1.4749588888
H	4.917360141	2.0888861439	-1.8969978298
H	6.952256097	3.5034413041	-1.6235766936
Cl	8.4542340749	4.1728337947	0.7012262079
H	6.9028850055	2.7450051548	2.6114780082

H	4.8624206544	1.3343488957	2.3331792515
H	1.9293528385	-0.9790405429	0.6285178697
H	-2.5300488762	3.2987515286	1.2361920135
H	-5.2574615113	3.6032186864	0.022296384

IIb

C	3.6274790212	1.4491951567	-2.0194994187
C	3.1938671719	1.4929198625	-0.5417033461
O	1.8450298207	1.0031285703	-0.5753175969
C	2.1128553868	-0.2178397203	-1.257717449
C	2.7722167945	0.2768621415	-2.5514680732
C	3.8555734437	0.3748070825	0.2881231891
C	3.1548026253	-0.902438553	-0.2899342926
C	4.1141453552	-1.8049079677	-1.0704194877
O	3.7341607744	-3.0781077906	-1.063572461
C	4.5654939806	-3.9676653573	-1.8291889449
C	5.364953121	0.3467677117	0.406679436
O	5.9221254856	1.4744945046	-0.0301626121
C	0.8683046336	-1.040764959	-1.4432217857
N	0.3463702357	-1.438854222	-0.150646907
C	1.1473476722	-1.8236993642	0.842661187
N	0.6020177716	-2.3821584615	1.9272132484
C	1.3053838305	-2.7080342644	3.1689569836
C	2.6653714298	-2.034554929	3.1757171875
C	3.3207400099	-2.2469424192	1.8214819388
N	2.4788799952	-1.6612502759	0.7639617413
O	5.0717388437	-1.3813386448	-1.6730032476
O	5.9873798758	-0.5514012804	0.9268242299
H	-0.6611445018	-1.5822112987	-0.0650879846
H	-0.396404252	-2.5635760844	1.8879814641
H	1.4183036713	-3.7955477998	3.2366345515
H	0.6877091828	-2.3722153689	4.0042774851
H	4.2966970221	-1.7615200103	1.7696682883
H	3.4588445233	-3.3145417174	1.6164885402
H	1.0998007377	-1.9115756128	-2.0690987511
H	0.1099098752	-0.4407338529	-1.9488604153
H	3.2851180253	-2.4679158922	3.9628164763
H	2.5627911426	-0.9619522699	3.3677722613
H	3.2253016731	2.4713961699	-0.0653401813
H	4.1186894864	-4.9539547391	-1.7223050101
H	5.5824994282	-3.9612594682	-1.4336210223
H	4.5761075789	-3.6628236315	-2.8770072842
H	3.3404944462	-0.4816153101	-3.0875715813
H	3.3768336639	2.38783336	-2.5160374583
H	3.4970363673	0.4839339883	1.3183699434
C	7.3476151773	1.5539345162	0.1155300734
H	7.6335239988	2.5190526657	-0.2984164084
H	7.6242038729	1.4928645607	1.1696933597
H	7.8269219379	0.7435054076	-0.4364260394
O	-2.4224759974	-2.17574439	-0.4957024033
C	-2.7542614142	-3.1428731625	0.15904672
O	-2.1475778965	-3.4083610426	1.3144049336
C	-3.8992563709	-4.0645028898	-0.2407024974
N	-3.7696423746	-5.3756173447	0.3626934227

C	-4.9493609525	-5.7229835233	1.1610263309
C	-5.4749817459	-4.3512493179	1.5671950952
C	-5.2423303652	-3.5214476156	0.30245085
C	-2.8996890115	-6.3205717788	-0.1893262807
H	-4.6626469198	-6.3358279698	2.018718773
H	-5.6994469828	-6.2680930283	0.5698129361
H	-4.8812638087	-3.9602601564	2.4007215474
H	-6.5232099841	-4.3757299503	1.8693778836
H	-6.0209317909	-3.7314523104	-0.4352051026
H	-5.218591551	-2.4434403094	0.4755137757
H	-3.8920553303	-4.1019871023	-1.3367821538
C	-3.105553518	-7.7476190007	0.2685408548
C	-2.3646332834	-8.7500485781	-0.615373443
H	-4.1761241788	-7.977085329	0.2701783195
C	-0.9269909218	-8.2985001539	-0.8499145928
H	-2.3954934024	-9.7392233302	-0.1486534173
H	-2.880920841	-8.8308933754	-1.5802254873
C	-0.912081397	-6.9670211748	-1.5995774414
H	-0.4297216298	-8.1804729249	0.1214367317
H	-0.366652408	-9.0534699322	-1.4103618306
C	-1.9188276804	-5.9855798542	-1.0533422149
H	0.0915116347	-6.5244593002	-1.5500164382
H	-1.1055015778	-7.1457476293	-2.6674754728
H	-1.8171711155	-4.9569626028	-1.3927535354
C	-1.6857092956	-6.4802616594	3.260983946
C	-0.3885877782	-6.7631290453	2.6214758928
H	-2.0155190352	-7.2215503665	4.0095954698
O	-2.3841271364	-5.5103977114	3.0183932273
C	0.1692419834	-5.8794613073	1.6899052731
C	1.3880913273	-6.1722654573	1.0957935364
C	2.0299376952	-7.3634966036	1.4358967744
C	1.493008693	-8.2588568307	2.3563484493
C	0.2759465805	-7.9468314776	2.9517936214
H	-0.3519156894	-4.9654183523	1.4269861527
H	1.8343173466	-5.4964555822	0.374210842
Cl	3.5543652728	-7.7424220528	0.6774398999
H	2.0165525961	-9.1763116613	2.5997321589
H	-0.1641369264	-8.6280339734	3.6751991949
H	-2.4381705716	-4.268150229	1.7067430449
H	-2.7703981839	-7.8489069657	1.3109846268
H	4.6966911678	1.2694496549	-2.1256127035
H	1.9697674332	0.632998266	-3.2024540959

IIc

C	3.5647310739	2.5208723868	-0.5904733258
C	3.1368324293	1.737089923	0.6639812022
O	1.8924542084	1.1338313789	0.2710824368
C	2.3673437079	0.491256716	-0.9111488937
C	2.9239737819	1.6726614383	-1.7131102586
C	3.9799445304	0.4636074354	0.8707343214
C	3.507075146	-0.428189899	-0.3299531827
C	4.6115304841	-0.6539718347	-1.3668735204
O	4.4876580834	-1.8131489716	-2.0010160709
C	5.4745092696	-2.0636013063	-3.0166749852

C	5.4722994213	0.602697246	1.0849403953
O	5.825124215	1.8607499366	1.3404799012
C	1.2869271546	-0.3041102292	-1.5889729974
N	0.8126671953	-1.330012515	-0.6811744144
C	1.6692964844	-2.0589766804	0.0449278392
N	1.2134097814	-3.1358719781	0.6799095844
C	1.9724721081	-3.9465321533	1.63255086
C	3.2183880616	-3.1957754439	2.0653109649
C	3.8903322629	-2.6188575202	0.8305851682
N	2.9641066804	-1.7065978422	0.1354290786
O	5.4614102838	0.170726721	-1.6070753781
O	6.2419634281	-0.3306090031	1.1166277038
H	-0.1488868034	-1.6471383696	-0.7594202164
H	0.2203766335	-3.3425108566	0.567347975
H	2.2441246365	-4.8935418046	1.1557066419
H	1.3187800986	-4.1631063369	2.4798098102
H	4.7809478743	-2.0448027263	1.091495
H	4.1865920257	-3.4146493493	0.1389779347
H	1.6836693939	-0.7356165837	-2.5165502442
H	0.4530026202	0.3521128937	-1.8428568622
H	3.9010485836	-3.8781516766	2.575048354
H	2.9596245034	-2.3861158932	2.7548764435
H	2.9964152839	2.3252933082	1.5692719919
H	5.2363103202	-3.0448066732	-3.4219928467
H	6.4727568561	-2.0614541046	-2.5756914002
H	5.4137332293	-1.301274548	-3.7952131252
H	3.6126042889	1.3936190811	-2.5084460833
H	3.1521239317	3.5307960694	-0.5648221181
H	3.6095327605	-0.0202713146	1.7819441865
C	7.2166665894	2.0623291127	1.6292597281
H	7.3305007207	3.1303680573	1.8044282988
H	7.5040728491	1.4962238675	2.5171710505
H	7.8253448312	1.7462484663	0.78027807
O	-1.659076183	-3.3453950861	0.7847661955
C	-2.5035161183	-2.653797361	0.252049656
O	-2.1909527883	-1.9043398144	-0.8001036883
C	-3.9539288085	-2.6411824061	0.7228022464
N	-4.7101311972	-1.5291519086	0.1515508805
C	-5.8099287545	-2.0184684558	-0.684744437
C	-5.3552762523	-3.4193310051	-1.072697401
C	-4.6800138218	-3.9051419286	0.209773295
C	-4.8368237085	-0.3063751565	0.8178744836
H	-6.7470098786	-2.0467666017	-0.1079858468
H	-5.9508523621	-1.3591404108	-1.5444791496
H	-6.1843338329	-4.0566746085	-1.3839835656
H	-4.6325508945	-3.3649159347	-1.8944557613
H	-5.4349529681	-4.1911880754	0.9467181845
H	-3.997522887	-4.7456378191	0.0706496202
H	-3.9189524617	-2.6299364379	1.8151791362
C	-5.8504616996	0.5476585803	0.5629541444
C	-6.0162208555	1.869297298	1.2675864971
H	-6.6150057592	0.2738081815	-0.1579252949
C	-5.1747487759	1.9470017806	2.539688067
H	-5.7343881039	2.6966164247	0.5975030132

H	-7.0757609649	2.0227613039	1.5026661449
C	-3.7501844131	1.4925381553	2.2376219975
H	-5.6107773831	1.2953328693	3.30770986
H	-5.1783081453	2.9669699516	2.9364366469
C	-3.729449756	0.0288694932	1.7940751998
H	-3.1011058979	1.6151033659	3.1098781623
H	-3.3416604385	2.1242727446	1.4400618989
H	-3.8137009204	-0.622131629	2.6740263557
H	-2.7599439865	-0.2029034616	1.3310611913
C	-4.1495562971	0.9213264924	-2.0020396641
C	-3.2528766032	1.8937368975	-1.3488056298
H	-5.1259147868	1.3176279348	-2.3308655089
O	-3.8461873684	-0.2392902821	-2.2295810632
C	-3.6691415255	3.2187362864	-1.2014492578
C	-2.8290846273	4.157004112	-0.6095825149
C	-1.5720135889	3.7448406241	-0.1783170353
C	-1.1318876475	2.4282438811	-0.3170371072
C	-1.9840104155	1.5056685065	-0.9064397316
H	-4.6512860585	3.5184738577	-1.557548172
H	-3.1395232231	5.188895152	-0.4910298727
Cl	-0.5024100785	4.9120572463	0.5586760989
H	-0.1422462622	2.1365430129	0.0231324524
H	-1.6648039503	0.4749655937	-1.0261054093
H	-2.9616761875	-1.3722824658	-1.1207603225
H	2.0674273809	2.1857561822	-2.1585876725
H	4.6486432376	2.5844464959	-0.68053734

IId

C	2.1993171752	2.780122572	0.1585046681
C	1.8029265018	1.5210285583	0.9516265893
O	0.8651523397	0.864574182	0.0793356974
C	1.7000223464	0.8100089629	-1.0801210768
C	2.0347079866	2.2894397415	-1.2988060069
C	2.9248729311	0.4655960508	0.960713584
C	2.9265768517	-0.0132736833	-0.5341936438
C	4.2224592812	0.353957422	-1.264429443
O	4.5845090922	-0.5553456693	-2.1596216639
C	5.782928784	-0.246686371	-2.89239994
C	4.2627619072	0.8242043827	1.571650426
O	4.1981384709	1.9360828719	2.3001802623
C	1.0103771011	0.1175541532	-2.2250168559
N	0.6909459254	-1.2421512473	-1.8394694773
C	1.5409842197	-1.9855682553	-1.1249706748
N	1.2512526664	-3.2669014448	-0.9096752879
C	1.9526044365	-4.1580935328	0.016323854
C	2.8926596752	-3.3520896664	0.8942534956
C	3.6372207889	-2.3585260979	0.0166215251
N	2.6703505656	-1.452067453	-0.6281078729
O	4.8131906558	1.3883669771	-1.0605325655
O	5.2500825599	0.1294608531	1.4879732932
H	-0.2180725926	-1.6370432368	-2.0558754366
H	0.409734056	-3.6458781329	-1.3391180494
H	2.5133087564	-4.8969757417	-0.5643471402
H	1.1964056715	-4.6830670875	0.6042628631

H	4.3261304044	-1.7436431541	0.5980413135
H	4.2109767922	-2.8731572521	-0.7609081553
H	1.6615501466	0.1418876682	-3.1073019994
H	0.0814042424	0.6394753937	-2.4661582984
H	3.5986922516	-4.0219991549	1.3883357453
H	2.333050338	-2.8100841007	1.6644117096
H	1.3451060408	1.6900716667	1.9259170044
H	5.9385810153	-1.0853966833	-3.5675220336
H	6.6240042619	-0.144504878	-2.2047168826
H	5.6513467065	0.6796988605	-3.4540092475
H	2.906000793	2.4650254403	-1.9262019977
H	1.5190152378	3.6031810597	0.387057192
H	2.5673735409	-0.3800164282	1.5605929912
C	5.4109930614	2.3013739101	2.976567168
H	5.1821231918	3.2167230557	3.5188488652
H	5.7106011494	1.5114786642	3.6675108667
H	6.2071890636	2.4751599843	2.2506126496
O	-1.2622166567	-4.5918117427	-0.7681932731
C	-2.0243391968	-3.6621373886	-0.5945076101
O	-1.9539342314	-2.5847577475	-1.3705049645
C	-3.1137449468	-3.6921288048	0.4741533077
N	-3.5818722588	-2.3698107337	0.8278129631
C	-5.0186089896	-2.2102583264	0.5911827082
C	-5.2862471476	-3.2620327809	-0.4806270821
C	-4.375242568	-4.414042574	-0.0505760869
C	-2.8189221028	-1.5297025235	1.6346738708
H	-5.2425287637	-1.1965446287	0.2504338464
H	-5.6087462559	-2.4163110995	1.4959459879
H	-4.981408862	-2.8780417947	-1.460363851
H	-6.337604031	-3.5495625342	-0.5333513915
H	-4.8290297775	-4.9650144171	0.777306844
H	-4.1472790872	-5.1227490243	-0.8493655994
H	-2.6729984897	-4.2217251375	1.3279828542
C	-3.5485274438	-0.3219537951	2.1825988182
C	-2.7369098872	0.4242502975	3.2411113788
H	-4.5039629027	-0.648598141	2.6084234015
C	-1.2845057011	0.5784785239	2.7997227092
H	-3.1980900574	1.3977653466	3.4326948635
H	-2.7644231608	-0.1374626042	4.1832474834
C	-0.6382379513	-0.7980459681	2.6629017096
H	-1.2533931189	1.0909668155	1.8275429224
H	-0.7255054605	1.1942770063	3.5121195188
C	-1.5112688377	-1.7534877835	1.8878409249
H	0.3290999338	-0.7003563248	2.1540487988
H	-0.4172870603	-1.2002576975	3.6628243236
H	-1.029849532	-2.6558882095	1.5157086318
H	-2.5929882468	-1.868150611	-1.1136255634
H	-3.8059164677	0.3545959098	1.3564720775
O	-3.2208320019	-0.2647325291	-1.267545088
C	-2.416894881	0.5669634839	-0.8693284228
C	-2.7036803465	2.0102912839	-0.844588509
H	-1.4201609619	0.2609941079	-0.4995516622
C	-1.6978845833	2.8962417857	-0.448452513
C	-1.9412270472	4.2646981039	-0.4260699864

C	-3.2022484881	4.7203774571	-0.7977758567
C	-4.2219881398	3.852655554	-1.1917275073
C	-3.9650649431	2.4906266283	-1.2155467035
H	-0.7248174061	2.5046908145	-0.1623988307
H	-1.171142887	4.9657629284	-0.1243521271
Cl	-3.5221587752	6.4349694413	-0.7674331977
H	-5.193420631	4.2437975987	-1.4724765373
H	-4.7368426708	1.7903146547	-1.5186433746
H	3.2178588179	3.0971583459	0.3803339576
H	1.1637328133	2.7473156749	-1.7762951778

IIIa

C	-3.6072344315	3.3252709736	-0.7339747299
C	-3.6707411955	2.0605471595	-1.612422883
O	-2.4456249387	1.3797146765	-1.3063938893
C	-2.6100449004	1.3374999188	0.1087453909
C	-2.7556030507	2.824410672	0.4546980933
C	-4.682418459	1.0314716695	-1.0707303821
C	-3.9779210089	0.5611568843	0.2493083642
C	-4.7343002177	1.004750259	1.50480275
O	-4.5498519335	0.1814955315	2.5305805485
C	-5.1981299973	0.5734782586	3.7520611751
C	-6.1426090272	1.4215796826	-0.9817154282
O	-6.4013232093	2.5468904865	-1.6464963573
C	-1.4723568872	0.622880178	0.784722175
N	-1.4398613037	-0.7623398117	0.3702719282
C	-2.554199313	-1.4750763825	0.2153865302
N	-2.4433632657	-2.7870512884	0.0107256415
C	-3.5484627135	-3.6893707138	-0.2993435556
C	-4.7650357149	-2.8897688114	-0.7302193372
C	-4.9628392246	-1.7431240397	0.2475145727
N	-3.7670892142	-0.8857434924	0.2584119232
O	-5.3797777331	2.0254127232	1.5577111612
O	-6.9961938919	0.7447593852	-0.4543390104
H	-0.5257661434	-1.2324796506	0.2454679856
H	-1.4979343965	-3.1956950822	0.1055604157
H	-3.7854699234	-4.2867365398	0.5878812214
H	-3.2180686402	-4.3695748426	-1.0879245951
H	-5.8126964493	-1.1196737367	-0.0358325146
H	-5.1402773976	-2.121317917	1.2604699881
H	-1.5882072977	0.7177819075	1.8722450156
H	-0.5275074051	1.0899686994	0.498801114
H	-5.6472421447	-3.5330113277	-0.7358098989
H	-4.6238777683	-2.489194885	-1.7393213438
H	-3.7472373457	2.2306711403	-2.6852424512
H	-4.9609970337	-0.205005361	4.4741467789
H	-6.2765687416	0.6396752572	3.5985511134
H	-4.8149705227	1.5387035527	4.0880226272
H	-3.1949718384	3.0202461815	1.43149414
H	-3.1121108062	4.1358587907	-1.2710140914
H	-4.6636440664	0.1725730042	-1.7515932189
C	-7.7794114766	2.9455799792	-1.6672951785
H	-7.8113851531	3.8698359425	-2.2411145141
H	-8.3890217406	2.1771877111	-2.1463268807

H	-8.1366763429	3.1144309837	-0.6498087551
O	0.0987397105	-3.9386645334	0.2443408505
C	1.0752753919	-3.1736994817	0.1897379041
O	1.0546610238	-1.9137633461	0.0907158455
C	2.4598050956	-3.8770622966	0.2503353143
N	3.5306860859	-2.8555054755	0.2539439216
C	4.0774881675	-2.6864975464	1.6354328893
C	3.1275808247	-3.5019403546	2.5070808059
C	2.6601599022	-4.6157949872	1.5725820526
C	3.8333318318	-2.1197264104	-0.7632805341
H	5.0912811484	-3.0962604429	1.6380598931
H	4.0963958341	-1.6299543238	1.8917233927
H	3.629927497	-3.8691954835	3.4024716028
H	2.2796162807	-2.8816633711	2.8138113221
H	3.4379838693	-5.3761647887	1.4589724248
H	1.7337502796	-5.0979787061	1.8843864913
H	2.5572335042	-4.5347818259	-0.6132681795
C	4.7333249455	-0.927873605	-0.6049185285
C	5.6872490724	-0.7777569777	-1.8027832791
H	5.3274262287	-1.0186666142	0.3069584525
C	4.9797980925	-0.8681267676	-3.1527345768
H	6.2287523347	0.1665590519	-1.6993905096
H	6.4331971246	-1.578352131	-1.7402055478
C	4.2098363908	-2.1807942777	-3.2500693752
H	4.2901249438	-0.0254248359	-3.2848054598
H	5.7194253295	-0.8051283627	-3.9562131826
C	3.1942122725	-2.317591875	-2.1059269563
H	3.6676391559	-2.2518277478	-4.1964124916
H	4.9109961994	-3.0225424026	-3.2125623297
H	2.6648779834	-3.2669966518	-2.1684860734
H	2.4363401126	-1.5272434858	-2.1968179851
C	3.7744274607	0.3033585624	-0.3918342554
C	4.59129932	1.5387070073	-0.0938081761
H	3.2116115584	0.4733519443	-1.319939315
O	2.9047237391	0.0735723298	0.6907945356
C	4.904563261	2.4462827872	-1.1053388856
C	5.6857032778	3.5687383227	-0.8410190785
C	6.1441201747	3.7725223161	0.454502729
C	5.8411513768	2.8867477599	1.4838512999
C	5.0644505629	1.7685264989	1.199894752
H	4.532558782	2.2813319778	-2.1132714255
H	5.9278671868	4.2751184839	-1.6272660313
Cl	7.1239009565	5.1822035284	0.8019810698
H	6.2055055034	3.0703597091	2.488775655
H	4.8112273356	1.0735205365	1.994669829
H	2.251307307	-0.6173471173	0.4535543587
H	-1.7514897302	3.2555431021	0.4335622136
H	-4.5979244405	3.6590215817	-0.427174068

IIIb

C	-5.8508634781	-2.1480440359	-1.3755468945
C	-5.1635886864	-2.3815958517	-0.0165147328
O	-3.8275747009	-2.7593736813	-0.3790497345
C	-3.5357030319	-1.6303217508	-1.1977233778

C	-4.6545549537	-1.6992413266	-2.2451003504
C	-4.8697059577	-1.0585228994	0.7178181043
C	-3.7502918258	-0.4369570238	-0.1871230569
C	-4.218735504	0.8212710467	-0.9236028167
O	-3.2173319475	1.645921815	-1.212138395
C	-3.5881313425	2.8201259123	-1.9542992537
C	-6.0284415077	-0.1648965411	1.107328095
O	-7.2020130257	-0.7822883908	0.9790998345
C	-2.1379564439	-1.6806035986	-1.7484264282
N	-1.1745887233	-1.6002475208	-0.6708815735
C	-1.3525925632	-0.7781693436	0.3646289491
N	-0.3287086248	-0.5805064992	1.1950491213
C	-0.3767377673	0.1929963132	2.4330251132
C	-1.8203732038	0.3891284095	2.8579864424
C	-2.6244254382	0.8398183192	1.6501710165
N	-2.5346822201	-0.167013081	0.5794023741
O	-5.3665939363	1.0017128769	-1.2557402925
O	-5.9032751975	0.9447451733	1.573821876
H	-0.2252752642	-1.9785642801	-0.8326776638
H	0.5691109019	-1.028755236	0.9496792468
H	0.1088986779	1.1626254565	2.2713312973
H	0.1946079864	-0.3496853137	3.1897909906
H	-3.6802724022	0.9627677104	1.8971745316
H	-2.2511199737	1.7979942098	1.2708056945
H	-2.009211369	-0.8629871953	-2.4697784007
H	-1.9925869511	-2.6275889288	-2.2718508305
H	-1.8770698109	1.144723197	3.6439523648
H	-2.2356736547	-0.5461375392	3.2468787751
H	-5.610272674	-3.1461979641	0.617032169
H	-2.6676028772	3.3824648292	-2.0963869021
H	-4.3132779623	3.4068355368	-1.3879159382
H	-4.0167166247	2.5360606035	-2.9170446395
H	-4.8223927922	-0.7706239786	-2.7882508006
H	-6.2894082804	-3.0770317139	-1.7432504636
H	-4.3869852082	-1.3198975429	1.6664817194
C	-8.3456753419	-0.0219915939	1.3946201606
H	-9.207217799	-0.6627912663	1.2164627108
H	-8.2691388357	0.229729913	2.4540062091
H	-8.421647076	0.8938974693	0.8057990727
O	1.4042957428	-2.4866688792	-1.2870064049
C	2.3249741751	-2.1202231967	-0.5386914195
O	2.2083518718	-1.5415194375	0.5782578743
C	3.7509586342	-2.4034880685	-1.0736212882
N	4.8035270686	-2.1691534793	-0.0502304001
C	5.2937861201	-3.4693476646	0.4960519424
C	4.324305005	-4.4942181369	-0.0813438841
C	3.9409022147	-3.8794681609	-1.4259807179
C	5.2682522229	-1.0184102469	0.3014794011
H	5.2981421157	-3.424095568	1.5848677616
H	6.3121131626	-3.6247099135	0.1296168395
H	3.4429635093	-4.5840851709	0.5612871162
H	4.7915045508	-5.4753360453	-0.1706018109
H	4.7577698328	-3.9893998269	-2.1447741411
H	3.0266148348	-4.2854137459	-1.8570164473

H	3.9281490666	-1.7462579415	-1.926538623
C	6.4666633035	-0.8856724188	1.1834499275
C	7.5855403829	-0.2344353919	0.3365582862
H	6.7933127843	-1.841709729	1.5882813489
C	7.142292838	1.1092340526	-0.2402945757
H	8.4690785698	-0.1161458143	0.9687584078
H	7.8546063132	-0.9190203614	-0.4769296608
C	5.8470432064	0.9730790575	-1.0449660614
H	7.0112723809	1.8284220882	0.5760855221
H	7.9267097657	1.5099457393	-0.8890522735
C	4.7240903693	0.2697106062	-0.2503187332
H	5.4785740964	1.9527262718	-1.366694331
H	6.0422027584	0.3889859183	-1.9517569248
H	3.8812068998	0.0716368863	-0.9150287915
C	4.1969684983	1.1341884554	0.9341550716
C	3.2555481908	2.2036168416	0.4124033391
H	5.0458507688	1.6361070547	1.4102474529
O	3.6040060182	0.3285816646	1.9250383152
C	1.9510679853	1.8611346564	0.0457376872
C	1.0676103483	2.8209753997	-0.4336674312
C	1.5084281734	4.1364855602	-0.5508885121
C	2.8011306871	4.505209037	-0.2016951527
C	3.6694605654	3.5282844115	0.2833541049
H	1.6207814865	0.8290787115	0.1363742554
H	0.0539230438	2.5534810267	-0.714167159
Cl	0.4037547274	5.356504161	-1.1514087519
H	3.1228108932	5.5363999021	-0.2978761146
H	4.6794399517	3.8080516508	0.5721955644
H	3.075933705	-0.3680913808	1.4819687858
H	6.2032953807	-0.2299012176	2.0195769423
H	-6.6315301715	-1.3907015107	-1.3129186812
H	-4.3727476373	-2.4735755401	-2.9630699779

IIIc

C	-3.6317776144	3.2495337434	-0.8668919373
C	-3.7616456596	1.9600977345	-1.700512573
O	-2.5130642854	1.2900454337	-1.4749542802
C	-2.5637073985	1.2869350468	-0.0510279459
C	-2.6884511387	2.7828832753	0.2651331266
C	-4.7230961074	0.9460419697	-1.0497700152
C	-3.9126893869	0.5133315052	0.2207189818
C	-4.5589470642	0.9925360801	1.5233616049
O	-4.2518916404	0.217092046	2.5573412668
C	-4.7782781861	0.6485026221	3.8232537533
C	-6.1732640661	1.3347011734	-0.853336924
O	-6.4890798237	2.4466864957	-1.5158606387
C	-1.3728700222	0.5967270196	0.5543821512
N	-1.3684584719	-0.8022543496	0.186371761
C	-2.4890099217	-1.5225612744	0.1486043079
N	-2.3884499985	-2.8420632602	-0.0029112808
C	-3.5094874772	-3.7584916297	-0.1910209705
C	-4.7561808099	-2.9801254137	-0.5714743965
C	-4.8886920291	-1.7896184291	0.3638372653
N	-3.6978392722	-0.9325771081	0.2516541448

O	-5.22028566	2.0013427162	1.6000604323
O	-6.97959038	0.6651080457	-0.2481542209
H	-0.4601740366	-1.2858868082	0.0808729785
H	-1.4339941387	-3.2409460407	0.0124169799
H	-3.6807579165	-4.3139983579	0.7375502546
H	-3.2329021784	-4.4743368983	-0.9684943924
H	-5.7580086905	-1.1797944035	0.111839424
H	-4.994144787	-2.1205441247	1.403314991
H	-1.4011288746	0.7296030462	1.6438158785
H	-0.4570538318	1.0556270556	0.1758054848
H	-5.6346544028	-3.6226108572	-0.485434234
H	-4.6873647544	-2.6262898471	-1.6052136475
H	-3.925040336	2.098722371	-2.7680935858
H	-4.4381856853	-0.0868405183	4.5494502794
H	-5.8685673039	0.6756329503	3.7858067511
H	-4.394365328	1.6394761139	4.0720751136
H	-3.0516104047	3.004849644	1.2673969653
H	-3.1813915744	4.0437839061	-1.464309826
H	-4.7556798712	0.0682971513	-1.7052741532
C	-7.8671307752	2.8381251232	-1.4365190418
H	-7.9485747209	3.7542712686	-2.0183504447
H	-8.5068236049	2.0600508195	-1.8572301051
H	-8.1469507206	3.0189835424	-0.3971386612
O	0.1719554353	-3.9597644536	0.0186561854
C	1.1584503144	-3.2052166182	0.0016924693
O	1.1552420127	-1.9427157863	-0.022869369
C	2.5326042575	-3.938144884	0.0258391991
N	3.6140917525	-2.9416531278	0.1753195105
C	4.0911001921	-2.9141997181	1.5902548383
C	3.1083096268	-3.8144962733	2.3320423332
C	2.6789162862	-4.8188162827	1.2644373293
C	3.9892572389	-2.130991169	-0.7594768672
H	5.1054429406	-3.3227802666	1.5944152759
H	4.1097926354	-1.8960826477	1.973332757
H	3.575829395	-4.2773773037	3.2015710218
H	2.2474886532	-3.2290220181	2.6694028146
H	3.4604882922	-5.5657720418	1.09946073
H	1.7400733136	-5.325538935	1.4854497035
H	2.6443236889	-4.4980930538	-0.9025821443
C	4.8226640401	-0.9256070799	-0.409880443
C	5.7089435395	-0.4603559562	-1.5733920211
H	5.4667777082	-1.1595917081	0.4428549947
C	4.9614460451	-0.3820399721	-2.9002617121
H	6.1560519954	0.5009087058	-1.3069020235
H	6.5346202834	-1.1747753546	-1.6762429148
C	4.4167025302	-1.7636642063	-3.2414653668
H	4.1402568868	0.3398269318	-2.8312296225
H	5.6407158277	-0.0414933591	-3.6873070236
C	3.4627769164	-2.2823886121	-2.1590415105
H	3.8743377738	-1.7563643893	-4.1905067065
H	5.2528551645	-2.4639226827	-3.3520432307
H	3.1964788823	-3.3214074858	-2.3561472491
H	2.5311974926	-1.7043566379	-2.1899817036
C	3.7824706873	0.1507001111	0.1052980054

C	4.4599969793	1.4892215997	0.2859480889
H	3.4429472891	-0.1902528859	1.0921877291
O	2.6739817332	0.2846001955	-0.7490657523
C	4.1787016667	2.5588808988	-0.5624218091
C	4.8343916565	3.7786338682	-0.4067833271
C	5.7741796265	3.9123038531	0.6071768346
C	6.0696438482	2.8612698859	1.470008236
C	5.4059503226	1.6516307859	1.3010305757
H	3.4355869699	2.4398260328	-1.3430618193
H	4.6161025747	4.612819642	-1.0648751281
Cl	6.6052271956	5.4408225678	0.8104115037
H	6.8012449485	2.9893568175	2.2601800913
H	5.6260457213	0.8255293003	1.9730948153
H	2.0565288453	-0.4383907585	-0.5259972482
H	-1.6909533497	3.2156596482	0.1548872016
H	-4.5962444862	3.5926542452	-0.4941288737

IIId

C	4.3771062839	0.8997813648	-2.9502830002
C	3.5805423671	1.597130407	-1.8300971474
O	2.4104944558	0.7782333388	-1.6943519792
C	3.0800403771	-0.4639048859	-1.4969226865
C	3.9327615947	-0.569585246	-2.7673141739
C	4.215643192	1.3746045176	-0.4433046451
C	3.9608359349	-0.1569239366	-0.223230136
C	5.2539862185	-0.9762972458	-0.2479979234
O	5.1542852206	-2.1036498515	0.4473539843
C	6.3168004117	-2.9480360162	0.4123772325
C	5.615307522	1.8937285929	-0.1900389869
O	6.0305218747	2.7086749694	-1.1590301591
C	2.1117084181	-1.5916488686	-1.2668996845
N	1.3697688618	-1.3703553133	-0.0451082099
C	1.9546741921	-0.8848837117	1.0492450352
N	1.2633393607	-0.8784976348	2.1876462466
C	1.7011274194	-0.2671716166	3.4391955191
C	2.8733474576	0.6625398748	3.1808426614
C	3.8678541644	-0.0501519441	2.2787892248
N	3.2168804969	-0.4102542972	1.0091708708
O	6.2287226389	-0.652023918	-0.8850838067
O	6.2490438446	1.6764749336	0.8179127525
H	0.3628602249	-1.6069740229	-0.0091945652
H	0.3585245336	-1.3799559924	2.1820959132
H	1.9938338281	-1.0561753182	4.1404526166
H	0.8525661298	0.2706635256	3.8686897063
H	4.7200354409	0.5893113216	2.0419068059
H	4.2457745903	-0.9606500159	2.7574931244
H	2.6673469392	-2.5377677195	-1.2365515702
H	1.4033166851	-1.6348621112	-2.0964411618
H	3.3492319112	0.9292554601	4.1265913576
H	2.5342981884	1.5825239074	2.6938032293
H	3.2949753044	2.6300327851	-2.0226914118
H	6.069957512	-3.8140178355	1.0229592062
H	7.1767424124	-2.4187862654	0.8261733019
H	6.5308828055	-3.2503368333	-0.6143089727

H	4.7564356155	-1.2790757065	-2.7013800166
H	4.0820005635	1.2909519574	-3.9252246019
H	3.586010198	1.8977450481	0.2854136411
C	7.3242851324	3.2946833838	-0.9565313547
H	7.5093412246	3.9134881986	-1.8325810744
H	7.3270481583	3.9038223076	-0.0507237448
H	8.0816848135	2.5130896819	-0.8736299369
O	-1.1874868482	-2.2119650521	2.1940144291
C	-1.7809966203	-2.2967025487	1.1064637496
O	-1.3397766122	-1.9937514295	-0.0399847322
C	-3.2110655489	-2.8938833551	1.1880756806
N	-3.8206993543	-2.9075675169	-0.1576573821
C	-3.4559933471	-4.1791014958	-0.8491863753
C	-2.6642495835	-4.9762576613	0.189169757
C	-3.1190733158	-4.3822115729	1.5240146972
C	-4.4946459211	-1.9540739707	-0.712000477
H	-2.887391504	-3.9421141273	-1.7496385966
H	-4.3831974362	-4.6854736315	-1.1266430117
H	-1.5920790133	-4.8152920487	0.0505207868
H	-2.8682007215	-6.0438482964	0.1036084934
H	-4.1051627399	-4.7606481488	1.8076296
H	-2.4196833672	-4.5523811122	2.3424873791
H	-3.8152409447	-2.3222097253	1.8914091794
C	-4.84726057	-2.0856585997	-2.1667009346
C	-6.0336213812	-1.2496943412	-2.6512169287
H	-4.9866852283	-3.1340120052	-2.4342869032
C	-6.0215278057	0.1476955031	-2.0468284588
H	-6.0005185178	-1.2171986698	-3.7433515834
H	-6.9667690587	-1.7514665576	-2.37110667
C	-6.0518762326	0.0138262977	-0.5293359524
H	-5.1222938726	0.6906261463	-2.3629142267
H	-6.8896508957	0.7164081425	-2.3920816847
C	-4.7937134084	-0.667764248	0.029295451
H	-6.1663232929	0.983805597	-0.0405173076
H	-6.924690652	-0.5866754956	-0.2451925979
H	-4.9791312411	-0.9015397485	1.0815818383
C	-3.4832135809	0.2128818269	-0.0132264472
C	-3.7780020053	1.6785940841	0.2166604308
H	-2.862016125	-0.1176634016	0.8284306508
O	-2.7804393461	0.0356686728	-1.2198542941
C	-4.218707728	2.1045782958	1.4729018885
C	-4.5141506888	3.4418556354	1.7098742792
C	-4.3582736084	4.3554065407	0.6714055295
C	-3.9131288798	3.9604180261	-0.5831658959
C	-3.6236158409	2.6149492771	-0.8036738586
H	-4.3324736563	1.38342929	2.2788705734
H	-4.8542240514	3.7724802227	2.685134524
Cl	-4.7237265595	6.0444547882	0.9598803865
H	-3.7904512662	4.6904034102	-1.3759746887
H	-3.2610022196	2.2908988337	-1.7728328383
H	-2.1449304262	-0.6852709912	-1.0427981691
H	-3.9315453785	-1.7458145367	-2.6751009669
H	5.4517378878	1.0306279576	-2.8281676638
H	3.2671549887	-0.8874355287	-3.5738991389

TS1a

C	-3.8679938125	3.3637394131	-0.3045861564
C	-3.7371135317	2.2331367123	-1.3435424597
O	-2.5012929307	1.5958984418	-0.9883094391
C	-2.8133243017	1.3455808869	0.3790769059
C	-3.1133126422	2.756417144	0.9001707376
C	-4.7152922232	1.0746823623	-1.0653693061
C	-4.1234661877	0.4735458685	0.2558927323
C	-5.0366111003	0.6926649911	1.465149235
O	-4.8615538694	-0.2289462273	2.4050330176
C	-5.6482237313	-0.0477801709	3.5946067744
C	-6.2035669069	1.3511477441	-1.1007455153
O	-6.4790914131	2.5443847902	-1.6248567594
C	-1.7019154377	0.6165722497	1.0823952572
N	-1.5205815735	-0.6908509985	0.4879468357
C	-2.5553229601	-1.4476884614	0.1204167493
N	-2.3237149093	-2.7100510151	-0.2365243266
C	-3.3191985016	-3.6331504987	-0.7771502452
C	-4.5452787826	-2.8605495601	-1.229267552
C	-4.9256623224	-1.8694094861	-0.1416795914
N	-3.804881991	-0.9466655646	0.1051258061
O	-5.7822938558	1.6383932616	1.5657745927
O	-7.0511387795	0.5477889708	-0.7832100962
H	-0.5769709322	-1.0745934534	0.4062439723
H	-1.3708024949	-3.0617507433	-0.0887287422
H	-3.592424891	-4.3578827595	-0.0030226105
H	-2.8606063343	-4.1758422358	-1.606564131
H	-5.7876984447	-1.2678577725	-0.4354048589
H	-5.1717899927	-2.3880925682	0.7914667195
H	-1.9388511151	0.5434527916	2.1512921984
H	-0.7711822657	1.176169565	0.9714772472
H	-5.3705743166	-3.5520608465	-1.4094049747
H	-4.3379917515	-2.3212490034	-2.1590798662
H	-3.7111920723	2.5464255762	-2.3858995872
H	-5.3980229818	-0.884854144	4.2430446699
H	-6.7106070347	-0.0584753554	3.3458782696
H	-5.3920989602	0.8988550735	4.073748754
H	-3.6705607092	2.7843514865	1.8353214946
H	-3.3817416774	4.2704266959	-0.6678384484
H	-4.5511759789	0.3225896895	-1.8456628964
C	-7.8750810518	2.850137152	-1.7549794102
H	-7.9204496385	3.8485576732	-2.1856932973
H	-8.3610649283	2.1272977227	-2.4128855897
H	-8.3557459898	2.8340512845	-0.7751495893
O	0.3338848964	-3.698383429	0.165892361
C	1.2962255348	-2.953518175	0.3182626384
O	1.1824170636	-1.6588706225	0.338209993
C	2.6763298845	-3.590684269	0.5223524236
N	3.7839714904	-2.6444532314	0.3965643756
C	4.4829181548	-2.4450601229	1.6791320434
C	3.4837964191	-2.9723964118	2.7016935198
C	2.8188567832	-4.1301040742	1.9586827816
C	4.1946597231	-2.0971967495	-0.7675788715

H	5.415383323	-3.0242546359	1.679391522
H	4.7172129016	-1.3925684719	1.8318641127
H	3.9659608662	-3.2804193098	3.6305181276
H	2.7489639342	-2.1932009684	2.9310103799
H	3.4812793732	-4.9998370983	1.9438014842
H	1.8563736592	-4.4346131962	2.3724186375
H	2.7473004675	-4.4040049706	-0.2029559917
C	5.162364799	-1.1089425174	-0.7957787387
C	5.7628140109	-0.6120725921	-2.0873532683
H	5.7578566584	-0.9454349782	0.096793349
C	4.8131038495	-0.7571423115	-3.2740839054
H	6.077131955	0.4308998393	-1.9677782706
H	6.6834617026	-1.1771524358	-2.2913780738
C	4.22786878	-2.1652087844	-3.2997092711
H	3.9989069623	-0.0246684435	-3.1961857678
H	5.3448491335	-0.545427663	-4.2065522363
C	3.4377958087	-2.4646161312	-2.0243829897
H	3.5722550405	-2.3034638208	-4.1641162632
H	5.0441943724	-2.8916799003	-3.3937263154
H	3.1662926089	-3.5229304641	-2.0016838677
H	2.4926835509	-1.9009193355	-2.0264677426
C	3.5118945969	0.3901043893	-0.1621386892
C	4.4419280226	1.5192961516	0.0291873822
H	3.0460379748	0.307417366	-1.1511845862
O	2.9251672653	-0.1082545476	0.8469993639
C	4.7331511424	2.3645453512	-1.0442380778
C	5.5782843055	3.4570573839	-0.8725369611
C	6.123070805	3.6868650955	0.3852963351
C	5.8409754771	2.8607995939	1.4728194
C	4.9940497925	1.7779629472	1.2887040215
H	4.2884206641	2.1746585008	-2.0177528532
H	5.8060613685	4.1198256668	-1.6998039892
Cl	7.1865746436	5.0547411861	0.6159346389
H	6.2763939262	3.0684550074	2.4439935018
H	4.7514665252	1.1263569781	2.1224007769
H	2.0766733867	-0.9672905292	0.5801716439
H	-2.1508670769	3.2501252538	1.0564678561
H	-4.9090552013	3.5894717975	-0.0764514592

TS1b

C	3.8354123634	1.165295488	-2.5414085242
C	3.1533703504	1.4875803915	-1.1978455066
O	1.9322554637	0.7346929472	-1.2583165195
C	2.5146052994	-0.5368093707	-1.5347954646
C	3.2915042044	-0.2543272341	-2.8258089131
C	3.8381633748	0.7742882404	-0.0150457192
C	3.474508363	-0.7257065053	-0.2964470656
C	4.6994407209	-1.565443661	-0.6695050527
O	4.5703777813	-2.839452339	-0.3200390357
C	5.6723056175	-3.6877613827	-0.6851358623
C	5.2867096141	1.093239994	0.2898022552
O	5.7087931727	2.1726764535	-0.3657526306
C	1.465449869	-1.6113271147	-1.6165750702
N	0.7947489529	-1.7292613989	-0.3383863148

C	1.4645447652	-1.6650182101	0.8152067258
N	0.8141102954	-1.9376600408	1.9441887049
C	1.3521568487	-1.7718776031	3.2934626675
C	2.6162143373	-0.9319952345	3.2454035458
C	3.4938871678	-1.4362546999	2.1110558853
N	2.7668752484	-1.3276634697	0.8348099675
O	5.6485717186	-1.1123435863	-1.2649031314
O	5.9553516536	0.499055047	1.1050410006
H	-0.2201031682	-1.8279857139	-0.3125542265
H	-0.1568551415	-2.2503706969	1.844211247
H	1.5690506593	-2.7594538881	3.7133427312
H	0.5812975258	-1.3009361642	3.9072677267
H	4.4070056597	-0.8454181583	2.0203004038
H	3.7763344814	-2.4824189666	2.2699747789
H	1.9345587804	-2.5574179789	-1.9128263317
H	0.7226846498	-1.3397999052	-2.3691494492
H	3.1482496397	-1.0140024075	4.1950996248
H	2.372095446	0.1221211048	3.077277844
H	2.9351955557	2.5389120183	-1.0131600563
H	5.4135472643	-4.6794172115	-0.3201009985
H	6.5899071483	-3.3317798713	-0.2138641145
H	5.7953559886	-3.6978010037	-1.7695096596
H	4.0615434366	-0.9877137516	-3.0587536272
H	3.5204366768	1.8767325257	-3.3062118784
H	3.2932793337	1.0592729724	0.8931958874
C	7.0539728263	2.5813845667	-0.0773509114
H	7.2337445781	3.4613460802	-0.691993782
H	7.1562505483	2.8271250608	0.9811892495
H	7.7521929198	1.7838177121	-0.3374533986
O	-1.9599326485	-2.564461729	1.6246643158
C	-2.6367764471	-2.1181169573	0.7050459416
O	-2.1015823314	-1.6269009581	-0.3744536789
C	-4.1587956024	-2.2518911941	0.8225908166
N	-4.9699724797	-1.5819770953	-0.1970782568
C	-5.4734218208	-2.5380456894	-1.2030146511
C	-4.692867006	-3.8139545239	-0.9002551172
C	-4.5205335034	-3.7356986706	0.6165065081
C	-5.432916492	-0.3235870664	-0.0724262759
H	-5.3045056823	-2.1440080491	-2.2078213677
H	-6.5458551059	-2.7094942862	-1.0612644513
H	-3.7166463432	-3.7920316332	-1.395043559
H	-5.2286080696	-4.7057376184	-1.2280470431
H	-5.4697360944	-3.9424406159	1.1190503288
H	-3.755461437	-4.4005849705	1.0181437048
H	-4.430068697	-1.8883995792	1.8166815199
C	-6.6307766748	0.075040951	-0.8979836816
C	-7.3979231567	1.2442745075	-0.2722287423
H	-7.2993095597	-0.7793246093	-1.0227007489
C	-6.471177048	2.3973077975	0.1034770391
H	-8.1709745958	1.57795915	-0.9703158347
H	-7.9106912414	0.8872790114	0.6292785769
C	-5.4013359348	1.9224918162	1.0859343875
H	-6.0000335794	2.795174572	-0.8041452518
H	-7.0454272805	3.2160652848	0.5474480995

C	-4.7401943818	0.6366505449	0.6492503517
H	-4.6411353408	2.6960911797	1.23649343
H	-5.8614530105	1.7630426179	2.0712811846
H	-3.8911040888	0.3090101787	1.2476882073
C	-3.5700705443	1.0519937493	-1.2445892239
C	-2.5455906258	1.9262602107	-0.6203367533
H	-4.4105965788	1.6015508586	-1.6833699866
O	-3.2967458614	-0.0790097857	-1.7591017023
C	-1.2651977501	1.4637864593	-0.2972555413
C	-0.3203030551	2.3272369902	0.2419891873
C	-0.6604748166	3.6610873369	0.4446855235
C	-1.914748109	4.1560040614	0.104483285
C	-2.8503676742	3.280379877	-0.4342075192
H	-0.9908293814	0.4309216718	-0.4803490537
H	0.6750739345	1.9690820215	0.4788841781
Cl	0.5336797239	4.7471645874	1.1182358246
H	-2.1505121543	5.2047528714	0.247401692
H	-3.8262612358	3.6563702665	-0.7296737906
H	-2.6991522598	-0.9270941026	-1.0336732372
H	-6.2771331493	0.341036111	-1.9055452575
H	2.5640714532	-0.2440638378	-3.6413371925
H	4.9217487467	1.1894392529	-2.4628831856

TS1c

C	-4.0172753903	3.1720795433	-0.9676510754
C	-4.0996893769	1.8597620932	-1.7711861138
O	-2.8022770576	1.2746828644	-1.5878697726
C	-2.7925253383	1.2988682097	-0.1633810936
C	-2.9993497736	2.7901746977	0.1307593895
C	-4.9658182956	0.8009479056	-1.0608064443
C	-4.076158609	0.4463326513	0.1804976791
C	-4.6990717702	0.904931052	1.5022157863
O	-4.316833139	0.1572018412	2.5313743182
C	-4.8229675897	0.5731679865	3.8108580744
C	-6.4274580223	1.1042384047	-0.8074946612
O	-6.8397700021	2.1775429589	-1.4803122373
C	-1.5329005926	0.7004632351	0.3988639309
N	-1.4580501673	-0.703778692	0.0561038152
C	-2.5305844943	-1.4961564426	0.0810591929
N	-2.350118026	-2.8094736864	-0.0494411551
C	-3.415304225	-3.8019378998	-0.1718723244
C	-4.723618267	-3.1122913055	-0.514163709
C	-4.8952977429	-1.9126585084	0.4027963155
N	-3.7676733864	-0.9829355301	0.2243788317
O	-5.4101347218	1.87796029	1.5926054698
O	-7.1626970624	0.4020075188	-0.1510836395
H	-0.5359490943	-1.1308577938	-0.0766609478
H	-1.3772020909	-3.1425468922	-0.0562612585
H	-3.5100443143	-4.3457436189	0.77404931
H	-3.1242306997	-4.5140681758	-0.9471209702
H	-5.8117244657	-1.3651661113	0.1758367404
H	-4.9360204918	-2.2257168984	1.4519936111
H	-1.5162081675	0.8536161644	1.4855199357
H	-0.6678514473	1.2066130125	-0.0344755818

H	-5.5540569463	-3.8077273643	-0.3781612783
H	-4.7202170323	-2.7780604686	-1.5565341198
H	-4.3168492295	1.9652956148	-2.832792512
H	-4.4254590798	-0.1396387919	4.5303589308
H	-5.9139168211	0.5486557284	3.8078545268
H	-4.4786917832	1.5831086185	4.040691494
H	-3.3341192096	3.008728401	1.1435100605
H	-3.6444543077	3.9803153881	-1.5987619545
H	-4.9717215746	-0.0906861101	-1.6983096846
C	-8.2340365388	2.4892221644	-1.3470804909
H	-8.3956585567	3.3832096953	-1.9464330308
H	-8.8440385138	1.6640610366	-1.7192845622
H	-8.4774891386	2.6811486683	-0.3005313353
O	0.3303703551	-3.7620802383	-0.0464022346
C	1.3054643041	-3.0123771629	-0.0981629931
O	1.1885351924	-1.7296945796	-0.1889562752
C	2.70274131	-3.6643740523	-0.0821941166
N	3.787118306	-2.7411017715	0.2477101855
C	4.2351467766	-2.9198124128	1.6408956064
C	3.2252416424	-3.9015681277	2.2315179807
C	2.7996983993	-4.7268283543	1.018306672
C	4.4908891217	-1.9901583977	-0.639823805
H	5.253760213	-3.3283461733	1.6379007448
H	4.2568091815	-1.971768252	2.1852726457
H	3.6596218485	-4.4997742755	3.0337351493
H	2.3643376264	-3.3569587528	2.6345316478
H	3.5812548318	-5.4447364942	0.7534860283
H	1.8560593198	-5.2554720534	1.1462754911
H	2.8369355454	-4.1093072252	-1.0711556514
C	5.4303440446	-1.0722191971	-0.2187329557
C	6.2909940463	-0.2886086941	-1.1765095521
H	5.7542947026	-1.0936930837	0.8180566456
C	5.6184689887	-0.1194714885	-2.5359661533
H	6.5213539385	0.690003675	-0.7373550196
H	7.257789569	-0.7958792966	-1.3065634257
C	5.1354124822	-1.4741198015	-3.0387506206
H	4.758458127	0.5556460925	-2.4401682714
H	6.312791892	0.3350363808	-3.2489454616
C	4.095642155	-2.0749397697	-2.091735907
H	4.6979863272	-1.3918695147	-4.0378035173
H	5.9906452447	-2.156519831	-3.1158787021
H	3.9187168924	-3.1189301519	-2.3627736221
H	3.1327365251	-1.5547072205	-2.2082200454
C	3.5401158941	0.2937941209	0.3074322088
C	4.2849230557	1.5527023331	0.3617130717
H	3.5244075773	-0.3235000358	1.2089559483
O	2.6210357759	0.1326957896	-0.5550850099
C	4.1305361315	2.5194401824	-0.6372001832
C	4.8483913729	3.7071216502	-0.5754541587
C	5.7123178439	3.9134326557	0.4967169174
C	5.8744181783	2.9685758768	1.5083009374
C	5.1561176134	1.7841668876	1.4321488804
H	3.4402051886	2.3411342919	-1.4551659402
H	4.7380783515	4.4645821651	-1.3434593848

Cl 6.6170432903 5.4034388264 0.5815161443
H 6.5508540492 3.1598863226 2.3335166502
H 5.2738550988 1.0284725033 2.2045745876
H 2.0152155422 -0.845654865 -0.3699040567
H -2.0370874101 3.2829668903 -0.0296879277
H -4.9847229088 3.4617871753 -0.559112612

TS1d

C 2.5641742378 2.285328187 -1.3625432314
C 2.0562769007 1.8607916047 0.0276857458
O 1.1961876583 0.7458345309 -0.2606487715
C 2.1430370206 -0.0265810429 -0.9979283733
C 2.5318228393 0.9391141423 -2.122081959
C 3.1427577379 1.1400607668 0.8501702481
C 3.29355724 -0.2102003559 0.064492651
C 4.6576558079 -0.3446306398 -0.6167225303
O 5.0583716767 -1.6058438736 -0.7162368101
C 6.3120415644 -1.796894628 -1.393807274
C 4.4137234576 1.8867728811 1.1956550312
O 4.3018172901 3.1943849428 0.9699649796
C 1.5587395161 -1.3411615579 -1.4322314964
N 1.1713474709 -2.0995868335 -0.2632343365
C 1.931493064 -2.1357688137 0.8307757885
N 1.5834553045 -2.9576894299 1.8205582061
C 2.2189203765 -3.0121657642 3.1366435853
C 3.0929454517 -1.7871701934 3.3411879015
C 3.9202135825 -1.5613356704 2.0856055174
N 3.028425399 -1.3606124756 0.9311199929
O 5.2631206062 0.603518715 -1.0587139666
O 5.3831164833 1.374266424 1.7075181037
H 0.3051307481 -2.6498280628 -0.3019002691
H 0.7036732528 -3.4639781737 1.6878648569
H 2.8219612282 -3.9234566628 3.2037666144
H 1.4282639716 -3.0691420504 3.8876088034
H 4.547635312 -0.6729250587 2.1756626133
H 4.5697866529 -2.419824751 1.8850065764
H 2.2929042141 -1.8812150967 -2.0432755057
H 0.6681916178 -1.1666418294 -2.04103224
H 3.7499081517 -1.9428532934 4.1990141917
H 2.4757861281 -0.9035882387 3.5342682086
H 1.5111446667 2.6205628217 0.5867937007
H 6.4867326835 -2.8707424095 -1.391798815
H 7.1087677421 -1.2768237965 -0.8593741284
H 6.2475169146 -1.4197185039 -2.4158532929
H 3.4627119787 0.6896812947 -2.6281690364
H 1.8828539498 3.0132504146 -1.8065552486
H 2.6974766588 0.8853665843 1.819262184
C 5.4426073945 3.9829247654 1.3403138444
H 5.1855383062 5.01025759 1.0893157734
H 5.637276624 3.8876420998 2.41011091
H 6.3204833766 3.6597674866 0.7780935386
O -1.1856209585 -3.716521891 1.5689710499
C -1.8769739301 -3.3252007034 0.6304040864
O -1.4035768019 -3.0665427639 -0.5479162579

C	-3.3943040758	-3.2391792595	0.8311874413
N	-4.0945528392	-2.3386410657	-0.0892425847
C	-4.8627917332	-3.0851769789	-1.1036228721
C	-4.3117559448	-4.5029724477	-0.9873441319
C	-4.0087056963	-4.6149502779	0.5069104707
C	-4.2503396395	-1.0159480543	0.1520113892
H	-4.7225872155	-2.64205303	-2.0922101407
H	-5.9314511586	-3.0711560042	-0.8586545398
H	-3.3904795997	-4.6018222841	-1.5683778831
H	-5.0287843955	-5.247634109	-1.3361118164
H	-4.9360096436	-4.7327885983	1.0750523802
H	-3.3355961697	-5.4345028919	0.7650079577
H	-3.5720730025	-2.9388132454	1.8666905568
C	-5.3050334234	-0.2819782732	-0.635075853
C	-5.8471520523	0.9247426264	0.1349315306
H	-6.123922511	-0.9573786362	-0.8910893819
C	-4.716052915	1.8352506155	0.600269051
H	-6.5463911427	1.4734839843	-0.5027500643
H	-6.4144277482	0.565479978	1.0023204188
C	-3.7286471449	1.0723067508	1.4863268246
H	-4.201969805	2.2384235226	-0.2789115827
H	-5.1172054909	2.6897876689	1.1540135273
C	-3.404391323	-0.3152286008	0.9873544377
H	-2.8061105871	1.6524488413	1.6064018909
H	-4.1487404696	0.9791144996	2.4982173139
H	-2.6900591667	-0.8762172608	1.5870192191
C	-1.8509621161	-0.1844796116	-0.8789623703
C	-2.0217076131	1.2053411722	-1.2909888373
H	-1.143970011	-0.3910299405	-0.0700375786
O	-2.1645859996	-1.1214530232	-1.6871596402
C	-1.4647626149	2.229929092	-0.5162404882
C	-1.6243095056	3.5578329264	-0.8896269475
C	-2.3371147963	3.8438763715	-2.0518509722
C	-2.8762698625	2.8400653266	-2.8543354422
C	-2.7108579378	1.5165589646	-2.4705959061
H	-0.9038890629	1.9811196204	0.3788854493
H	-1.2015311562	4.3587083411	-0.2930788907
Cl	-2.5456157157	5.5101039093	-2.529212048
H	-3.4101732743	3.0936366188	-3.7632656184
H	-3.1089271702	0.716718167	-3.0862545027
H	-1.899712936	-2.1118694124	-1.2058619746
H	-4.8558546824	0.0423563302	-1.5866930919
H	3.5633492712	2.7173857047	-1.3171008779
H	1.7194895087	0.917118286	-2.8532359817

B4

C	-0.0172633456	0.0011455514	0.0699235297
O	0.0147889083	-0.4370724472	1.1969498885
H	0.8549341456	0.5024998681	-0.3755692875
C	-1.271384621	-0.0453171117	-0.7487741363
C	-1.3164091998	0.0091351978	-2.1454309606
C	-2.5053168743	0.0804297349	-2.8555773071
C	-3.7043009715	0.0803973394	-2.1491047105
C	-3.6949926788	0.0107074338	-0.7580172015

C   -2.4881459289   -0.0463809153   -0.0666979022
N   -0.0758764545   -0.0784666784   -2.9219517728
H   -2.4847336845   0.1123542532   -3.9383735454
H   -4.6431293711   0.1260875449   -2.6897802303
H   -4.630279091   0.0072687073   -0.2086652698
H   -2.4683686171   -0.075463028   1.0176777715
O   0.8722217706   -0.6538593799   -2.409324547
O   -0.0647349863   0.4066179297   -4.0381993185

IIa(NO_2)

C   4.144170436   3.4031100809   0.0588510216
C   3.845090261   2.349949432   1.1431779754
O   2.6378702533   1.7274649557   0.6796118972
C   3.1028942412   1.3768826202   -0.6203207062
C   3.5169853417   2.7397984615   -1.1886600528
C   4.8050119754   1.1462741889   1.0609437578
C   4.3535701986   0.4751613175   -0.2823232775
C   5.4164800663   0.5811264903   -1.379740996
O   5.3413658726   -0.4106833223   -2.2594146809
C   6.2844056151   -0.3404246194   -3.3424524232
C   6.2881548489   1.3799028857   1.2580504136
O   6.542582782   2.5924603552   1.7467243792
C   2.0552362756   0.6363258052   -1.4049758376
N   1.7522928145   -0.620225099   -0.7504743123
C   2.7039730893   -1.3790243386   -0.2021917138
N   2.3813716686   -2.6002987019   0.2214656396
C   3.263852892   -3.5042217937   0.958340185
C   4.4621999536   -2.7351245873   1.4839727926
C   5.0060830947   -1.8590873282   0.3675803083
N   3.9625061125   -0.9213302183   -0.0815778776
O   6.1923084724   1.5045335498   -1.45526833
O   7.1374438973   0.534895733   1.0865261135
H   0.7894654451   -0.9408734761   -0.7079861702
H   1.4469220521   -2.9389625932   -0.0175901846
H   3.5911113961   -4.3056717768   0.2881804796
H   2.6866592972   -3.9506860437   1.7708071054
H   5.8569260703   -1.2651094382   0.7052119312
H   5.3283088183   -2.4664147226   -0.4850996484
H   2.4134512316   0.4771260792   -2.429297617
H   1.1415755167   1.2314035852   -1.4443937676
H   5.2304833805   -3.4352121613   1.8175077375
H   4.1744295806   -2.108101206   2.3337952991
H   3.7053531647   2.7330905025   2.152707038
H   6.0987185733   -1.2226180158   -3.9515985467
H   7.3034489442   -0.3501997038   -2.9521301413
H   6.1216159139   0.5688501602   -3.9237098368
H   4.1828442356   2.687681271   -2.0484905862
H   3.6540170503   4.3471730268   0.3015871191
H   4.5212822805   0.4551706006   1.8631432254
C   7.9227607514   2.862995761   2.0318883599
H   7.9516825453   3.8815159762   2.4142433569
H   8.2988507873   2.1629575142   2.780235287
H   8.5175863161   2.779115958   1.1206257008
O   -0.2866832652   -3.5886766066   -0.3867298493

C	-1.2434075784	-2.8518536423	-0.5243268306
O	-1.0707131111	-1.5363698482	-0.5774508961
C	-2.6587269975	-3.3946884236	-0.6921628016
N	-3.6753966175	-2.3780194649	-0.4554850672
C	-4.517310528	-2.1795528763	-1.6352888852
C	-3.6167587282	-2.6339623994	-2.7762674767
C	-2.8899530264	-3.8295614712	-2.1590970381
C	-4.1520660657	-2.0646721337	0.8169657622
H	-5.4296812801	-2.7923468473	-1.5733249545
H	-4.8156603993	-1.1302543921	-1.7120687446
H	-4.1768724405	-2.8982588446	-3.6745378882
H	-2.9079428092	-1.8382506041	-3.0303668449
H	-3.543306164	-4.7057104888	-2.1569300173
H	-1.9594771087	-4.0978814824	-2.6630603038
H	-2.7347653682	-4.2555857111	-0.0224679472
C	-5.3378581794	-1.4459010772	1.0056515166
C	-5.8287563228	-0.9734274037	2.350383778
H	-5.9993405942	-1.2808847507	0.1599879516
C	-4.7181734417	-0.9365724284	3.400365536
H	-6.2866544113	0.0190389098	2.2466685913
H	-6.6373080772	-1.6307515622	2.7021087238
C	-3.893470127	-2.217387439	3.3257408782
H	-4.0535257901	-0.0801140663	3.2237610521
H	-5.1471975906	-0.803431526	4.398076448
C	-3.204242379	-2.3346505761	1.9663331297
H	-3.140019739	-2.2475705149	4.1181842663
H	-4.5534584883	-3.0815028977	3.4718570821
H	-2.7622595247	-3.3316616579	1.8689508479
H	-2.3694631877	-1.6194695756	1.9067850553
C	-3.4952393593	0.7891724145	0.0893558945
C	-4.6969685178	1.6639287398	-0.0250931988
H	-3.1342207383	0.5291959306	1.0901249178
O	-2.9278293176	0.3749471671	-0.910074872
C	-5.3161086491	2.3117752184	1.0514719426
C	-6.5022008451	3.0177396206	0.9183411565
C	-7.085067528	3.1149149759	-0.3413420351
C	-6.4847385945	2.4991621348	-1.437606937
C	-5.3086453304	1.7749773052	-1.276128042
N	-4.7065685362	2.2972013497	2.3863694778
H	-6.9429948831	3.4959932009	1.7849892688
H	-8.0049314532	3.6761229566	-0.4626434413
H	-6.938370833	2.5771598806	-2.4196737245
H	-4.8457511423	1.2708884772	-2.1181161746
H	-1.9120061461	-1.0192502237	-0.6869407507
H	2.5995826311	3.2507980518	-1.491563889
H	5.2132179734	3.5798739806	-0.0540671381
O	-5.4489684446	2.3089132815	3.350255723
O	-3.4866734588	2.293311176	2.4537543316

IIb(NO_2)

C	-5.5514768738	-1.9328406202	-1.5275559455
C	-5.1372215473	-2.1213053969	-0.0555533852
O	-3.8296222887	-2.7061516025	-0.1491524151
C	-3.2470181528	-1.7127635532	-0.9865166007

C	-4.1766991412	-1.7365188825	-2.2063004597
C	-4.7754374694	-0.7828447576	0.6188505592
C	-3.4465771586	-0.4079655116	-0.1212988553
C	-3.5902092872	0.820494009	-1.0232560279
O	-2.4346854914	1.451293687	-1.2087541082
C	-2.4891058769	2.5760643853	-2.1034530964
C	-5.8370781272	0.2913014398	0.7318502016
O	-7.0516224645	-0.1730373877	0.4451028503
C	-1.7993249883	-1.9973232981	-1.2742706461
N	-1.0365419531	-1.9482319034	-0.0425834977
C	-1.2592121944	-1.0153889703	0.8827427337
N	-0.3768465171	-0.8723101127	1.8762842816
C	-0.5427164237	-0.0202511853	3.0543205885
C	-1.9893930027	0.4268917545	3.1568407813
C	-2.4563483716	0.8856775418	1.7856875047
N	-2.3432711141	-0.2238020401	0.8231927917
O	-4.629585501	1.130080406	-1.5549497996
O	-5.6253853195	1.4141928256	1.1305791819
H	-0.2095408657	-2.5426707346	0.0310051543
H	0.4739173144	-1.421346651	1.8033897496
H	0.1197013858	0.8474119174	2.9598620762
H	-0.2374242596	-0.5936037656	3.9317674117
H	-3.5016221603	1.1984098682	1.8040946391
H	-1.8533033362	1.7289463454	1.4300250653
H	-1.4267726853	-1.269563286	-2.0059869439
H	-1.702986049	-2.9981281327	-1.6982682005
H	-2.0704595131	1.2470910006	3.8723842693
H	-2.6226133228	-0.3964894534	3.5018046514
H	-5.7884096419	-2.7544163809	0.5447903586
H	-1.4783790014	2.9781912053	-2.1349734051
H	-3.1882514281	3.3226757077	-1.723433108
H	-2.80270486	2.2500560882	-3.0967007352
H	-4.1143447457	-0.8533264783	-2.8403477922
H	-6.0580103702	-2.8259291202	-1.896458247
H	-4.4967832913	-1.0101678148	1.6544237389
C	-8.1227534531	0.7700850042	0.5966094745
H	-9.0287984048	0.2356097137	0.3174794584
H	-8.1834250275	1.111150697	1.6316875573
H	-7.9649372457	1.6258457626	-0.0622758456
O	1.3930530473	-3.4300767162	-0.4986553072
C	2.3570714292	-2.8179057457	-0.0869771679
O	2.2325340825	-1.9803543919	0.9408019814
C	3.7615343753	-2.9922127959	-0.6514186218
N	4.606455768	-1.8591638245	-0.3294942897
C	5.8074276714	-2.2715669424	0.4043518656
C	5.3397264871	-3.5512633909	1.0878936736
C	4.4652909463	-4.1991144295	0.0118230648
C	4.5195336631	-0.6818214197	-1.0793607706
H	6.1064921676	-1.4975648445	1.1150140406
H	6.6505058305	-2.4758776014	-0.2712370096
H	4.7427363037	-3.3012203475	1.9717527752
H	6.1692597757	-4.1883755185	1.3986687045
H	5.0912365024	-4.6875053649	-0.7392716573
H	3.7568647146	-4.9353888436	0.397004335

H	3.6429911903	-3.1369805388	-1.7320674384
C	5.6673352691	0.2880731013	-0.9095601996
C	5.6783309805	1.3652052002	-1.9933867461
H	6.6131507442	-0.2632636264	-0.9277299473
C	4.2852502827	1.957456535	-2.1770085163
H	6.4016787846	2.1415018189	-1.724977456
H	6.0126576224	0.9229927536	-2.9403029014
C	3.307400269	0.8802034135	-2.6425736169
H	3.9474650536	2.3643633453	-1.2167094316
H	4.3035696196	2.7869271494	-2.8909757751
C	3.467407414	-0.4103845575	-1.8790587031
H	2.2766991829	1.2428361553	-2.5330014064
H	3.4454751643	0.69789494	-3.7183900042
H	2.6735628822	-1.1433228517	-2.0056240758
C	4.1409661567	0.8545788071	2.3571644772
C	3.4172238401	1.8513443617	1.5172218145
H	4.7787705903	1.2383312176	3.1642996343
O	3.9957792293	-0.3437788209	2.2125494045
C	2.2171539068	1.4710289969	0.9149291142
C	1.4417563911	2.3978741965	0.2211586869
C	1.850719149	3.7257706188	0.1290023242
C	3.0502676336	4.1306189731	0.711547978
C	3.8133304393	3.1870121156	1.3795742789
H	1.8841964813	0.4431853686	1.0125815923
H	0.5138218675	2.0812128676	-0.243729428
H	1.2473003366	4.449113065	-0.4083429124
H	3.4073332773	5.1500386638	0.6250908209
N	5.1287205626	3.6112297921	1.8693433882
H	3.0731014554	-1.4919527252	1.1135333741
H	5.6086217945	0.7574413528	0.083137491
H	-6.2097576921	-1.074906435	-1.6590072681
H	-3.8988952039	-2.6094648974	-2.8022800198
O	5.2724372477	4.7757990274	2.1861731445
O	6.0133645386	2.7674514731	1.9046797422

IIc(NO_2)

C	-3.5886199069	2.5569286611	0.6973701532
C	-3.1109660079	1.8612030829	-0.59116293
O	-1.8686462296	1.2524409075	-0.2003851757
C	-2.3670745665	0.5296726884	0.9245614609
C	-2.9636425691	1.6506192483	1.7826041713
C	-3.9287856784	0.5925349468	-0.9017090901
C	-3.4762623453	-0.3666350031	0.2539746739
C	-4.6071896767	-0.6741833172	1.2401701198
O	-4.4902181487	-1.8747387363	1.7936652116
C	-5.4983013457	-2.202019625	2.7656653498
C	-5.4165240534	0.7264220405	-1.1499042624
O	-5.7803647638	1.9936365774	-1.3352439335
C	-1.2930051767	-0.289874511	1.5833155426
N	-0.7825431384	-1.2584141405	0.6332631724
C	-1.6072712119	-1.9515775332	-0.1614563052
N	-1.1204820235	-2.9858096384	-0.8426617021
C	-1.8369793078	-3.7392692485	-1.8725160096
C	-3.0768450568	-2.9749127623	-2.2982996512

C	-3.7944499534	-2.4830681077	-1.0524898121
N	-2.9014213744	-1.6046974579	-0.2762456229
O	-5.4721646929	0.1245117392	1.5123617318
O	-6.171076855	-0.2127903097	-1.2635486013
H	0.1784863634	-1.5748580382	0.7229359552
H	-0.135122851	-3.2141681452	-0.6986693497
H	-2.1125573958	-4.7184512315	-1.4684357417
H	-1.1522611211	-3.8927396396	-2.7092583723
H	-4.6842198179	-1.9041959578	-1.3058406574
H	-4.1012635986	-3.3225815271	-0.4198126952
H	-1.7052745231	-0.7755887581	2.4765908575
H	-0.4737722482	0.3620160341	1.8914909637
H	-3.7340525875	-3.6301607159	-2.8728939547
H	-2.8053254557	-2.1203589281	-2.9261306997
H	-2.95509175	2.5075794398	-1.4531343702
H	-5.2614169834	-3.2073650273	3.1076570494
H	-6.4863943226	-2.1760691525	2.3032764645
H	-5.4615395331	-1.4952072336	3.5963548937
H	-3.6679783817	1.3124818589	2.5404128762
H	-3.196228366	3.5737802869	0.7474840938
H	-3.5279618017	0.1690727374	-1.830050699
C	-7.1665118646	2.1971326483	-1.6478938447
H	-7.2904240411	3.2731002115	-1.7539864822
H	-7.4229780848	1.6888660692	-2.5791698114
H	-7.7931746685	1.8176446884	-0.8389446111
O	1.7250697581	-3.4144502683	-0.8975808472
C	2.5337082891	-2.7439600505	-0.288944394
O	2.154905241	-2.0211174594	0.7619467531
C	4.0129924259	-2.727135824	-0.6496774262
N	4.706945839	-1.5816263587	-0.0634685363
C	5.8185845989	-2.0255864535	0.7795557055
C	5.3744333965	-3.4114666304	1.2254313039
C	4.7198967746	-3.9629045712	-0.0417364868
C	4.7978294783	-0.3652684671	-0.7514779515
H	6.7528303953	-2.0693236736	0.1989166847
H	5.9626748275	-1.3315374026	1.6112323692
H	6.2049343523	-4.0274098683	1.5738587315
H	4.6423693681	-3.3236436841	2.0355054377
H	5.4863595162	-4.2994453424	-0.7442171812
H	4.0291766077	-4.7900245032	0.133749716
H	4.0598587711	-2.755009451	-1.7417085112
C	5.7976514811	0.5145296699	-0.5351291777
C	5.9351533195	1.8064972891	-1.2994147473
H	6.5806204832	0.2788967843	0.1789290222
C	5.0749597676	1.8186640077	-2.5601052839
H	5.651813559	2.6605719388	-0.6673223747
H	6.9904098292	1.9620518996	-1.5516398345
C	3.6608490875	1.3762394197	-2.2004167611
H	5.4968803591	1.1306724618	-3.3039321161
H	5.0672274852	2.8182587503	-3.0055582429
C	3.6586390466	-0.0701015287	-1.7042201847
H	2.9820407046	1.4683250154	-3.0535493937
H	3.2798087267	2.035288142	-1.4104341955
H	3.7117393721	-0.753080857	-2.5620022795

H	2.7062290988	-0.2823609602	-1.1979065892
C	4.139178931	0.7226674003	2.259045069
C	3.3856640686	1.7862106453	1.5310695155
H	5.0510970222	1.0064094129	2.7948528664
O	3.7221597799	-0.4202063486	2.3271990276
C	3.8123740287	3.1109128418	1.3553629627
C	3.0100090581	4.0774569874	0.763253079
C	1.7384460399	3.7258715429	0.3221330381
C	1.2792286926	2.4200843635	0.4865528784
C	2.0972034459	1.4705923652	1.0890588308
N	5.1483872391	3.5494790545	1.7868850244
H	3.3867106732	5.0866606264	0.6491435477
H	1.110103022	4.4751353611	-0.1467076583
H	0.2824735835	2.1404022423	0.1550138335
H	1.7352184885	0.4592797667	1.2433443418
H	2.9047911372	-1.4924483979	1.1309639498
H	-2.1274783935	2.1475203743	2.2817215276
H	-4.6756031487	2.5949602082	0.760162662
O	5.2938029215	4.727328894	2.0567436126
O	6.0415055665	2.7184164145	1.8389423269

IId(NO_2)

C	2.7019486064	2.5919119814	-1.9568057127
C	2.0880621988	2.1581978575	-0.614987205
O	1.1231262793	1.1587949835	-0.9981512667
C	2.0190598946	0.3200436486	-1.7346635381
C	2.5742685576	1.2922880379	-2.7818026138
C	3.0576880022	1.2842905395	0.203311163
C	3.0699155009	-0.0483934819	-0.6222280172
C	4.4515121122	-0.3570639225	-1.2082872718
O	4.7600573529	-1.645989046	-1.1561853589
C	6.0481040226	-1.9867615872	-1.6960394342
C	4.3986286541	1.8510755572	0.6132801576
O	4.4439805328	3.1753285343	0.4868292983
C	1.3264773805	-0.8868240485	-2.3063574765
N	0.8279163286	-1.7232138411	-1.2350103582
C	1.4702927254	-1.865513794	-0.0725058579
N	0.9794535508	-2.7198094971	0.824062191
C	1.3499106543	-2.7634427587	2.2406731874
C	2.2816863265	-1.6096684749	2.5699177807
C	3.2960146371	-1.4727958875	1.4444447507
N	2.5819159034	-1.1618470437	0.1948577574
O	5.1580351675	0.4975404826	-1.6891389007
O	5.2901455546	1.1884138971	1.0941735422
H	-0.0615660957	-2.1974849503	-1.3536728474
H	0.151881552	-3.2480520119	0.5480318641
H	1.8369177963	-3.721737688	2.4447962935
H	0.4300596155	-2.7106801621	2.8284067687
H	4.0061818021	-0.665359534	1.6290820934
H	3.8639757624	-2.3989925991	1.3074731175
H	2.0268634592	-1.4309201116	-2.951708507
H	0.4758027677	-0.5646003108	-2.9117654439
H	2.7881273564	-1.8051450222	3.5169598141
H	1.7128026502	-0.678701601	2.6576790017

H	1.6009581653	2.940661588	-0.0326203116
H	6.1480456926	-3.0618038285	-1.5620667617
H	6.8317765972	-1.4563396785	-1.1525086171
H	6.0933648475	-1.7256072496	-2.7546501227
H	3.5032407077	0.9669705498	-3.243975538
H	2.123987245	3.4068585683	-2.3950424087
H	2.5572478853	1.0594476894	1.1516042572
C	5.6564671074	3.7966085388	0.9401778262
H	5.5245804446	4.8628274113	0.7667763893
H	5.8082812289	3.5973194356	2.0024632896
H	6.5075922668	3.4186077031	0.3711842607
O	-1.6800568178	-3.8799782939	0.3864329234
C	-2.4498867872	-3.1674554222	-0.2218559617
O	-2.0198106625	-2.430462392	-1.2478042882
C	-3.9445705535	-3.1066146367	0.0491198407
N	-4.4950774903	-1.8146386028	-0.3371468837
C	-5.5810387688	-1.9812401042	-1.3151555979
C	-5.2050998705	-3.2859132907	-2.0059879985
C	-4.6831466476	-4.132647824	-0.843357161
C	-4.4783889117	-0.7642513998	0.5940909789
H	-5.613602484	-1.1287311063	-1.9973150035
H	-6.5596983446	-2.0773587273	-0.8237883353
H	-4.4150886626	-3.1098704835	-2.7438025853
H	-6.0532683546	-3.7467604402	-2.5147899265
H	-5.5190387578	-4.5437120915	-0.2719007619
H	-4.0406599951	-4.9608640989	-1.1504375041
H	-4.0847851409	-3.3201879512	1.1140471897
C	-5.4258949262	0.3817321096	0.3232424696
C	-5.6924453354	1.2092782168	1.58081802
H	-6.3741878143	0.0023614376	-0.067091163
C	-4.3868787264	1.6005477237	2.2641031795
H	-6.2724063176	2.0987948448	1.3157862063
H	-6.3050415118	0.618151909	2.2733106549
C	-3.5981107546	0.3569659755	2.6757070371
H	-3.7952728688	2.201823515	1.5660286799
H	-4.5803112054	2.2271380516	3.1406466564
C	-3.6296597373	-0.7412672031	1.6420760668
H	-2.5571678861	0.6282339988	2.8942102588
H	-4.0003759248	-0.03362513	3.6218423952
H	-2.9467985106	-1.5703973053	1.8119018918
C	-1.9050894217	0.4747803777	-1.0345866309
C	-1.9359567336	1.7897466123	-0.3378065818
H	-1.0624765229	-0.1871507428	-0.8141068204
O	-2.7602557369	0.1619979463	-1.8441844738
C	-1.1730040747	2.1165618473	0.7920420348
C	-1.2128243191	3.3781230025	1.3707582603
C	-2.0352100797	4.3523749069	0.8114482718
C	-2.7958407483	4.0604610033	-0.3178671222
C	-2.7405762955	2.7910918678	-0.8851823234
N	-0.3125351587	1.1241737413	1.451948019
H	-0.6192720684	3.5844618394	2.2528182939
H	-2.0783399563	5.3369659106	1.2637317108
H	-3.4315914804	4.8212315496	-0.7577592908
H	-3.3148994004	2.5530946626	-1.7744011829

H	-2.7048636065	-1.773171652	-1.5120718588
H	-4.9917015422	1.0101616689	-0.4687501554
H	3.7364896236	2.9148165485	-1.8435883458
H	1.8112093927	1.3895512561	-3.5582847762
O	0.5993599065	1.5501702566	2.1440933421
O	-0.5551898065	-0.0614507551	1.306541117

IIIa(NO_2)

C	-3.5567988137	3.3191787958	-0.5448978356
C	-3.5413004451	2.1146013115	-1.5061260499
O	-2.3365936264	1.4208442513	-1.1518320716
C	-2.6082571722	1.2847659771	0.240386979
C	-2.7949504476	2.7447624334	0.67123
C	-4.5804745743	1.0460726435	-1.1133674354
C	-3.9773123604	0.4970277763	0.2259119955
C	-4.8276760707	0.8625618648	1.4453886513
O	-4.6848576017	-0.0035022072	2.4422836005
C	-5.4107702143	0.3174547625	3.6405105018
C	-6.0477263614	1.4192587759	-1.1173778791
O	-6.2645482591	2.5913820866	-1.7125906504
C	-1.5193327074	0.5301692219	0.9526901188
N	-1.4468280569	-0.8258413109	0.4550112025
C	-2.5416976612	-1.5291151804	0.1728241581
N	-2.4064572238	-2.8251806964	-0.1071054322
C	-3.4789188465	-3.7080428597	-0.556995201
C	-4.6697640459	-2.885294864	-1.0156331173
C	-4.943975523	-1.8070239124	0.0200365286
N	-3.7585945223	-0.9474349214	0.1671842476
O	-5.5020013401	1.8641325691	1.503130222
O	-6.9338427776	0.6990073811	-0.7163098957
H	-0.5217441306	-1.2713519241	0.3217631838
H	-1.4714051542	-3.236415264	0.0469966618
H	-3.77167158	-4.3668000154	0.2678730898
H	-3.0889898505	-4.3302826804	-1.3659412253
H	-5.7777249617	-1.1696404304	-0.2796931027
H	-5.1858730834	-2.2514201049	0.9920471316
H	-1.7162389947	0.5537625352	2.0322472846
H	-0.5590503111	1.0161248545	0.7679004851
H	-5.5438856915	-3.5301367651	-1.1257959375
H	-4.4632501488	-2.4167280266	-1.9833438645
H	-3.5377604037	2.3548379954	-2.5681312318
H	-5.1942793906	-0.4870995099	4.3403931203
H	-6.4802741515	0.3656863005	3.4292375532
H	-5.0718125659	1.2750612113	4.0398348116
H	-3.3106353675	2.872547082	1.6218198531
H	-3.0279790531	4.1644088779	-0.9882736794
H	-4.4939659041	0.2330319814	-1.8431721112
C	-7.6405936485	2.9851244652	-1.8115264895
H	-7.6362367534	3.9533401967	-2.3085896232
H	-8.2021820378	2.2564688075	-2.3989992681
H	-8.0796489544	3.0684015091	-0.8156289512
O	0.1402752284	-3.9580460403	0.3027374836
C	1.1050636978	-3.1794212933	0.2511242105
O	1.0636165267	-1.92032521	0.1357374479

C	2.5002562977	-3.8578886991	0.3440520888
N	3.5577912631	-2.8210674824	0.3031529827
C	4.1295925039	-2.6143985217	1.6691391503
C	3.1746128027	-3.3777520211	2.5797157824
C	2.7046556863	-4.5338377712	1.6998144658
C	3.8263832202	-2.108726151	-0.7398554761
H	5.1322908513	-3.0510218729	1.6700460314
H	4.1796005242	-1.5516627676	1.8910037794
H	3.6731963159	-3.7017834515	3.4936760826
H	2.3292417011	-2.7371423109	2.8506357009
H	3.4822269447	-5.2986608831	1.6202331379
H	1.7791971129	-5.0011593776	2.0360447422
H	2.6115605377	-4.5530269022	-0.4876910548
C	4.7000188863	-0.8894143356	-0.6282661146
C	5.6120943939	-0.7315044415	-1.8583564476
H	5.3268215526	-0.9523833541	0.2636833655
C	4.8843619482	-0.8955315306	-3.1920926998
H	6.1292703493	0.2308654495	-1.798826931
H	6.3915967184	-1.4987793225	-1.7911483436
C	4.1695224243	-2.2413845032	-3.2265581749
H	4.1527096117	-0.0932123615	-3.3472977735
H	5.6093473084	-0.828483697	-4.008215362
C	3.1719886337	-2.3647228576	-2.0652723102
H	3.6196922174	-2.3715044774	-4.1619985227
H	4.9030377363	-3.0533761524	-3.1629658485
H	2.6656275891	-3.3284247006	-2.0902350442
H	2.3956520026	-1.5940832032	-2.1723469762
C	3.7063399662	0.3139685717	-0.3958901496
C	4.5145659508	1.5626001728	-0.0884303909
H	3.1141580235	0.4617339712	-1.3032868361
O	2.8735903121	0.0673926861	0.7088850289
C	4.9735179103	2.4725364741	-1.0431952374
C	5.8166810889	3.5366600168	-0.7429076916
C	6.1933800192	3.7389517362	0.5777548549
C	5.744971072	2.8599408542	1.5616565946
C	4.9303854283	1.7828911103	1.2272606313
N	4.5879090578	2.3458580846	-2.4559434519
H	6.1494609552	4.1967555636	-1.5360457419
H	6.8334884397	4.575783656	0.8339453489
H	6.0380384761	3.0087261208	2.595700092
H	4.5954562379	1.0925653427	1.993839123
H	2.2179834517	-0.6267817215	0.4798165201
H	-1.796961438	3.1819618662	0.7576502472
H	-4.5709977663	3.6292710955	-0.2957304222
O	5.471508219	2.4376712482	-3.2891426855
O	3.4080807978	2.1768373997	-2.7189665335

IIIb(NO_2)

C	-5.8520599353	-2.1172117625	-1.2778631611
C	-5.1353750271	-2.355802303	0.0653035842
O	-3.8071440946	-2.7309988567	-0.3262516677
C	-3.5341028236	-1.5998812003	-1.1484602522
C	-4.67437479	-1.6687079772	-2.1724624737
C	-4.8264013238	-1.0350929565	0.7970827424

C	-3.7309617185	-0.4076215272	-0.1319316422
C	-4.2203245132	0.8491057772	-0.857226033
O	-3.2269189386	1.6757071621	-1.168345366
C	-3.6145972117	2.8477376552	-1.9048909725
C	-5.9790602514	-0.1482340627	1.2177592276
O	-7.1512113635	-0.7744627018	1.1230351264
C	-2.148789441	-1.6470346253	-1.7295736098
N	-1.1609468425	-1.5554312043	-0.6751327677
C	-1.317536088	-0.7271318631	0.3601309251
N	-0.2700260222	-0.5073970447	1.1534077283
C	-0.2864597642	0.2819005286	2.3817468259
C	-1.7169881744	0.4552068449	2.8575101518
C	-2.5728602203	0.8803783542	1.6764763156
N	-2.5007761928	-0.131083729	0.6082124895
O	-5.3757906271	1.0274699907	-1.1625245066
O	-5.8490941454	0.9629651087	1.6791258757
H	-0.211899491	-1.9186764398	-0.8643355282
H	0.6221105103	-0.9580175818	0.8904816062
H	0.1744294126	1.2581192633	2.1899772716
H	0.3229281431	-0.2397799367	3.1234479997
H	-3.6214224654	0.9839072131	1.9605706388
H	-2.2324632772	1.8430387061	1.2778321468
H	-2.0398827063	-0.8332200435	-2.4584460464
H	-2.0109388849	-2.5961358314	-2.2512032141
H	-1.7604659439	1.2164499684	3.6388272032
H	-2.0997575145	-0.4844737946	3.2684699149
H	-5.5683064362	-3.1225544473	0.705711272
H	-2.6999738	3.4183701013	-2.0537479077
H	-4.3399368209	3.4274344294	-1.3316827406
H	-4.0486526287	2.5612710271	-2.8645198762
H	-4.8526437399	-0.7394685206	-2.7114498085
H	-6.3017165684	-3.0437036106	-1.6383359097
H	-4.317945893	-1.2992139127	1.7314391996
C	-8.287931198	-0.02226429	1.5713330734
H	-9.1487188783	-0.6721824271	1.4259366726
H	-8.1784923398	0.2368631036	2.6259782609
H	-8.3923629941	0.8891033502	0.9796549061
O	1.4209849006	-2.4171211557	-1.3622049643
C	2.3432998793	-2.0777568501	-0.6047974815
O	2.2317944189	-1.5038869765	0.5165238616
C	3.770230338	-2.3874887872	-1.1206824894
N	4.7943396616	-2.1941173612	-0.0625038357
C	5.2180065636	-3.5148987646	0.4915979021
C	4.2469406511	-4.5059649688	-0.1401471383
C	3.930692662	-3.8625239038	-1.4887211129
C	5.2758074714	-1.0631171462	0.3283569857
H	5.170092391	-3.4791866449	1.5796034664
H	6.248303667	-3.6966707151	0.1738988391
H	3.339405364	-4.584616672	0.4663734012
H	4.6935138792	-5.4968581993	-0.2266982797
H	4.7696663632	-3.9851898174	-2.1793727032
H	3.0211537938	-4.235764991	-1.9578998142
H	3.9816338958	-1.7247182941	-1.9612024345
C	6.4390780009	-0.9886403259	1.261134828

C	7.6292524303	-0.4581838691	0.427288364
H	6.6748841872	-1.9489174392	1.7157328408
C	7.3015863528	0.9075443202	-0.1718538406
H	8.5060007	-0.3996686714	1.0770817966
H	7.8559172048	-1.1797136315	-0.3671470947
C	6.0212982537	0.869328565	-1.0106673391
H	7.2002434214	1.6366361406	0.6374387461
H	8.1290653206	1.2474885659	-0.80207196
C	4.8227662583	0.2511181958	-0.2528054634
H	5.7405856866	1.8753155681	-1.3364526442
H	6.1935236649	0.2678982769	-1.9109741523
H	4.0038430305	0.0883836162	-0.9558874899
C	4.2866092239	1.1524334991	0.8970464725
C	3.3551800108	2.2301806818	0.3339857586
H	5.1287554973	1.6223012446	1.4063266173
O	3.6153355065	0.3829446864	1.8673760306
C	2.106065894	1.8182737429	-0.1476540625
C	1.1589629649	2.7248947539	-0.6085684509
C	1.4208435224	4.0940377522	-0.5773231369
C	2.6354019689	4.5418188659	-0.0830314717
C	3.5790767807	3.609824454	0.3449732167
H	1.8734010974	0.7562335876	-0.1485010288
H	0.208779304	2.3651943614	-0.9904252768
H	0.6848582938	4.8067775141	-0.9324060896
H	2.8720285634	5.5981426963	-0.034560411
N	4.8447496358	4.1768051409	0.8235411648
H	3.0906385013	-0.309717685	1.41411075
H	6.2007320009	-0.2767725371	2.0576485498
H	-6.6284323959	-1.3570914433	-1.196416599
H	-4.4069069995	-2.442745721	-2.8961909929
O	4.7993111308	5.2376109692	1.4195049141
O	5.8785971096	3.5733554385	0.5799485781

IIIc(NO_2)

C	-3.552529832	3.2595807017	-0.6956139395
C	-3.6179957244	2.0197194916	-1.6083079085
O	-2.390406416	1.3327958796	-1.326002911
C	-2.5498785844	1.2499836237	0.0875389131
C	-2.6987889393	2.7260921116	0.4773230077
C	-4.6255021955	0.9737854254	-1.0918880188
C	-3.9159395588	0.4685926725	0.2118148051
C	-4.6611095644	0.8793438275	1.4845453304
O	-4.4345507618	0.0484670785	2.4956462109
C	-5.0548322741	0.4148644075	3.7393670915
C	-6.0868793776	1.3555093563	-0.9877122794
O	-6.3502011995	2.5064768149	-1.6049087852
C	-1.408073307	0.5212295338	0.7400988361
N	-1.3771914032	-0.8547118021	0.2950131431
C	-2.4918827553	-1.566027208	0.1310006075
N	-2.3805600732	-2.8742966161	-0.0928384623
C	-3.4851332137	-3.7734196781	-0.4136032786
C	-4.7023711496	-2.9691174904	-0.833010473
C	-4.9007668005	-1.8352677791	0.1593032698
N	-3.7048874858	-0.9778036062	0.1821051122

O	-5.3269646357	1.8849838073	1.5641437273
O	-6.9378914261	0.6538898965	-0.4894923872
H	-0.4629368598	-1.328418152	0.199808993
H	-1.4307953058	-3.2776786626	-0.024049949
H	-3.7205481865	-4.3828678994	0.4657818335
H	-3.1546326673	-4.4426420175	-1.2114005895
H	-5.75028526	-1.2083479851	-0.1171075486
H	-5.0791717283	-2.2261439043	1.1674456981
H	-1.5161061492	0.5926485919	1.8301626949
H	-0.4661168102	0.9951986488	0.4555385653
H	-5.5845555068	-3.6122557739	-0.8464821037
H	-4.5618602388	-2.555244919	-1.8367219668
H	-3.6992911906	2.2189274442	-2.6757348923
H	-4.7733456728	-0.3610702042	4.4482335435
H	-6.1389758299	0.4522822759	3.6206959508
H	-4.6876348525	1.3881930084	4.0696824854
H	-3.1386036647	2.8918018145	1.4595754415
H	-3.0577727736	4.0842634857	-1.2110880476
H	-4.6043133318	0.1343781714	-1.7962245489
C	-7.7304269692	2.8982703115	-1.6113057525
H	-7.7664430228	3.845503487	-2.1461134956
H	-8.3349392673	2.1470631514	-2.1229987216
H	-8.0903187098	3.022480001	-0.588325643
O	0.1757145796	-3.9932467778	0.0598208492
C	1.1619530465	-3.2395150809	0.0854906134
O	1.1576291633	-1.9762736612	0.1008702817
C	2.533938762	-3.9739208886	0.1183578296
N	3.6190096356	-2.9824310783	0.2752920157
C	4.0865782806	-2.9567583164	1.693347286
C	3.090911805	-3.8471921336	2.4293371322
C	2.6656016548	-4.8524076683	1.3607638266
C	4.0082845894	-2.1773991294	-0.6578466353
H	5.0965445592	-3.3768281527	1.7037017944
H	4.119395965	-1.9375734164	2.0717261825
H	3.5476024691	-4.3103135513	3.3043629662
H	2.2311991946	-3.2548348165	2.7571996913
H	3.4451363689	-5.6032266988	1.2037345995
H	1.7228072353	-5.3547861125	1.5750154864
H	2.6485071626	-4.5344825291	-0.8087538487
C	4.8581779644	-0.9820560263	-0.2988936642
C	5.7329504763	-0.5112149664	-1.4698811305
H	5.5112374878	-1.2458369683	0.5369429363
C	4.9653195643	-0.4051261818	-2.783273446
H	6.1999370381	0.4397644163	-1.1998643302
H	6.5470535469	-1.2350057989	-1.5953095572
C	4.4173519659	-1.7802999635	-3.1438523489
H	4.1458839333	0.3157690719	-2.6892179292
H	5.6335658157	-0.0498087565	-3.5731176868
C	3.4856579795	-2.331467499	-2.0583823586
H	3.8589953633	-1.7541768119	-4.0831577214
H	5.2546817024	-2.4733792089	-3.2851882524
H	3.2542316623	-3.378555167	-2.2606402945
H	2.5343587066	-1.7850143385	-2.0796218868
C	3.8298650778	0.0967016272	0.2354543933

C	4.4842152096	1.4674561302	0.3391469935
H	3.5136172179	-0.2143688168	1.2338645155
O	2.7025050025	0.1971717299	-0.5991058056
C	4.06164687	2.4731444516	-0.5333575487
C	4.6478046059	3.7362612441	-0.5392377122
C	5.6940832741	4.0336505403	0.3278506096
C	6.1479710437	3.055812822	1.2012516533
C	5.5421290412	1.802676952	1.1997169813
H	3.2500282613	2.2489152364	-1.2137409181
H	4.2824115121	4.4892910668	-1.2298903927
H	6.1533527391	5.0156709928	0.3302385817
H	6.9581681225	3.2516598568	1.8927259418
N	6.0789570567	0.8479108469	2.1777285981
H	2.0932187114	-0.5241761893	-0.342138324
H	-1.6961571637	3.1612497048	0.4697823394
H	-4.542754228	3.5850333785	-0.3788929543
O	7.2274199004	0.9961358951	2.5501896452
O	5.3445137429	-0.0397365807	2.5919054449

IIId(NO_2)

C	4.3701846498	0.8897766748	-2.9138025668
C	3.5477213035	1.5905984459	-1.8144567328
O	2.389136221	0.7547847616	-1.6815375887
C	3.0760465234	-0.4732478877	-1.4576424398
C	3.9458631858	-0.5836603126	-2.7160562551
C	4.1673591926	1.3987841797	-0.416140887
C	3.9385480556	-0.134170001	-0.1796909245
C	5.2454299282	-0.9314152376	-0.1861732999
O	5.1482065529	-2.0680277136	0.494220815
C	6.3246949243	-2.8936298956	0.4773940891
C	5.5529362309	1.9487483776	-0.1499907538
O	5.9718004573	2.7493897665	-1.1293427311
C	2.1232876352	-1.6126929196	-1.220189055
N	1.3645987009	-1.3832663455	-0.0101809241
C	1.9339703632	-0.8797474446	1.0847143471
N	1.2335598683	-0.8723432964	2.217105128
C	1.6559159008	-0.249526238	3.4681760405
C	2.8171284049	0.6943581514	3.2120422208
C	3.8277544132	-0.0088949522	2.3205465777
N	3.1900341458	-0.3886875673	1.0496340581
O	6.2275327114	-0.5859652741	-0.8002869412
O	6.1719190908	1.7657795951	0.8737724985
H	0.3587754275	-1.6232493305	0.0167480176
H	0.3381237137	-1.3907618574	2.2092037845
H	1.9547382854	-1.0314924525	4.1747808271
H	0.7977533129	0.2787728137	3.890291882
H	4.6702610543	0.6432445431	2.0838128042
H	4.2173799278	-0.9097825735	2.8079656787
H	2.6934356154	-2.5489904596	-1.1669711073
H	1.4255677294	-1.6812373438	-2.0568740826
H	3.283192552	0.9728758305	4.1592669741
H	2.4687781742	1.6070683257	2.7178762159
H	3.2482093612	2.6157600496	-2.0263341593
H	6.0793268226	-3.7674581657	1.0773569884

H	7.1674668441	-2.3535348493	0.9122284367
H	6.5644793406	-3.1856229062	-0.5466462319
H	4.7793343514	-1.2798301696	-2.630252273
H	4.0828297105	1.2625870897	-3.8982134735
H	3.5167688966	1.9187368317	0.2962429054
C	7.2523105086	3.3604058643	-0.9168228155
H	7.4395860622	3.9699147442	-1.7988934418
H	7.2316279981	3.9820174568	-0.0197470332
H	8.021550861	2.5930126733	-0.8121073733
O	-1.1866872838	-2.2496330774	2.2055909219
C	-1.7901815623	-2.3115114204	1.122778113
O	-1.3551461531	-1.9975251754	-0.0235466827
C	-3.2301542774	-2.8865386384	1.2130928468
N	-3.8343260317	-2.9254040473	-0.1354117919
C	-3.4655620759	-4.2093079667	-0.8003519404
C	-2.6985914293	-4.9953826266	0.265594818
C	-3.1613013228	-4.3676023806	1.5826796091
C	-4.5107739692	-1.985081577	-0.7073388738
H	-2.8799209384	-3.9902213662	-1.6943470289
H	-4.3909610377	-4.7146043967	-1.0857817682
H	-1.6223146184	-4.8539724436	0.1377004147
H	-2.9167296131	-6.0615037452	0.1993715198
H	-4.1551790645	-4.7274272768	1.8630220467
H	-2.4726588659	-4.5284620572	2.4117876929
H	-3.8249799196	-2.2856699336	1.9013056733
C	-4.847382273	-2.1287661524	-2.1639536871
C	-6.0178398202	-1.2841260055	-2.6721275169
H	-4.9943240348	-3.1785107468	-2.4222228662
C	-6.0065027642	0.1167714745	-2.0746969686
H	-5.9655935806	-1.2563471488	-3.7635434662
H	-6.9600618226	-1.7763489264	-2.4061059117
C	-6.0684682269	-0.0098836141	-0.55734144
H	-5.0985068819	0.6527882618	-2.3765291645
H	-6.864955611	0.6884053478	-2.438618636
C	-4.8299092332	-0.7019147459	0.0319430494
H	-6.1901970249	0.963241477	-0.075071823
H	-6.9514614086	-0.6020929333	-0.2887526378
H	-5.0517898088	-0.9526391628	1.0714728204
C	-3.5154064496	0.1652417209	0.0293878427
C	-3.7931556981	1.6399713248	0.2827512609
H	-2.8904243179	-0.1893795557	0.8498013267
O	-2.8218277031	0.0149236895	-1.1870963782
C	-4.3096655724	2.1708027028	1.4758612223
C	-4.5889071034	3.5240328487	1.6415345259
C	-4.3416553125	4.403627493	0.5970395051
C	-3.8277787645	3.9118720487	-0.5986080664
C	-3.5640694353	2.5526976856	-0.7486408589
N	-4.5886502435	1.3221012906	2.6431529175
H	-4.9865746339	3.8709375899	2.587119405
H	-4.5472240891	5.4608535805	0.7214104499
H	-3.6298140733	4.5872680475	-1.4245914086
H	-3.1619027075	2.1751870272	-1.6805273921
H	-2.1607524702	-0.6839946378	-1.0112719222
H	-3.9206618544	-1.8064554291	-2.6637569803

H	5.4409017369	1.0387190328	-2.7789480768
H	3.2955963251	-0.9228246021	-3.5264446625
O	-5.4062189559	1.7181544143	3.4530838743
O	-3.9766330333	0.2689717507	2.762376895

TS1a(NO_2)

C	-3.9096087504	3.3075313254	-0.4593056311
C	-3.7569868685	2.1425901242	-1.4564979621
O	-2.519910698	1.5289816335	-1.0652373576
C	-2.8461119421	1.3236745347	0.3062068769
C	-3.1654846199	2.748750667	0.7748366022
C	-4.7295556599	0.9868893184	-1.1487212571
C	-4.1452043839	0.43403475	0.1969006838
C	-5.0745643143	0.677415988	1.3890075277
O	-4.9118096047	-0.2222269779	2.3523312707
C	-5.7190702796	-0.0167982164	3.5240209677
C	-6.2194862374	1.2510437084	-1.2050715885
O	-6.4994742376	2.4219663976	-1.7749257284
C	-1.7367844847	0.6320508264	1.0493898697
N	-1.5326609319	-0.6929906958	0.5025626816
C	-2.5526908688	-1.471058339	0.1371583361
N	-2.3022716352	-2.739656583	-0.183067193
C	-3.2747890439	-3.6840372607	-0.7296540015
C	-4.5002412176	-2.9341828954	-1.2198341697
C	-4.9115489631	-1.927442192	-0.1581403779
N	-3.8070861847	-0.9856803017	0.0894154343
O	-5.8235650056	1.6233778373	1.4568423901
O	-7.063443549	0.4536295866	-0.8637211037
H	-0.586550694	-1.0711552969	0.4532606997
H	-1.3459449954	-3.074966464	-0.0280241717
H	-3.5549047284	-4.3997451008	0.0502875849
H	-2.7913645638	-4.2333841044	-1.5405038835
H	-5.7764207727	-1.3429553455	-0.4761983425
H	-5.1656312291	-2.4313166051	0.7808061899
H	-1.988225446	0.5927368378	2.1167764299
H	-0.8111218072	1.198261233	0.931402347
H	-5.3142539397	-3.6382925174	-1.4024266403
H	-4.2813618174	-2.4096504839	-2.1553938397
H	-3.7218656108	2.4191854739	-2.5088830979
H	-5.4753693383	-0.8366053334	4.1966164217
H	-6.7770246022	-0.0394433496	3.2577669475
H	-5.475941248	0.9425015625	3.9843447348
H	-3.7354080368	2.8017712187	1.7012194006
H	-3.426020092	4.2049766657	-0.8481732396
H	-4.5550745064	0.2092701303	-1.9014293658
C	-7.896395082	2.7148047998	-1.9231882438
H	-7.9447004024	3.6958602496	-2.3919325028
H	-8.3755718189	1.96500817	-2.5553360588
H	-8.3815745919	2.7333614573	-0.9455169744
O	0.3752639511	-3.7195577595	0.1751187869
C	1.3339191098	-2.9763422006	0.3360718903
O	1.2004248397	-1.6817314086	0.424442285
C	2.7267125976	-3.601905637	0.4673853527
N	3.8178465809	-2.6358235931	0.3361910553

C	4.571645772	-2.4905812736	1.5968193262
C	3.6172055342	-3.0640052261	2.6365271067
C	2.9284346725	-4.1953656182	1.8750798254
C	4.1728016745	-2.042387451	-0.8193623012
H	5.5008980118	-3.0704565242	1.5296441015
H	4.8143018921	-1.4461320542	1.7838926468
H	4.1389656369	-3.4070117744	3.5309204313
H	2.8894636102	-2.2992497527	2.9280737436
H	3.5932362053	-5.0597706261	1.7985079611
H	1.984671748	-4.5223029605	2.3140854815
H	2.7823219538	-4.3837314195	-0.2924709855
C	5.1280291454	-1.0365176882	-0.8473496502
C	5.6768442643	-0.5027955667	-2.1488315141
H	5.7738167001	-0.9177052481	0.0168172279
C	4.6922637744	-0.625414947	-3.3110068677
H	6.0051193366	0.5349259036	-2.0191685052
H	6.5905895036	-1.0607920829	-2.3973465907
C	4.1177519655	-2.0372393558	-3.3498619706
H	3.869791258	0.0934448852	-3.2001279848
H	5.1975682632	-0.3865581685	-4.2512528934
C	3.3663507176	-2.3629033353	-2.0577579765
H	3.4370785407	-2.1613808151	-4.1965977487
H	4.9344466857	-2.7573117889	-3.4813806194
H	3.0761477513	-3.4163365446	-2.0548089353
H	2.4323895791	-1.7822384744	-2.0124406941
C	3.5217806131	0.3983132212	-0.0861601785
C	4.4890804288	1.5067343747	0.1596164108
H	3.0172371191	0.3636747176	-1.0524344855
O	2.9686810061	-0.1306778058	0.9232796218
C	4.8812560926	2.453496546	-0.7944005784
C	5.8432912574	3.4246229701	-0.542968338
C	6.4155987667	3.4909862736	0.7204180135
C	6.0294434875	2.5794165316	1.7033643972
C	5.0864958602	1.5997007347	1.4214083858
N	4.266258791	2.48519422	-2.1279888156
H	6.1138989696	4.1247226649	-1.3249013359
H	7.1553463025	4.2537488676	0.9361823016
H	6.4708619305	2.6304698545	2.6930161264
H	4.7951960995	0.8783576124	2.1779307554
H	2.0714069876	-1.0274336061	0.6478789817
H	-2.2096374439	3.25649607	0.9267235834
H	-4.9550654651	3.5326354886	-0.2515323907
O	4.9873984143	2.7315161111	-3.0774448751
O	3.0641843109	2.285864226	-2.2100352301

TS1b(NO_2)

C	-6.4724561398	-1.6748226404	-1.2815985011
C	-5.8601083135	-1.8210100414	0.1243854469
O	-4.5674020516	-2.389897577	-0.1313472396
C	-4.1190480317	-1.4088610048	-1.0614030716
C	-5.2054148771	-1.4732688391	-2.1428689934
C	-5.433594388	-0.4615355557	0.7126142156
C	-4.2192830098	-0.0867165962	-0.2044940333
C	-4.500379282	1.1247388678	-1.0978623645

O	-3.3952162376	1.794327189	-1.4087815395
C	-3.5858042533	2.9087119809	-2.2972638767
C	-6.4881669528	0.6013553768	0.9394752953
O	-7.7222630592	0.1115778655	0.8368396548
C	-2.7241188155	-1.6865816337	-1.5466866094
N	-1.7849943128	-1.5754376356	-0.4485944417
C	-1.8941949382	-0.6202375941	0.477050623
N	-0.8669939237	-0.4267815279	1.3083544255
C	-0.8558529433	0.4840877121	2.4533257008
C	-2.280971483	0.8635350728	2.8097146475
C	-3.0129601036	1.2524579191	1.5371201496
N	-3.0060279962	0.1266060972	0.5864491846
O	-5.6015283186	1.3931410745	-1.5160568133
O	-6.2437061227	1.7393630476	1.2710649888
H	-0.8749543113	-2.034275966	-0.5422563554
H	-0.0367549168	-0.9978240185	1.1577655573
H	-0.2743055208	1.3766157007	2.1963950059
H	-0.3557730094	-0.0248773957	3.2797830485
H	-4.0551671541	1.5058331786	1.7371850055
H	-2.538088467	2.1189506838	1.063103176
H	-2.4797704956	-0.988803405	-2.3575427151
H	-2.6765747394	-2.7038221882	-1.9392708538
H	-2.2762432734	1.7048212895	3.5052311005
H	-2.7936773839	0.0224772655	3.2868434696
H	-6.4139056717	-2.4470919201	0.8218745953
H	-2.5994110295	3.346928918	-2.435307557
H	-4.2688428004	3.63196834	-1.8488673694
H	-3.9892731056	2.5632404984	-3.2507332384
H	-5.2435309145	-0.6028281715	-2.7957998383
H	-7.0074239763	-2.5844067336	-1.5588814199
H	-5.0179517433	-0.6606330884	1.7071964035
C	-8.7784892414	1.0441047366	1.1097467212
H	-9.7049572715	0.4896732063	0.9730304137
H	-8.7016220308	1.4135410907	2.1339744115
H	-8.7279228831	1.8827664878	0.4130411929
O	0.8694696033	-2.5920257375	-0.9099557438
C	1.858073641	-2.3060603987	-0.2512361722
O	1.7599044907	-1.6607592095	0.8836638642
C	3.2196883241	-2.8202044966	-0.7275701346
N	4.3930337192	-2.3958670021	0.0421610627
C	4.8022741286	-3.4401760727	1.0059219124
C	3.6759383921	-4.468250863	0.9284943993
C	3.2258035005	-4.3480937253	-0.5271125078
C	5.1926606685	-1.3704769407	-0.3194960974
H	4.9343926793	-3.0012046636	1.9975877071
H	5.7454567652	-3.8962436076	0.6904898203
H	2.8576783519	-4.1976014186	1.6034063344
H	4.0234368872	-5.4681204245	1.1912263405
H	3.9702101011	-4.7884907772	-1.1963587435
H	2.2517277174	-4.7905134398	-0.7361115421
H	3.3264128853	-2.5308131577	-1.7754638078
C	6.6117798196	-1.362070848	0.1936513982
C	7.5438540907	-0.5465215704	-0.7074671368
H	6.9860027916	-2.3842000885	0.2751451897

C	6.9785330363	0.840104022	-0.9958116982
H	8.5258900562	-0.4746864821	-0.2316659624
H	7.6815016351	-1.0849121633	-1.6531166863
C	5.6146904983	0.7222250649	-1.6786870141
H	6.8975736438	1.3944024229	-0.0526561085
H	7.6610556097	1.4084898905	-1.6344226442
C	4.6980498214	-0.2682167138	-0.9989556337
H	5.1260528015	1.7008837741	-1.7459357715
H	5.764303009	0.4033677289	-2.7195384558
H	3.6813426803	-0.3225893128	-1.384227099
C	4.134142346	0.6031315726	1.0119147544
C	3.3046011073	1.706437066	0.4238581408
H	5.1738096049	0.8536068884	1.2257223548
O	3.6105717347	-0.3215416825	1.6940234231
C	1.9441571954	1.5028158784	0.1672937968
C	1.1586543354	2.4871005286	-0.4212695362
C	1.7155439921	3.7111985683	-0.7872866619
C	3.062558389	3.9487135351	-0.5481864482
C	3.8204134703	2.9600154747	0.0669571869
H	1.5005985579	0.5484858284	0.4240424744
H	0.1061020524	2.2961479672	-0.605420552
H	1.1067172724	4.4791155232	-1.2511714695
H	3.526254479	4.8934301901	-0.8081369753
N	5.2150611686	3.3234193034	0.3579993308
H	2.6248783672	-1.1211424807	1.2325081291
H	6.6055544053	-0.9521843977	1.2148196887
H	-7.1585538288	-0.8306554704	-1.3413233666
H	-4.9950591049	-2.3550961376	-2.7533086982
O	5.8303012387	3.9276018835	-0.5016396912
O	5.6687323615	3.0267631052	1.4515994476

TS1c(NO_2)

C	-3.9671565992	3.1681201784	-0.9110648426
C	-4.0045332435	1.8749069258	-1.7481963485
O	-2.7255392201	1.2758994784	-1.4944140002
C	-2.8077184991	1.2694758496	-0.0721187687
C	-3.0226964154	2.7554023326	0.2407759913
C	-4.9203248089	0.8060496508	-1.1201586375
C	-4.1179184721	0.4211756303	0.170398551
C	-4.8251760623	0.8608098668	1.4552904625
O	-4.5210700722	0.089971412	2.4928830799
C	-5.112301642	0.485151457	3.7423007669
C	-6.3948981083	1.1103605695	-0.9610196497
O	-6.757494582	2.197137914	-1.6406412728
C	-1.591829363	0.645173283	0.5552279474
N	-1.5082246451	-0.7496916208	0.1776222864
C	-2.5860907911	-1.5323058721	0.115544272
N	-2.4098914422	-2.8426628544	-0.0491563496
C	-3.4743351279	-3.8206843251	-0.2630907186
C	-4.7573106193	-3.1064181747	-0.6481021689
C	-4.96675933	-1.9363807277	0.2987133262
N	-3.8237593989	-1.0118230979	0.2097732942
O	-5.5325504706	1.8389157328	1.5151583233
O	-7.1751249843	0.398922922	-0.3696048735

H	-0.5853976397	-1.1717673736	0.0606835897
H	-1.4421210221	-3.1790000117	-0.02496316
H	-3.6220124674	-4.3963378136	0.6566395374
H	-3.1478861226	-4.5069351898	-1.0474677184
H	-5.8656902903	-1.3726468274	0.0435918321
H	-5.0629558819	-2.2831880598	1.3334030282
H	-1.6457310308	0.766212248	1.644477201
H	-0.6943783801	1.1514252434	0.1936349171
H	-5.5992009956	-3.7979270682	-0.5782846322
H	-4.6977260724	-2.7379364766	-1.6771561815
H	-4.1513792869	2.0057001647	-2.818986678
H	-4.770688017	-0.2448942057	4.4731151679
H	-6.2004746454	0.4693000559	3.6624719274
H	-4.7783097575	1.4876779929	4.0156143257
H	-3.4187797236	2.9541791467	1.2353223242
H	-3.5514725989	3.9884246331	-1.498252368
H	-4.8851560229	-0.0717496579	-1.7754340923
C	-8.1573617352	2.5099063176	-1.5986340154
H	-8.2761479074	3.4148169931	-2.1915598226
H	-8.7409028069	1.6929600303	-2.0270656887
H	-8.4725007618	2.6836363223	-0.5681397189
O	0.3196420586	-3.7455390232	-0.0333618998
C	1.3041640823	-3.0159940696	-0.0617045872
O	1.1887241533	-1.7209792451	-0.0497265656
C	2.690213282	-3.6830768862	-0.138240602
N	3.7996981189	-2.8013060025	0.2246073672
C	4.2685770202	-3.0761688796	1.5997141647
C	3.2557306996	-4.0813832039	2.1419163455
C	2.7988954865	-4.8195211436	0.8847206474
C	4.4850145483	-1.9989770868	-0.6180272785
H	5.278628284	-3.4999262837	1.5452003446
H	4.3191112514	-2.163969247	2.1999292483
H	3.6976172341	-4.7369899426	2.8932880784
H	2.409844704	-3.555084715	2.5973076796
H	3.5685276595	-5.52220354	0.5532893539
H	1.8535133329	-5.3487456375	0.9950168779
H	2.7870957363	-4.0561840176	-1.1604085577
C	5.4281372289	-1.0981814452	-0.140001407
C	6.3251337075	-0.3144029195	-1.068073086
H	5.795197599	-1.2436736128	0.8727031681
C	5.6606029512	-0.0494045826	-2.4159109703
H	6.6159197388	0.628282598	-0.5891355864
H	7.2601098685	-0.8708621336	-1.224650331
C	5.1223615819	-1.3554029847	-2.9859218092
H	4.8304833358	0.656414847	-2.2869404227
H	6.3756004149	0.4094696623	-3.1050746088
C	4.065810862	-1.9645993156	-2.0627700092
H	4.6789728723	-1.201941805	-3.9737363953
H	5.948642366	-2.0658674549	-3.1091183508
H	3.8206464475	-2.9733497886	-2.4029838009
H	3.1373547633	-1.3748019592	-2.1163042393
C	3.6374026144	0.1992744638	0.4783696094
C	4.3814704329	1.4864737827	0.5534566733
H	3.6049935732	-0.4322187953	1.3629172265

O	2.6859986501	0.1144339712	-0.3578256427
C	4.0771599912	2.4624821272	-0.4007099734
C	4.7438438875	3.6830908486	-0.4339343276
C	5.7495690802	3.9585156357	0.4877139584
C	6.087247282	3.0052609269	1.4419282111
C	5.4011603488	1.7990007755	1.4686698185
H	3.296177566	2.2417448704	-1.1193558096
H	4.4750706641	4.4198531624	-1.1833670669
H	6.2720940978	4.9082984003	0.4692156153
H	6.8696446728	3.1889094991	2.168209209
N	5.8233460153	0.8494281149	2.5044540984
H	1.9949784633	-0.9415787471	-0.1898629108
H	-2.0492484624	3.2442321145	0.1513030035
H	-4.9574516011	3.4538026681	-0.5580346302
O	6.9851346377	0.8837780607	2.8637552167
O	4.9887337602	0.0832039446	2.965474508

TS1d(NO_2)

C	2.797943322	2.3847808904	-1.7938475535
C	2.1923264256	1.9633376663	-0.4438831766
O	1.2094172188	0.9763086974	-0.8113861172
C	2.0845391579	0.1255139018	-1.5565861563
C	2.6410664044	1.0855958238	-2.6141678585
C	3.1577487707	1.0786048971	0.3684794363
C	3.1487893555	-0.2514976276	-0.4601506158
C	4.5121363031	-0.5695003969	-1.0800057252
O	4.7654147616	-1.871289783	-1.1244531
C	6.0198885634	-2.2296939885	-1.7279106409
C	4.5069794889	1.6315442545	0.7714973168
O	4.5756978368	2.9525289189	0.6190465962
C	1.3742453109	-1.0789152287	-2.1100475998
N	0.8671534798	-1.8925861569	-1.0267757753
C	1.5314052633	-2.0412341062	0.121633763
N	1.0335013656	-2.8660465347	1.0419204466
C	1.4686063787	-2.9417668213	2.4375103898
C	2.4534737093	-1.824838597	2.7432096295
C	3.4217081872	-1.6994774329	1.5774785881
N	2.6686297473	-1.365308145	0.3577654361
O	5.2448281582	0.2863353942	-1.5178397262
O	5.3867451083	0.9652560787	1.268561571
H	-0.0209769038	-2.3816912265	-1.1580431242
H	0.2119846295	-3.4079251405	0.7662914857
H	1.9335824276	-3.917942133	2.607082281
H	0.5801409853	-2.8692791333	3.0698311937
H	4.1532294616	-0.9065884122	1.7412860025
H	3.965909627	-2.6354930626	1.4121723807
H	2.0678378155	-1.6390822159	-2.7496775603
H	0.5283517646	-0.7529635733	-2.7193004619
H	2.9957755838	-2.05729848	3.6617975244
H	1.9235043934	-0.876717535	2.875097524
H	1.7233283796	2.7562820935	0.1386831676
H	6.0732117438	-3.3150094879	-1.6728296712
H	6.8436697384	-1.7753415038	-1.1749055765
H	6.0459621901	-1.8961756173	-2.7667731921

H	3.5599857111	0.7454128718	-3.0867951424
H	2.2263487599	3.206940537	-2.2270884736
H	2.6612717353	0.8547332185	1.3197953177
C	5.7993102192	3.5593899612	1.0608511591
H	5.6868856719	4.6248250521	0.8696754205
H	5.9490503757	3.3755237166	2.1262181341
H	6.6428888471	3.1562135395	0.4977870697
O	-1.4954519823	-4.0548710794	0.2736398676
C	-2.2444032148	-3.3209984052	-0.3634818519
O	-1.8606720546	-2.6533216307	-1.413817365
C	-3.7226383378	-3.2589216238	0.0281450845
N	-4.3809105407	-1.9759275821	-0.2394443541
C	-5.3561389294	-2.0792603561	-1.342130548
C	-4.9726742845	-3.3890193219	-2.0222080744
C	-4.5158752715	-4.2516334218	-0.8452733075
C	-4.2892716583	-0.9206339618	0.5933163732
H	-5.2819209004	-1.2087616754	-1.9968872293
H	-6.3752214315	-2.1381537426	-0.9420154422
H	-4.1451737345	-3.2270205307	-2.7185135171
H	-5.8108418645	-3.8242990069	-2.5682644109
H	-5.3796375886	-4.6079473003	-0.2770903132
H	-3.9133375548	-5.1143272081	-1.1365255756
H	-3.7832438513	-3.4987212554	1.0921941376
C	-5.2918462959	0.1903000372	0.4226810255
C	-5.5479491444	0.9327029434	1.7363499725
H	-6.2300550572	-0.2074077675	0.030255026
C	-4.2432579977	1.3994640587	2.3716242418
H	-6.2111128717	1.7812291294	1.5448270691
H	-6.0734953649	0.2608470613	2.4259160939
C	-3.3082550852	0.2167288692	2.6261768218
H	-3.7626504556	2.1275559472	1.7094525482
H	-4.4427227624	1.9139479798	3.3166409166
C	-3.2393898541	-0.7751951436	1.4876680723
H	-2.3053498104	0.5746282321	2.8785600984
H	-3.6516209949	-0.3265406022	3.5183982855
H	-2.5776196366	-1.623833199	1.6513507592
C	-1.9074133023	-0.0320630988	-0.354147634
C	-1.8430793181	1.395003495	0.0081064478
H	-1.1812194909	-0.7115624259	0.0799270923
O	-2.4838067934	-0.3667190434	-1.4379302671
C	-1.0251104641	1.948463991	1.011502999
C	-1.030397702	3.3099748966	1.2970509422
C	-1.8502679599	4.1611685827	0.5664636199
C	-2.6280079401	3.6501926784	-0.4707255114
C	-2.6144863718	2.2890168422	-0.746360079
N	-0.0733596071	1.1362122338	1.7792762615
H	-0.3894911254	3.6911214671	2.0821460578
H	-1.8617606029	5.2207119799	0.7958692169
H	-3.2452696694	4.3130657208	-1.0676212048
H	-3.2025311845	1.8883621367	-1.5640804191
H	-2.296835275	-1.5453781139	-1.5290131963
H	-4.901296515	0.8829954392	-0.3386179945
H	3.8380472471	2.6933074258	-1.6931072371
H	1.8717586657	1.192390208	-3.3830450343

O	0.8101558837	1.721629841	2.386105301
O	-0.1942554181	-0.0791608729	1.7901906481

TBDH⁺

C	0.096621781	-0.1673538285	-0.6511320971
N	-0.1562367701	0.27061005	0.5870851242
N	1.231004824	0.1674628928	-1.2771729263
N	-0.7605296688	-0.9823497856	-1.2771730119
H	-0.5278866686	-1.2583615848	-2.2223158042
C	-2.0576968736	-1.3800881141	-0.7357869328
H	1.3537165387	-0.1720177474	-2.2223157233
C	2.2240400949	1.0919732384	-0.7357867486
H	3.2021084468	0.7814097503	-1.1058134097
H	2.0230805243	2.106813402	-1.0961045644
C	2.1660843348	1.0417579531	0.7806938922
H	2.7958727287	1.8273067748	1.2022006171
C	0.7307200361	1.2415749356	1.2373084739
H	2.5346248851	0.0748226437	1.1357199211
H	0.6399486509	1.0816663758	2.3135393338
H	0.3860637121	2.2580832187	1.0122409102
H	-2.2777753799	-2.3824018345	-1.1058136453
C	-1.9852313731	-1.3550046689	0.7806937136
H	-2.8360942749	-0.6986317513	-1.0961047734
H	-2.9804308668	-1.5076427991	1.2022003687
H	-1.3321113089	-2.1576379618	1.1357197548
C	-1.4405955354	-0.0120343701	1.2373083805
H	-1.2567250375	-0.0133784058	2.3135392522
H	-2.1485891748	0.7947010704	1.0122408012

Ia(TBD)

C	-3.4565256008	-0.0727100708	0.1434236178
N	-4.7508179053	0.2652810958	0.2217242403
N	-3.0249886297	-0.9304323727	-0.7858570499
N	-2.5574715651	0.4718445263	0.9737886295
H	-1.593696133	0.1677878847	0.8872806814
C	-2.8940493369	1.3792727887	2.0670063516
H	-2.0294263312	-1.1524142178	-0.80182004
C	-3.9046236997	-1.6317184312	-1.7163789205
H	-3.3465380489	-1.7935252205	-2.6401972031
H	-4.179319512	-2.6095839254	-1.3050294774
C	-5.144292567	-0.789765028	-1.9593601806
H	-5.874907837	-1.3582039889	-2.5379777993
C	-5.7515956287	-0.3934950996	-0.6239733188
H	-4.879238195	0.108892507	-2.5243596543
H	-6.567345756	0.3181289963	-0.7669233313
H	-6.1520914769	-1.2708585145	-0.1014857871
H	-2.0585422417	2.0700149211	2.1929722531
C	-4.1741020307	2.118833803	1.7213118409
H	-3.0171541056	0.8132044115	2.9971463643
H	-4.5202868741	2.6879381309	2.5861087666
H	-3.9913315078	2.8164391915	0.8985474159
C	-5.2394446903	1.1153347191	1.3121319613
H	-6.1308853818	1.6271004177	0.9439480016
H	-5.5329716094	0.4885413692	2.1631538041

O	0.3859033426	-0.2611751485	0.7241615417
C	0.6814143926	-1.0932905736	-0.2699443526
O	-0.1783718931	-1.5804117207	-0.9728518832
C	2.1698881919	-1.4022974684	-0.4142375808
C	2.4528950363	-2.8469632141	0.0425252662
C	2.8021831852	-2.671684536	1.520564382
C	3.6355939194	-1.3971692526	1.4950139779
N	2.9379555549	-0.54726945	0.5135826656
H	1.606198446	-3.5087231563	-0.147713138
H	3.3193683975	-3.2241579088	-0.5066606606
H	1.8958765389	-2.5259275291	2.11879729
H	3.3539701724	-3.5181389931	1.9318898168
H	3.6960445664	-0.8872956241	2.4596601444
H	4.6571032207	-1.6121592356	1.150753822
C	3.6201849323	0.5878188245	-0.0001257208
H	2.4351038092	-1.2526402464	-1.4630481658
C	4.776035853	1.0275026598	0.5204677122
C	5.4874624741	2.2661473058	0.0382189254
H	5.2357760127	0.4955470373	1.3478997683
C	4.9405621541	2.7694273348	-1.296554768
H	5.3976750512	3.0580110709	0.7958404001
H	6.5600723462	2.0568427836	-0.0470007626
C	3.4147419911	2.7530045272	-1.2746930975
H	5.2928816085	2.1178319779	-2.1063012335
H	5.3166525142	3.7758990069	-1.5035370882
C	2.9045146089	1.3211703887	-1.1151587069
H	3.0029017607	3.1884690897	-2.1898233856
H	3.0585905603	3.3621260924	-0.4344478479
H	3.0377595072	0.7786613238	-2.0602181317
H	1.8263863277	1.3290947357	-0.913294569
H	1.2707740816	-0.014975995	1.1092472139

Ib(TBD)

C	3.3963954464	-0.0485697339	-0.1771295231
N	4.6893824807	0.2964586317	-0.2464388986
N	2.96730215	-0.9280285901	0.7328921937
N	2.4960585066	0.5106344485	-0.9960765645
H	1.5309756241	0.2107580245	-0.9090772788
C	2.8348158347	1.4359009581	-2.0730950577
H	1.9765379483	-1.1715306064	0.7269562708
C	3.8452797952	-1.6411016197	1.6561424966
H	3.2877506042	-1.8100663576	2.5790884897
H	4.1156530065	-2.6158690373	1.2347966656
C	5.0892133307	-0.8077530482	1.9069369791
H	5.8198398243	-1.3891604926	2.4724810767
C	5.6906652466	-0.391258695	0.574974105
H	4.8315210573	0.0827528347	2.4880240392
H	6.5154804001	0.3082262495	0.7247528564
H	6.0766717769	-1.2621648017	0.0311370625
H	1.9927924504	2.118369318	-2.1998001232
C	4.1018788288	2.1848160301	-1.7007489526
H	2.9761486368	0.8847147896	-3.0096580873
H	4.4483831851	2.7770617285	-2.5497563321
H	3.9019989543	2.8618650801	-0.8649459649

C	5.1773583402	1.1870743737	-1.304486931
H	6.0548639618	1.7029001107	-0.9086432921
H	5.4938438838	0.5900890261	-2.1686006436
O	-0.4353084911	-0.264136965	-0.7679015984
C	-0.7212293045	-1.1762082066	0.1578618449
O	0.1458657251	-1.6812925311	0.8386298526
C	-2.1952989229	-1.5533078777	0.2426270226
C	-2.4639319066	-2.9078655225	-0.4553077232
C	-3.0650035624	-2.505921787	-1.803258373
C	-3.8836905635	-1.2756227943	-1.4390378378
N	-2.9911451188	-0.5710637369	-0.496062348
H	-1.562579096	-3.5186647497	-0.5330716443
H	-3.1994193936	-3.4599112335	0.1344594823
H	-2.2772299707	-2.2311340354	-2.5125930823
H	-3.6727987259	-3.2957406686	-2.2472103367
H	-4.1132040192	-0.6432854805	-2.2975388436
H	-4.8243311283	-1.5642230101	-0.9495410077
C	-3.4560348422	0.5769660993	0.1939673328
H	-2.459525438	-1.6028441471	1.3039775012
C	-2.9760905323	0.9550710201	1.3906934585
C	-3.4464301489	2.187672922	2.1207015997
H	-2.2078227613	0.3696207323	1.8886124998
C	-4.7450273274	2.7405375207	1.5373689783
H	-3.5793991387	1.9477079905	3.1818848446
H	-2.6669919354	2.9624709405	2.0842445239
C	-4.6403465129	2.8016553084	0.0166833619
H	-5.5797002984	2.0841689215	1.8148815042
H	-4.956632067	3.730070854	1.9537168636
C	-4.4689102496	1.4004958655	-0.5698904288
H	-5.5233690308	3.2753339572	-0.4222223914
H	-3.7735971551	3.4167686139	-0.2564087294
H	-5.4319544675	0.8725726239	-0.5771120967
H	-4.1497640243	1.4717265466	-1.6157063222
H	-1.3095428658	-0.0008957918	-1.1518824935

IIa(TBD)

C	4.2087482732	-0.4860542434	-1.3880058414
C	3.6748804009	-0.4109239909	-0.096720068
C	4.4708629318	-0.6999807826	1.0137613886
C	5.8029962708	-1.061653683	0.8452007248
C	6.3141156427	-1.1252915098	-0.4471889811
C	5.5361026535	-0.8424174924	-1.5703053271
C	2.2662786504	-0.0350809786	0.1082596943
O	1.4880719744	0.1643584236	-0.8152097783
H	4.0445018839	-0.6440023195	2.0123078159
C	3.2017168329	2.8148082902	0.8832563819
C	1.8566773109	2.8983176045	0.7991813842
C	0.9711782023	2.7742062203	2.0207774023
C	1.754153613	2.9263508871	3.3248645883
C	3.0300070296	2.0907710013	3.2892676625
C	3.946837163	2.5880036161	2.1732578343
N	1.1968924996	2.9785475967	-0.4251988024
C	-0.1486143356	3.513812038	-0.571069096
C	-0.2231239562	3.9469545147	-2.0550433561

C	0.8507407796	3.0922512994	-2.7295672619
C	1.9494638671	3.0644133319	-1.6750339191
C	-1.2305231999	2.4781148157	-0.2814104518
O	-0.9018902332	1.1950621348	-0.3283841138
O	-2.3798383342	2.8077506701	-0.0581138617
N	-4.7181776821	1.1181578754	0.3280135048
C	-4.5085290743	-0.1972858063	0.2213374246
N	-5.5173948255	-1.0700528936	0.3507842156
C	-6.9020469568	-0.6008861077	0.4613696954
C	-6.9553467425	0.7331719166	1.1873691705
C	-6.0370487333	1.7217211529	0.4907733065
C	-5.3052480247	-2.5047162819	0.1340384481
C	-3.8974879581	-2.9044089467	0.5441828951
C	-2.8952976198	-2.0250943961	-0.1821877889
N	-3.2559993501	-0.6255151924	0.0207170519
H	-3.7272884685	-3.9526019012	0.2911248126
H	-6.0478718689	-3.0316677666	0.7370681761
H	-5.4881518915	-2.7471553617	-0.9200170996
H	-2.5279962204	0.0743113947	-0.0870061515
H	-3.9110944341	1.7287826774	0.197919289
H	-6.4440807365	1.998598389	-0.4883330777
H	-5.9148214071	2.6319742621	1.0802367448
H	-7.4540505219	-1.3642872395	1.0133582254
H	-7.3426444033	-0.5186315935	-0.5398455617
H	-2.8858176767	-2.2583764436	-1.2529273676
H	-1.8855652918	-2.1660024471	0.2077264954
H	-7.9798858073	1.1099730771	1.1847901156
H	-6.639308792	0.5988305228	2.2261870236
H	2.5605126594	3.9798154828	-1.7064471671
H	2.6172143343	2.2029183766	-1.7684623941
H	1.1882161723	3.5094317695	-3.6796978665
H	0.4818032206	2.0759940988	-2.9064396661
H	0.0453265464	5.0047369765	-2.1171792022
H	-1.2241119871	3.8228770436	-2.4728831035
H	-0.3570754569	4.3634822912	0.0857180924
H	3.8019056823	2.9214810052	-0.0156798554
H	4.7584128452	1.8690421477	2.0016843206
H	4.4383298835	3.5191162969	2.4913343994
H	2.7643101407	1.040229335	3.1069364814
H	3.547906374	2.1299953427	4.2525164649
H	1.1169594088	2.6387832633	4.1663818417
H	2.0198859029	3.9813983267	3.4659624085
H	0.170891686	3.5214584578	1.9889948609
H	0.4742634179	1.7926845914	1.9931137179
H	1.9292263638	0.0234592075	1.1573287989
H	6.4332680527	-1.2903262134	1.6970876792
Cl	7.9852100922	-1.5761479855	-0.6740121428
H	5.9682387187	-0.903492683	-2.5627979989
H	3.5753904885	-0.2638819773	-2.2412493203
H	0.0633401093	1.0132786794	-0.5065334358
H	-3.7815390887	-2.7861021683	1.6256635205

IIb(TBD)

C	0.9320749211	3.5249470584	1.1674067358

C	0.8726777843	2.1293480807	1.1556301295
C	-0.0679399681	1.4745504942	0.3525655861
C	-0.9468164185	2.2085710877	-0.4310625065
C	-0.8689548348	3.6015772973	-0.401099819
C	0.0594049774	4.2745032574	0.3866584208
C	1.8320628942	1.3764508084	1.9880265156
O	1.8736021392	0.1634273258	2.0878113686
Cl	-1.9713893549	4.527511698	-1.3870043534
C	2.57105196	0.2461559591	-1.3665080695
C	3.3984485643	-0.3924345044	-0.5164053714
C	4.6334095006	0.2745028469	0.0480425363
C	5.0266319028	1.518996745	-0.7458650054
C	3.8117282107	2.4106340267	-0.9741135888
C	2.7642550992	1.6755504279	-1.8101700966
N	3.1459081178	-1.6930036648	-0.0537518481
C	2.2860985396	-2.581899687	-0.8170850597
C	2.7807575303	-4.0017689783	-0.4623246447
C	3.4294601322	-3.8030163688	0.9079228871
C	4.1211348036	-2.4556391329	0.7388700979
C	0.8204212304	-2.4421492183	-0.4252476875
O	0.5586333459	-1.7990030496	0.7096994356
O	-0.0814077808	-2.9386101213	-1.068915694
N	-2.9131719297	-2.2642183156	-0.7186250759
C	-3.2358275464	-1.4108010511	0.257196244
N	-4.466648605	-0.8902037751	0.3420176829
C	-5.5314636689	-1.3314693966	-0.5651507862
C	-4.9538257971	-1.7207009272	-1.9159466729
C	-3.8597444519	-2.7545204898	-1.7160329671
C	-4.847269466	-0.0400118413	1.475616139
C	-3.6519585268	0.7581970928	1.9713910144
C	-2.5105080012	-0.1920627813	2.2843099671
N	-2.2811728055	-1.0516648672	1.1270861917
H	-3.9312633534	1.3145894415	2.8680912878
H	-5.6374662041	0.6261663729	1.1234832348
H	-1.3807061404	-1.5133991607	1.0499115635
H	-1.9446287144	-2.5808290167	-0.7670105883
H	-4.2878408134	-3.7074828527	-1.3858831905
H	-3.3050199195	-2.9300708437	-2.6391926553
H	-6.2283749371	-0.4973352768	-0.6700484681
H	-6.0713524349	-2.1726400093	-0.1137879317
H	-5.7452780045	-2.1305357446	-2.5462618201
H	-4.5432344154	-0.8344936903	-2.409322999
H	4.3212253558	-1.9521697886	1.6878032919
H	5.0719094716	-2.5843508766	0.2026748712
H	2.6646670754	-3.7396739706	1.6895090022
H	4.1278224557	-4.5998487883	1.1684895272
H	3.5346264422	-4.2990319307	-1.1960802257
H	1.9751758398	-4.7381105626	-0.4776508472
H	2.3415562615	-2.3911698453	-1.8952279154
H	5.4660746101	-0.4355956918	0.0497396777
H	5.8157093314	2.0582609714	-0.2129785964
H	5.4407328313	1.2136432133	-1.7150685812
H	3.3835787203	2.6742358679	0.0016295687
H	4.0968494201	3.3471104934	-1.4636945209

H	1.8038148591	2.205383143	-1.7500337433
H	3.0562528934	1.7045248366	-2.8700707395
H	1.6949898966	-0.2656902698	-1.7610235096
H	2.5448689361	2.000685589	2.558242474
H	-0.1016073512	0.3907125833	0.3350862351
H	-1.6756380048	1.7173230158	-1.0665172781
H	0.0970181602	5.3578271229	0.3877298358
H	1.6686190402	4.0270368741	1.7895322483
H	1.347140053	-1.3175981175	1.055246325
H	4.4484310624	0.5343094013	1.0998070382
H	-1.5815446068	0.3488820118	2.4822790797
H	-2.7476651698	-0.8017371172	3.1634441471
H	-3.3399258309	1.4737184407	1.2035029195
H	-5.2597843135	-0.6630798607	2.2784185767

IIc(TBD)

C	4.7099089613	-0.1127646756	-0.667801578
C	3.3407469098	-0.3638633235	-0.5679453624
C	2.8617588146	-1.332261712	0.3212134834
C	3.7474413086	-2.0415387623	1.1186968599
C	5.1104757582	-1.7613858887	1.0161994972
C	5.6093215876	-0.8094199074	0.1319131353
C	2.400775432	0.431791192	-1.3779549813
H	2.8621829344	1.1417198011	-2.0863800246
H	1.7940702206	-1.5180644024	0.3822711914
C	3.2942226971	3.1611573997	0.1851850069
C	1.9702815227	2.985819743	0.3503396033
C	1.4129016732	2.2656043674	1.5577601966
C	2.3982656758	2.2561239166	2.7266868919
C	3.7803737064	1.8012956736	2.2673871992
C	4.3304584729	2.7470886769	1.1997749391
N	1.0263735115	3.3348045087	-0.6376608092
C	-0.2639823791	3.9132886132	-0.2381421204
C	-0.6266780675	4.8968252011	-1.3753748612
C	0.1917351561	4.3812075631	-2.5589299698
C	1.4883772089	3.9656909555	-1.878674268
C	-1.3407847982	2.8427497651	-0.1217753281
O	-1.0628691586	1.6593900594	-0.6541094939
O	-2.4221678701	3.0515034557	0.3909932095
N	-4.369797103	0.8894912586	0.6704371831
C	-4.0801338828	-0.377358179	0.3583773405
N	-4.9693720312	-1.3592046041	0.5638835929
C	-6.3328757342	-1.0553231838	1.009975726
C	-6.3407996128	0.1789247922	1.8966980417
C	-5.6720419979	1.3278978045	1.1633911246
C	-4.6931295997	-2.727399161	0.1136575499
C	-3.2055418804	-3.0269829798	0.2001475295
C	-2.4364433874	-1.9724243218	-0.5748150929
N	-2.8631286384	-0.6498520231	-0.1291266593
H	-3.006941946	-4.0159719284	-0.2171098335
H	-5.2589323739	-3.3989523681	0.7625894243
H	-5.058298673	-2.857103659	-0.9125732229
H	-2.2458772705	0.1359856709	-0.3087121152
H	-3.6547360109	1.6003889103	0.5090308571

H	-6.2948669433	1.6624744518	0.3260685803
H	-5.5045656817	2.1784924493	1.8263797656
H	-6.6924236601	-1.9277147988	1.5598170583
H	-6.9806916996	-0.9124162003	0.1363534214
H	-2.6150091619	-2.0805925971	-1.6506878479
H	-1.3621694975	-2.0505599806	-0.396799582
H	-7.3704161245	0.4427805505	2.1456550795
H	-5.8036704556	-0.0325552846	2.826120832
H	2.1091472063	4.8489280177	-1.6651201017
H	2.0865664214	3.2607529874	-2.4628047774
H	0.3473193566	5.1384721283	-3.3289282305
H	-0.2916123864	3.5118367749	-3.017592949
H	-0.2851653157	5.8954737412	-1.091498462
H	-1.7025026204	4.9404097929	-1.555247351
H	-0.2277075619	4.4314437546	0.7242784863
H	3.6639798754	3.6627417117	-0.7048899358
H	5.1817639474	2.2798663855	0.6891983595
H	4.7337966567	3.6504086116	1.6801348427
H	3.7015235263	0.7881876968	1.8557298404
H	4.4717579798	1.7506433257	3.1144236821
H	2.0180729998	1.6049611034	3.5195633039
H	2.4673075797	3.2683350638	3.1445827235
H	0.4709537033	2.7189747327	1.884447353
H	1.1697260456	1.2354501153	1.2518523253
O	1.1897612419	0.3202269682	-1.2934076754
H	3.3963422923	-2.7955668986	1.8142772733
Cl	6.2271424385	-2.6377622851	2.0321624091
H	6.6750016435	-0.6185643217	0.0741886996
H	5.0713957892	0.6399572181	-1.3640177299
H	-2.8905509138	-3.0237364452	1.2479106161
H	-0.1286498171	1.6218029824	-0.9711858721

IId(TBD)

C	2.540080611	0.1738679377	-1.2348989748
C	3.6226145643	-0.6910248212	-0.6112216367
H	4.1788745461	-1.2363609685	-1.3833701176
C	1.4724183379	-0.719237314	-1.8397727986
N	1.021312976	-1.6674574711	-0.8250858608
C	1.8110492119	-2.1448953015	0.1494113974
N	1.3247154522	-3.0957902115	0.9510046017
C	2.0790504504	-3.711475744	2.0392856183
C	3.1317765737	-2.7357066043	2.533740797
C	3.9328267885	-2.2192350514	1.3502685134
N	3.0400115214	-1.6466281959	0.3372080816
H	0.1285275541	-2.1309098786	-0.9642688387
H	0.3709728456	-3.4212309146	0.7889978096
H	2.5480782591	-4.637239152	1.6882266444
H	1.3702019555	-3.9652044334	2.8293858199
H	4.6136400935	-1.4245566269	1.6616967514
H	4.5286558579	-3.0229040445	0.9007043016
H	1.8681604044	-1.2573489093	-2.7085429152
H	0.6055646468	-0.1390778651	-2.1632719416
H	3.8003029286	-3.2336673409	3.2384641406
H	2.6519568469	-1.896898927	3.046821289

O	-1.4755971184	-3.8861403972	0.9356375529
C	-2.2489069921	-3.2521997934	0.2470981163
O	-1.8124767563	-2.6204809912	-0.8409098747
C	-3.7468506471	-3.211778188	0.513256052
N	-4.3757034443	-2.0219207332	-0.03322238
C	-5.3996186277	-2.366247336	-1.0291257055
C	-4.9407076305	-3.7358914818	-1.5154564264
C	-4.431010434	-4.3844337645	-0.2273215655
C	-4.4026458829	-0.840039723	0.7110451836
H	-5.4276565149	-1.61706927	-1.8239216296
H	-6.3992799936	-2.4380117822	-0.5778938583
H	-4.1278499292	-3.6274206445	-2.2414952512
H	-5.7471818698	-4.3024079197	-1.9837897897
H	-5.2722648499	-4.7291736339	0.379547727
H	-3.7562810557	-5.2280608458	-0.3881304481
H	-3.8720016783	-3.2872154608	1.5991006705
C	-5.4267729966	0.1878954436	0.2893444527
C	-5.7109818911	1.1967332857	1.401913455
H	-6.3576264878	-0.3090522658	0.0022211744
C	-4.4142409945	1.7787729718	1.9532853235
H	-6.3585122181	1.9908353201	1.0174630255
H	-6.2595735781	0.6931240859	2.2078326898
C	-3.5252277104	0.675268599	2.5321101526
H	-3.8894150329	2.2929515211	1.1405909692
H	-4.6230617596	2.528866259	2.722703993
C	-3.5314672919	-0.5920988879	1.7123597458
H	-2.4956034984	1.04479578	2.6295537117
H	-3.8508851576	0.4428307386	3.5564953724
H	-2.803772773	-1.349379228	1.9987865262
C	-2.0455840869	0.3202458797	-0.975033101
C	-2.1347399468	1.7737223436	-0.7767773054
H	-1.313536308	-0.1992511977	-0.3286193344
O	-2.7054666309	-0.300282052	-1.7958691678
C	-1.3292273233	2.3708349478	0.1954562231
C	-1.4287556265	3.7346560004	0.4445428075
C	-2.3426022966	4.4785645931	-0.2967545208
C	-3.1485840611	3.9039191051	-1.2804618202
C	-3.0379183213	2.5422634418	-1.5193566007
H	-0.6315292334	1.7634294885	0.7663157581
H	-0.815247762	4.2132684171	1.1992743943
Cl	-2.4825538794	6.1902265041	0.0110617453
H	-3.845382112	4.5160804302	-1.8418467151
H	-3.650467037	2.0629387632	-2.276584732
H	-2.4779262178	-1.9856286467	-1.2103146616
H	-5.057588711	0.7006062366	-0.6118143258
H	2.0957853385	0.819171586	-0.4707510645
H	2.9797928694	0.8069605029	-2.0077659988
H	4.331947735	-0.0779521637	-0.0512792273

IIIa(TBD)

C	3.8032046899	-0.6776114467	-2.1450616143
C	3.39467745	-0.9317145857	-0.8340945574
C	4.0965133452	-1.8633630417	-0.0698596249
C	5.1988012654	-2.5331931939	-0.5957763648

C	5.5845889211	-2.2599422834	-1.9021629184
C	4.8982121858	-1.3401297729	-2.6889444162
C	2.2202457661	-0.1807802834	-0.251635762
O	1.2316766677	-0.0643786479	-1.2456633365
H	3.7797375967	-2.0746998013	0.9482700918
C	2.6901191829	1.2418592979	0.2356982896
C	1.4412540402	1.9525679648	0.671446819
C	0.9034214041	1.6234927195	2.0331927232
C	2.004346746	1.6217827893	3.1038128062
C	3.1738149361	0.7320957841	2.696879608
C	3.7407503191	1.1992207356	1.358392368
N	0.7922148817	2.7079697293	-0.1507711378
C	-0.573447662	3.2207985641	0.1016740251
C	-0.8059677799	4.2107774923	-1.0393376121
C	-0.0544970356	3.5616065899	-2.1995288943
C	1.2204281019	3.042573667	-1.543351145
C	-1.5878199949	2.0443311275	0.0514514068
O	-1.1412594331	0.8849382711	-0.1781455132
O	-2.7705227995	2.3703370955	0.2435337726
N	-4.697085354	0.3249214621	0.2021123016
C	-4.3324851379	-0.9179124418	-0.1272157169
N	-5.2377530217	-1.9103813146	-0.1913131595
C	-6.6687375023	-1.6276255888	-0.0592233552
C	-6.8932067732	-0.4591232859	0.886645732
C	-6.0809084971	0.7326096398	0.4106665364
C	-4.8535398307	-3.2464586639	-0.6526323006
C	-3.4173679796	-3.5539717246	-0.2604637453
C	-2.5130068423	-2.4484568247	-0.7764555271
N	-3.0374916504	-1.1569681689	-0.3511679104
H	-3.1195241702	-4.5142878017	-0.6860777917
H	-5.5415523688	-3.9561821024	-0.1878103376
H	-4.9814338214	-3.3134784348	-1.7404809355
H	-2.3818472258	-0.363197405	-0.2810561574
H	-3.9653655495	1.0535851451	0.2059532543
H	-6.5009327858	1.1304052074	-0.5209745893
H	-6.0837865739	1.5342265333	1.1519995123
H	-7.1405312341	-2.5339560267	0.3270335785
H	-7.0979265777	-1.416380362	-1.0469190514
H	-2.4502904786	-2.4884733371	-1.8705188304
H	-1.5005984094	-2.5423792365	-0.3773247115
H	-7.9547351593	-0.2046095646	0.909698185
H	-6.5837705399	-0.7412983454	1.8976294068
H	1.9887326224	3.8175601498	-1.470726131
H	1.6223609819	2.155090837	-2.0257620551
H	0.1714050029	4.2531709159	-3.0117764558
H	-0.6307423542	2.7238311998	-2.6043692457
H	-0.3641846201	5.1790105283	-0.7876377113
H	-1.8729563545	4.3425256167	-1.2187315053
H	-0.6308597179	3.6933660693	1.0817530457
H	3.1257898155	1.7381256535	-0.6343815913
H	4.5699758581	0.5644762352	1.0340946324
H	4.1453473774	2.2106620157	1.4795448451
H	2.8404257774	-0.3110132686	2.6321710508
H	3.9583159228	0.7704566893	3.4581929659

H	1.5624079127	1.2879069904	4.0460665277
H	2.3594103268	2.6476125239	3.2564077083
H	0.0894227139	2.2892867869	2.3168608902
H	0.4738831107	0.6153648592	1.9493949359
H	1.845865344	-0.740729526	0.6165752982
H	5.7433172365	-3.2591602871	-0.0021710995
Cl	6.9669288538	-3.0971684724	-2.5780910242
H	5.2142884839	-1.148089679	-3.7084823101
H	3.2509340117	0.0352752903	-2.7496719173
H	0.3946082367	0.2589154434	-0.8496950604
H	-3.3405608536	-3.6200587009	0.8291228063

IIIb(TBD)

C	-2.4782278397	-3.5972862235	-1.0254802423
C	-3.9839566894	-3.6692543118	-0.8210530455
H	-4.5099851567	-3.6147035626	-1.7824786361
C	-2.1085351452	-2.2192184967	-1.5446606072
N	-2.712829123	-1.2033214023	-0.6912541499
C	-3.8226192169	-1.3886646267	0.0292920661
N	-4.2800039586	-0.3629702526	0.7543286127
C	-5.4971046728	-0.4178953745	1.5555118016
C	-5.7593658136	-1.8522531029	1.9823785766
C	-5.7060190648	-2.7571116849	0.7618368762
N	-4.4355298387	-2.5836856068	0.0538204897
H	-2.297291002	-0.2585927942	-0.7001541648
H	-3.7153014542	0.4982153052	0.7657990382
H	-6.3447490707	-0.0324378847	0.976864952
H	-5.3543877847	0.2323551744	2.4207065539
H	-5.7710406973	-3.8070741311	1.055416006
H	-6.5404959645	-2.5426167809	0.0822669415
H	-2.4553061483	-2.095270252	-2.5773700879
H	-1.0262640307	-2.0594513455	-1.5282153582
H	-6.7412341134	-1.9302651931	2.4534126802
H	-5.0026175776	-2.1696307698	2.7064736278
O	-2.6215233225	1.9385433063	0.9660582331
C	-1.5981972857	1.9683491472	0.2656992429
O	-1.2911303684	1.170860961	-0.6662073989
C	-0.6067592763	3.112447559	0.597441672
N	0.4955394472	3.2055335794	-0.395381418
C	0.2477036316	4.3368912543	-1.3374662796
C	-1.1666469929	4.7934976331	-0.9964914822
C	-1.2759080848	4.4835271572	0.4949900731
C	1.5322484124	2.4394630427	-0.4430786847
H	0.3627358618	3.9832183969	-2.3617942691
H	0.9872730168	5.1148869043	-1.1298822902
H	-1.8982149713	4.2073307786	-1.5611167197
H	-1.3094773702	5.8484372162	-1.2322294345
H	-0.7162339443	5.2171670382	1.0820947973
H	-2.2991421223	4.4328592092	0.865681106
H	-0.1896778859	2.9265132712	1.588222519
C	2.6828000853	2.71391321	-1.3563515469
C	3.9047358181	3.0413333915	-0.4662874172
H	2.4820878038	3.5266655972	-2.0521606842
C	4.205078855	1.9056211033	0.5103289789

H	4.761247776	3.2336617783	-1.1172956037
H	3.6999137474	3.9657431935	0.0869492134
C	2.9792640945	1.5577520243	1.3584925937
H	4.5443795524	1.0272150977	-0.0500447693
H	5.0279310637	2.1934689645	1.1713055302
C	1.7202914246	1.2869825853	0.5042162072
H	3.168383535	0.6758929296	1.9794059231
H	2.7591266049	2.3892666458	2.0376937505
H	0.8555147569	1.192487113	1.1640033024
C	1.832462391	-0.0322708568	-0.3172260909
C	1.5601445874	-1.2204559651	0.5859772932
O	0.9866325004	-0.0074521556	-1.4438711329
H	2.8542941535	-0.1259895052	-0.6999651442
C	0.2558614174	-1.4795194722	1.0178454991
C	-0.0205277416	-2.5605949417	1.8467917229
C	1.0296059115	-3.3848410641	2.2439070168
C	2.3355532422	-3.1486479074	1.8354520723
C	2.5911941901	-2.0600736798	1.0026614756
H	-0.5562818829	-0.8274756842	0.7035439802
H	-1.0324303952	-2.7592994792	2.1849017957
Cl	0.6934550974	-4.752751912	3.284791251
H	3.1379735059	-3.8043688435	2.1552496597
H	3.6083453445	-1.8709018704	0.6693127716
H	2.8790880556	1.8050556741	-1.9351071119
H	-1.9681518301	-3.7861552747	-0.0752218806
H	-2.1686676279	-4.3633224118	-1.7392300447
H	-4.2619822203	-4.607455633	-0.3357337951
H	0.1382588012	0.4155152106	-1.1896404133

IIIc(TBD)

C	4.1651860152	-0.4340281514	-1.9410835228
C	3.2806100982	-0.835717114	-0.9368715994
C	3.4792977803	-2.0606050379	-0.3023550601
C	4.5495316925	-2.8786304777	-0.6597811648
C	5.4166807903	-2.454088069	-1.6582274333
C	5.2379444582	-1.2372935922	-2.3095245615
C	2.1434698891	0.0764535684	-0.539193946
H	1.6438592109	0.4096470378	-1.4585400719
H	2.7870163019	-2.3802918744	0.468552089
C	2.7048767353	1.3762667525	0.1667883039
C	1.4819182084	2.0968739585	0.6713107357
C	0.9870803927	1.8259535674	2.0643690321
C	2.1031328395	1.5531186161	3.0799363291
C	3.1226330364	0.5535893858	2.5476364412
C	3.7391417358	1.1113093878	1.2691623798
N	0.7857098343	2.8211115509	-0.1429335086
C	-0.5971063795	3.2695435442	0.1260529581
C	-0.875784554	4.2768177615	-0.9882614707
C	-0.1145216086	3.6798786301	-2.1702355814
C	1.1815201662	3.1889110011	-1.5347620944
C	-1.5632490482	2.0498453165	0.045615665
O	-1.069110846	0.9069272196	-0.1611840022
O	-2.7633976206	2.3391388234	0.1834495909
N	-4.6472423645	0.26827088	0.1693804155

C	-4.242194597	-0.9782605387	-0.0885045909
N	-5.1165489236	-1.9996081616	-0.109982304
C	-6.5565951964	-1.7517102315	-0.0099821585
C	-6.8264227318	-0.545441423	0.8747750586
C	-6.0437091269	0.6452686361	0.3491320198
C	-4.6866602182	-3.3479141527	-0.4882477535
C	-3.246693168	-3.5873776447	-0.0637910964
C	-2.3696542064	-2.4860268586	-0.6331745876
N	-2.9374760858	-1.1890868419	-0.286322831
H	-2.9143813203	-4.5607904099	-0.4300163797
H	-5.3583211088	-4.0503611984	0.0105136167
H	-4.7993519556	-3.4821839967	-1.571537839
H	-2.307585748	-0.3739283257	-0.2510895695
H	-3.9330591703	1.0146396057	0.157843191
H	-6.4665795202	0.9867414983	-0.6032845922
H	-6.0752675183	1.4806481778	1.0516099966
H	-7.0086041321	-2.6510043282	0.4141821635
H	-6.9776874803	-1.6009216094	-1.0121105495
H	-2.2942791084	-2.585613851	-1.7226639822
H	-1.3595082138	-2.5276306599	-0.2199586868
H	-7.8951229361	-0.3218033766	0.8741780343
H	-6.5208613918	-0.768762463	1.9015035065
H	1.9256977738	3.9866376279	-1.4549183068
H	1.618906174	2.3384326702	-2.0528994712
H	0.083126993	4.3971499608	-2.9673395124
H	-0.6675106196	2.8342135609	-2.5903627385
H	-0.4651059035	5.2539574639	-0.7186842114
H	-1.9481543131	4.3740249393	-1.1552433317
H	-0.6771169851	3.7106150181	1.1191217197
H	3.1814994824	1.9697777388	-0.6184542865
H	4.5084003415	0.4446806413	0.8704342391
H	4.2352400057	2.060906661	1.5032252075
H	2.6382833467	-0.4081606142	2.3472967266
H	3.9064129053	0.3852933955	3.2921218585
H	1.6369835243	1.196020616	4.0019867189
H	2.6120196447	2.4943081508	3.3185947318
H	0.3547288934	2.6446651827	2.4109023625
H	0.345092339	0.9400674158	1.9836413231
O	1.2229053312	-0.6171642036	0.2659437688
H	4.7054710713	-3.8330257682	-0.1684957254
Cl	6.7651165074	-3.4750005184	-2.1139747091
H	5.9218872333	-0.9275731409	-3.092005293
H	4.0141595436	0.5162466902	-2.4480105055
H	-3.1810082174	-3.5882118756	1.0284858894
H	0.3586220225	-0.1792441434	0.141235956

IIId(TBD)

C	-1.7116123626	-3.1920772787	-1.0859695799
C	-3.1915877296	-3.4913022349	-0.9088344939
H	-3.6846658039	-3.5980617135	-1.8831507767
C	-1.5532363768	-1.810163074	-1.6928104645
N	-2.299835139	-0.8484600422	-0.8875428294
C	-3.4178786558	-1.1564926494	-0.2167509142
N	-4.0719561443	-0.1718223388	0.4044931408

C	-5.3033701746	-0.3665327883	1.161412969
C	-5.3650150772	-1.7938475293	1.6768655871
C	-5.1093903591	-2.7524711939	0.5254458633
N	-3.8446281333	-2.4278742217	-0.1393649322
H	-2.0684071984	0.1497113086	-0.9731239363
H	-3.6417549028	0.7658352465	0.4087175864
H	-6.1702417056	-0.154534842	0.5248963786
H	-5.303151637	0.3524122402	1.9831427847
H	-5.0279741125	-3.7793394189	0.8886024867
H	-5.9296397874	-2.7136343905	-0.2022619899
H	-1.9244294234	-1.8046914597	-2.7249246416
H	-0.5093582098	-1.4885729361	-1.7040238406
H	-6.3467869083	-1.9950768677	2.1101122468
H	-4.608060386	-1.9434388241	2.4528602181
O	-2.7985818965	2.3026421378	0.7722619791
C	-1.7419420033	2.4388607824	0.135700887
O	-1.4040165868	1.8264200013	-0.9188465084
C	-0.7752817414	3.5025658819	0.7009269125
N	0.5850094696	3.4120405602	0.1022667038
C	0.8207106459	4.5452711019	-0.8376743219
C	-0.5705231156	5.1340970036	-1.0378815837
C	-1.2288353563	4.9179843408	0.3229555477
C	1.4411815606	2.4771946476	0.3461546101
H	1.2743517093	4.1657240157	-1.752526902
H	1.4979222382	5.2548811973	-0.3541325336
H	-1.1037745301	4.5748878266	-1.8116245353
H	-0.5198075387	6.1829244753	-1.3317586333
H	-0.8445141258	5.6362383306	1.0525494872
H	-2.3172627136	4.9835174552	0.3051751309
H	-0.7009752916	3.3687024813	1.7801458504
C	2.8515667964	2.5461797179	-0.1379745045
C	3.7812083088	2.5436198642	1.0950018671
H	3.035815782	3.4184999184	-0.7628983997
C	3.5568035499	1.294221826	1.9420267243
H	4.8144961589	2.5979404086	0.743060875
H	3.5902115397	3.444762168	1.689834821
C	2.089617117	1.1656700318	2.3541032743
H	3.8768065275	0.4125042335	1.377581784
H	4.1752464557	1.3398110584	2.8436642498
C	1.0844316591	1.2795790459	1.1854216229
H	1.9114597884	0.2283291716	2.8872585843
H	1.8512906718	1.9746005078	3.0553764903
H	0.0995371457	1.4290258319	1.627941423
C	0.9256549232	0.0316890669	0.2057422993
C	2.0123746062	-1.0157700814	0.2915268028
H	-0.0100865289	-0.4512110161	0.5254320683
O	0.8245438894	0.4520422604	-1.1350372608
C	2.0429560456	-1.9025733725	1.3711787768
C	3.0473476345	-2.85802508	1.4859961237
C	4.0166387452	-2.9327085605	0.4916330951
C	3.9891388891	-2.0908984444	-0.6137492537
C	2.9790431349	-1.1370501345	-0.7089603594
H	1.2693793885	-1.8533303489	2.1330741149
H	3.0701538896	-3.5418304007	2.3271967

Cl 5.2841621175 -4.1341428546 0.6236113545
H 4.7405337141 -2.1818317407 -1.3907922989
H 2.935198089 -0.486906716 -1.575791942
H 0.0356085379 1.0416101249 -1.1898383437
H 3.0376811795 1.6444754488 -0.7316297978
H -1.205344208 -3.2326779119 -0.115778093
H -1.2632036783 -3.9434109121 -1.7390249878
H -3.3344113665 -4.4213043419 -0.3542767626

TS1a(TBD)
C -3.8878066134 0.3242789774 -1.2546956654
C -3.303422039 0.2474464184 0.0143092191
C -3.9572400658 0.8070602116 1.1146867366
C -5.1919796097 1.4304087727 0.959962393
C -5.7569808421 1.4844728559 -0.3086404064
C -5.1198570825 0.9393188692 -1.4227409923
C -1.9734967589 -0.3704697264 0.190877063
O -1.2045664175 -0.4797246032 -0.8142124637
H -3.4930495864 0.7653265451 2.0965914013
C -2.781833636 -2.4645879544 0.669548645
C -1.4773070222 -2.9270234876 0.6191774998
C -0.6443448661 -3.0076349628 1.8790493227
C -1.4929444067 -3.175804471 3.1403756614
C -2.6382053661 -2.1695448905 3.1754679631
C -3.5475214798 -2.3779136714 1.9668483978
N -0.8596677076 -3.1551177047 -0.55776334
C 0.5541932491 -3.5025924776 -0.6958722809
C 0.6836711798 -3.9284268345 -2.1711954076
C -0.4215046504 -3.1325714303 -2.8639593637
C -1.5614053581 -3.1878674809 -1.8542061743
C 1.5072902774 -2.3427082526 -0.382587876
O 1.0423817678 -1.1302383552 -0.3266917218
O 2.6944672533 -2.603011133 -0.2202806064
N 4.66881363 -0.5972348997 0.3178555527
C 4.3425544975 0.6775631561 0.0836914711
N 5.2547094726 1.656043722 0.1906715391
C 6.669980416 1.3333905154 0.394555126
C 6.8093513443 0.0808219208 1.243988812
C 6.0263121955 -1.0502425725 0.6013434631
C 4.9183218164 3.0417601699 -0.1495379892
C 3.4537698534 3.3251020409 0.1423815555
C 2.5927265336 2.2927478246 -0.5637715278
N 3.0707463725 0.9579440822 -0.2213741818
H 3.2003821264 4.3280258931 -0.206501802
H 5.5621411825 3.685958766 0.4536209231
H 5.1471994497 3.2257553361 -1.2066430014
H 2.4084353275 0.182519156 -0.2579275147
H 3.945795366 -1.3035017286 0.1467518137
H 6.514065837 -1.3731308562 -0.325728726
H 5.9576983357 -1.9121546674 1.2677167375
H 7.1264497685 2.1907557149 0.893443566
H 7.1628614575 1.2028576118 -0.5769370105
H 2.632138833 2.4395899718 -1.6493452465
H 1.5497317398 2.3609070369 -0.2486576272

H 7.8630911402 -0.1930691415 1.3240295827
H 6.4259886762 0.2761747513 2.2500223129
H -2.1350541265 -4.12030831 -1.9332210962
H -2.2404461402 -2.3416391587 -1.94742925
H -0.705691566 -3.5485362631 -3.8315842814
H -0.1106131473 -2.092364628 -3.0083675173
H 0.4822558135 -5.0003885338 -2.2470425337
H 1.6850242408 -3.7360146214 -2.5594065692
H 0.8406624717 -4.3209745344 -0.0321144495
H -3.3846149127 -2.5281475174 -0.2307542936
H -4.2924689317 -1.5771502519 1.8992392882
H -4.1210741876 -3.3056951247 2.1030932851
H -2.2285811713 -1.1508140129 3.1670777694
H -3.2142476316 -2.2751280791 4.0996088849
H -0.8488285725 -3.0709764106 4.0180030467
H -1.90592307 -4.1916286468 3.1620128117
H 0.0696416647 -3.8315078965 1.8035232951
H -0.0479562472 -2.085221661 1.9485913605
H -1.5411622687 -0.299061122 1.1963201829
H -5.703278141 1.8708188002 1.8085531047
Cl -7.3067286434 2.2662604263 -0.5165159034
H -5.5822925563 1.0022256654 -2.4015797138
H -3.3672031143 -0.0972966909 -2.1091008374
H -0.0573828669 -0.8910061874 -0.5613590071
H 3.2780505134 3.2798757403 1.221401591

TS1b(TBD)
C 1.52908225 3.2463301501 0.4947160192
C 1.1515304975 1.9040845897 0.6018356926
C -0.1501408548 1.5300323672 0.2487581847
C -1.0563930773 2.4761616761 -0.2109521645
C -0.6537724881 3.8066254217 -0.3073775013
C 0.6305496216 4.2069660707 0.040694954
C 2.1472713712 0.9394450555 1.1380980793
O 1.8309963735 -0.1358528861 1.7342014332
Cl -1.7957431436 5.0021055866 -0.8759022286
C 2.9705076744 0.371682223 -0.8819139239
C 3.6412135986 -0.6660823975 -0.2552993978
C 4.9970647259 -0.4247825012 0.3612854081
C 5.7863334339 0.6564809526 -0.3829649817
C 4.953583678 1.9146399229 -0.6102412211
C 3.6974689626 1.5831237823 -1.415354981
N 3.044746126 -1.8533612719 -0.0270264358
C 1.9960657732 -2.4123792479 -0.8843499302
C 2.2218791816 -3.9301937452 -0.7384128966
C 2.6321084245 -4.0507195978 0.729139326
C 3.5837320316 -2.8690221653 0.8996166986
C 0.543474491 -2.1235418835 -0.487441966
O 0.2765958834 -1.6368679808 0.6931607026
O -0.3387595454 -2.4520340922 -1.2699708641
N -3.1676726147 -1.9269448597 -0.9014850414
C -3.4879112473 -1.2088661332 0.1783496071
N -4.7324521381 -0.7467556686 0.3605829989
C -5.8111789249 -1.1119452299 -0.5627187257

C	-5.2640816288	-1.3190513755	-1.9656892733
C	-4.1308949737	-2.3297251269	-1.9210317228
C	-5.1001903676	-0.0682009685	1.6073606259
C	-3.9220675476	0.7275928217	2.1464381619
C	-2.72412643	-0.1921079077	2.3026292068
N	-2.5181392208	-0.9263332506	1.0582992561
H	-4.1852600365	1.1631365487	3.112222444
H	-5.9386814153	0.5915326973	1.3758232114
H	-1.6038524941	-1.3494710155	0.9038801226
H	-2.1844187158	-2.1842149725	-1.0155696169
H	-4.5180233143	-3.3297558633	-1.69517084
H	-3.6030011539	-2.3756313568	-2.8749307156
H	-6.536136045	-0.2950163944	-0.5473467948
H	-6.3117151103	-2.018715906	-0.2015284187
H	-6.0617856787	-1.6790605817	-2.6182827106
H	-4.8975148893	-0.3669857196	-2.3612863007
H	3.6121769093	-2.4687901767	1.9158567626
H	4.5974002942	-3.1551428893	0.601034004
H	1.758322856	-3.9364215461	1.3780935279
H	3.1174454704	-4.9988378884	0.9642419052
H	3.0468244141	-4.2289797315	-1.3914804526
H	1.3331130749	-4.5026254305	-1.0054787735
H	2.1231533322	-2.0654295395	-1.9122686672
H	5.5714322713	-1.3537705184	0.3741138971
H	6.6943177128	0.8870612488	0.1816007143
H	6.1039124275	0.256197455	-1.3535362957
H	4.6766865145	2.3473124618	0.359211045
H	5.5434263203	2.6705155239	-1.1374341015
H	3.015357309	2.4401391491	-1.4368064763
H	3.9767216835	1.3968726297	-2.461737337
H	2.0077740007	0.1495996915	-1.3403161481
H	3.0886024301	1.4111744997	1.4442110018
H	-0.4610409062	0.4942894925	0.3204067319
H	-2.062596867	2.1876545971	-0.496044896
H	0.9221139808	5.2484330596	-0.0364292794
H	2.5343691424	3.546488415	0.7796258823
H	1.032122755	-0.97217026	1.1757125271
H	4.846280741	-0.1388842631	1.4132379836
H	-1.8114906352	0.3739451264	2.5072102993
H	-2.8853380137	-0.8943057669	3.1282938577
H	-3.6811700284	1.5420730404	1.4561775847
H	-5.4412172325	-0.8071391468	2.3428142225

TS1c(TBD)

C	4.2210726846	-0.0858223062	-1.0638921604
C	3.2219732236	-0.2781164891	-0.1030989388
C	3.4176206633	-1.193570745	0.9359289695
C	4.6063258097	-1.9066646669	1.0235373013
C	5.5874563608	-1.6960014812	0.0585907855
C	5.4107518244	-0.7963013258	-0.9909404359
C	1.9742833736	0.4826479035	-0.202945699
H	1.7772672872	1.006994129	-1.1411957534
H	2.6323488745	-1.3502460766	1.6681560078
C	2.9205255551	2.5743699192	0.4258390383

C	1.6224370237	2.9425142929	0.7183824889
C	1.0898300323	2.8210730336	2.1231727642
C	2.188287938	2.8040168711	3.1868501293
C	3.307396374	1.8449810209	2.8012587034
C	3.9489277515	2.3028866822	1.4944944029
N	0.7403855775	3.2742287421	-0.258977067
C	-0.6749302903	3.5802127111	-0.0606051123
C	-1.0063250357	4.4985086771	-1.2453036995
C	-0.1232084813	3.9346858044	-2.3568073612
C	1.1770893199	3.61152106	-1.6257466957
C	-1.5992167482	2.3471857201	-0.0580308303
O	-1.1010290375	1.1558793645	-0.0454471274
O	-2.8114954342	2.5595623427	-0.0737907314
N	-4.7256762266	0.4589615569	0.2022924971
C	-4.2993770892	-0.8008444482	0.0717341443
N	-5.1570452687	-1.8320204281	0.1328086536
C	-6.6025995309	-1.5958076071	0.1803741678
C	-6.9042126701	-0.3177029206	0.9459422987
C	-6.1300509118	0.8327412259	0.3268346267
C	-4.6999001735	-3.2045291098	-0.1029545564
C	-3.264780712	-3.3778790046	0.3673180804
C	-2.3920525602	-2.3240788169	-0.2912559936
N	-2.9880414167	-1.0095393831	-0.0838255149
H	-2.9122497291	-4.3767936949	0.1030112523
H	-5.3692150345	-3.8658686247	0.4518189773
H	-4.7914491518	-3.4451464372	-1.1695672693
H	-2.3736256675	-0.1914046074	-0.0944144005
H	-4.0265267916	1.2041175422	0.1002850992
H	-6.5403064777	1.0810968382	-0.6589660663
H	-6.1827742776	1.7263061147	0.9518463906
H	-7.0534173535	-2.4582083878	0.6762619935
H	-7.0024992255	-1.5427775913	-0.8399999602
H	-2.2928877021	-2.5272595996	-1.3640028416
H	-1.391406629	-2.3056264295	0.145238135
H	-7.9755086067	-0.1110258151	0.9070778072
H	-6.6143198341	-0.441749558	1.9937340152
H	1.851874423	4.4762507067	-1.5834489139
H	1.7210009524	2.7853983051	-2.0907143181
H	0.0340646498	4.6311322925	-3.1816027214
H	-0.5645541498	3.0162292886	-2.7588914961
H	-0.7023500356	5.5203197847	-1.0006040031
H	-2.0722926784	4.4887627034	-1.4703351351
H	-0.8502645946	4.0923884717	0.8880352436
H	3.3007352831	2.754664548	-0.5756301663
H	4.658823392	1.5497975476	1.1307586376
H	4.5407030871	3.2115732245	1.676252934
H	2.8886608254	0.838705035	2.6733403362
H	4.0603810559	1.7866751585	3.5928839002
H	1.7453744445	2.5278780525	4.1480130197
H	2.599224069	3.8147312205	3.2990951939
H	0.3952650029	3.6391361555	2.3288295264
H	0.5020915016	1.8914756474	2.1745870315
O	0.9907440523	0.1925709567	0.5485195812
H	4.7702081891	-2.6189305939	1.8243634026

Cl	7.0830796058	-2.589907664	0.1639902374
H	6.1906699849	-0.6567316582	-1.7309527118
H	4.0653795638	0.6284591364	-1.8682799308
H	-3.2191516494	-3.2717149892	1.4553575043
H	0.0173144193	0.7657966724	0.2470493325

TS1d(TBD)

C	-1.8502770634	-3.6014867715	-1.4454208702
C	-3.236777663	-4.0141014476	-0.9783920711
H	-3.9139504209	-4.1380825746	-1.8325039448
C	-1.9170106081	-2.2013169732	-2.0277240792
N	-2.5540260705	-1.3135621148	-1.0606763631
C	-3.4846193561	-1.7173509625	-0.1839157407
N	-4.0679675503	-0.7937289495	0.5834450812
C	-5.113639462	-1.0912029673	1.5561382025
C	-4.9531784836	-2.5194547375	2.0470670914
C	-4.8535026515	-3.4536827213	0.852321509
N	-3.7875634401	-3.0174836818	-0.0547096902
H	-2.421072497	-0.3043632383	-1.1708088236
H	-3.7321923835	0.1728131474	0.4997566324
H	-6.0995903276	-0.9529844464	1.0979637814
H	-5.0159915862	-0.3766267973	2.3752982046
H	-4.6051629427	-4.4672683477	1.1745295964
H	-5.805078381	-3.4928971202	0.3079548375
H	-2.4822631878	-2.2038540698	-2.9668852032
H	-0.9180873789	-1.8100736041	-2.2357026191
H	-5.8096257574	-2.8036185923	2.6615497113
H	-4.0480856844	-2.60412058	2.6561654999
O	-2.9001884491	1.8009235446	0.6310273396
C	-1.9544302269	2.0446639291	-0.1177415812
O	-1.729372063	1.409486375	-1.2230004868
C	-1.0538459898	3.2357368076	0.2302159273
N	0.3253140143	3.1465371772	-0.2594302928
C	0.5861782572	4.1074469159	-1.3463213645
C	-0.8151405926	4.5101466739	-1.7930580074
C	-1.5964622444	4.4931213119	-0.4786845906
C	1.2924636588	2.4668607605	0.3958331844
H	1.1779413337	3.6383548367	-2.1353982793
H	1.1322734418	4.9784747417	-0.9641418245
H	-1.2205165096	3.7651598702	-2.4837284483
H	-0.8233505557	5.4851701936	-2.2826071439
H	-1.3487326912	5.3733976874	0.1213625788
H	-2.6799669796	4.4557537198	-0.6079722694
H	-1.0614509088	3.3363842377	1.3183742808
C	2.7248735477	2.771701301	0.0422698611
C	3.6661214721	2.496466066	1.2175307578
H	2.818599971	3.8121655719	-0.2765262759
C	3.476520045	1.0823652746	1.7563817672
H	4.6997413707	2.6503506345	0.8942115542
H	3.4639996033	3.225451837	2.0118660198
C	2.0313098626	0.8563670664	2.2062277457
H	3.7446179561	0.3650546821	0.9732229342
H	4.1522345666	0.901394698	2.5979972219
C	1.0013298022	1.4362548122	1.2677742492

H	1.8453327573	-0.214617384	2.3494891218
H	1.8833540379	1.3136703647	3.1950293093
H	-0.0364822359	1.2863420583	1.5600826099
C	0.7529035814	0.023948932	-0.6754907395
C	1.9887298003	-0.7508745304	-0.6275968692
H	-0.0899943391	-0.3377442698	-0.0791134317
O	0.5202501395	0.786347173	-1.6728214634
C	2.1140329847	-1.7813496145	0.3108937557
C	3.283718182	-2.5272820029	0.3814666994
C	4.3152761379	-2.2356811894	-0.5069735929
C	4.2000303383	-1.2366908332	-1.4726773771
C	3.0279646645	-0.4970542265	-1.5331145839
H	1.2905373741	-2.0001915436	0.9848063876
H	3.3937728114	-3.3248023778	1.1077664187
Cl	5.7903149798	-3.1650897938	-0.4270471999
H	5.0135852969	-1.0489587749	-2.1642176753
H	2.9080922551	0.2759252448	-2.2851777539
H	-0.5252348501	1.2019744062	-1.5418511657
H	3.003852634	2.1509204119	-0.8228792859
H	-1.1543114923	-3.619675488	-0.6005421324
H	-1.4942156122	-4.3051520932	-2.2002101817
H	-3.1965632417	-4.9621836451	-0.4379434494

S6. References

1. Yu, L.-J.; Blyth, M.T.; Coote, M.L. Re-Examination of Proline-Catalyzed Intermolecular Aldol Reactions: An Ab Initio Kinetic Modelling Study, *Top. Catal.* **2022**, *65*, 354–365. DOI: 10.1007/s11244-021-01501-5.
2. Chankeshwara S.V.; Chakraborti, A. K. Catalyst-Free Chemoselective N-tert-Butyloxycarbonylation of Amines in Water, *Org. Lett*. **2006**, *8*, 3259-3262. DOI: 10.1021/ol0611191.
3. Barešić, L.; Margetić, D.; Glasovac, Z. Anion-controlled synthesis of novel guanidine-substituted oxanorbornanes. *Int. J. Mol. Sci*. **2022**, *23*, 16036. DOI: 10.3390/ijms232416036.
4. Aoyagi, N.; Endo, T. Synthesis of five- and six-membered cyclic guanidines by guanylation with isothiouronium iodides and amines under mild conditions. *Synth. Commun*. **2017**, *47*, 442–448.
5. Maji, S. K.; Banerjee, R.; Velmurugan, D.; Razak, A.; Fun, H. K.; Banerjee, A. Peptide Design Using ω-Amino Acids: Unusual Turn Structures, Nucleated by an N-Terminal Single γ-Aminobutyric Acid Residue in Short Model Peptides, *J. Org. Chem*. **2002**, *67*, 633-639. DOI: 10.1021/jo010314k.
6. Saroj, S.; Janni, D. S., Reddy, U.C.; Muraleedharan, K.M. Functionalizable oxanorbornane-based head-group in the design of new Non-ionic amphiphiles and their drug delivery properties. *M. Sci. Eng. C* **2020**, *112*, 110857. DOI: 10.1016/j.msec.2020.110857.
